# Immunogenicity and Safety of ChAdOx1 nCoV-19 (AZD1222) as a Homologous Fourth-Dose Booster: A Substudy of the Phase 3 COV003 Trial in Brazil

**DOI:** 10.1016/j.mayocpiqo.2025.100642

**Published:** 2025-07-11

**Authors:** Sue Ann Costa Clemens, Sagida Bibi, Natalie G. Marchevsky, Parvinder K. Aley, Federica Cappuccini, Sophie A. Davies, Isabela Gonzalez, Sarah C. Kelly, Yama F. Mujadidi, Eveline Pipolo Milan, Alexandre V. Schwarzbold, Eduardo Sprinz, Merryn Voysey, Lily Y. Weckx, Daniel Wright, Himanshu Bansal, Maria A.S. Bergagård, Abby J. Isaacs, Elizabeth J. Kelly, Dongmei Lan, Shethah Morgan, Nirmal Kumar Shankar, Kathryn Shoemaker, Tonya L. Villafana, Teresa Lambe, Justin A. Green, Andrew J. Pollard

**Affiliations:** a Oxford Vaccine Group, Department of Paediatrics, University of Oxford, and the NIHR Oxford Biomedical Research Centre, United Kingdom; b Institute for Global Health, University of Siena, Italy; c Centro de Estudos e Pesquisas em Moléstias Infec, CePCLIN – Centro de Pesquisas Clínicas de Natal, Natal, Rio Grande do Norte, Brazil; d Clinical Research Unit, Department of Clinical Medicine, Universidade Federal de Santa Maria, Brazil; e Hospital de Clínicas de Porto Alegre, Universidade Federal do Rio Grande do Sul, Brazil; f Department of Pediatrics, Universidade Federal de São Paulo, Brazil; g Biometrics, Vaccines & Immune Therapies, BioPharmaceuticals R&D, AstraZeneca, Gaithersburg, MD; h Translational Medicine, Vaccines & Immune Therapies, BioPharmaceuticals R&D, AstraZeneca, Gaithersburg, MD; i Clinical Development, Vaccines & Immune Therapies, BioPharmaceuticals R&D, AstraZeneca, Gaithersburg, MD; j Clinical Operations, Vaccines & Immune Therapies, BioPharmaceuticals R&D, AstraZeneca, Gothenburg, Sweden; k Clinical Development, Vaccines & Immune Therapies, BioPharmaceuticals R&D, AstraZeneca, Cambridge, United Kingdom; l Patient Safety, Vaccines & Immune Therapies, BioPharmaceuticals, AstraZeneca, Bangalore, India; m Chinese Academy of Medical Science, Oxford Institute, University of Oxford, United Kingdom; n Currently at Global Immunology, Vaccines R&D, Sanofi, Swiftwater, PA

## Abstract

**Objective:**

To address that, despite widespread use of ChAdOx1 nCoV-19 (AZD1222) as a COVID-2019 booster, fourth-dose clinical outcomes data are limited. We report immunogenicity and safety for ChAdOx1 nCoV-19 as a homologous fourth-dose booster.

**Participants and Methods:**

Participants (aged ≥18 years) who had received 2 doses of ChAdOx1 nCoV-19 in phase 3 COV003 trial in Brazil were offered a third dose after a planned dose interval from 11 to 13 months and a fourth dose after a planned interval from 6 to 15 months (both 5 × 10^10^ viral particles). All fourth doses were administered to substudy participants between August 18 and October 28, 2022. The data cutoff was December 9, 2022. The primary immunogenicity outcome was noninferiority of ancestral severe acute respiratory syndrome coronavirus (SARS-CoV)-2–neutralizing antibody responses 28 days after dose 4 versus dose 3. Solicited and unsolicited adverse events were recorded 7 and 28 days postdose 4, respectively.

**Results:**

172 participants received a fourth dose (median interval postthird dose, 10.7 months). Ancestral SARS-CoV-2–neutralizing antibody titers postdose 4 were noninferior to those postdose 3; geometric mean fold rise was 1.9 (95% CI, 1.6-2.4; n=112). Immunogenicity results were consistent across all variants analyzed. Local and systemic solicited adverse events were reported in 60.3% (n=35/58) and 43.1% (n=25/58) of participants, respectively.

**Conclusion:**

Immune responses after a fourth dose of ChAdOx1 nCoV-19 were noninferior to those after a third dose across SARS-CoV-2 variants. The fourth dose was well tolerated with no emergent safety concerns, supporting the continued development of the ChAdOx1 platform in preparation for future pandemics.

**Trial Registration:**

clinicaltrials.gov Identifier: NCT04536051

COVID-19 vaccines saved ∼19.8 million lives during the first year of rollout.^1^ ChAdOx1 nCoV-19 (AZD1222), an adenoviral-vectored vaccine using the ChAdOx1 platform,^2^^,^^3^ was one of the most used vaccines, with >3 billion doses distributed in >180 countries and ∼6.3 million deaths averted.^1^^,^^4^ Following initial COVID-19 vaccine shortages, ChAdOx1 nCoV-19 became a key component of Brazil’s pandemic response owing to a technology transfer allowing for local manufacture by the Oswald Cruz Foundation (Fiocruz).^5^^,^^6^

The phase 3 COV003 study evaluated the safety, efficacy, and immunogenicity of a 2-dose ChAdOx1 nCoV-19 primary series in Brazilian adults and was 1 of the 4 global studies that, together, established that initial vaccine efficacy against symptomatic COVID-19 was 62.1%.^2^^,^^3^ Over the course of COV003, booster vaccination strategies were used to reduce breakthrough infections.^7^, ^8^, ^9^, ^10^ Consequently, study amendments were allowed for the provision of third-dose and fourth-dose boosters.

Although an end to the COVID-19 public health emergency has been declared, booster vaccinations remain recommended for the elderly, immunocompromised, and individuals with underlying comorbidities.^11^ Thus, ChAdOx1 nCoV-19 was widely used as a fourth dose, including in Brazil. Additionally, the ChAdOx1 vaccine platform is in development as a rapid response technology for future pandemic threats.^12^^,^^13^ Therefore, although ChAdOx1 nCoV-19 has had marketing authorization withdrawn owing to the availability of variant-updated COVID-19 vaccines, data on ChAdOx1 nCoV-19 boosters are still needed to inform future vaccination strategies and evaluate the potential of the ChAdOx1 platform. However, clinical data on homologous ChAdOx1 nCoV-19 boosters are limited.^14^, ^15^, ^16^ To address this, we report on immunogenicity and safety from a cohort of COV003 participants who received ChAdOx1 nCoV-19 third and fourth doses.

## Participants and Methods

### Study Design

**Figure 1:**
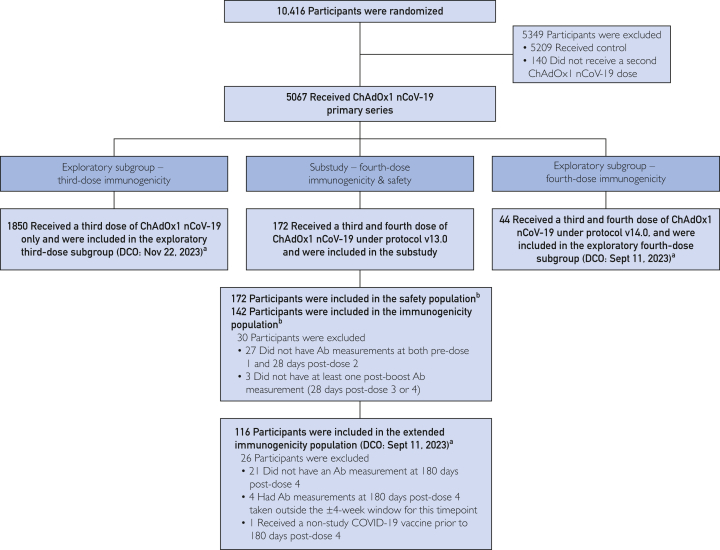
Participant disposition. Flow chart showing the number of participants randomized and vaccinated with ChAdOx1 nCoV-19 primary series or control; the number of participants vaccinated with third and fourth doses of ChAdOx1 nCoV-19 in the substudy; and the number of participants included in the substudy and exploratory subgroup analysis populations. DCO: December 9, 2022, unless otherwise specified. ^a^Analyses in these populations were performed by The University of Oxford. ^b^Analyses in these populations were performed by AstraZeneca. COVID-19, coronavirus disease 2019; DCO, data cutoff.

The phase 3 COV003 study (ClinicalTrials.gov Identifier: NCT04536051) began assessing the safety, efficacy, and immunogenicity of ChAdOx1 nCoV-19 across 6 sites (São Paulo, Rio de Janeiro, Salvador, Natal, Santa Maria, and Porto Alegre) on June 23, 2020. ChAdOx1 nCoV-19 primary series data, the statistical analysis plan, and a previous study protocol (https://www.thelancet.com/journals/lancet/article/PIIS0140-6736(20)32661-1/fulltext#supplementary-material), have been previously published.^2^^,^^3^ In this study, we report data from participants who received ChAdOx1 nCoV-19 third and fourth doses under updated protocols (Supplemental Appendix, available online at http://www.mcpiqojournal.org) (Figure 1).

The initial COV003 study protocol and subsequent amendments were approved by the Oxford Tropical Research Ethics Committee (OxTREC; September 13, 2022) in the United Kingdom and by the Comissão Nacional de Ética em Pesquisa (CONEP; November 9, 2022) in Brazil.^2^^,^^3^ This study was performed in accordance with ethical principles originating in the Declaration of Helsinki and consistent with International Council for Harmonisation Good Clinical Practice, applicable regulatory requirements, and AstraZeneca’s policy on Bioethics.

### Participants

COV003 enrolled adults (≥18 years old) at high risk of severe acute respiratory syndrome coronavirus (SARS-CoV)-2 exposure, particularly health care workers.^2^^,^^3^^,^^17^ The substudy included participants who received ChAdOx1 nCoV-19 third and fourth doses under protocol v13.0 (Supplemental Appendix). Participants with previous SARS-CoV-2 infection (self-reported and confirmed via reverse transcription polymerase chain reaction or rapid lateral flow testing) or nonstudy COVID-19 vaccinations were excluded. Separate exploratory subgroups were assessed by The University of Oxford and included individuals who received a ChAdOx1 nCoV-19 third dose only and individuals who received a fourth dose under a later protocol (protocol v14.0; Supplemental Appendix), allowing for previous SARS-CoV-2 infection (Exploratory subgroup participants, Supplemental Appendix) (Figure 1). All participants provided written informed consent.

**Figure 2:**
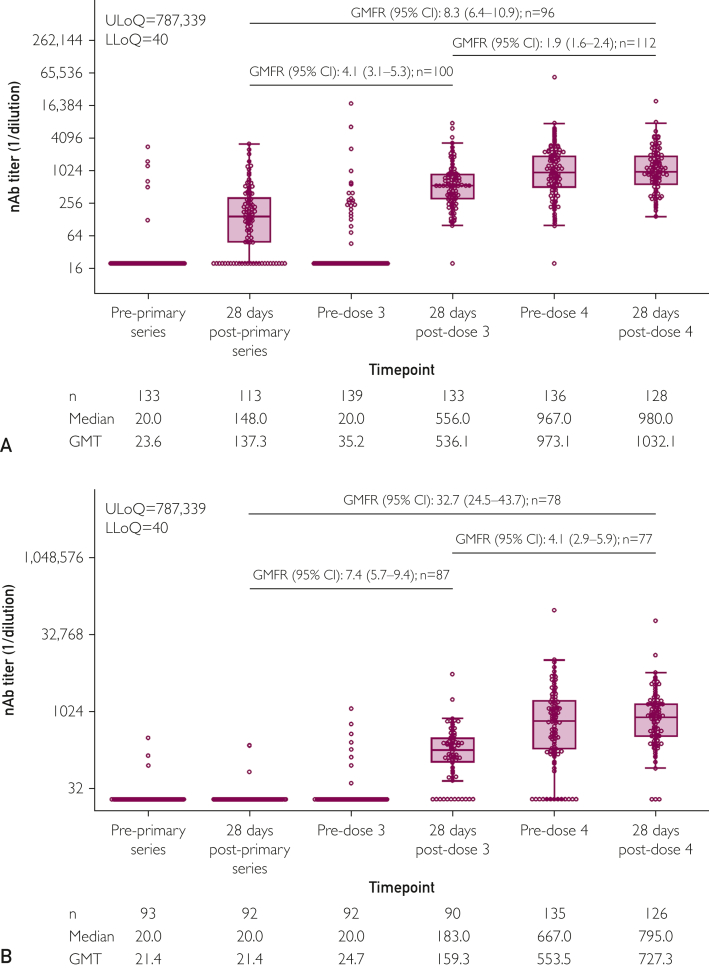
nAb titers against (A) ancestral SARS-CoV-2 and (B) Omicron BA.4/5 before and 28 days postprimary series, third dose, and fourth-dose of ChAdOx1 nCoV-19. nAb titers were assessed before and 28 days postprimary series (dose 2), dose 3, and dose 4 of ChAdOx1 nCoV-19 in the immunogenicity population. The bottom and top edges of the box indicate the first and third quartiles (the difference is the IQR), and the line inside the box is the median. Whiskers extend to the minimum and maximum values, excluding outliers. Any points with a log-transformed value of more than 1.5× IQR from the respective edges of the box were considered outliers. Boxplots were created based on the log-normal distribution. GMFRs were calculated in participants with Ab measurements at both compared time points; for a full listing of GMTs associated with the provided GMFRs, see Supplemental Table 1. CI, confidence interval; GMFR, geometric mean fold rise; GMT, geometric mean titer; LLoQ, lower limit of quantification; nAb, neutralizing antibody; SARS-CoV, severe acute respiratory syndrome coronavirus; ULoQ, upper limit of quantification.

### Randomization and Masking

As previously described, participants were initially masked and randomized 1:1 to receive either a ChAdOx1 nCoV-19 primary series or control.^2^^,^^3^^,^^17^ Following ChAdOx1 nCoV-19 emergency use authorization in Brazil on January 17, 2021, 99.2% of COV003 study participants (N=10,416) were unblinded, but continued to attend follow-up visits.^17^ Therefore, all participants in these analyses were unblinded and received treatment.

### Procedures

Previously, substudy participants received 2 ChAdOx1 nCoV-19 (3.5-6.5 × 10^10^ viral particles [vp]) doses via intramuscular injection 4 to 12 weeks apart.^2^^,^^3^^,^^17^ Substudy participants received a ChAdOx1 nCoV-19 third dose (5 × 10^10^ vp) 11 to 13 months after the second dose, then a ChAdOx1 nCoV-19 fourth dose (5 × 10^10^ vp) 6 to 15 months later, both via intramuscular injection.

Serum samples were collected at the following time points: predose 1, 28 days postdose 2, predose 3, 28 days postdose 3, predose 4, 28 days postdose 4, and 180 days postdose 4. For participants who were dosed under protocol v13.0, analyses up to 28 days postdose 4 were performed by AstraZeneca, the rest by The University of Oxford. Serological assessments included neutralizing antibody (nAb) titers against ancestral SARS-CoV-2 and Omicron BA.4/5, antispike antibody (Ab) titers against multiple SARS-CoV-2 variants (ancestral, Alpha, Beta, Gamma, Delta, and Omicron BA.1), and anti-SARS-CoV-2 nucleocapsid Ab titers. Anti-ChAdOx1 nAbs titers were also measured. Serological assessment procedures are described in the Supplemental Appendix.

Predefined local and systemic solicited adverse events (AEs) (Supplemental Appendix) were collected for 7 days postfourth dose in a subset of substudy participants via participant diaries or investigator case report forms. Unsolicited AEs were collected for 28 days postfourth dose in all substudy participants. Unsolicited serious adverse events (SAEs) and adverse events of special interest (AESIs) were collected through 180 days postfourth dose, or DCO, whichever occurred first. Prespecified AESI categories included disorders that were neurologic, potentially immune mediated, vascular, or hematologic in nature.^3^ Unsolicited AEs, SAEs, and AESIs were classified as related to study intervention by the investigator.

### Outcomes

The primary substudy immunogenicity outcome was noninferiority of ancestral SARS-CoV-2 nAb responses at 28 days postfourth dose versus 28 days postthird dose. Secondary immunogenicity analyses included noninferiority assessments of nAb and antispike Ab responses against several SARS-CoV-2 variants at 28 days postfourth dose versus 28 days postthird dose or primary series vaccination. Exploratory immunogenicity outcomes are in the Supplemental Appendix).

Safety analyses included incidence of solicited AEs, incidence of unsolicited AEs, and incidence of unsolicited SAEs and AESIs within 7, 28, and 180 days of dosing, respectively.

### Statistical Analyses

Planned enrollment was 350 participants (±10%) to provide >99% power for noninferiority of postfourth dose versus postthird dose humoral responses. The immunogenicity population comprised all substudy participants who received a ChAdOx1 nCoV-19 fourth-dose and had baseline (preprimary series), 28 days postprimary series (postsecond dose), and either 28 days postthird dose or postfourth dose Ab measurements. An extended immunogenicity population includes those who also had 180 days postdose 4 (±4 weeks) Ab measurements. For all noninferiority analyses, the noninferiority margin for the lower bound of the 95% CI of the geometric mean fold rise (GMFR) was defined as two-thirds (hereafter, referred to as 0.67). Spearman correlation coefficients were used to assess the relationship between prethird and prefourth dose anti–ChAdOx1 nAb levels and anti–SARS-CoV2 responses at 28 days after each dose. Immunogenicity was summarized descriptively as median values, first and third quartiles, interquartile ranges (IQRs), geometric mean titers (GMTs), and GMFRs.

The safety population comprised all substudy participants who received a ChAdOx1 nCoV-19 fourth dose. Solicited AEs were to be collected for 100 participants to provide >99% probability that at least 1 solicited AE would be observed, assuming a true rate of solicited AEs of 5%. Unsolicited AEs were collected for the full safety population. Frequencies of AEs were reported using descriptive statistics. Nonstudy COVID-19 vaccination was treated as an intercurrent event; all subsequent participant data were excluded from analyses.

## Results

### Participants

A total of 10,416 adults (≥18 years) were enrolled and randomized in COV003 between June 23 and December 1, 2020 (Figure 1); 5067 received a primary series of ChAdOx1 nCoV-19. In this substudy, 172 participants received a third dose after a median of 11.1 months (range, 10.9-12.8 months) and a fourth dose after a similar interval (median, 10.7 months [range, 9.7-13.4 months]). All fourth doses were administered between August 18 and October 28, 2022. DCO was December 9, 2022, unless otherwise specified (Figure 1).

The safety population included all substudy participants (Figure 1). The immunogenicity population comprised 142 participants, of whom 116 had Ab measurements at 180 days postfourth dose and were also included in the exploratory extended immunogenicity population.

Safety and immunogenicity population baseline (preprimary series) demographic characteristics were similar (Supplemental Table 1, available online at http://www.mcpiqojournal.org). Most safety population participants (94.8%, n=163) were <65 years; median age was 36.8 years (range, 20.5-78.3 years). Immediately before fourth dose administration, 32.6% (n=56) of participants were seropositive for SARS-CoV-2 infection (antinucleocapsid Ab threshold ≥19,904 AU/mL).

COV003 participants were included in 2 exploratory subgroups. One exploratory third dose subgroup (n=1850) received a ChAdOx1 nCoV-19 third dose only. Another exploratory subgroup (n=44) received a fourth dose under a protocol amendment allowing for previous SARS-CoV-2 infection (Exploratory subgroup participants, Supplemental Appendix) (Figure 1).

### Immunogenicity

The primary immunogenicity outcome was met; nAb titers against ancestral SARS-CoV-2 measured 28 days postfourth dose were noninferior to those measured 28 days postthird dose (ie, the lower bound of the 95% CI of the GMFR between time points was >0.67). In participants who had Ab measurements at both time points (n=112), GMTs increased from 545.0 (95% CI, 461.7-643.3) to 1048.0 (95% CI, 891.4-1232.1; GMFR, 1.9 (95% CI, 1.6-2.4) (Figure 2A; Supplemental Table 2, available online at http://www.mcpiqojournal.org).

Additionally, both nAb and antispike Ab responses recorded postfourth dose were noninferior to those recorded postthird dose for all SARS-CoV-2 variants analyzed (Figure 2, Supplemental Figures 1 and 2, available online at http://www.mcpiqojournal.org; Supplemental Table 2). Postfourth dose nAb titers against Omicron BA.4/5 were also noninferior versus postthird dose titers, and responses were also numerically higher than those against ancestral SARS-CoV-2; in 77 participants with available Ab measurements, GMTs increased from 170.7 (95% CI, 133.7-217.9) to 702.2 (95% CI, 529.2-931.7) and GMFR was 4.1 (95% CI, 2.9-5.9) (Figure 2B; Supplemental Table 2). Antispike Ab titers against ancestral SARS-CoV-2 and Omicron BA.1 also increased postfourth dose; GMFRs were 1.6 (95% CI, 1.3-1.9; n=127) (Supplemental Figure 1A; Supplemental Table 2) and 1.7 (95% CI, 1.4-2.0; n=127) (Supplemental Figure 1B; Supplemental Table 2), respectively.

Postbooster humoral responses were also noninferior to those recorded postprimary series (postdose 2) (Figure 2; Supplemental Figures 1 and 2; Supplemental Table 2). For example, ancestral SARS-CoV-2 antispike Ab GMFRs from postsecond dose were 3.0 (95% CI, 2.4-3.6; n=138) to postthird dose and 4.9 (95% CI, 4.1-5.8; n=129) to postfourth dose.

nAb and antispike Ab titers measured 28 days after dosing were compared with those of predosing titers. Overall, the increase in humoral responses was smaller for the fourth dose versus the third dose; for nAbs against ancestral SARS-CoV-2, GMTs increased from 35.2 (95% CI, 28.5-43.6; n=139) predose 3 to 536.1 (95% CI, 460.8-623.6; n=133) postdose 3 and from 973.1 (95% CI, 811.5-1166.8; n=136) predose 4 to 1032.1 (95% CI, 889.7-1197.4; n=128) postdose 4 (Figure 2A). A similar pattern was observed for both Omicron BA.4/5 nAb (Figure 2B) and SARS-CoV-2 and Omicron BA.1 antispike Ab titers (Supplemental Figure 1). Before fourth dose administration, we found that, in addition to high rates of SARS-CoV-2 seropositivity, participants also had high levels of immunity against SARS-CoV-2 (Figure 2; Supplemental Figure 1).

### Exploratory Analyses

**Figure 3:**
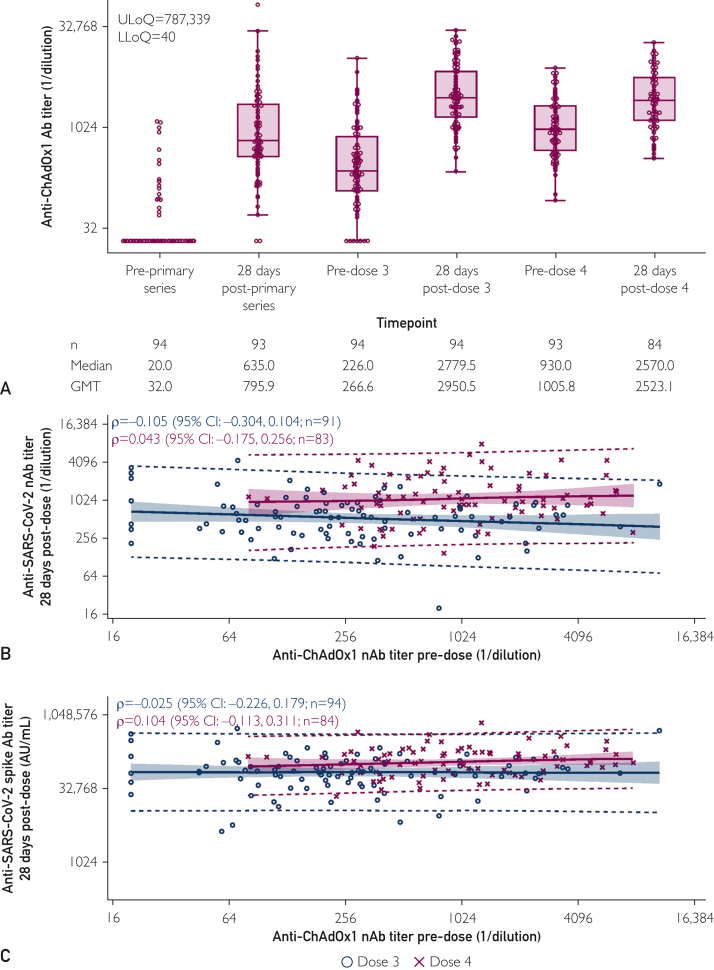
Anti-ChAdOx1 nAb titers over time and the impact on ChAdOx1 nCoV-19-induced humoral immunogenicity against ancestral SARS-CoV-2. (A) Anti-ChAdOx1 nAb titers were assessed before and 28 days postprimary series (dose 2), dose 3, and dose 4 of ChAdOx1 nCoV-19 in the immunogenicity population. Correlations of after third and fourth dose (B) anti-SARS-CoV-2 nAb titers and (C) anti-SARS-CoV-2 spike Ab titers versus the corresponding predose anti-ChAdOx1 nAb titers were assessed in the immunogenicity population. For (A), the bottom and top edges of the box indicate the first and third quartiles (the difference is the IQR), and the line inside the box is the median. Whiskers extend to the minimum and maximum values, excluding outliers. Any points with a log-transformed value of more than 1.5× IQR from the respective edges of the box were considered outliers. Boxplots werecreated based on the log-normal distribution. For (B,C), the dotted lines indicate the 95% prediction limits and the shaded areas the 95% confidence limits. CI, confidence interval; GMT, geometric mean titer; LLoQ, lower limit of quantification; nAb, neutralizing antibody; SARS -CoV, severe acute respiratory syndrome coronavirus; ULoQ, upper limit of quantification.

Anti–ChAdOx1 nAb titers were assessed over time, as antivector responses may inhibit ChAdOx1 nCoV-19–induced immunogenicity. Although anti–ChAdOx1 nAb titers increased following each dose, levels also waned over time (Figure 3A). Anti-ChAdOx1 nAbs GMT postfourth dose (2523.1) was slightly lower numerically than GMT postthird dose (2950.5). Additionally, levels of anti–SARS-CoV-2 nAbs and anti–SARS-CoV-2 spike Abs following third-dose and fourth-dose boosters were not correlated with the corresponding predose levels of anti–ChAdOx1 nAbs (Figure 3B,C); Spearman correlation coefficients were close to 0, with 95% CIs encompassing 0, for all analyses.

Exploratory analysis of antispike Ab responses postthird dose, anti–SARS-CoV-2 immunity through 180 days postfourth dose, and postfourth dose nAb titers against Omicron BA.4/5 in participants with no detectable predosing Omicron BA.4/5 nAbs to can be found in Supplementary Results (Supplemental Appendix; Supplemental Figures 3-6, available online at http://www.mcpiqojournal.org).

### Safety

Median follow-up duration was 107 days for substudy safety population participants (n=172). Local and systemic solicited AEs were reported within 7 days of dosing by 60.3% (n=35/58) and 43.1% (n=25/58) of participants, respectively; most were of mild or moderate severity (Table 1). The most frequent local solicited AEs were pain (58.6% of participants; n=34/58), tenderness (36.2%; n=21/58), and warmth (10.3%; n=6/58); no grade 3 or 4 events were reported (Supplemental Table 3, available online at http://www.mcpiqojournal.org). The most frequent systemic solicited AEs were headache (36.2%; n=21/58), fatigue (22.4%; n=13/58), and malaise (13.8%; n=8/58); 2 participants each reported 2 grade 3 events (muscle pain, headache, fatigue, and malaise), and no grade 4 events were reported.

**Table 1:** Summary of Solicited AEs Within 7 days after the Fourth Dose of ChAdOx1 nCoV-19

Solicited AEs	Fourth dose of ChAdOx1 nCoV-19 (n=58); n (%) of participants
Any solicited AE	38 (65.5)
Any solicited AE ≥ grade 3	2 (3.4)
Any local solicited AE	35 (60.3)
Any local solicited AE ≥ grade 3	0
Any systemic solicited AE	25 (43.1)
Any systemic solicited AE ≥ grade 3	2 (3.4)

Local and systemic AEs known to be associated with vaccination were collected as solicited AEs for 7 days after fourth dose administration in a subset of participants in the safety population. Severity was graded on a 4-point scale: mild (grade 1), moderate (grade 2), severe (grade 3), or potentially life threatening (grade 4). Participants with multiple events in the same category are counted once in that category. Participants with events in >1 category are counted once in each of those categories.

AE, adverse event.

Unsolicited AEs were reported by 12.8% (n=22/172) of participants, all events were of mild/moderate severity, and none were reported in more than 2 participants (Table 2). Supplemental Table 4 (available online at http://www.mcpiqojournal.org) presents a full listing of unsolicited AEs by severity. No SAEs or AEs leading to death were reported. One participant (0.06%) reported an AESI (grade 1 paresthesia of the forearm) that was not considered related to study intervention by the investigator. Unsolicited AEs were considered related to study intervention in 5.8% (n=10/172) of participants; all were mild/moderate severity and none occurred in more than 1 participant (Supplemental Table 5, available online at http://www.mcpiqojournal.org).

**Table 2:** Summary of Unsolicited AEs After the Fourth Dose of ChAdOx1 nCoV-19

Unsolicited AEs	Fourth dose of ChAdOx1 nCoV-19 (n=172)		
	No. (%) of participants	No. of events	Exposure-adjusted rate^a^
Within 28 d after the fourth dose			
Any AE	22 (12.8)	28	1.69
Any related AE	10 (5.8)	11	0.77
Any AE ≥ grade 3	0	0	0
Any related AE ≥ grade 3	0	0	0
AEs with the outcome of death	0	0	0
Within 180 d after the fourth-dose			
Any SAE	0	0	0
Any related SAE	0	0	0
Any SAE ≥ grade 3	0	0	0
Any AESI^b^	1 (0.6)	1	0.02
Any related AESI	0	0	0
Any AESI ≥ grade 3	0	0	0

All participants were evaluated for unsolicited AEs within 28 days of fourth dose administration and unsolicited SAEs and AESIs through to 180 days postdose 4 or the data cutoff, whichever occurred first. AEs were classified as related to study intervention by the investigator. Severity was graded on a 4-point scale: mild (grade 1), moderate (grade 2), severe (grade 3), or potentially life threatening (grade 4). Fatal events were not assigned a severity grading but were reported as SAEs. Prespecified AESI categories included disorders that were neurologic, potentially immune mediated, vascular, or hematologic in nature.^3^ All AEs through 28 days were unsolicited AEs unless categorized as solicited AEs. Participants with multiple events in the same category are counted once in that category. Participants with events in >1 category are counted once in each of those categories.

AE, adverse event; AESI, adverse event of special interest; SAE, serious adverse event.

a Exposure-adjusted rate was calculated as the number of participants with the event divided by the total duration of exposure. Exposure duration was calculated from the date of fourth dose to the relevant time point postdose or the end of study participation, whichever occurred first.

b AESI categories included neurologic, potentially immune mediated, vascular, or hematologic disorders.

## Discussion

These COV003 data, spanning mid-to-late-2022, report that humoral responses following a homologous ChAdOx1 nCoV-19 fourth-dose booster were noninferior to those observed after either a primary series or third-dose booster across all tested SARS-CoV-2 variants; however, the magnitude of postdose elevation, or boost, in humoral responses was smaller following the fourth dose. Additionally, a fourth-dose booster was well tolerated, with lower reactogenicity than previous doses and no SAEs or emergent safety concerns detected.^3^^,^^14^^,^^15^^,^^18^^,^^19^ It should be noted that fourth-dose boosters were prioritized in Brazil for older individuals following Omicron emergence, whereas these data are from a generally young population (median age, 36.8 years).^16^^,^^20^^,^^21^

At the time of this study, a high degree of hybrid immunity (from natural infection and COVID-19 vaccination) was observed in Brazil.^22^ Indeed, despite the exclusion of participants with confirmed SARS-CoV-2 infection, before fourth dose administration approximately one-third of participants were SARS-CoV-2 seropositive and higher than expected anti–SARS-CoV-2 immunogenicity was observed. Underreporting of infections could have contributed to these findings; however, given the generally lower severity of Omicron in younger individuals,^23^ it is likely that the elevated SARS-CoV-2 immunity was driven primarily by undetected infections in the study population.

It is also likely that the elevated immunity observed contributed to the comparatively lower boost in response observed after the fourth dose. A Brazilian real-world study during Omicron predominance found that fourth-dose boosters of monovalent COVID-19 vaccines, including ChAdOx1 nCoV-19, provided a smaller relative increase in protection against COVID-19 hospitalization versus third-dose boosters.^16^ Additionally, data from 2 studies suggest that participants with lower predosing Ab levels derived more benefit from a fourth-dose booster than those without. These results highlight the importance of optimizing COVID-19 booster timing and are supportive of the theory that boosters confer the greatest benefit to population-level immunity during periods of reduced viral transmission.^24^

Antivector immunity to adenoviral-vectored vaccines, including ChAdOx1 nCoV-19, has been hypothesized to hamper the humoral responses induced by subsequent doses, negatively impacting vaccine efficacy.^25^ Although anti–ChAdOx1 nAb titers did increase after each dose, levels waned during each vaccination interval (median, 11.1 months for the third dose and 10.7 months for the fourth dose). In fact, postfourth dose anti–ChAdOx1 nAb levels were numerically lower than postthird dose levels. Furthermore, postdosing anti–SARS-CoV-2 responses were not correlated with anti–ChAdOx1 nAbs levels for either booster. These results are consistent with previous analyses suggesting that anti–ChAdOx1 nCoV-19 nAbs do not impede vaccine-induced anti–SARS-CoV-2 responses.^2^^,^^26^ These data suggest that, provided that there is a sufficient dosing interval, repeated doses of ChadOx1 nCoV-19 remain immunogenic.

Reactogenicity following a fourth dose of ChAdOx1 nCoV-19 was similar to or lower than that observed for previous doses. Younger age is a described risk factor for reactogenicity following COVID-19 vaccines; however, despite this study’s generally young population, a fourth dose of ChAdOx1 nCoV-19 remained well tolerated.^18^^,^^27^ The safety profile of ChAdOx1 nCoV-19 as a fourth-dose booster is consistent with third dose and primary series vaccination in previous clinical studies.^3^^,^^14^^,^^15^^,^^18^^,^^19^ Extremely rare events of thrombotic thrombocytopenia syndrome have been detected through postmarketing data, mainly in Europe; however, no cases were reported in this COV003 substudy, nor any other clinical study of ChAdOx1 nCoV-19.^3^^,^^14^^,^^15^^,^^18^^,^^19^ Therefore, ongoing pharmacovigilance remains the most appropriate method for assessing the etiology and dynamics of thrombotic thrombocytopenia syndrome.

The lack of controls, resulting from the need to immunize participants according to Government recommendations, was a key limitation of these analses.^16^ Several phase 3 COVID-19 vaccine trails were also affected thusly, highlighting challenges in conducting randomized, placebo-controlled studies while prioritizing pandemic responses.^28^ Pandemic dynamics also affected enrolment. Participants with previous SARS-CoV-2 infection were generally ineligible, particularly reducing eligible participants following Omicron.^21^ Additionally, older (≥65 years of age) participants were reduced as, in Brazil, nonstudy COVID-19 booster vaccinations were already available. Consequently, the number of substudy participants (n=172) was lower than the 350 planned.^16^^,^^29^ However, the ChAdOx1 nCoV-19 immunogenicity findings in this study are unlikely to have been affected because no difference has been observed in older versus younger adults.^18^^,^^19^ Finally, an exhaustive comparison of postdosing safety events was not possible owing to the small sample size and lack of controls.

## Conclusion

We find that a ChAdOx1 nCoV-19 fourth dose induced noninferior humoral responses in adults compared with both third dose and primary series. Additionally, reactogenicity was low, and the safety profile was consistent with third dose and primary series vaccination. However, immunogenicity analyses were complicated by the detection of hybrid immunity in approximately one-third of substudy participants predosing, likely owing to Omicron infections.

## Potential Competing Interests

Sara Kelly is a contributor to intellectual property licensed by Oxford University Innovation to AstraZeneca. Merryn Voysey reports receiving institutional grants from AstraZeneca to University of Oxford. Dr Lambe reports consulting fees from Vaccitech for an unrelated project, an honorarium from Seqirus for an unrelated influenza meeting, work-related investments, and grants from Vaccine Taskforce via NIHR and AstraZeneca and is named as an inventor on a patent application for a vaccine against SARS-CoV-2. Dr Kelly, Dr Green, Dr Shoemaker, Maria Bergagård, Dr Shankar, Dr Morgan, and Dr Villafana are current or former employees of AstraZeneca and may hold AstraZeneca stock or stock options. Dr Isaacs, Dr Lan, and Dr Bansal were contracted to AstraZeneca at the time of the study. Dr Isaacs is an employee of Phastar (Durham, NC); Dr Lan is an employee of Cytel, Inc (Cambridge, MA); Dr Bansal is an employee of Bogier Consulting (Raleigh, NC). Dr Kelly is a former employee of AstraZeneca and now an employee of Sanofi (Swiftwater, PA, USA) and has held AstraZeneca stocks. Dr Pollard was a member of WHO’s Strategic Advisory Group of Experts on Immunization until January 2022, remains Chair of WHO’s salmonella Technical Advisory Group, and Chair of the UK Department of Health and Social Care’s Joint Committee on Vaccination and Immunisation (JCVI) but did not participate in the JCVI COVID-19 committee during the pandemic and reports providing advice to Shionogi on COVID-19, receiving mRNA from Moderna, and funding from the National Institute for Health and Care Research (NIHR), AstraZeneca, the Bill & Melinda Gates Foundation, Wellcome, the Medical Research Council, the Coalition for Epidemic Preparedness Innovations, the Serum Institute of India, and the European Commission. The University of Oxford entered into a partnership with AstraZeneca for the development of COVID-19 vaccines. Grants to support the conduct of this study were provided by AstraZeneca and the UK Vaccine Taskforce via NIHR. The other authors report no competing interests.

## Ethics Statement

The initial COV003 study protocol and subsequent protocol amendments were approved by the Oxford Tropical Research Ethics Committee (OxTREC; most recent approval September 13, 2022) in the United Kingdom and by the Comissão Nacional de Ética em Pesquisa (CONEP; most recent approval November 9, 2022) in Brazil.^2^^,^^3^ This study was performed in accordance with ethical principles originating in the Declaration of Helsinki and consistent with International Council for Harmonisation Good Clinical Practice, applicable regulatory requirements, and AstraZeneca’s policy on Bioethics. All participants provided written informed consent.
