# Supplementary material for: Immunogenicity and Safety of ChAdOx1 nCoV-19 (AZD1222) as a Homologous Fourth-Dose Booster: A Substudy of the Phase 3 COV003 Trial in Brazil
Source: Mayo Clin Proc Innov Qual Outcomes. 2025 Jul 11;9(4):100642. doi: 10.1016/j.mayocpiqo.2025.100642 (PMC12275839; doi:10.1016/j.mayocpiqo.2025.100642)
Supplement: Supplementary Materials [file mmc1.pdf]

Supplementary materials

Immunogenicity and safety of ChAdOx1 nCoV-19 (AZD1222) as a homologous fourth-dose booster: a substudy of the Phase 3 COV003 trial in Brazil

Sue Ann Costa Clemens, MD PhD,* Sagida Bibi, PhD, Natalie G. Marchevsky, MSc, Parvinder K. Aley, PhD, Federica Cappuccini, PhD, Sophie A. Davies, BSc, Isabela Gonzalez, PhD, Sarah C. Kelly, MSc, Yama F. Mujadidi, MSc, Eveline Pipolo Milan, MD PhD Alexandre V. Schwarzbold, PhD, Eduardo Sprinz, MD DSc, Merryn Voysey, DPhil, Prof Lily Y. Weckx, MD PhD, Daniel Wright, DPhil, Himanshu Bansal, MS, Maria A. S. Bergagård, MSc, Abby J. Isaacs, MS, Elizabeth J. Kelly, PhD, Dongmei Lan, MS, Shethah Morgan, PhD, Nirmal Kumar Shankar, BAMS, Kathryn Shoemaker, MS, Tonya L. Villafana, PhD, Teresa Lambe, PhD, Justin A. Green, MD PhD, Prof Andrew J. Pollard, FMedScia, and the COV003 Study Group

**Contents**	Page
**Supplementary Acknowledgments**	1
COV003 Clinical Study Group members	1
**Supplementary Methods**	2
Exploratory subgroup participants	2
Exploratory third-dose subgroup	2
Exploratory fourth-dose subgroup	2
Serology assessments	2
Multiplex immunoglobulin G (IgG) immunoassay	2
Pseudovirus neutralization assay	2
ChAdOx1 neutralization assay	3
Standardized enzyme-linked immunosorbent assay (ELISA)	3
Local and systemic solicited adverse events (AEs)	3
Severity grading for adverse events	3
Exploratory immunogenicity outcomes	3
Exploratory substudy immunogenicity	3
Exploratory subgroup immunogenicity	4
**Supplementary Results**	5
Post-third dose anti-spike humoral responses	5
Durability of post-fourth dose anti-SARS-CoV-2 immunity	5
Post-fourth dose nAb titers against Omicron BA.4/5 in infection naïve participants	5
**Supplementary Figures**	6
Fig. S1. Anti-spike Ab titers against (A) ancestral SARS-CoV-2 and (B) Omicron BA.1 pre- and 28 days post-primary-series, third dose, and fourth dose of ChAdOx1 nCoV-19.	6
Fig. S2. Anti-spike Ab titers against multiple SARS-CoV-2 variants pre- and 28 days post-primary-series, third dose, and fourth dose of ChAdOx1 nCoV-19.	8
Fig. S3. Anti-spike Ab titers against ancestral SARS-CoV-2 28 days post-primary-series and third dose of ChAdOx1 nCoV-19 via ELISA, in participants who received a third dose only.	11
Fig. S4. Anti-spike Ab titers against ancestral SARS-CoV-2 up to 180 days post-fourth dose of ChAdOx1 nCoV-19, summarized by evidence of SARS-CoV-2 infection after the fourth dose.	12
Fig. S5. nAb titers against Omicron BA.4/5 pre- and 28 days post-primary-series, third dose, and fourth dose of ChAdOx1 nCoV-19, in participants with no measurable nAb response to Omicron BA.4/5 prior to the fourth dose.	13
Fig. S6. Anti-spike Ab titers against ancestral SARS-CoV-2 at pre- and 28-days post-fourth dose via ELISA.	14
**Supplementary Tables**	15
Table S1. Participant demographics and clinical characteristics in key analysis populations collected at baseline (prior to receiving the primary series, unless indicated otherwise).	15
Table S2. Immunogenicity outcomes comparisons of nAb and anti-spike Ab titers against multiple SARS-CoV-2 variants.	17
Table S3. Solicited AEs within 7 days after the fourth dose of ChAdOx1 nCoV-19 by severity.	20
Table S4. Unsolicited AEs within 28 days after the fourth dose of ChAdOx1 nCoV-19 by severity.	23
Table S5. Unsolicited AEs related to study intervention within 28 days after the fourth dose of ChAdOx1 nCoV-19.	26
**Supplementary References**	27
**COV003 Protocol v13.0**	28
**COV003 Protocol v14.0**	138

Supplementary Acknowledgments

COV003 Clinical Study Group members

The following table shows the COV003 Clinical Study Group members who contributed to exploratory immunogenicity analyses and are included in the list of collaborators for this manuscript.

Collaborators	Affiliation
Ahmed Eldawi	Oxford Vaccine Group, Department of Paediatrics, University of Oxford
Emma Sheehan	Oxford Vaccine Group, Department of Paediatrics, University of Oxford
Suzanne Woodlock	Oxford Vaccine Group, Department of Paediatrics, University of Oxford

Supplementary Methods

1. Exploratory subgroup participants

1.1. Exploratory third-dose subgroup

Under protocol v13.0 and v14.0, COV003 participants were not eligible to receive a third dose if they had self-reported prior severe acute respiratory syndrome coronavirus 2 (SARS-CoV-2) infection (confirmed via reverse transcription-polymerase chain reaction [RT-PCR] test) or had received a non-study COVID-19 vaccine. Participants who received a third dose of ChAdOx1 nCoV-19 within COV003 but did not receive a subsequent fourth dose, and who had Ab measurements at 28 days post-dose 3, were included in the exploratory third-dose subgroup. Participants with SARS-CoV-2 infection (confirmed via RT-PCR test) between third dose administration and 28 days post-third dose were excluded.

1.2. Exploratory fourth-dose subgroup

Under protocol v14.0, the criteria for eligibility to receive a fourth-dose booster of ChAdOx1 nCoV-19 within COV003 were updated. Participants with self-reported SARS-CoV-2 infection (confirmed via RT-PCR or rapid lateral flow test) within 4 weeks of enrollment to receive a fourth dose were excluded, thus allowing participants with SARS-CoV-2 infection prior to 4 weeks before fourth dose enrollment to receive a fourth-dose booster. Participants who received a fourth dose under this later protocol amendment, and who had Ab measurements at pre-dose 4, were included in the exploratory fourth-dose subgroup. Participants with SARS-CoV-2 infection (reported via 'COVID Diagnosis' case report form) between fourth dose administration and 28 days post-fourth dose were excluded.

2. Serological assessments

2.1. Multiplex immunoglobulin G (IgG) immunoassay

Titers of anti- SARS-CoV-2 nucleocapsid antibodies (Abs) and anti-SARS-CoV-2 spike Abs against multiple SARS-CoV-2 variants (ancestral, Alpha, Beta, Gamma, Delta and Omicron BA.1) were quantified in participant serum samples using a multiplexed immunoassay, unless otherwise specified.[1,2] Briefly, the Meso Scale Discovery® platform was used to determine Ab concentrations through indirect binding. Relative light unit outputs were then interpolated relative to a standard curve generated from a serially diluted reference standard (pooled coronavirus disease 2019 [COVID-19]-positive serum samples) and assigned a concentration of arbitrary units (AU)/mL. An assay cut point for nucleocapsid seropositivity was defined as ≥19,904 AU/mL; this threshold corresponds to a previously determined 99th percentile cut point for nucleocapsid antigens of 9787 AU/mL, multiplied by a factor of 2.03375 to account for the different assay being used.[1] All analyses were performed at PPD Vaccines (Richmond, VA, USA) in validated assays.

2.2. Pseudovirus neutralization assay

Pseudovirus neutralization assays were used to determine anti-SARS-CoV-2 nAb titers in participant serum samples.[2] Briefly, pseudovirions containing luciferase and a variant-dependent SARS-CoV-2 spike protein were preincubated with serial dilutions of serum and subsequently inoculated onto target cells. Anti-SARS-CoV-2 nAb titers were reported as the reciprocal of the serum dilution equivalent to the ID_{50} of pseudovirus infection (a fifty percent reduction of luciferase reporter signal). All analyses were performed at Monogram Biosciences (South San Francisco, CA) in validated assays.

2.3. ChAdOx1 neutralization assay

Anti-ChAdOx1 nAb titers were quantified in participant serum using a ChAdOx1 neutralization assay. Briefly, recombinant ChAdOx1 vectors containing luciferase and the chimpanzee adenoviral capsid were preincubated with serial dilutions of serum and subsequently transduced into target cells. Anti-ChAdOx1 nAb titers were reported as the reciprocal of the serum dilution equivalent to the ID_{50} of recombinant ChAdOx1 vector transduction (a fifty percent reduction of luciferase reporter signal). All analyses were performed at Monogram Biosciences (South San Francisco, CA) in validated assays.

2.4. Standardized enzyme-linked immunosorbent assay (ELISA)

Anti-SARS-CoV-2 spike Ab titers were determined using an in-house indirect ELISA relative to a standard curve derived from a pool of SARS-CoV-2 convalescent plasma samples on each plate, as described previously.[3,4] ELISA plates were coated with full-length trimerized ancestral SARS-CoV-2 spike glycoprotein. Serum samples diluted in casein were plated and incubated alongside two internal positive controls (controls 1 and 2). Control 1 was a dilution of convalescent plasma sample and control 2 was a research reagent for anti-SARS-CoV-2 Ab (code 20/130; National Institute for Biological Standards and Control). The standard pool was serially diluted to produce ten standard points that were assigned arbitrary ELISA units (EUs). Goat anti-human IgG (γ-chain specific) conjugated to alkaline phosphatase was used as secondary Ab and plates were developed by adding 4-nitrophenyl phosphate in diethanolamine substrate buffer. An ELx808 microplate reader (BioTek Instruments) was used to provide optical density measurement of the plates at 405 mm. Standardised EUs were determined from a single dilution of each sample against the standard curve which was plotted using the 4-parameter logistic model (Gen5 v3.09, BioTek). Each assay plate consisted of samples and controls plated in triplicate, with ten standard points in duplicate and four blank wells. All analyses were performed at The University of Oxford.

3. Local and systemic solicited adverse events (AEs)

Local solicited AEs included: pain, tenderness, redness, warmth, itching, swelling, and induration. Systemic solicited AEs included: fever, feverishness, chills, joint pain, muscle pain, fatigue, headache, malaise, nausea, and vomiting.

4. Severity grading for adverse events

The severity of AEs was graded on a 4-point scale: mild (Grade 1), moderate (Grade 2), severe (Grade 3), or potentially life-threatening (Grade 4). Fatal events were not assigned a severity grading but were always reported as serious AEs.

5. Exploratory immunogenicity outcomes

5.1. Exploratory substudy immunogenicity

To assess the durability of humoral immune responses following the fourth dose of ChAdOx1 nCoV-19, anti-spike Ab titers were examined through 180 days after the fourth dose in the extended immunogenicity population (data cutoff [DCO]: September 11, 2023). To evaluate the impact of post-fourth dose SARS-CoV-2 infection on extended immunogenicity, anti-spike Ab titers were summarized by evidence of SARS-CoV-2 infection at 180 days post-fourth dose administration, defined as an anti-SARS-CoV-2 nucleocapsid fold-rise of ≥2 from 28 days post-dose 4 to 180 days post-dose 4.

To investigate the impact of high-levels of pre-existing anti-SARS-CoV-2 immunity on the nAb response following the fourth dose of ChAdOx1 nCoV-19, a post hoc analysis was conducted to assess nAb titers against Omicron BA.4/5 at pre- and 28 days post- primary series, third dose, and fourth dose of ChAdOx1 nCoV-19 in a subset of participants in the immunogenicity population with no measurable nAb response against Omicron BA.4/5 at fourth dose administration.

To explore the effect of repeated ChAdOx1 nCoV-19 dosing on the accumulation of anti-ChAdOx1 nAbs, anti-ChAdOx1 nAb titers were assessed pre- and 28 days post- primary series, third dose, and fourth dose of ChAdOx1 nCoV-19 in the immunogenicity population. The relationship between anti-ChAdOx1 nAb levels before the third and fourth dose, and anti-SARS-CoV-2 spike Abs and nAbs at 28 days after each corresponding dose, was determined to assess the impact of anti-vector nAbs on vaccine-induced humoral immunity.

5.2. Exploratory subgroup immunogenicity

To evaluate the immunogenicity of ChAdOx1 nCoV-19 as a third-dose booster compared with primary-series vaccination, anti-spike Ab titers against ancestral SARS-CoV-2 were assessed at 28 days post- primary series and third dose in subgroup participants who received a third-dose booster only (DCO: November 22, 2023), using the ELISA described above.

To investigate the effect of pre-existing anti-SARS-CoV-2 immunity on the immune response induced by a fourth dose of ChAdOx1 nCoV-19, anti-spike Ab titers against ancestral SARS-CoV-2 were assessed in paired samples collected at pre- and 28-days post-fourth dose in participants who received a fourth-dose booster under a protocol amendment that allowed for the inclusion of individuals with prior SARS-CoV-2 infection (DCO: September 11, 2023), using the ELISA described above.

Supplementary Results

Post-third dose anti-spike humoral responses

An exploratory analysis compared anti-spike Ab responses at 28 days post-third dose and 28 days post-primary-series in the exploratory third-dose subgroup, who received a third dose ChAdOx1 nCoV-19 only (n = 1327); geometric mean fold rise (GMFR) was 5.0 (95% [confidence interval] CI: 4.6–5.3) in this larger cohort (see 'Exploratory subgroup immunogenicity', appendix p.4; **Fig. S3**).

Durability of post-fourth dose anti-SARS-CoV-2 immunity

Anti-SARS-CoV-2 immunity was durable through 180 days after the fourth dose in the extended immunogenicity population (**Fig. S4**). Anti-spike Ab geometric mean titers (GMTs) against ancestral SARS-CoV-2 decreased only slightly from 101,329 AU/mL (95% CI: 89,224–115,077; n = 130) at 28 days post-dose 4 to 95,009 AU/mL (95% CI: 82,563–109,330; n = 116) at 180 days post-dose 4, with a GMFR of 0.9 (95%CI: 0.8–1.1; n = 112). Participants with evidence of SARS-CoV-2 infection at 180 days post-dose 4 (defined as an anti-SARS-CoV-2 nucleocapsid fold-rise of ≥2 from 28 days post-dose 4 to 180 days post-dose 4; see 'Exploratory subgroup immunogenicity', appendix p.4) had higher Ab levels at 180 days after the fourth dose than those with no evidence of infection (GMT: 137,123 AU/mL [95% CI: 111,920–168,001; n = 46] vs 72,756 AU/mL [95% CI: 61,487–86,089; n = 65).

Post-fourth dose nAb titers against Omicron BA.4/5 in infection naïve participants

To test the hypothesis that the smaller increase in humoral response after the fourth dose versus the third dose was a result of pre-existing anti-SARS-CoV-2 immunity, an exploratory post hoc analysis was performed to assess nAb titers against Omicron BA.4/5 in a small subset of participants in the immunogenicity population with no measurable nAb response to Omicron BA.4/5 immediately before fourth dose administration (n = 13). In this subset, median nAb responses were numerically higher after a fourth dose of ChAdOx1 nCoV-19 (GMT: 389.9 [95% CI: 281.8–539.3]) relative to pre-dose levels (173.8 [95% CI: 80.2–376.7]; **Fig. S5**). A further analysis of anti-spike Ab titers pre-dose and 28 days post-dose 4 in the exploratory fourth-dose subgroup, which allowed the inclusion of participants with SARS-CoV-2 infection prior to 4 weeks before fourth dose enrolment, revealed mixed results (see 'Exploratory subgroup participants', appendix p.2; **Fig. S6**). Overall, GMFR was 0.7 (95% CI: 0.6–1.0; n = 43); however, observation of paired samples revealed a possible trend for increased Ab responses post-dose 4 in participants with lower pre-dose 4 Ab titers.

Supplementary Figures

Fig. S1. Anti-spike Ab titers against (A) ancestral SARS-CoV-2 and (B) Omicron BA.1 pre- and 28 days post-primary-series, third dose, and fourth dose of ChAdOx1 nCoV-19.

Anti-spike Ab titers were assessed pre- and 28 days post-primary series (dose 2), dose 3, and dose 4 of ChAdOx1 nCoV-19 in the immunogenicity population. The bottom and top edges of the box indicate the first and third quartiles (the difference is the IQR), and the line inside the box is the median. Whiskers extend to the minimum and maximum values, excluding outliers. Any points with a log-transformed value of more than 1.5 x IQR from the respective edges of the box were considered outliers. Boxplots were created based on the log-normal distribution. GMFRs were calculated in participants with Ab measurements at both compared timepoints; for a full listing of GMTs associated with the provided GMFRs see Table S1.

Ab, antibody; AU, arbitrary units; CI, confidence interval; GMFR, geometric mean fold rise; GMT, geometric mean titer; IQR, interquartile range; LLoQ, lower limit of quantification; SARS-CoV-2, severe acute respiratory syndrome coronavirus 2; ULoQ, upper limit of quantification.

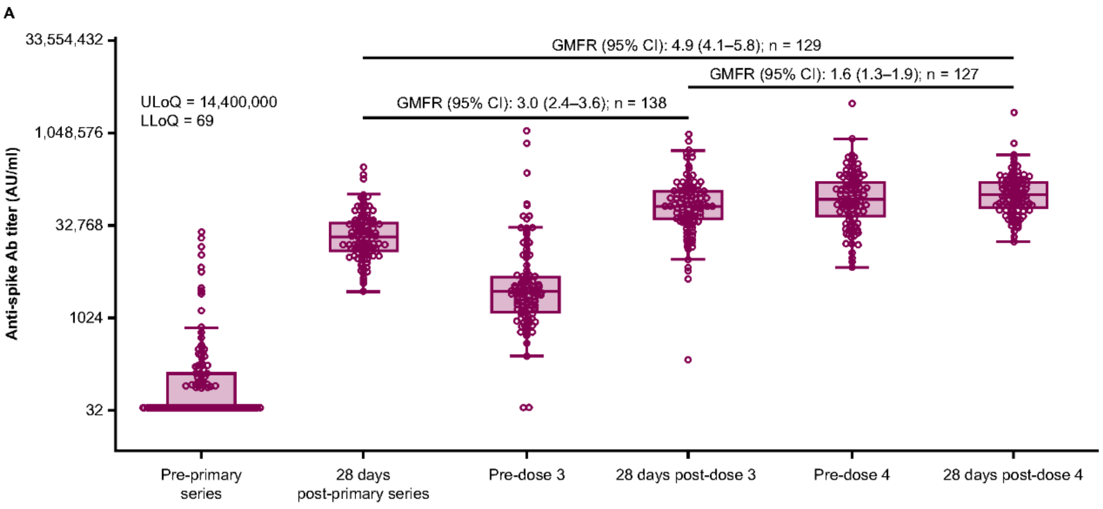

	Pre-primary series	28 days post-primary series	Pre-dose 3	28 days post-dose 3	Pre-dose 4	28 days post-dose 4
n	142	141	142	139	141	130
Median	34.5	21,205.0	2589.5	67,153.0	85,228.0	100,815.0
GMT	83.9	21,524.6	3048.1	63,704.8	86,343.4	101,329.4

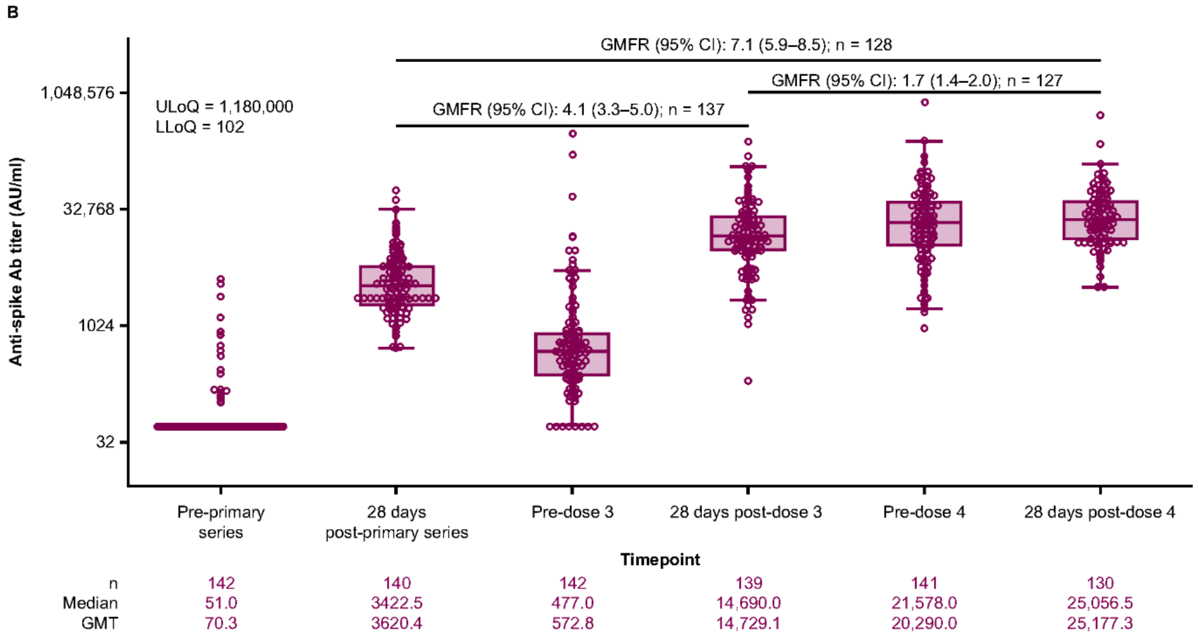
B
GMFR (95% CI): 7.1 (5.9–8.5); n = 128
GMFR (95% CI): 1.7 (1.4–2.0); n = 127
GMFR (95% CI): 4.1 (3.3–5.0); n = 137
ULoQ = 1,180,000
LLoQ = 102
Anti-spike Ab titer (AU/ml)
1,048,576
32,768
1024
32
Pre-primary series
28 days post-primary series
Pre-dose 3
28 days post-dose 3
Pre-dose 4
28 days post-dose 4
Timepoint
n 142 140 142 139 141 130
Median 51.0 3422.5 477.0 14,690.0 21,578.0 25,056.5
GMT 70.3 3620.4 572.8 14,729.1 20,290.0 25,177.3

Fig. S2. Anti-spike Ab titers against multiple SARS-CoV-2 variants pre- and 28 days post-primary-series, third dose, and fourth dose of ChAdOx1 nCoV-19.

Anti-spike Ab titers against **(A)** Alpha, **(B)** Beta, **(C)** Gamma and **(D)** Delta variants were assessed pre- and 28 days post-primary series, dose 3, and dose 4 of ChAdOx1 nCoV-19 in the immunogenicity population. The bottom and top edges of the box indicate the first and third quartiles (the difference is the IQR), and the line inside the box is the median. Whiskers extend to the minimum and maximum values, excluding outliers. Any points with a log-transformed value of more than 1.5 x IQR from the respective edges of the box were considered outliers. Boxplots were created based on the log-normal distribution. GMFRs were calculated in participants with Ab measurements at both compared timepoints; for a full listing of GMTs associated with the provided GMFRs see Table S1.

Ab, antibody; AU, arbitrary units; CI, confidence interval; GMFR, geometric mean fold rise; GMT, geometric mean titer; IQR, interquartile range; LLoQ, lower limit of quantification; SARS-CoV-2, severe acute respiratory syndrome coronavirus 2; ULoQ, upper limit of quantification.

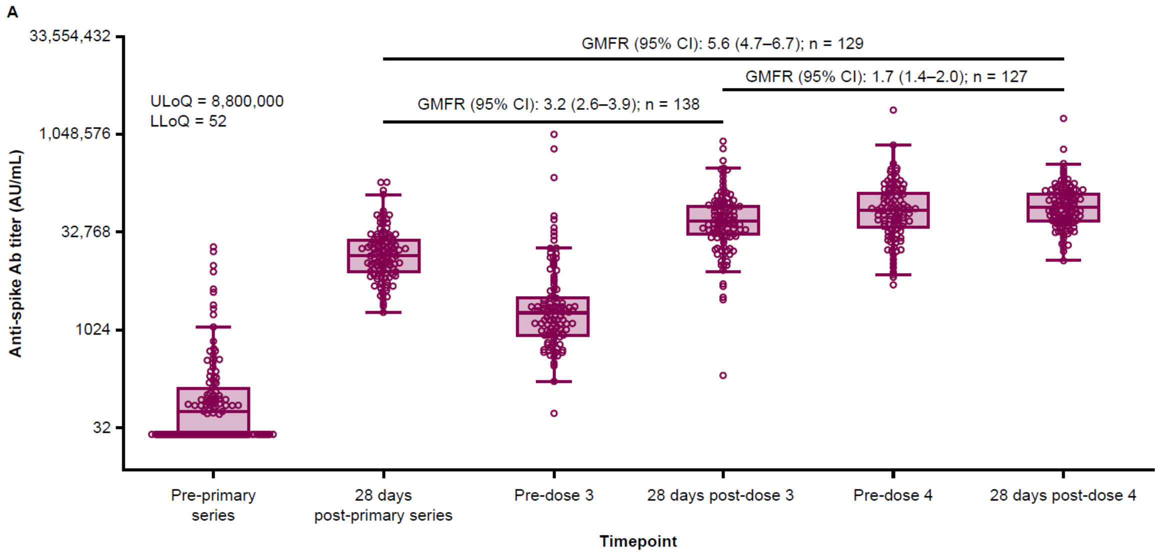

	Pre-primary series	28 days post-primary series	Pre-dose 3	28 days post-dose 3	Pre-dose 4	28 days post-dose 4
n	142	141	142	139	141	130
Median	59.0	14,263.0	1931.5	48,322.0	71,312.0	77,046.0
GMT	78.9	14,611.2	2223.6	47,066.4	67,189.2	79,257.4

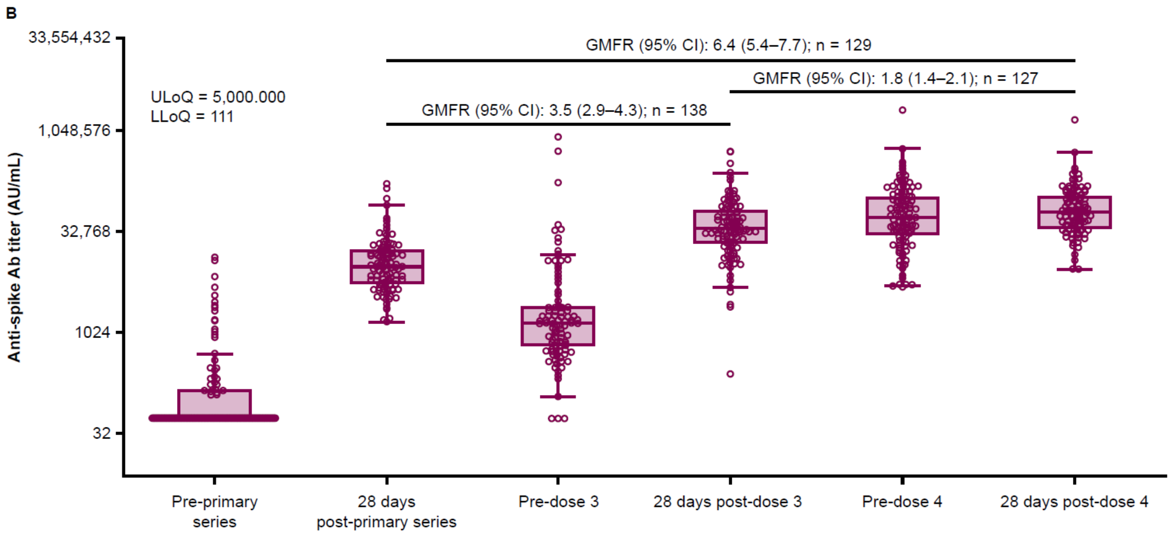

	Pre-primary series	28 days post-primary series	Pre-dose 3	28 days post-dose 3	Pre-dose 4	28 days post-dose 4
n	142	141	142	139	141	130
Median	55.0	9766.0	1424.0	36,127.0	53,398.0	63,237.5
GMT	107.1	10,188.3	1690.9	36,071.9	52,510.1	63,989.1

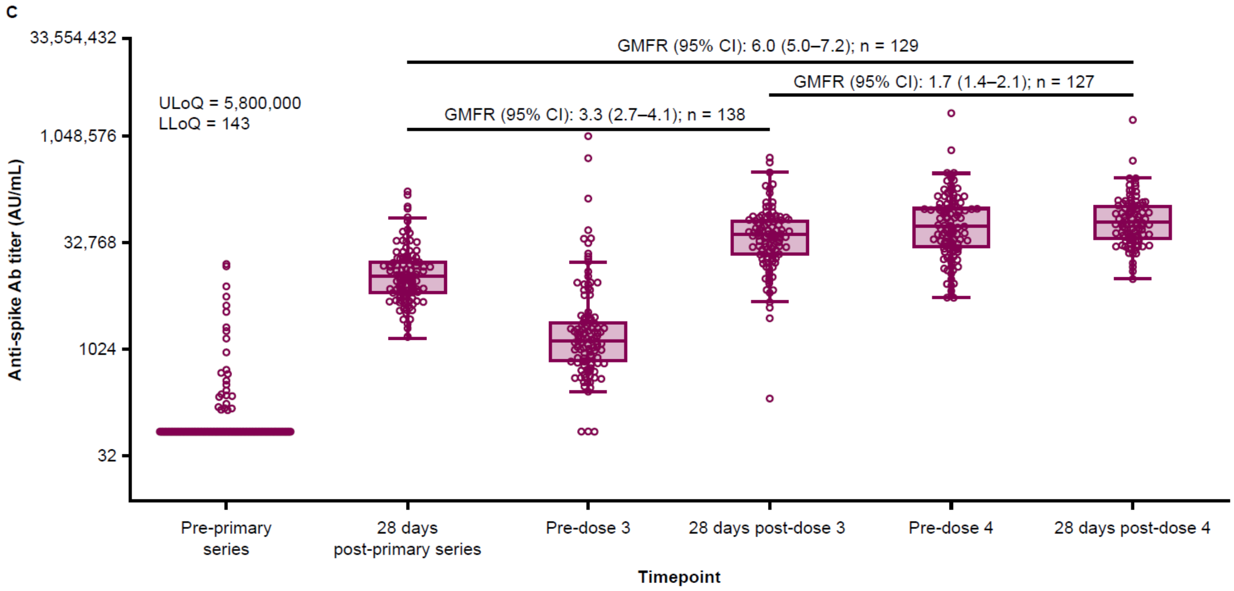

	Pre-primary series	28 days post-primary series	Pre-dose 3	28 days post-dose 3	Pre-dose 4	28 days post-dose 4
n	142	141	142	139	141	130
Median	71.5	11,959.0	1400.5	45,970.0	61,185.0	67,154.5
GMT	117.1	11,975.8	1853.5	40,072.8	59,293.1	69,755.4

D

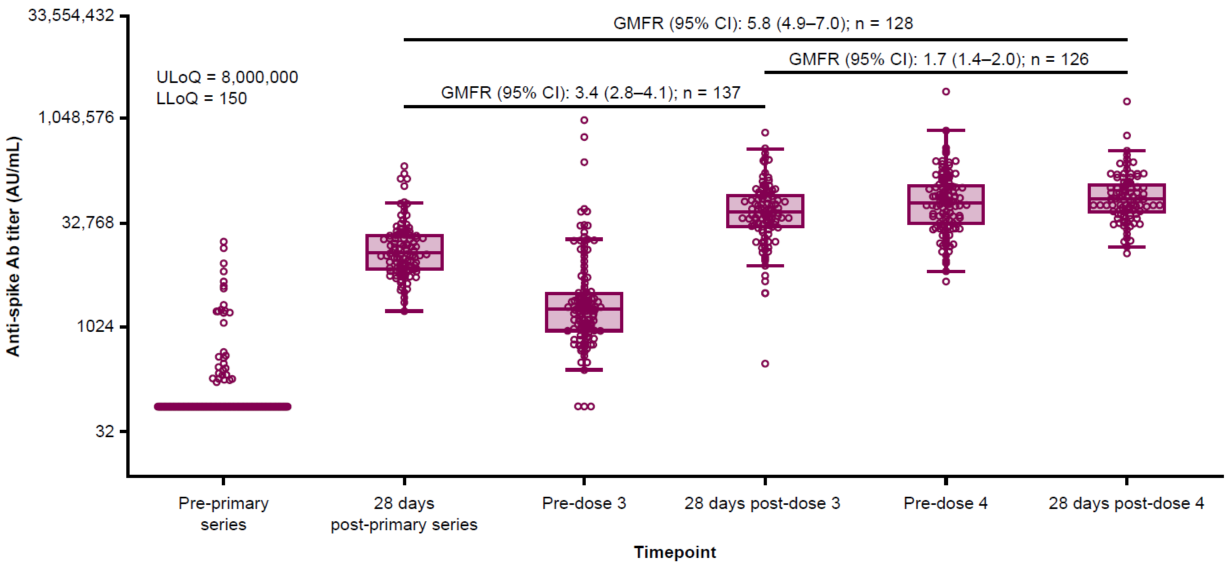

	Pre-primary series	28 days post-primary series	Pre-dose 3	28 days post-dose 3	Pre-dose 4	28 days post-dose 4
n	141	141	142	139	141	130
Median	75.0	12,629.0	1854.5	45,311.0	64,234.0	72,307.5
GMT	130.1	13,252.1	2211.2	44,779.4	62,292.8	74,391.3

Fig. S3. Anti-spike Ab titers against ancestral SARS-CoV-2 28 days post-primary-series and third dose of ChAdOx1 nCoV-19 via ELISA, in participants who received a third dose only.

Anti-spike Ab titers were assessed at 28 days post-primary-series (dose 2; n = 1327) and dose 3 (n = 1850) of ChAdOx1 nCoV-19 in the exploratory third-dose subgroup. The bottom and top edges of the box indicate the first and third quartiles (the difference is the IQR), and the line inside the box is the median. Whiskers extend to 1.5 x IQR from the respective edges of the box.

Data cutoff: November 22, 2023.

Ab, antibody; CI, Confidence interval; ELISA, enzyme-linked immunosorbent assay; GMFR, geometric mean fold rise; GMT, geometric mean titer; IQR, interquartile range; SARS-CoV-2, severe acute respiratory syndrome coronavirus 2.

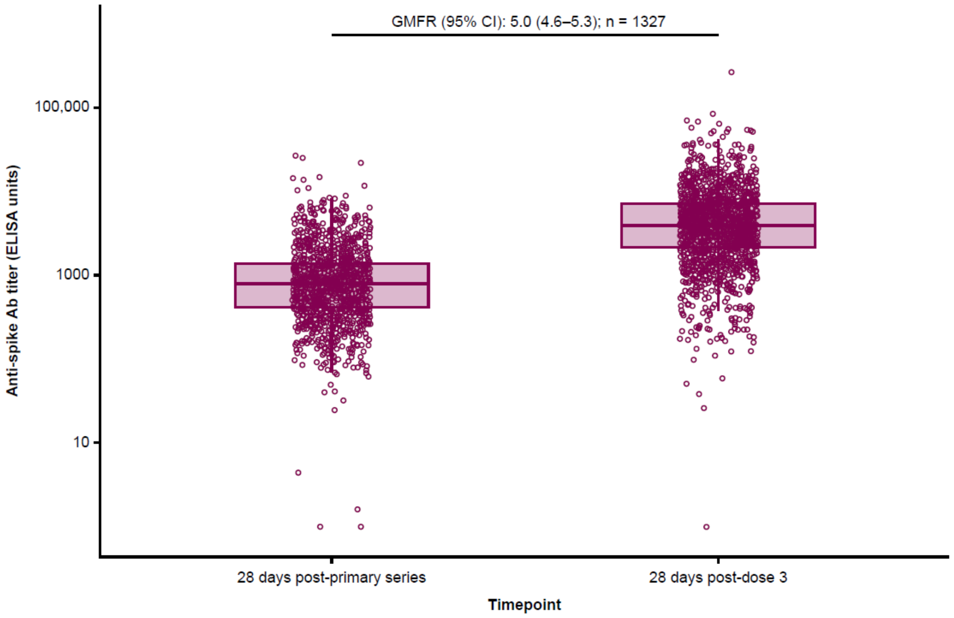

	28 days post-primary series	28 days post-dose 3
n	1327	1850
Median	791	3954
GMT	768	3738

Fig. S4. Anti-spike Ab titers against ancestral SARS-CoV-2 up to 180 days post-fourth dose of ChAdOx1 nCoV-19, summarized by evidence of SARS-CoV-2 infection after the fourth dose.

Anti-spike Ab titers were assessed pre- (n = 141), 28 days post- (n = 130), and 180 days post-dose 4 (n = 116) of ChAdOx1 nCoV-19 in the extended immunogenicity population. Data are summarized by evidence of SARS-CoV-2 infection at 180 days post-fourth dose administration, defined as an anti-SARS-CoV-2 nucleocapsid fold-rise of ≥2 from 28 days post-dose 4 to 180 days post-dose 4. Solid data points and error bars indicate the GMTs and 95% CIs, respectively. Lines connect the GMTs at each timepoint within each population. GMFRs presented are from the 28 days post-dose 4 to 180 days post-dose 4 timepoints.

Data cutoff: September 11, 2023.

Ab, antibody; AU, arbitrary units; CI, confidence interval; GMFR, geometric mean fold-rise; GMT, geometric mean titer; SARS-CoV-2, severe acute respiratory syndrome coronavirus 2.

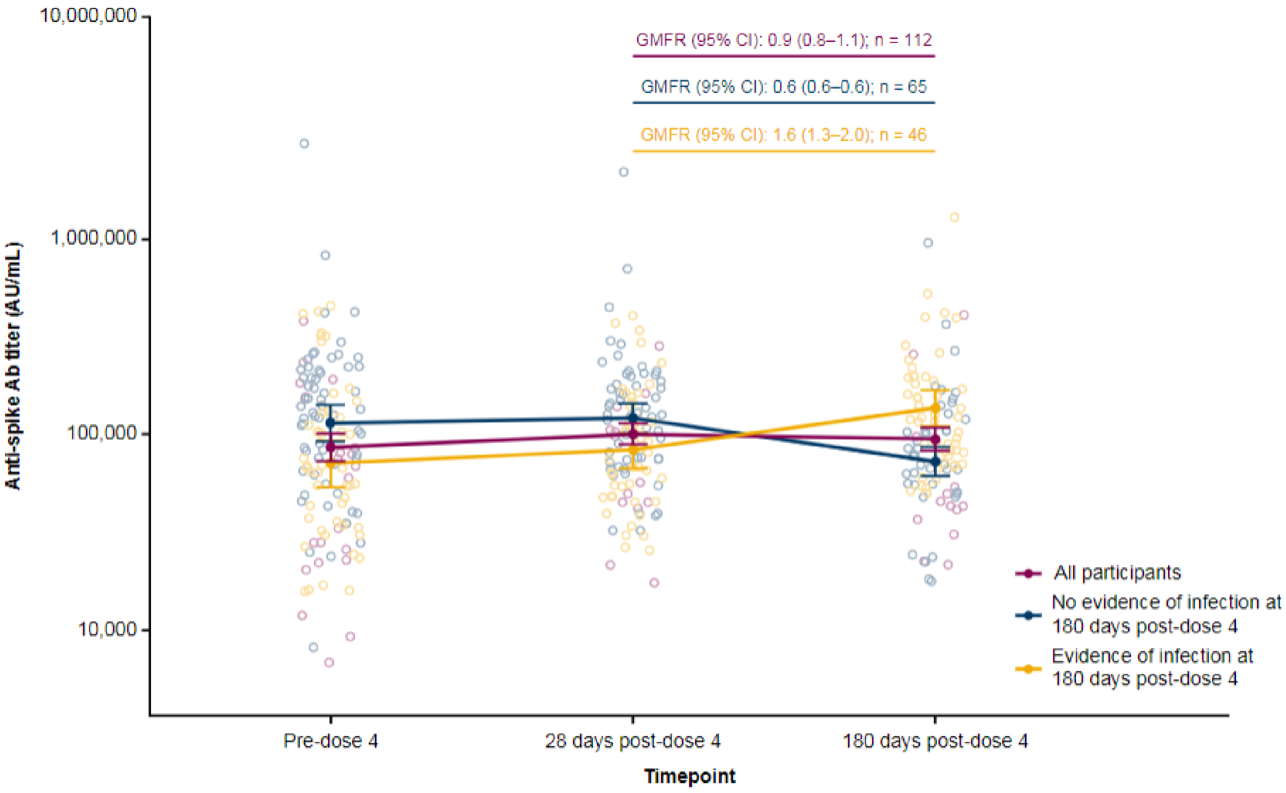

	Pre-dose 4	28 days post-dose 4	180 days post-dose 4
n	141	130	116
Median	85,228	100,815	90,108
GMT	86,343	101,329	95,009

Fig. S5. nAb titers against Omicron BA.4/5 pre- and 28 days post-primary-series, third dose, and fourth dose of ChAdOx1 nCoV-19, in participants with no measurable nAb response to Omicron BA.4/5 prior to the fourth dose.

nAb titers were assessed pre- and 28 days post- primary series (dose 2), dose 3, and dose 4 of ChAdOx1 nCoV-19, as presented in Fig. 2B. This post hoc analysis displays data in a subset of participants in the immunogenicity population, who had no measurable nAb response to Omicron BA.4/5 pre-dose 4 (n = 13). The bottom and top edges of the box indicate the first and third quartiles (the difference is the IQR), and the line inside the box is the median. Whiskers extend to the minimum and maximum values, excluding outliers. Any points with a log-transformed value of more than 1.5 x IQR from the respective edges of the box were considered outliers. Boxplots were created based on the log-normal distribution.

GMT, geometric mean titer; IQR, interquartile range; LLoQ, lower limit of quantification; nAb, neutralizing antibody; SARS-CoV-2, severe acute respiratory syndrome coronavirus 2; ULoQ, upper limit of quantification.

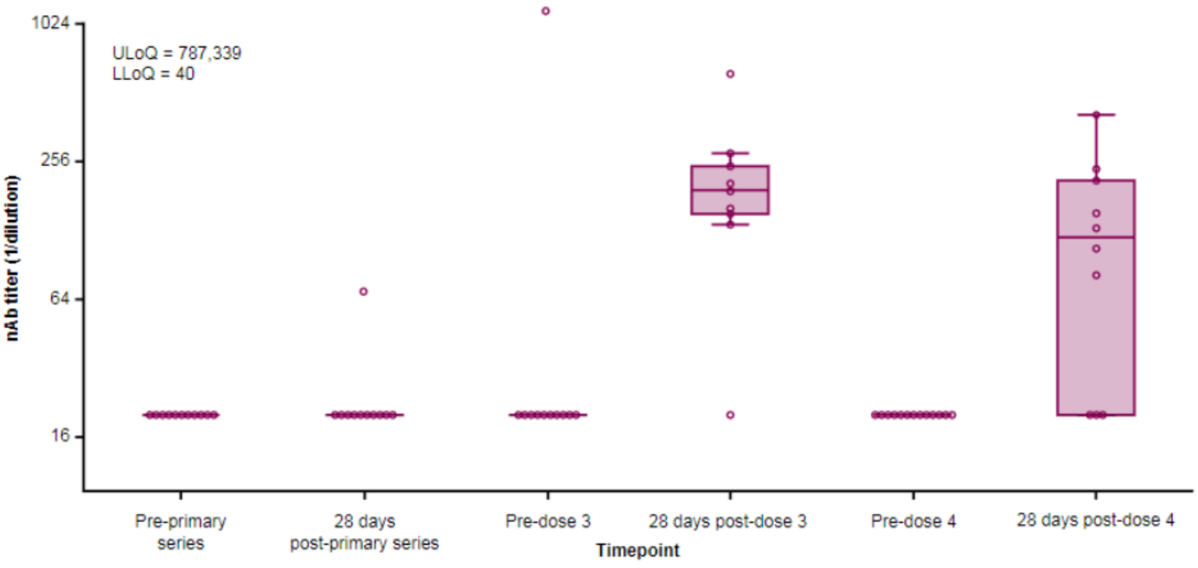

	Pre-primary series	28 days post-primary series	Pre-dose 3	28 days post-dose 3	Pre-dose 4	28 days post-dose 4
n	11	11	11	10	13	10
Median	20.0	20.0	20.0	192.0	20.0	119.5
GMT	20.0	22.4	29.0	170.5	20.0	88.3

Fig. S6. Anti-spike Ab titers against ancestral SARS-CoV-2 at pre- and 28-days post-fourth dose via ELISA.

Anti-spike Ab titers were assessed pre- (n = 44) and 28 days post-dose 4 (n = 43) of ChAdOx1 nCoV-19 in the exploratory fourth-dose subgroup, which allowed inclusion of participants with confirmed SARS-CoV-2 infection within 4 weeks of enrolment to receive a fourth dose. Participants with SARS-CoV-2 infection (reported via 'COVID Diagnosis' case report form) between fourth dose administration and 28 days post-fourth dose were excluded. Paired samples of pre- and post-dose Ab titers for individual participants are displayed using connected lines. The bottom and top edges of the box indicate the first and third quartiles (the difference is the IQR), and the line inside the box is the median. Whiskers extend to 1.5 x IQR from the respective edges of the box.

Data cutoff: September 11, 2023.

Ab, antibody; CI; confidence interval; ELISA, enzyme-linked immunosorbent assay; GMFR, geometric mean fold rise; GMT, geometric mean titer; IQR, interquartile range; SARS-CoV-2, severe acute respiratory syndrome coronavirus 2.

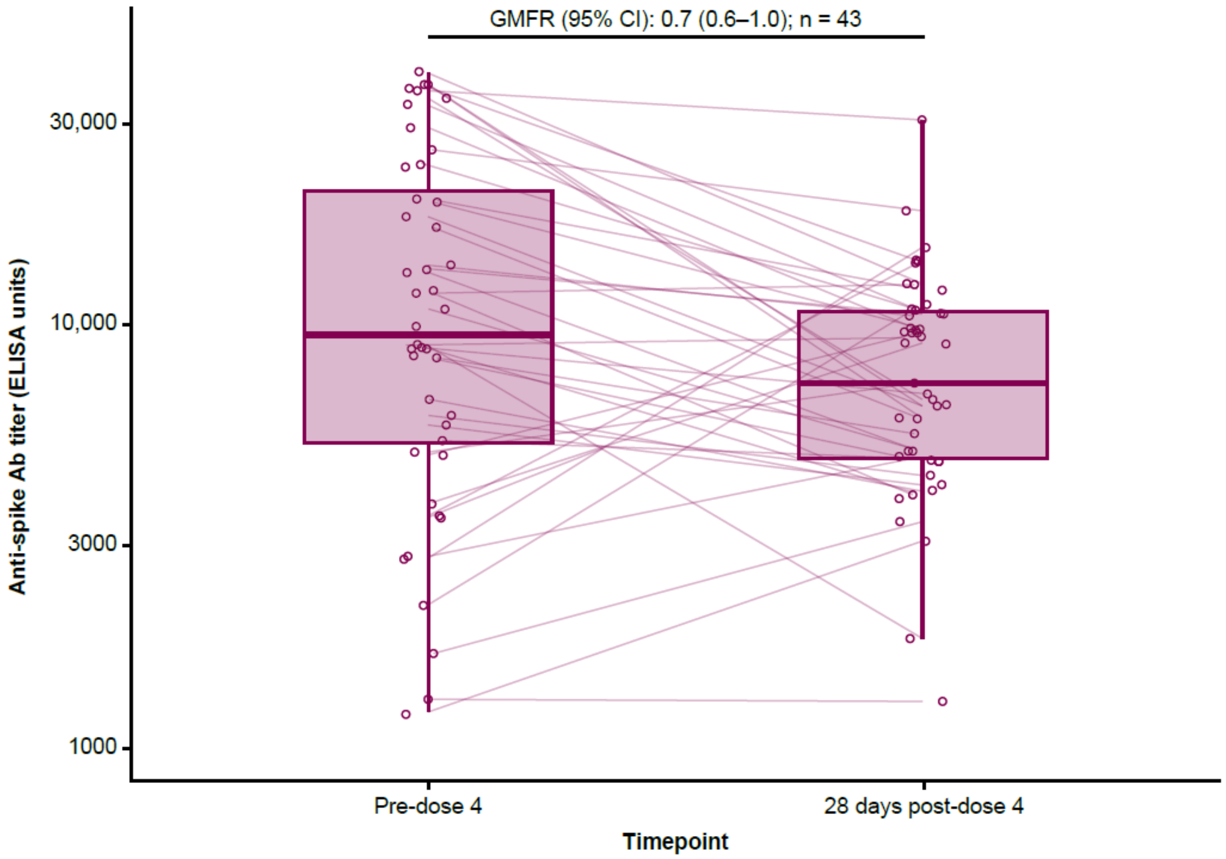

	Pre-dose 4	28 days post-dose 4
n	44	43
Median	9484	7302
GMT	9815	7220

Supplementary Tables

Table S1. Participant demographics and clinical characteristics in key analysis populations collected at baseline (prior to receiving the primary series, unless indicated otherwise).

The safety analysis population included all substudy participants who received a fourth dose of ChAdOx1 nCoV-19. The immunogenicity population included participants who received a fourth dose of ChAdOx1 nCoV-19 within the substudy and had Ab measurements for baseline (pre-primary series), 28 days post-primary series (dose 2), and at least one post-booster timepoint (28 days post-dose 3 or 4).

[a]Collected prior to receiving a fourth dose

[b]Each race category counted participants who selected that category. Arab was counted under White.

[c]Seropositivity was defined as an above-threshold (≥19904 AU/mL) anti-SARS-CoV-2 nucleocapsid assay value.

[d]Comorbidities included BMI ≥30 kg/m^2, cardiovascular disorders, respiratory diseases, and diabetes.

Ab, antibody; AU, arbitrary units; BMI, body mass index; SARS-CoV-2, severe acute respiratory syndrome coronavirus 2; SD, standard deviation.

	**Fourth dose of ChAdOx1 nCoV-19 (n = 172)**	
**Characteristic**	**Safety population (n = 172)**	**Immunogenicity population (n = 142)**
**Age (years)[a]**		
**Mean (SD)**	39.81 (13.43)	40.26 (13.65)
**Median (range)**	36.78 (20.5–78.3)	37.23 (20.5–78.3)
**Age group,[a] n (%)**		
**18–64 years**	163 (94.8)	133 (93.7)
**≥65 years**	9 (5.2)	9 (6.3)
**Sex, n (%)**		
**Male**	96 (55.8)	84 (59.2)
**Female**	76 (44.2)	58 (40.8)
**Race,[b] n (%)**		
**White**	110 (64.0)	90 (63.4)
**Black**	22 (12.8)	17 (12.0)
**Asian**	1 (0.6)	0
**Mixed**	29 (16.9)	27 (19.0)
**Other**	8 (4.7)	7 (4.9)
**Unknown**	2 (1.2)	1 (0.7)
**Bmi category, n (%)**		
**<30 kg/m^2**	113 (65.7)	89 (62.7)
**≥30 kg/m^2**	26 (15.1)	21 (14.8)
**Missing**	33 (19.2)	32 (22.5)
**Serostatus,[a,c] n (%)**		
**Negative**	114 (66.3)	94 (66.2)
**Positive**	56 (32.6)	46 (32.4)
**Missing**	2 (1.2)	2 (1.4)
**Cardiovascular disorder, n (%)**		
**No**	153 (89.0)	126 (88.7)
**Yes**	19 (11.0)	16 (11.3)
**Respiratory disease, n (%)**		
**No**	158 (91.9)	129 (90.8)

Characteristic	Fourth dose of ChAdOx1 nCoV-19 (n = 172) Safety population (n = 172)	Immunogenicity population (n = 142)
Yes	14 (8.1)	13 (9.2)
**Diabetes, n (%)**		
No	169 (98.3)	139 (97.9)
Yes	3 (1.7)	3 (2.1)
**Comorbidity,[d] n (%)**		
No	94 (54.7)	72 (50.7)
Yes	50 (29.1)	43 (30.3)
Missing	28 (16.3)	27 (19.0)
**Current smoker, n (%)**		
No	162 (94.2)	132 (93.0)
Yes	10 (5.8)	10 (7.0)
**Former smoker, n (%)**		
No	147 (85.5)	118 (83.1)
Yes	15 (8.7)	14 (9.9)
Missing	10 (5.8)	10 (7.0)

Table S2. Immunogenicity outcomes comparisons of nAb and anti-spike Ab titers against multiple SARS-CoV-2 variants.

nAb titers against **(A)** ancestral SARS-CoV-2 and **(B)** the Omicron BA.4/5 variant, and anti-spike Ab titers against **(C)** ancestral SARS-CoV-2 and **(D)** Omicron BA.1, **(E)** Alpha, **(F)** Beta, **(G)** Gamma, and **(H)** Delta variants were assessed in the immunogenicity population. GMFRs were calculated in participants with Ab measurements at both compared timepoints.

Ab, antibody; AU, arbitrary units; CI, confidence interval; GMFR, geometric mean fold rise; GMT, geometric mean titer; n, number of participants with Ab measurements at both compared visits; nAb, neutralizing antibody; SARS-CoV-2, severe acute respiratory syndrome coronavirus 2.

A. nAb titers – ancestral SARS-CoV-2 (1/dilution)

Visit	n	GMT (95% CI)	Reference visit	Reference GMT (95% CI)	GMFR from reference (95% CI)
**Primary immunogenicity outcome**					
28 days post-dose 4	112	1048.0 (891.4, 1232.1)	28 days post-dose 3	545.0 (461.7, 643.3)	1.9 (1.6, 2.4)
**Additional immunogenicity outcomes**					
28 days post-dose 4	96	1099.5 (941.9, 1283.6)	28 days post-primary series	132.0 (101.0, 172.4)	8.3 (6.4, 10.9)
28 days post-dose 3	100	562.9 (466.7, 679.0)	28 days post-primary series	137.7 (105.6, 179.6)	4.1 (3.1, 5.3)

B. nAb titers – Omicron BA.4/5 (1/dilution)

Visit	n	GMT (95% CI)	Reference visit	Reference GMT (95%CI)	GMFR from reference (95% CI)
**Additional immunogenicity outcomes**					
28 days post-dose 4	77	702.2 (529.2, 931.7)	28 days post-dose 3	170.7 (133.7, 217.9)	4.1 (2.9, 5.9)
28 days post-dose 4	78	685.0 (517.4, 907.0)	28 days post-primary series	20.9 (19.6, 22.4)	32.7 (24.5, 43.7)
28 days post-dose 3	87	157.7 (124.2, 200.3)	28 days post-primary series	21.4 (19.8. 23.3)	7.4 (5.7, 9.4)

C. Anti-spike Ab titers – ancestral SARS-CoV-2 (AU/mL)

Visit	n	GMT (95% CI)	Reference visit	Reference GMT (95%CI)	GMFR from reference (95% CI)
**Additional immunogenicity outcomes**					
28 days post-dose 4	127	100987.1 (88816.4, 114825.6)	28 days post-dose 3	64268.1 (53345.3, 77427.4)	1.6 (1.3, 1.9)
28 days post-dose 4	129	102075.7 (89867.0, 115943.0)	28 days post-primary series	20986.5 (18133.2, 24288.9)	4.9 (4.1, 5.8)
28 days post-dose 3	138	63689.0 (53391.0, 75973.1)	28 days post-primary series	21543.2 (18677.8, 24848.1)	3.0 (2.4, 3.6)

D. Anti-spike Ab titers - Omicron BA.1 (AU/mL)

Visit	n	GMT (95% CI)	Reference visit	Reference GMT (95%CI)	GMFR from reference (95% CI)
**Additional immunogenicity outcomes**					
28 days post-dose 4	127	25082.4 (21835.9, 28811.7)	28 days post-dose 3	14913.0 (12388.1, 17952.6)	1.7 (1.4, 2.0)
28 days post-dose 4	128	25231.6 (21954.2, 28998.3)	28 days post-primary series	3566.4 (3059.9, 4156.8)	7.1 (5.9, 8.5)
28 days post-dose 3	137	14809.5 (12406.9, 17677.5)	28 days post-primary series	3626.8 (3121.3, 4214.1)	4.1 (3.3, 5.0)

E. Anti-spike Ab titers – Alpha (AU/mL)

Visit	n	GMT (95% CI)	Reference visit	Reference GMT (95%CI)	GMFR from reference (95% CI)
**Additional immunogenicity outcomes**					
28 days post-dose 4	127	78867.8 (69335.0, 89711.4)	28 days post-dose 3	47150.6 (39146.3, 56791.7)	1.7 (1.4, 2.0)
28 days post-dose 4	129	79806.1 (70244.8, 90668.9)	28 days post-primary series	14202.7 (12258.9, 16454.8)	5.6 (4.7, 6.7)
28 days post-dose 3	138	47209.7 (39604.1, 56275.9)	28 days post-primary series	14644.4 (12672.8, 16922.7)	3.2 (2.6, 3.9)

F. Anti-spike Ab titers – Beta (AU/mL)

Visit	n	GMT (95% CI)	Reference visit	Reference GMT (95%CI)	GMFR from reference (95% CI)
**Additional immunogenicity outcomes**					
28 days post-dose 4	127	63722.7 (55727.2, 72865.5)	28 days post-dose 3	36247.8 (30153.9, 43573.4)	1.8 (1.4, 2.1)
28 days post-dose 4	129	64400.3 (56347.5, 73604.0)	28 days post-primary series	10009.6 (8618.0, 11625.9)	6.4 (5.4, 7.7)
28 days post-dose 3	138	36231.0 (30438.3, 43126.3)	28 days post-primary series	10227.4 (8822.7, 11855.7)	3.5 (2.9, 4.3)

G. Anti-spike Ab titers – Gamma (AU/mL)

Visit	n	GMT (95% CI)	Reference visit	Reference GMT (95%CI)	GMFR from reference (95% CI)
**Additional immunogenicity outcomes**					
28 days post-dose 4	127	69533.3 (60944.4, 79332.5)	28 days post-dose 3	40406.7 (33778.9, 48335.0)	1.7 (1.4, 2.1)
28 days post-dose 4	129	70185.5 (61568.6, 80008.3)	28 days post-primary series	11777.9 (10129.8, 13694.2)	6.0 (5.0. 7.2)
28 days post-dose 3	138	40028.0 (33780.3, 47431.2)	28 days post-primary series	11992.9 (10351.9, 13893.9)	3.3 (2.7, 4.1)

H. Anti-spike Ab titers – Delta (AU/mL)

Visit	n	GMT (95% CI)	Reference visit	Reference GMT (95%CI)	GMFR from reference (95% CI)
**Additional immunogenicity outcomes**					
28 days post-dose 4	126	74965.2 (65868.9, 85317.8)	28 days post-dose 3	45059.9 (37483.0, 54168.3)	1.7 (1.4, 2.0)
28 days post-dose 4	128	75650.7 (66509.1, 86048.9)	28 days post-primary series	12939.5 (11180.1, 14975.9)	5.8 (4.9, 7.0)
28 days post-dose 3	137	45017.5 (37813.4, 53594.1)	28 days post-primary series	13265.0 (11477.5, 15330.9)	3.4 (2.8, 4.1)

Table S3. Solicited AEs within 7 days after the fourth dose of ChAdOx1 nCoV-19 by severity.

Local and systemic AEs known to be associated with vaccination were collected as solicited AEs for 7 days after fourth dose administration in a subset of participants in the safety population. Severity was graded on a 4-point scale: mild (Grade 1), moderate (Grade 2), severe (Grade 3), or potentially life-threatening (Grade 4). Participants with multiple events in the same category are counted once in that category. Participants with events in more than one category are counted once in each of those categories. The number of participants with non-missing data was used as the denominator for percentage calculations.

[a]For Redness and Swelling, severity grading was derived based on the reported value. Bruising was not collected as a solicited AE in COV003.

AE, adverse event; ER, emergency room; IwA interfere with activity; PDA, prevent daily activity.

**Solicited AEs**		**Fourth dose of ChAdOx1 nCoV-19 (n = 58)**<br>**n (%) of participants**
**Local solicited AEs**		
**Any**	Any severity	35 (60.3)
	1: Mild	24 (41.4)
	2: Moderate	11 (19.0)
	3: Severe	0
	4: ER or hospitalization	0
**Pain**	Any severity	34 (58.6)
	1: Mild	24 (41.4)
	2: Moderate	10 (17.2)
	3: Severe	0
	4: ER or hospitalization	0
**Tenderness**	Any severity	21 (36.2)
	1: Mild	16 (27.6)
	2: Moderate	5 (8.6)
	3: Severe	0
	4: ER or hospitalization	0
**Redness**[A]	Any severity	0
	1: 2.5–5 cm	0
	2: 5.1–10 cm	0
	3: >10 cm	0
	4: Necrosis or exfoliative dermatitis	0
	Missing	1
**Warmth**	Any severity	6 (10.3)
	1: Mild	5 (8.6)
	2: Moderate	1 (1.7)
	3: Severe	0
	4: ER or hospitalization	0
**Itch**	Any severity	3 (5.2)
	1: Mild	3 (5.2)
	2: Moderate	0
	3: Severe	0
	4: ER or hospitalization	0
**Swelling**[A]	Any severity	0
	1: 2.5–5 cm and no IwA	0
	2: 5.1–10 cm or IwA	0
	3: >10 cm or PDA	0
	4: Necrosis	0
**Induration**	Any severity	1 (1.7)
	1: 2.5–5 cm and no IWA	0

Solicited AEs		Fourth dose of ChAdOx1 nCoV-19 (n = 58) n (%) of participants
	2: 5.1–10 cm or IWA	1 (1.7)
	3: >10 cm or PDA	0
	4: Necrosis	0
**Systemic solicited AEs**		
**Any**	Any severity	25 (43.1)
	1: Mild	13 (22.4)
	2: Moderate	10 (17.2)
	3: Severe	2 (3.4)
	4: ER or hospitalization	0
**Fever**	Any severity	0
	1: 38.0–38.4°C	0
	2: 38.5–38.9°C	0
	3: 39.0–40°C	0
	4: >40°C	0
	Missing	2
**Feverishness**	Any severity	3 (5.2)
	1: Mild	1 (1.7)
	2: Moderate	2 (3.4)
	3: Severe	0
	4: ER or hospitalization	0
**Chills**	Any severity	3 (5.2)
	1: Mild	2 (3.4)
	2: Moderate	1 (1.7)
	3: Severe	0
	4: ER or hospitalization	0
**Joint pain**	Any severity	3 (5.2)
	1: Mild	1 (1.7)
	2: Moderate	2 (3.4)
	3: Severe	0
	4: ER or hospitalization	0
**Muscle pain**	Any severity	6 (10.3)
	1: Mild	2 (3.4)
	2: Moderate	3 (5.2)
	3: Severe	1 (1.7)
	4: ER or hospitalization	0
**Fatigue**	Any severity	13 (22.4)
	1: Mild	8 (13.8)
	2: Moderate	4 (6.9)
	3: Severe	1 (1.7)
	4: ER or hospitalization	0
**Headache**	Any severity	21 (36.2)
	1: Mild	12 (20.7)
	2: Moderate	8 (13.8)
	3: Severe	1 (1.7)
	4: ER or hospitalization	0
**Malaise**	Any severity	8 (13.8)
	1: Mild	3 (5.2)
	2: Moderate	4 (6.9)
	3: Severe	1 (1.7)
	4: ER or hospitalization	0
**Nausea**	Any severity	4 (6.9)
	1: Mild	3 (5.2)
	2: Moderate	1 (1.7)
	3: Severe	0

Solicited AEs		Fourth dose of ChAdOx1 nCoV-19 (n = 58)<br>n (%) of participants
	4: ER or hospitalization	0
**Vomiting**	Any severity	0
	1: Mild	0
	2: Moderate	0
	3: Severe	0
	4: ER or hospitalization	0

Table S4. Unsolicited AEs within 28 days after the fourth dose of ChAdOx1 nCoV-19 by severity.

Unsolicited AEs were collected for 28 days after fourth dose administration in participants in the safety population. Unsolicited AEs are listed by preferred term, according to MedDRA version 23.1. Severity was graded on a 4-point scale: mild (Grade 1), moderate (Grade 2), severe (Grade 3), or potentially life-threatening (Grade 4). Participants with multiple events in the same category are listed once under the maximum severity level reported. Participants with events in more than one category are thus counted once in each of those categories.

[a]Each participant is only counted once, based on the unsolicited AE with the maximal severity reported, for this overall summary.

AE, adverse event; MedDRA, Medical Dictionary for Regulatory Activities.

**Preferred term**		**Fourth dose of ChAdOx1 nCoV-19 (n = 172) n (%) of participants**
**Any Unsolicited AE[A]**	**Any severity**	**22 (12.8)**
	1: Mild	9 (5.2)
	2: Moderate	13 (7.6)
	3: Severe	0
	4: Potentially life-threatening	0
**Asthenia**	**Any severity**	**2 (1.2)**
	1: Mild	1 (0.6)
	2: Moderate	1 (0.6)
	3: Severe	0
	4: Potentially life-threatening	0
**Influenza**	**Any severity**	**2 (1.2)**
	1: Mild	0
	2: Moderate	2 (1.2)
	3: Severe	0
	4: Potentially life-threatening	0
**Rhinitis**	**Any severity**	**2 (1.2)**
	1: Mild	1 (0.6)
	2: Moderate	1 (0.6)
	3: Severe	0
	4: Potentially life-threatening	0
**Allergic pharyngitis**	**Any severity**	**1 (0.6)**
	1: Mild	0
	2: Moderate	1 (0.6)
	3: Severe	0
	4: Potentially life-threatening	0
**Axillary pain**	**Any severity**	**1 (0.6)**
	1: Mild	1 (0.6)
	2: Moderate	0
	3: Severe	0
	4: Potentially life-threatening	0
**Blood pressure fluctuation**	**Any severity**	**1 (0.6)**
	1: Mild	0
	2: Moderate	1 (0.6)
	3: Severe	0
	4: Potentially life-threatening	0
**Contusion**	**Any severity**	**1 (0.6)**
	1: Mild	0

Preferred term		Fourth dose of ChAdOx1 nCoV-19 (n = 172) n (%) of participants
	2: Moderate	1 (0.6)
	3: Severe	0
	4: Potentially life-threatening	0
**Cough**	**Any severity**	**1 (0.6)**
	1: Mild	1 (0.6)
	2: Moderate	0
	3: Severe	0
	4: Potentially life-threatening	0
**Dysphonia**	**Any severity**	**1 (0.6)**
	1: Mild	0
	2: Moderate	1 (0.6)
	3: Severe	0
	4: Potentially life-threatening	0
**Ecchymosis**	**Any severity**	**1 (0.6)**
	1: Mild	1 (0.6)
	2: Moderate	0
	3: Severe	0
	4: Potentially life-threatening	0
**Eczema**	**Any severity**	**1 (0.6)**
	1: Mild	1 (0.6)
	2: Moderate	0
	3: Severe	0
	4: Potentially life-threatening	0
**Gastroesophageal reflux disease**	**Any severity**	**1 (0.6)**
	1: Mild	0
	2: Moderate	1 (0.6)
	3: Severe	0
	4: Potentially life-threatening	0
**Hypersomnia**	**Any severity**	**1 (0.6)**
	1: Mild	1 (0.6)
	2: Moderate	0
	3: Severe	0
	4: Potentially life-threatening	0
**Joint dislocation**	**Any severity**	**1 (0.6)**
	1: Mild	0
	2: Moderate	1 (0.6)
	3: Severe	0
	4: Potentially life-threatening	0
**Lymphadenopathy**	**Any severity**	**1 (0.6)**
	1: Mild	1 (0.6)
	2: Moderate	0
	3: Severe	0
	4: Potentially life-threatening	0
**Migraine**	**Any severity**	**1 (0.6)**
	1: Mild	0
	2: Moderate	1 (0.6)
	3: Severe	0
	4: Potentially life-threatening	0
**Nasal congestion**	**Any severity**	**1 (0.6)**
	1: Mild	1 (0.6)
	2: Moderate	0

Preferred term		Fourth dose of ChAdOx1 nCoV-19 (n = 172) n (%) of participants
	3: Severe	0
	4: Potentially life-threatening	0
**Odynophagia**	**Any severity**	**1 (0.6)**
	1: Mild	1 (0.6)
	2: Moderate	0
	3: Severe	0
	4: Potentially life-threatening	0
**Palpitations**	**Any severity**	**1 (0.6)**
	1: Mild	1 (0.6)
	2: Moderate	0
	3: Severe	0
	4: Potentially life-threatening	0
**Paraesthesia**	**Any severity**	**1 (0.6)**
	1: Mild	1 (0.6)
	2: Moderate	0
	3: Severe	0
	4: Potentially life-threatening	0
**Rhabdomyolysis**	**Any severity**	**1 (0.6)**
	1: Mild	0
	2: Moderate	1 (0.6)
	3: Severe	0
	4: Potentially life-threatening	0
**Rhinorrhoea**	**Any severity**	**1 (0.6)**
	1: Mild	1 (0.6)
	2: Moderate	0
	3: Severe	0
	4: Potentially life-threatening	0
**Scrotal pain**	**Any severity**	**1 (0.6)**
	1: Mild	0
	2: Moderate	1 (0.6)
	3: Severe	0
	4: Potentially life-threatening	0
**Vaccination site erythema**	**Any severity**	**1 (0.6)**
	1: Mild	0
	2: Moderate	1 (0.6)
	3: Severe	0
	4: Potentially life-threatening	0
**Vision blurred**	**Any severity**	**1 (0.6)**
	1: Mild	1 (0.6)
	2: Moderate	0
	3: Severe	0
	4: Potentially life-threatening	0

Table S5. Unsolicited AEs related to study intervention within 28 days after the fourth dose of ChAdOx1 nCoV-19.

Unsolicited AEs were collected for 28 days after fourth dose administration in the safety population and were classified as related to study intervention by the Investigator. Unsolicited AEs relating to study intervention are listed by preferred term, according to MedDRA version 23.1.Participants with multiple events in the same category are counted once in that category. Participants with events in more than one category are counted once in each of those categories.
AE, adverse event; MedDRA, Medical Dictionary for Regulatory Activities.

Preferred term	Fourth dose of ChAdOx1 nCoV-19 (n = 172)<br>n (%) of participants
Any	10 (5.8)
Asthenia	1 (0.6)
Axillary pain	1 (0.6)
Contusion	1 (0.6)
Cough	1 (0.6)
Hypersomnia	1 (0.6)
Lymphadenopathy	1 (0.6)
Nasal congestion	1 (0.6)
Rhinorrhea	1 (0.6)
Scrotal pain	1 (0.6)
Vaccination site erythema	1 (0.6)
Blurred vision	1 (0.6)

Supplementary References

1. Wilkins D, Aksyuk AA, Ruzin A, et al. Validation and performance of a multiplex serology assay to quantify antibody responses following SARS-CoV-2 infection or vaccination. *Clin Transl Immunology* 2022; **11**(4): e1385.
2. Falsey AR, Sobieszczyk ME, Hirsch I, et al. Phase 3 safety and efficacy of AZD1222 (ChAdOx1 nCoV-19) Covid-19 vaccine. N Engl J Med 2021; **385**(25): 2348-60.
3. Folegatti PM, Ewer KJ, Aley PK, et al. Safety and immunogenicity of the ChAdOx1 nCoV-19 vaccine against SARS-CoV-2: a preliminary report of a phase 1/2, single-blind, randomised controlled trial. *Lancet* 2020; **396**(10249): 467-78.
4. Ramasamy MN, Minassian AM, Ewer KJ, et al. Safety and immunogenicity of ChAdOx1 nCoV-19 vaccine administered in a prime-boost regimen in young and old adults (COV002): a single-blind, randomised, controlled, phase 2/3 trial. *Lancet* 2021; **396**(10267): 1979-93.

Redacted for Public Disclosure

**Study Title:**	A Randomized, Controlled, Phase III Study to Determine the Safety, Efficacy, and Immunogenicity of the Non-Replicating ChAdOx1 nCoV-19 Vaccine.

Study Reference: COV003

**Protocol Version:**	13.0
**Date:**	**12 Apr 2022**
**Chief Investigator:**	PPD
**Sponsor:**	University of Oxford
**Funder:**	OXFORD University and external donors (Fundação Lemann, Fundação Brava, Fundação Telles, Instituto D'or de Ensino e Pesquisa and AstraZeneca Brasil, AstraZeneca UK)

Main contacts of the study

**Chief Investigator**	PPD
**Address**	Center for Clinical Vaccinology and Tropical Medicine<br>University of Oxford,<br>Churchill Hospital, Old Road, Headington<br>Oxford, OX3 7LE<br>Email:PPD
**Local Principal Investigator**	PPD<br>Centro de Referência para Imunobiológicos Especiais - CRIE<br>Universidade Federal de São Paulo - UNIFESP<br>Rua Borges Lagoa, 770 - Vila Clementino<br>São Paulo - SP, 04038-001, Brazil<br>Email:PPD
**Co-Principal Investigator – National Coordination**	PPD<br>PPD<br>PPD<br>Institute for Global Health - Siena University<br>Via Val Di Montone, 1<br>Siena, 53-100, Italy<br><br>Centro de Referência para Imunobiológicos Especiais - CRIE<br>Universidade Federal de São Paulo - UNIFESP<br>Rua Borges Lagoa, 770 - Vila Clementino<br>São Paulo - SP, 04038-001, Brazil<br>Email:PPD
**Co-Investigators**	PPD<br>PPD<br><br>Oxford Vaccine Group<br>Center for Clinical Vaccinology and Tropical Medicine

University of Oxford,
Churchill Hospital, Old Road, Headington
Oxford, OX3 7LE

Study Sites

Centro de Referência para Imunobiológicos Especiais - CRIE
Universidade Federal de São Paulo - UNIFESP
Rua Borges Lagoa, 770 - Vila Clementino
São Paulo - SP, 04038-001, Brazil

Instituto D'Or de Pesquisa e Ensino - I'Dor
Rua Diniz Cordeiro, 30 - Botafogo
Rio de Janeiro - RJ, 22281-100, Brazil

Co-Participant Sites:

Hospital Quinta D'or

Rua Almirante Baltazar,383 – São Cristovão

Rio de Janeiro - RJ

Hospital Gloria D'or

Rua Santo Amaro, 80

Rio de Janeiro - RJ

Instituto D'Or de Pesquisa e Ensino - I'Dor
Avenida São Rafael, 2152 - São Marcos
Salvador - BA, 41253-190, Brazil

Centro de Pesquisas Clinicas de Natal (CPCLIN)
Rua Dr Ponciano Barbosa, 282 – Cidade Alta
Natal – RN, 59025-050, Brazil

	Universidade Federal de Santa Maria (UFSM)<br>Av. Roraima, 1000 Cidade Universitária Bairro Camobi<br>Santa Maria - RS, 97105-900, Brazil<br><br>Hospital das Clínicas de Porto Alegre<br>Universidade Federal do Rio Grande do Sul (UFRGS).<br>Rua Ramiro Barcelos, 2.350 Bairro Santa Cecília<br>Porto Alegre – RS, 90035-903, Brazil
**Sponsor Institution**	University of Oxford
**Funder**	University of Oxford and external donors (Fundação Lemann, Fundação Brava, Fundação Telles, Instituto D'or de Ensino e Pesquisa and AstraZeneca Brasil, AstraZeneca UK)

Confidentiality Statement

This document contains confidential information that should not be disclosed to anyone other than the Sponsor, the Research Team, the host organization, members of the Research Ethics Committee, and other regulatory bodies. This information may not be used for any purpose other than evaluating or conducting clinical research without the prior written consent of the Chief Investigator.

Compliance Statement

The study will be conducted in compliance with the Protocol, the principles of Good Clinical Practices, Standards for Medicines for Human Use (Clinical Trial) 2004 (as amended), and all other applicable regulatory requirements.

Investigator's Agreement and Conflict of Interest Notification

I approve this Protocol for use in the abovementioned clinical trial and agree to comply with all provisions established therein.

In accordance with the current revision of the Declaration of Helsinki, I have read this protocol, and declare that I have no conflict of interest.

Lead Investigator	Signature	Date

Research Site

I have read this protocol and agree to comply with all the provisions established therein.

In accordance with the current revision of the Declaration of Helsinki, I have read this protocol, and declare that I have no conflict of interest.

PPD	PPD<br>PPD (Sep 8, 2022 15:52 GMT+1)	08-Sep-2022
Principal Investigator	Signature	Date

TABLE OF CONTENTS

3.1 Background 19

3.2 Pre-clinical Studies 20

3.2.1 Immunogenicity (Jenner Institute) 20

3.2.2 Efficacy 23

3.2.3 Immunopathology and antibody-dependent potentiation 23

3.3 Previous clinical experience 24

3.4 Rationale 31

5.1 Study groups 40

5.2 Study participants 41

5.3 Definition of the End of Study 42

5.4 Potential risks for participants 42

5.5 Known potential benefits 43

6.1 Identification of Participants 44

6.2 Informed Consent Form 44

6.3 Inclusion and exclusion criteria 46

6.3.1 Inclusion Criteria 46

6.3.2 Exclusion Criteria 47

6.3.3 Re-vaccination exclusion criteria (two-dose groups only) 48

6.3.4 Exclusion Criteria for the third dose 49

6.3.5 Exclusion Criteria for the fourth dose 49

6.3.6 Effective birth control for volunteers 49

6.3.7 Withdrawal of participants 50

6.4 Pregnancy 51

7.1 Visit Schedule 51

7.2 Observations, medical history and physical examination 51

7.3 Blood samples, nose/throat swabs and urine analysis 52

7.4 Study visits 53

7.4.1 Screening visit and recruitment 53
7.4.2 Day 0: Recruitment and vaccination visit 54
7.4.2.1 Vaccination 54
7.4.3 Later visits 55
7.4.4 Symptomatic participants from groups 1a, 1b, 1c, 1d and 1e 65
7.4.4.1 Diagnostic SARS-CoV-2 PCR testing outside of the trial 65
7.4.4.2 COVID-19 testing visit 65
7.4.4.3 COVID-19 Testing plus 7 day phone call 66
7.4.4.4 Positive SARS-CoV-2 PCR test and vaccination 66
7.4.5 Symptomatic participants from Protocol 13 onwards 67
7.4.6 Review of the medical record 67
7.4.7 Randomization, blinding, and unblinding 68
7.4.8 Unblinded participants who are eligible to receive an approved or licensed SARS-CoV-2 vaccine 68
7.4.9 Eligible participants who will receive a third dose of ChAdOx1 nCoV-19 vaccine 70
7.4.10 Eligible participants who will receive a fourth dose of ChAdOx1 nCoV-19 vaccine 70

8.1 Description of ChAdOx1 nCoV-19 70

8.2 Storage 71

8.3 Administration 71

8.4 Rationale for selected dose 71

8.5 Environmental contamination control (GMO) 73

8.6 Investigational Medicinal Product for the 4th dose study 74

8.7 Control Vaccine 74

8.8 Placebo 75

8.9 Compliance with the investigational treatment 75

8.10 Investigational treatment accounting 75

8.11 Concomitant medication 75

8.12 Provision of treatment for controls 76

9.1 Definitions 76

9.1.1 Adverse Event (AE) 76

9.1.2 Adverse Reaction (AR) 76
9.1.3 Serious Adverse Event (SAE) 76
9.1.4 Serious Adverse Reaction (SAR) 77
9.1.5 Suspected Unexpected Serious Adverse Reaction (SUSAR) 77

9.2 Expectation 77

9.3 Predicted/expected adverse reactions: 78

9.4 Adverse Events of Special Interest 78

9.5 Causality 78

9.6 Reporting procedures for all adverse events 79

9.7 Evaluation of severity 80

9.8 Serious AE reporting procedures 82

9.9 Procedures for reporting SUSARs 83

9.10 Safety assessments 83

9.11 Data Safety Monitoring Committee 83

10.1 Description of statistical methods 84

10.2 Efficacy Outcomes 84

10.2.1 Efficacy 85
10.2.2 Safety and Reactogenicity 86
10.2.3 Immunogenicity 86

10.3 3rd and 4th dose studies 87

10.4 Subgroup analyses 87

10.5 Number of Participants 88

1.1.1 Fourth dose boost substudy 88

10.6 Interim and primary analyses of the primary outcome 89

10.7 Final Analysis 89

10.8 Inclusion in the analysis 90

10.9 Data and Safety Monitoring Committee 90

11.1 Data processing 90
11.2 Record keeping 91
11.3 Source data and technical data sheets (CRFs) 91
11.4 Data protection 91
11.5 Data quality 92
11.6 Archiving 92
12.1 Investigator's Procedures 93
12.2 Monitoring 93
12.3 Deviation from the protocol 93
12.4 Audit and inspection 93
13.1 Declaration of Helsinki 93
13.2 Guidelines for good clinical practices 93
13.3 Ethical and regulatory approvals 94
13.4 Volunteers confidentiality 94
14.1 Funding 95
14.2 Insurance 95
14.3 Publication Policy 95

1 SYNOPSIS

**Title**	A Randomized, Controlled, Phase III Study to Determine the Safety, Efficacy, and Immunogenicity of the Non-Replicating ChAdOx1 nCoV-19 Vaccine.
**Study Identifier**	COV003
**Trial Record**	https://www.isrctn.com/ (registration number: ISRCTN89951424)
**Study Sites**	Centro de Referência para Imunobiológicos Especiais (CRIE) - UNIFESP, Universidade Federal de São Paulo, Rua Borges Lagoa, 770, Vila Clementino, Zip Code 04038-001, São Paulo/SP, Brazil<br><br>Instituto D'Or de Pesquisa e Ensino<br>Rua Diniz Cordeiro, 30, Botafogo,<br>Zip Code 22281-100, Rio de Janeiro - RJ, Brazil<br><br>Instituto D'Or de Pesquisa e Ensino<br>Avenida São Rafael, 2152, São Marcos,<br>Zip Code 41253-190, Salvador - BA, Brazil<br><br>Centro de Pesquisas Clínicas de Natal (CPCLIN)<br>Rua Dr Ponciano Barbosa, 282 – Cidade Alta<br>Natal – RN, 59025-050, Brazil<br><br>Universidade Federal de Santa Maria (UFSM)<br>Av. Roraima, 1000 Cidade Universitária Bairro Camobi<br>Santa Maria - RS, 97105-900, Brazil<br><br>Hospital das Clínicas de Porto Alegre<br>Universidade Federal do Rio Grande do Sul (UFRGS).

	Rua Ramiro Barcelos, 2.350 Bairro Santa Cecília<br>Porto Alegre – RS, 90035-903, Brazil
**Clinical Phase**	3
**Design**	A Single-Blind, Randomized Study of Safety, Efficacy, and Immunogenicity.
**Population**	Health professionals and adults with high potential for exposure to SARS-CoV-2, aged ≥18 years.
**Planned Sample Size**	The total sample size will be up to 10,300 participants (with a margin of 1%).
**Planned Duration**	11 to 13 months post second vaccine dose, per participant<br>or<br>6 to 7 months post fourth vaccine dose, per participant in Group 2

**MAIN STUDY AND 3RD DOSE OBJECTIVES**		
	**Objective**	**Endpoint Measure**
**Primary Objective**	To evaluate the efficacy of ChAdOx1 nCoV-19 vaccine against COVID-19 disease confirmed with PCR.	a) COVID-19 virologically confirmed symptomatic cases (PCR positive).
**Secondary Objectives**	To evaluate the safety, tolerability, and reactogenicity profile of ChAdOx1 nCoV-19 candidate vaccine.	a) Occurrence of signs and symptoms of local and systemic reactogenicity requested during 7 days after vaccination (in a subset of 200 participants*);<br>b) Occurrence of serious adverse events;<br>c) Occurrence of disease enhancement episodes.

	To evaluate the efficacy of ChAdOx1 nCoV-19 candidate vaccine against severe and non-severe COVID-19 disease.	a) Hospitalization for COVID-19 disease confirmed by PCR;<br>b) COVID-19 severe disease confirmed by PCR;<br>c) Death associated with COVID-19 disease;<br>d) Antibodies against SARS-CoV-2 non-Spike protein (efficacy against non-spike seroconversion rates).
	To assess the humoral immunogenicity of ChAdOx1 nCoV-19 candidate vaccine.	a) Antibodies against SARS-CoV-2 spike protein (sero-conversion rates).<br>b) Virus neutralizing antibodies (NAb) against live and/or pseudotyped SARS-CoV-2 virus.
	To assess the cellular immunogenicity of ChAdOx1 nCoV-19 candidate vaccine.	a) Interferon-gamma (IFN-γ) enzyme-linked immunospot (ELISpot) responses to SARS-CoV-2 spike protein;
	To assess immunological correlates of protection in relation to occurrence of COVID-19 disease in ChAdOx1 nCoV-19 recipients	Immunological endpoints and COVID-19 disease endpoints in ChAdOx1 nCoV-19 recipients
	To evaluate the reactogenicity profile of the third dose of ChAdOx1 nCoV-19 candidate vaccine.	Occurrence of signs and symptoms of local and systemic reactogenicity requested during 7 days after vaccination.
	To determine if the neutralising antibody response to a third dose ChAdOx1 nCoV-19 is non-inferior to the response to the ChAdOx1 nCoV-19 primary series	GMTR of nAb response to the ancestral strain 28 days after a 3rd dose administration vs 28 days after primary series
	To determine if the humoral immune response to a 3rd dose ChAdOx1 nCoV-19 is non-inferior to the	GMTR of Spike binding antibody response to the ancestral strain 28 days after 3rd dose administration vs 28 days after primary series

	response to the ChAdOx1 nCoV-19 primary series	
**4th DOSE OBJECTIVES**		
	**Objective**	**Endpoint Measure**
**Primary Objective**	To determine if the neutralising antibody response to a 4th dose ChAdOx1 nCoV-19 is non-inferior to the response to the first ChAdOx1 nCoV-19 dose in a subgroup of study participants	GMTR of nAb response to the ancestral strain 28 days after 4th dose administration vs 28 days after a 3rd dose
**Secondary Objectives**	To determine if the humoral immune response to a 4th dose ChAdOx1 nCoV-19 is non-inferior to the response to the first ChAdOx1 nCoV-19 booster dose in a subgroup of study participants	a.) GMTR of nAb response to a contemporary variant of concern 28 days after 4th dose administration vs 28 days after a 3rd dose<br>b.) GMTR of Spike binding antibody response to the ancestral strain 28 days after 4th dose administration vs 28 days after a 3rd dose
	To determine if the humoral immune response to a 4th dose ChAdOx1 nCoV-19 is non-inferior to the response to primary series ChAdOx1 nCoV-19 in a subgroup of study participants	a.) GMTR of nAb response to the ancestral strain 28 days after 4th dose administration vs 28 days after primary series ChAdOx1 nCoV-19<br>b.) GMTR of nAb response to a contemporary variant of concern 28 days after 4th dose administration vs 28 days after primary series ChAdOx1 nCoV-19<br>c.) GMTR of Spike binding antibody response to the ancestral strain, variant of concern 28 days after 4th

		dose administration vs 28 days after primary series ChAdOx1 nCoV-19
	To characterise the cellular immune responses of a 4th dose ChAdOx1 nCoV-19 in seronegative and seropositive participants who previously received an initial ChAdOx1 nCoV-19 boost in a subgroup of participants	a.) Cellular immune responses by ICS (Th1/Th2) over time<br>b.) Cellular immune responses by ELISpot over time
	To evaluate the reactogenicity profile of the fourth dose of ChAdOx1 nCoV-19 vaccine.	a) Occurrence of signs and symptoms of local and systemic reactogenicity requested during 7 days after vaccination (in a subset of 100 participants).<br>Recording of unsolicited AEs for 28 days after vaccination
**Exploratory objectives**	To explore anti-vector responses to the ChAdOx-1 vector of a 4th dose ChAdOx1 nCoV-19 in seronegative and seropositive participants who previously received an initial ChAdOx1 nCoV-19 boost	a.) Geometric mean titres of ChAdOx1 neutralising antibody titres over time<br>b.) Pairwise correlations between anti-S, pseudo-neutralisation, and live neutralisation antibody titres over time
	To further evaluate humoral and cellular immunogenicity across all doses through exploratory immunology	Exploratory immunology such as systems serology profiling

**Investigational Products**	a) ChAdOx1 nCoV-19, a replication-defective adenoviral vector expressing the SARS-CoV-2 spike (S) protein;<br>b) MenACWY vaccine;<br>c) Saline Placebo (for the control arm boosting dose)

**Detailed assessments of local and systemic reactogenicity for 7 days after vaccination with ChAdOx1 nCoV-19 compared to MenACWY as a control have been documented in a sufficient number of participants in previous studies. In study COV003, detailed local and systemic reactogenicity will be evaluated in 200 randomized participants, who received two doses, a quantity statistically determined to ensure proportionality and comparative representativeness compared to studies COV001 and COV002. Local and systemic reactogenicity will be evaluated for 7 days in all participants who received the third dose and in a subset of 100 participants that have received the fourth dose. ** The fourth dose will be administered to a subset of 350 participants who have previously received 3 doses of ChAdOx1 nCoV-19 vaccine without prior confirmed COVID-19 infections.*

**Formulation**	ChAdOx1 nCoV-19: Liquid<br>MenACWY: powder and solvent for solution for injection
**Route of Administration**	Intramuscular (IM)
**Doses per Administration**	ChAdOx1 nCoV-19: 5 x 10^{10} vp<br>ChAdOx1 nCoV-19: 0.5mL (3.5 x 10^{10} to 6.5 x 10^{10})<br>MenACWY: 0.5 mL<br>0.9% saline solution: 0.5mL<br>Both groups will receive prophylactic paracetamol: 500mg - 1 g q6h/24 hours.

2 ABBREVIATIONS

**AdHu**	Human adenovirus
**AdHu5**	Human adenovirus serotype 5
**ADE**	Antibody-Dependent Enhancement
**AE**	Adverse Event
**AESI**	Adverse Event of Special Interest
**AID**	Autoimmune Disease
**CCVTM**	Centre for Clinical Vaccinology and Tropical Medicine, Oxford
**CBF**	Clinical Bio manufacturing Facility
**ChAdOx**	Chimpanzee adenovirus 1
**CI**	Confidence interval
**COP**	Code of Practice
**CRF**	Clinical Record or Clinical Research Facility
**CTRG**	Clinical Trials & Research Governance Office, University of Oxford
**CTL**	Cytotoxic T Lymphocyte
**DSMB**	Data and Safety Monitoring Board
**DSUR**	Development Safety Update Report
**ELISPOT**	Enzyme-linked immunospot
**GCP**	Good Clinical Practices
**GMO**	Genetically modified organism
**GMT**	Geometric mean titer
**GP**	General Practitioner
**HCG**	Human Chorionic Gonadotrophin
**HEK**	Human embryonic kidney
**HLA**	Human leukocyte antigen
**HRA**	Health Research Authority
**IB**	Investigator's Brochure

**ICH**	International Conference on Harmonization
**ICMJE**	International Committee of Medical Journal Editors
**ICS**	Intracellular Cytokine Staining
**ID**	Intradermal
**IFNγ**	Gamma interferon
**IM**	Intramuscular
**IMP**	Investigational medicinal product
**IMP-D**	Investigational Medicinal Product Dossier
**IV**	Intravenous
**MenACWY**	Quadrivalent meningococcal conjugate vaccine (protein-polysaccharide) against group A, C, W, and Y capsular serotype
**MHRA**	Medicines and Healthcare Products Regulatory Agency
**MVA**	Modified Vaccinia Ankara virus
**NHS**	National Health Service
**NIH**	National Institutes of Health
**NIHR**	National Institute for Health Research
**PBMC**	Peripheral blood mononuclear cell
**PCR**	Polymerase chain reaction
**PI**	Principal Investigator
**QP**	Qualified Person
**qPCR**	Quantitative polymerase chain reaction
**REC**	Research Ethics Committee
**SAE**	Serious adverse event
**SC**	Subcutaneous
**SOP**	Standard Operating Procedure
**SUSAR**	Suspected unexpected serious adverse reaction

**µg**	Microgram
**VP**	viral particle
**VV**	viral vector
**WHO**	World Health Organization

3 BACKGROUND AND RATIONALE

3.1 Background

In December 2019, a group of pneumonia patients of unknown cause was linked to a wholesale seafood market in Wuhan, China, and it was later confirmed that they were infected with a new coronavirus, known as 2019-nCoV [1]. The virus was later renamed SARS-CoV-2 because it is similar to the coronavirus responsible for severe acute respiratory syndrome (SARS-CoV), a *Betacoronavirus* lineage B. SARS-CoV-2 shares more than 79% of its sequence with SARS-CoV, and 50% with the coronavirus responsible for Middle East respiratory syndrome (MERS-CoV), a member of *Betacoronavirus* lineage C[2]. Covid-19 is the infectious disease caused by SARS-CoV-2. In January 2020, there was an increase in evidence of human-to-human transmission as the number of cases began to increase rapidly in China. Despite the unprecedented containment measures adopted by the Chinese government, SARS-CoV-2 has spread rapidly across the world. WHO declared the COVID-19 outbreak as an international public health emergency on January 30, 2020.

Coronaviruses (CoVs) are large, spherical, and enveloped single-stranded RNA genomes. A quarter of its genome is responsible for encoding structural proteins, such as glycoprotein spike (S), envelope (E), membrane (M) and nucleocapsid proteins (N). E, M, and N are mainly responsible for virion assembly, while protein S is involved in binding to the receptor, mediating the entry of the virus into host cells during CoVs infection through different receptors. [3] SARS-CoV-2 belongs to the phylogenetic lineage B of the genus *Betacoronavirus* and recognizes the angiotensin-converting enzyme 2 (ACE2) as an input receptor[4]. This is the seventh CoV that has been proven to cause infections in humans and the third that has been proven to cause serious illness after SARS-CoV and MERS-CoV.

The spike protein is a type I transmembrane, trimeric, type I glycoprotein located on the surface of the viral envelope of CoVs, which can be divided into two functional subunits: the N-terminal S1 and the C-terminal S2. S1 and S2 are responsible for the binding to the cellular receptor through the receptor binding domain (RBD, for its acronym in English) and fusion of virus and cell membranes, respectively, thereby mediating the entry of SARS-CoV-2 into target cells. [3] The functions of S in receptor binding and membrane fusion make it an ideal target for vaccine and antiviral development, as it is the main target for neutralizing antibodies.

ChAdOx1 nCoV-19 vaccine consists of a replication-deficient monkey adenoviral vector ChAdOx1, containing the SARS CoV-2 structural surface glycoprotein antigen (spike protein) (nCoV-19), with a signal sequence from the leading tissue plasminogen activator (tPA). ChAdOx1 nCoV-19 expresses a codon-optimized coding sequence for the Spike protein from GenBank genomic sequence access: MN908947. The leader tPA sequence was shown to be beneficial in increasing the immunogenicity of another CoV vaccine vectorized by ChAdOx1 (ChAdOx1 MERS)[5].

3.2 Pre-clinical Studies

Refer the Investigator's Brochure for the most recent update of preclinical data.

3.2.1 Immunogenicity (Jenner Institute)

The mice (balb/c and CD-1) were immunized with ChAdOx1 which expresses the SARS-CoV-2 Spike protein or green fluorescent protein (GFP). From 9 to 10 days after vaccination, spleen samples were used to assess IFN-γ ELISpot responses and serum samples for assessments of S1 and S2 antibody responses with ELISA. The results of these studies show that a single dose of ChAdOx1 nCoV was immunogenic in mice.

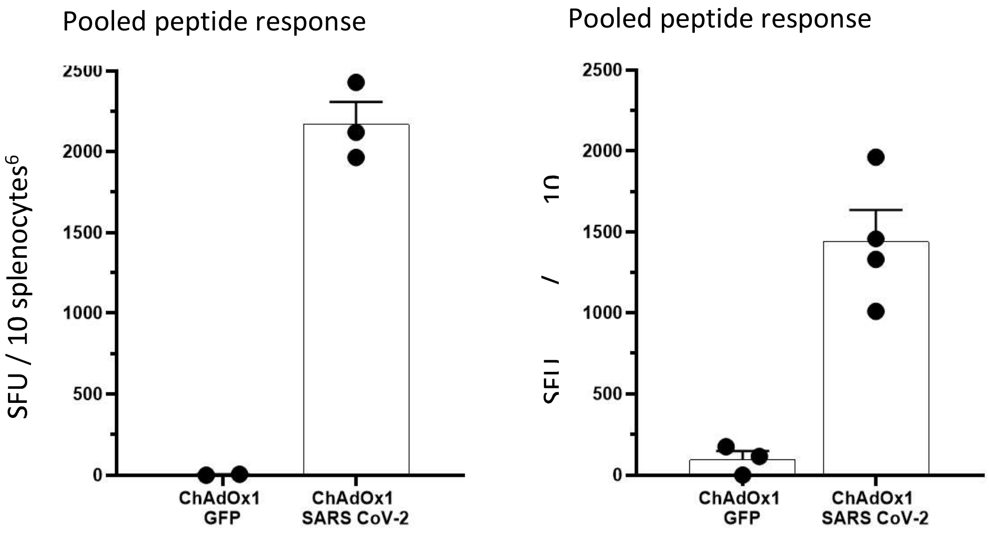

Figure 1. Splenic responses combined with IFN-γ ELISpot from BALB/c (left panel) and CD-1 (right panel) mice, in response to peptides that comprise the SARS-CoV-2 spike protein, nine or ten days after vaccination, with 1.7×10^{10} pv ChAdOx1 nCoV-19 or 8×10^{9} pv ChAdOx1 GFP. The means with SEM are described.

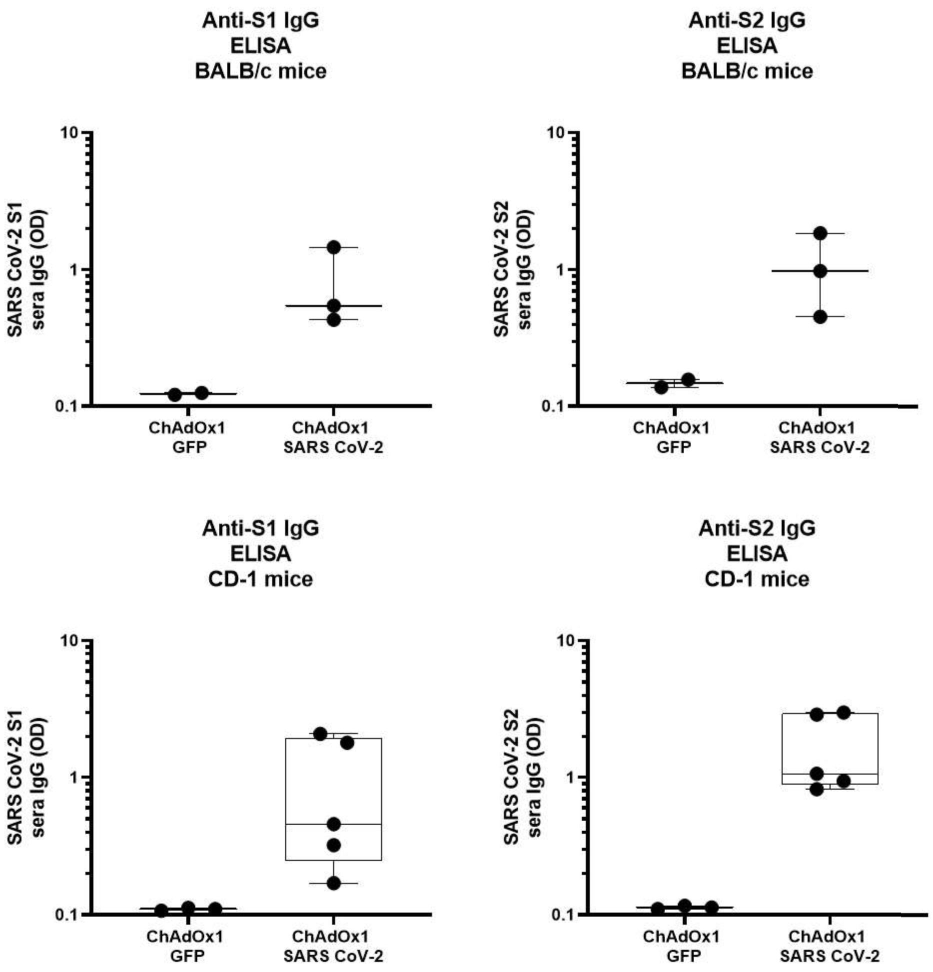

Figure 2. Box and mustache plot of optical densities after ELISA analysis of BALB/C mouse serum (top panel) incubated with purified protein spanning the S1 domain (left) or purified protein spanning the S2 domain (right) of the SARS-CoV spike -2 nine or ten days after vaccination, with 1.7 × 10^{10} pv ChAdOx1 nCoV-19 or 8 × 10^{9} pv ChAdOx1 GFP. Box and mustache plots of optical densities after ELISA analysis of CD-1 mouse serum (bottom panel) incubated with purified protein spanning the S1 domain (left) or purified protein spanning the S2 domain (right) of the SARS-CoV spike -2.

A second experiment was performed with a different dose. The results are summarized in the figure below. Intracellular cytokine staining shows a pattern consistent with predominantly Th1 responses.

A. ELISpot Responses

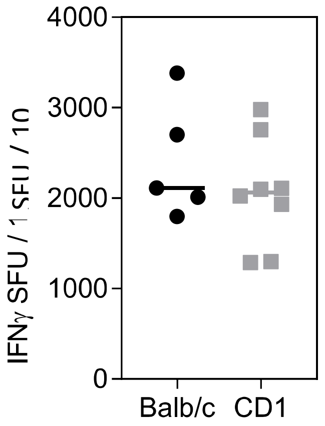

B. Intracellular cytokine staining

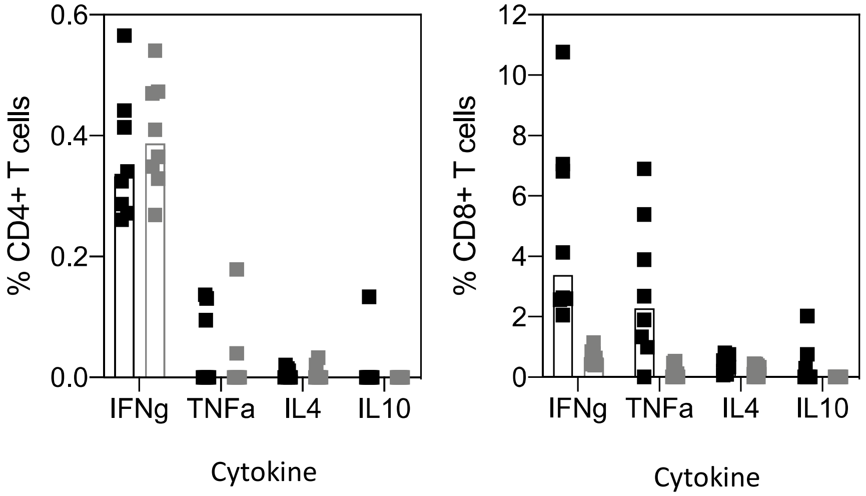

Figure 3. Specific antigen responses after vaccination with ChAdOx nCov19. 10^8 UI ChAdOx nCoV-19 was administered intramuscularly to heterogeneous BALB/c mice (CD1). Fourteen days later, the harvested spleens and cells stimulated peptides that span the extension of S1 and S2.

A. The graph shows the IFN-γ ELIspot responses summed up in BALB/c (black circles) and heterogeneous cd1 (gray squares) mice.
B. The graphs show the frequency of cytokine positive CD4 (left) or CD8 (right) cells, measured by intracellular cytokine staining after splenocyte stimulation with clustered S1 (black) or clustered S2 (gray) peptides in CD1 mice.

3.2.2 Efficacy

Pre-clinical efficacy studies of ChAdOx1 nCoV-19 in ferrets and non-human primates are ongoing. These results are included in the Investigator's Brochure.

3.2.3 Immunopathology and antibody-dependent potentiation

Safety concerns have arisen around the use of coronavirus Spike glycoproteins to their full extent and other viral antigens (nucleoprotein) as vaccine antigens after historical and limited reports of immunopathology and antibody dependent potentiation (ADE) reported *in vitro* and post challenge with SARS-CoV in mice, ferrets and non-human primates immunized with vaccines based on inactivated complete SARS-CoV or protein S in its full extension, including a study that used modified Vaccinia Ankara as a vector. [6-8] So far, there has been a report of pulmonary immunopathology after the challenge with MERS-CoV in mice immunized with a candidate vaccine against inactivated MERS-CoV. [9] However, in preclinical trials of immunization with ChAdOx1 and challenge with MERS-CoV, ADE was not observed in hDPP4 transgenic mice, dromedary camels or non-human primates (van Doremalen et al, submitted manuscript).[10.11]

The risks of inducing pulmonary immunopathology in the case of COVID-19 after vaccination with ChAdOx1 nCoV-19 are unknown. The NHP study conducted by NIH described in the investigator’s brochure showed no evidence of immune-enhanced inflammation in ChAdOx1 nCoV-19 vaccinated animals who underwent SARS-CoV-2 challenge 4 weeks post immunization, at 7 days post challenge. Results from a separate challenge study conducted on a purified inactivated SARS-CoV-2 vaccine also corroborate with NIH findings where no ADE has been detected in vaccinated animals [1]. However, the negative findings on ADE and lung immunopathology from both reports should be interpreted with caution, as challenged animals were sacrificed and examined shortly after challenge (7 days post inoculation). Challenge studies in ferrets and non-human primates (PNH) are ongoing and these pre-clinical trials will report on the presence or absence of pulmonary pathology. The results will be reviewed as soon as they are available and will be part of the risk/benefit discussions for participants who receive the investigational product (IMP). All pathological data from challenge studies of other SARS-CoV-2 candidate vaccines will also be taken into account.

3.3 Previous clinical experience

Prior to current COVID-19 studies, vaccines vectorized by ChAdOx1 that express different inserts have been used previously in more than 320 healthy volunteers participating in clinical trials conducted by University of Oxford in the United Kingdom and abroad (tables 1 and 2). Most importantly, a ChAdOx1 vector vaccine expressing the total Spike protein from another *Betacoronavirus*, MERS-CoVCoV, has been administered to 31 participants, so far, as part of the MERS001 and MERS002 studies. ChAdOx1 MERS was administered in doses ranging from $5x10^9$ pv $5x10^{10}$ pv (table 2) with no serious adverse reactions reported. Further references and results on safety and immunogenicity about ChAdOx1 MERS can be found in the ChAdOx1 Investigator's Brochure nCoV-19.

Clinical trials of ChAdOx1 vectorized vaccines encoding antigens for Influenza (NP + M1 fusion protein), Tuberculosis (85A), Prostate Cancer (5T4), Malaria (LS2), Chikungunya (structural polyprotein), Zika (prM and E), MERS-CoV (total spike protein) and Meningitis B are listed below.

None of the clinical trials mentioned below reported serious adverse events associated with the administration of ChAdOx1, which had a good safety profile.

Table 1. Non-COVID-19 clinical experience with ChAdOx1 viral vector vaccines.

Country	Study	Vaccine	Age	Route	Dose	Number of volunteers (received ChAdOx1)	Publication/Registration Number
UK	FLU004	ChAdOx1 NP + M1	18-50	IM	$5x10^8$ pv	3	Antrobus et al, 2014. Molecular Therapy. DOI: 10.1038/mt. 2013.284 [2]
					$5x10^9$ pv	3	
					$2.5x10^{10}$ pv	3	
					$5x10^{10}$ pv	6	
UK	FLU005	ChAdOx1 NP + M1<br>MVA NP + M1 (week 8)	18-50	IM	$2.5x10^{10}$ pv	12	Coughlan et al, 2018. EBioMedicine DOI: 10.1016/j.ebiom.2018.02.011 DOI: 10.1016/j.ebiom.2018.05.001 [3]
		ChAdOx1 NP + M1<br>MVA NP + M1 (week 52)	18-50	IM	$2.5x10^{10}$ pv	12	
		MVA NP+M1<br>ChAdOx1 NP + M1 (week 8)	18-50	IM	$2.5x10^{10}$ pv	12	
		MVA NP+M1<br>ChAdOx1 NP + M1 (week 52)	18-50	IM	$2.5x10^{10}$ pv	9	
		ChAdOx1 NP + M1	>50	IM	$2.5x10^{10}$ pv	12	

Country	Study	Vaccine	Age	Route	Dose	Number of volunteers (received ChAdOx1)	Publication/Registration Number
		ChAdOx1 NP + M1<br>MVA NP + M1 (week 8)	>50	IM	$2.5x10^{10}$ pv	12	
		ChAdOx1 85A	18-50	IM	$5x10^{9}$ pv	6	Wilkie et al, 2020 Vaccine
					$2.5x10^{10}$ pv	12	
UK	TB034	ChAdOx1 85A<br>MVA85A (week 8)	18-50	IM	$2.5x10^{10}$ pv	12	
		ChAdOx1 85A<br>(x2, 4 weeks apart)<br>MVA85A (in 4 months)	18-50	IM	$2.5x10^{10}$ pv	12	
Switzerland	TB039<br>(ongoing)	ChAdOx1 85A	18-55	Aerosol	$1x10^{9}$ pv	3	Clinicaltrials.gov:<br>NCT04121494
				Aerosol	$5x10^{9}$ pv	3	
				Aerosol	$1x10^{10}$ pv	11	
				Aerosol/IM	$1x10^{10}$ pv	15	
Uganda	TB042<br>(ongoing)	ChAdOx1 85A	18-49	IM	$5x10^{9}$ pv	6	Clinicaltrials.gov:<br>NCT03681860
					$2.5 x10^{10}$	6	

Country	Study	Vaccine	Age	Route	Dose	Number of volunteers (received ChAdOx1)	Publication/Registration Number
UK	VANCE01	ChAdOx1.5T4<br>MVA.5T4	18 - 75	IM	2.5×10^{10} pv	34	Clinicaltrials.gov:<br>NCT02390063
UK	ADVANCE (ongoing)	ChAdOx1.5T4<br>MVA.5T4	≥18	IM	2.5×10^{10} pv	23 (on Feb 20)	Clinicaltrials.gov:<br>NCT03815942
UK	VAC067	ChAdOx1 LS2	18-45	IM	5×10^{9} pv<br>2.5×10^{10} pv	3<br>10	Clinicaltrials.gov:<br>NCT03203421
UK	VAMBOX	ChAdOx1 MenB.1	18-50	IM	2.5×10^{10} pv<br>5×10^{10} pv	3<br>26	ISRCTN46336916
UK	CHIK001	ChAdOx1 Chik	18-50	IM	5×10^{9} pv<br>2.5×10^{10} pv<br>5×10^{10} pv	6<br>9<br>9	Clinicaltrials.gov:<br>NCT03590392<br>DOI: https://doi.org/10.4269/ajtmh.abstract2019<br>Abstract # 59, page 19.
UK	ZIKA001	ChAdOx1 Zika	18-50	IM	5×10^{9} pv	6	Clinicaltrials.gov:

Country	Study	Vaccine	Age	Route	Dose	Number of volunteers (received ChAdOx1)	Publication/Registration Number
	(ongoing)				$2.5x10^{10}$ pv	3 (on Feb 20)	NCT04015648
					$5x10^{10}$ pv	-	

Table 2. Clinical experience with ChAdOx1 MERS

Country	Study	Vaccine	Age	Route	Dose	Number of volunteers (received ChAdOx1)	Publication/Registration Number
UK	MERS001 (ongoing)	ChAdOx1 MERS	18-50	IM	$5x10^9$ pv<br>$2.5x10^{10}$ pv<br>$5x10^{10}$ pv<br>$2.5x10^{10}$ pv<br>Initiation - homologous reinforcement	6<br>9<br>9<br>3	Clinicaltrials.gov: NCT03399578<br>Folegatti et.al. 2020, Lancet Infect.Dis<br>DOI: https://doi.org/10.1016/S1473-3099(20)30160-2
Saudi Arabia	MERS002 (ongoing)	ChAdOx1 MERS	18-50	IM	$5x10^9$ pv<br>$2.5x10^{10}$ pv<br>$5x10^{10}$ pv	4<br>3<br>-	Clinicaltrials.gov: NCT04170829

The first clinical trial of the ChAdOx1 nCoV-19 candidate vaccine (COV001) started on April 23 2020, after approval by the IRB and CTA by the MHRA. ChAdOx1 nCoV-19 candidate vaccine has been studied in the following clinical trials:

- Eight clinical trials evaluating ChAdOx1 nCoV-19 given to adults as a 2-dose primary series: 5 ongoing studies sponsored by the University of Oxford, (COV001, COV002, COV003, COV004 and COV005), 2 ongoing studies sponsored by AstraZeneca (D8110C00001 and D8111C00002), and 1 completed study sponsored by the Serum Institute of India (COVISHIELD).
- One ongoing University of Oxford study evaluates ChAdOx1 nCoV-19 given as a 2-dose primary series to children and adolescents (COV006).
- Five additional clinical trials or sub-studies in adults. Of these:
    - One AstraZeneca study evaluates ChAdOx1 nCoV-19 given as a 2-dose primary series in immunocompromised adults (D8111C0010).
    - Two studies evaluate ChAdOx1 nCoV-19 as a booster immunisation at least 10 months after the first vaccine dose, are sub-studies of COV001 and COV003 (noted above) and are ongoing.
    - Three studies evaluate a 2-dose primary series consisting of ChAdOx1 nCoV-19 and another adenoviral vector-based SARS-CoV-2 vaccine, rAD26-S (CV03872097, formerly known as D8111C00003, CV03872091, and CV03872092), are sponsored by RPharm and are ongoing.
- One study sponsored by AstraZeneca has also evaluated AZD2816, a vaccine that uses the same adenoviral platform as ChAdOx1 nCoV-19 but is based on the Beta variant (B.1.351). The study evaluates both AZD2816 and ChAdOx1 nCoV-19 when given to adults with no prior history of SARS CoV-2 vaccination, and when given as a third dose boost to adults who previously completed a primary vaccination course with ChAdOx1 nCoV-19 or a SARS CoV-2 mRNA vaccine.

These clinical trials are summarised in full in the Investigator’s Brochure.

3.4 Rationale

The epidemic of COVID-19 has caused a major disruption in health systems with significant socioeconomic impacts. Containment measures have failed to stop the spread of the virus, which has reached pandemic levels. Currently, there are no specific treatments available against COVID-19 and accelerated vaccine development is sorely needed.

Live attenuated viruses have historically been one of the most immunogenic platforms available, as they have the ability to present multiple antigens throughout the viral life cycle in their native conformations. However, the manufacture of live attenuated viruses requires complex measures of containment and biosafety. In addition, live attenuated viruses carry the risk of inadequate attenuation, causing widespread disease, particularly in immunocompromised hosts. Given that COVID-19 is a serious and potentially fatal disease, which disproportionately affects elderly people with comorbidities, producing a vaccine with live attenuated viruses is the least viable option. The replication of competent viral vectors may pose a similar threat in relation to the disease spread in immunosuppressed individuals. Vectors with poor replication, however, avoid this risk by maintaining the advantages of the presentation of native antigens, increased immunity of T cells and the ability to express multiple antigens[15]. Subunit vaccines generally require the use of adjuvants and, although DNA and RNA vaccines can offer manufacturing advantages, they are often precariously immunogenic and require multiple doses, which is highly undesirable in the context of a pandemic.

Chimpanzee adenovirus vaccine vectors have been safely administered to thousands of people targeting a wide range of infectious diseases. ChAdOx1 vectorized vaccines were administered to more than 320 volunteers without safety concerns and were shown to be highly immunogenic with the administration of single doses. Relevant information refers to recent clinical trials where a single dose of a vectorized ChAdOx1 vaccine expressing the total spike protein of another beta-choronavirus (MERS-CoV) has been shown to induce neutralizing antibodies.

The use of an active vaccine as a comparator for the control group will minimize the chances of accidentally unblinding the participant, reducing the bias in the analysis of reactogenicity, in the safety report and/or changes in the search for health services once that were symptomatic for COVID-19.

Groups 1c and 1d have been added following interim immunogenicity results on homologous prime-boost groups (as part of the COV001 UK study – see investigator's brochure for further details) showing improved neutralizing antibody titers after 2 doses when compared to 1 dose regimen. A saline placebo will be used in place of an active comparator in group 1d. As we have seen less reactogenicity when giving the booster dose in the UK studies, there is less risk of unblinding the participant by using placebo as a comparator for the booster dose.

Late/annual booster vaccination

An urgent public health question related to COVID-19 vaccination that remains unmet is whether seasonal booster vaccinations may be necessary to enhance immune responses and strengthen protection against the SARS-CoV-2 virus. However, the impact of an annual vaccination with the same adenoviral vector on antigen responses remains uncertain. There are concerns that repeated doses of adenovirus vector vaccines may generate anti-vector immunity, which could compromise the vaccine response, although we have previously shown that ChAdOx1 anti-vector antibodies do not affect anti-spike or anti-spike antibody responses, the specific T cell responses against the S-protein (Spike) and also that individuals who received a previous vaccine with the ChAdOx1 vector for a non-spike transgene at least one year prior to receiving ChAdOx1 nCoV-19 have similar S-protein binding antibody responses (Spike) compared to ChAdOx1 naive individuals.

The addition of a third late dose of ChAdox1 nCoV-19 in subgroups of study participants who have already received two doses will allow the assessment of the immunogenicity and safety of a booster dose.

Fourth dose

A homologous third dose vaccine was shown to be effective in mounting a significant humoral immune response, with acceptable reactogenicity in the UK[20]. However, the emergence of new SARS-CoV-2 virus variants and the reported decrease in antibody titers in vaccinated subjects are of concern.

Because of the rapid decline of neutralizing activity and reduction in vaccine effectiveness against Omicron variant even in individuals who have received 3 doses of vaccine, regulatory authorities (i.e., Israel, Germany, Brazil[21]) now recommend a 4th dose especially for risk groups. The results of an Israeli effectiveness study suggest that a homologous fourth dose of mRNA vaccines could increase protection against severe illness relative to three doses that have been administered over four months ago[22,23]. Therefore, considering the current pandemic scenario and the risk of new variants surge, assessing the immunogenicity and safety of a 4th dose in individuals, who have already received three doses of ChAdOx1 nCOV-19, can underpin public health decisions and policy making.

4 OBJECTIVES AND ENDPOINTS

	MAIN STUDY AND 3RD DOSE OBJECTIVES	
	**Objective**	**Endpoint Measure**
**Primary Objective**	To evaluate the efficacy of ChAdOx1 nCoV-19 vaccine against COVID-19 disease confirmed with PCR.	a) COVID-19 virologically confirmed symptomatic cases (PCR positive).
**Secondary Objectives**	To evaluate the safety, tolerability, and reactogenicity profile of ChAdOx1 nCoV-19 candidate vaccine.	a) Occurrence of signs and symptoms of local and systemic reactogenicity requested during 7 days after vaccination (in a subset of 200 participants*);<br>b) Occurrence of serious adverse events;<br>c) Occurrence of disease enhancement episodes.
	To evaluate the efficacy of ChAdOx1 nCoV-19 candidate vaccine against severe and non-severe COVID-19 disease.	a) Hospitalization for COVID-19 disease confirmed by PCR;<br>b) COVID-19 severe disease confirmed by PCR;<br>c) Death associated with COVID-19 disease;<br>d) Antibodies against SARS-CoV-2 non-Spike protein (efficacy against non-spike seroconversion rates).
	To assess the humoral immunogenicity of ChAdOx1 nCoV-19 candidate vaccine.	a) Antibodies against SARS-CoV-2 spike protein (sero-conversion rates).<br>b) Virus neutralizing antibodies (NAb) against live and/or pseudotyped SARS-CoV-2 virus.

	To assess the cellular immunogenicity of ChAdOx1 nCoV-19 candidate vaccine.**	a) Interferon-gamma (IFN-γ) enzyme-linked immunospot (ELISpot) responses to SARS-CoV-2 spike protein;
	To assess immunological correlates of protection in relation to occurrence of COVID-19 disease in ChAdOx1 nCoV-19 recipients	Immunological endpoints and COVID-19 disease endpoints in ChAdOx1 nCoV-19 recipients
	To evaluate the reactogenicity profile of the third dose of ChAdOx1 nCoV-19 candidate vaccine.	Occurrence of signs and symptoms of local and systemic reactogenicity requested during 7 days after vaccination.
	To determine if the neutralising antibody response to a third dose ChAdOx1 nCoV-19 is non-inferior to the response to the ChAdOx1 nCoV-19 primary series	GMTR of nAb response to the ancestral strain 28 days after a 3rd dose administration vs 28 days after primary series
	To determine if the humoral immune response to a 3rd dose ChAdOx1 nCoV-19 is non-inferior to the response to the ChAdOx1 nCoV-19 primary series	GMTR of Spike binding antibody response to the ancestral strain 28 days after 3rd dose administration vs 28 days after primary series
**4th DOSE*** OBJECTIVES**		
	**Objective**	**Endpoint Measure**
**Primary Objective**	To determine if the neutralising antibody response to a 4th dose ChAdOx1 nCoV-19 is non-inferior to the response to the first ChAdOx1 nCoV-19 booster dose in a subgroup of study participants	GMTR of nAb response to the ancestral strain 28 days after 4th dose administration vs 28 days after a 3rd dose

**Secondary Objectives**	To determine if the humoral immune response to a 4th dose ChAdOx1 nCoV-19 is non-inferior to the response to the first ChAdOx1 nCoV-19 dose in a subgroup of study participants	a) GMTR of nAb response to a contemporary variant of concern 28 days after 4th dose administration vs 28 days after a 3rd dose<br>b) GMTR of Spike binding antibody response to the ancestral strain 28 days after 4th dose administration vs 28 days after a 3rd dose
	To determine if the humoral immune response to a 4th dose ChAdOx1 nCoV-19 is non-inferior to the response to primary series ChAdOx1 nCoV-19 in a subgroup of study participants	a) GMTR of nAb response to the ancestral strain 28 days after 4th dose administration vs 28 days after primary series ChAdOx1 nCoV-19<br>b) GMTR of nAb response to a contemporary variant of concern 28 days after 4th dose administration vs 28 days after primary series ChAdOx1 nCoV-19<br>c) GMTR of Spike binding antibody response to the ancestral strain, variant of concern 28 days after 4th dose administration vs 28 days after primary series ChAdOx1 nCoV-19
	To characterise the cellular immune responses of a 4th dose ChAdOx1 nCoV-19 in seronegative and seropositive participants who previously received an initial ChAdOx1 nCoV-19 boost in a subgroup of participants****	a) Cellular immune responses by ICS (Th1/Th2) over time<br>b) Cellular immune responses by ELISpot over time
	To evaluate the reactogenicity profile of the fourth dose of ChAdOx1 nCoV-19 vaccine.	a) Occurrence of signs and symptoms of local and systemic reactogenicity requested during 7 days after vaccination (in a subset of 100 participants).

		b) Recording of unsolicited AEs for 28 days post 4th dose vaccine<br>c) recording of serious adverse events (SAEs) and adverse events of special interest (AESIs) for duration of the study
**Exploratory objectives**	To explore anti-vector responses to the ChAdOx-1 vector of a 4th dose ChAdOx1 nCoV-19 in seronegative and seropositive participants who previously received an initial ChAdOx1 nCoV-19 boost	a) Geometric mean titres of ChAdOx1 neutralising antibody titres over time<br>b) Pairwise correlations between anti-S, pseudo-neutralisation, and live neutralisation antibody titres over time
	To further evaluate humoral and cellular immunogenicity across all doses through exploratory immunology	Exploratory immunology such as systems serology profiling

*Detailed assessments of local and systemic reactogenicity for 7 days after vaccination with ChAdOx1 nCoV-19 compared to MenACWY as a control have been documented in a sufficient number of participants in previous studies. In study COV003, detailed local and systemic reactogenicity will be evaluated in 200 randomized participants, who received two doses, a quantity statistically determined to ensure proportionality and comparative representativeness compared to studies COV001 and COV002. Local and systemic reactogenicity will be evaluated for 7 days in a subset of 100 participants after a third or fourth dose of ChAdOx1 nCoV-19.

** Cellular immune responses will be measured in a subset of individuals only (up to 60 volunteers who will be recruited from the CRIE-UNIFESP site, sequentially)

*** The fourth dose will be administered to a subset of 350 participants who have previously received 3 doses of ChAdOx1 nCoV-19 vaccine *without prior confirmed COVID-19 infections*.

**** Cellular immune responses will be measured in a subset of individuals only (up to 40 volunteers)

5 STUDY DESIGN

This is a phase III, controlled, randomized, single-blind study to be conducted in adults with high exposure to COVID-19, who are administered two-doses of ChAdOx1 nCoV-19 or MenACWY and saline placebo by means of an IM injection with co-administered paracetamol.

After reviewing all available data from animal studies and UK studies (COV001 and COV002), participants will be randomized to ChAdOx1 nCoV-19 or MenACWY vaccine in a 1:1 ratio in blocks of 4, and all participants will be blinded to the allocation of the vaccine groups. The DSMB will periodically evaluate safety and efficacy data, every 4-8 weeks and/or as needed. The DSMB will consist of the members of the DSMB currently convened who oversee trials in the UK.

Following the immunogenicity results of the UK phase I/II study which showed higher levels of neutralizing antibodies with a prime-boost schedule (https://doi.org/10.1016/S0140-6736(20)31604-4), a booster dose of vaccine will be offered to all participants in the study.

Participants enrolled on version 4.0 of the protocol onwards will only be allowed to take part in the study if they agree to receive 2 doses of either ChAdOx1 nCoV-19 or MenACWY/saline placebo.

Participants who already received a dose of either ChAdOx1 nCoV-19 or MenACWY (before approvals for the second dose were in place) will be offered a booster dose 4-12 weeks after the prime dose of either ChAdOx1 nCoV-19 or saline placebo, depending on which arm they were originally allocated to. Any volunteers enrolled prior to the booster dose protocol amendment will be able to refuse a second dose and will continue their follow-up as per their previously agreed schedule of attendances.

Participants will be followed for the duration of the study to record adverse events and episodes of symptomatic COVID-19 confirmed by PCR. Participants will be assessed for COVID-19 if they have a new fever (≥ 37.8 °C) OR cough OR shortness of breath OR anosmia/ageusia.

Moderate and severe COVID-19 disease will be defined by clinical criteria. Detailed clinical parameters will be collected from medical records and aligned with the definitions of moderate and severe disease agreed by the international scientific community, such as a score greater than 6 on the NEWS-2 scale, or a score of 4 and above on ISARIC/WHO (International Severe Acute Respiratory and Emerging Infection Consortium; WHO Working Group on Clinical Characterization and Management of COVID-19). Such parameters include,

but are not limited to oxygen saturation, need for oxygen therapy, respiratory rate, and other vital signs, need for ventilatory support, radiographic and computed tomography, and blood test results, among other clinically relevant parameters. Considering that the NEWS-2 scale is not used in the clinical routine of the research site, all staff involved in conducting the project will receive specific training.

From protocol 12.1, by October 31, 2021, a third late dose of ChAdOx1 nCov-19 will be offered to a subgroup of participants who previously received two doses of ChAdOx1 nCov-19 with an interval of 4 to 12 weeks between doses, 11-13 months after the second dose. Receiving the third dose will be optional.

From protocol 13.0, an optional fourth dose of ChAdOx1 nCov-19 will be offered to a subgroup of 350 (+/- 10%) participants who previously received three doses of ChAdOx1 nCov-19 within the trial, randomly selected, and regardless of interval after the third dose. These participants must also have collected study visits blood samples within study timeframes. For these participants collection of details of COVID-19 disease will be limited to the extent required to determine a score on ISARIC/WHO scale (section 7.4.5) or to meet safety reporting requirements.

NEWS-2 scoring system for serious COVID-19 assessment:

				Score			
**Physiological parameter**	3	2	1	0	1	2	3
**Breathing rate (per minute)**	≤8		9–11	12–20		21–24	≥25
**SpO2 Scale 1 (%)**	≤91	92–93	94–95	≥96			
**SpO2 Scale 2 (%) - use in hypercapnic respiratory failure**	≤83	84–85	86–87	88–92<br>≥93 in the air	93–94 in oxygen	95–96 in oxygen	≥97 in oxygen
**Air or oxygen?**		Oxygen		Air			
**Systolic blood pressure (mmHg)**	≤90	91–100	101–110	111–219			≥220
**Pulse (per minute)**	≤40		41–50	51–90	91–110	111–130	≥131
**Consciousness**				Alert			CVPU
**Temperature (°C)**	≤35.0		35.1–36.0	36.1–38.0	38.1–39.0	≥39.1	

Patient State	Descriptor	Score
Uninfected	Uninfected; no viral RNA detected	0
Ambulatory mild disease	Asymptomatic; viral RNA detected	1
	Symptomatic; independent	2
	Symptomatic; assistance needed	3
Hospitalised: moderate disease	Hospitalised; no oxygen therapy*	4
	Hospitalised; oxygen by mask or nasal prongs	5
Hospitalised: severe diseases	Hospitalised; oxygen by NIV or high flow	6
	Intubation and mechanical ventilation, $pO_2/FiO_2 \geq 150$ or $SpO_2/FiO_2 \geq 200$	7
	Mechanical ventilation $pO_2/FIO_2 < 150$ ($SpO_2/FiO_2 < 200$) or vasopressors	8
	Mechanical ventilation $pO_2/FiO_2 < 150$ and vasopressors, dialysis, or ECMO	9
Dead	Dead	10

WHO clinical progression scale

ECMO=extracorporeal membrane oxygenation. FiO2=fraction of inspired oxygen. NIV=non-invasive ventilation. pO2=partial pressure of oxygen. SpO2=oxygen saturation. *If hospitalised for isolation only, record status as for ambulatory patient.

5.1 Study groups

Vaccine	Number of Participants	Participants
1a) Single dose of ChAdOx1nCOV19 vaccine, $5x10^{10}$ vp + paracetamol	N = up to 1600 participants	Health professionals and adults with high known likely exposure to COVID-19.
1b) Single dose of MenACWY + paracetamol	N = up to 1600 participants	Health professionals and adults with high known likely exposure to COVID-19.
1c) Two doses of ChAdOx1 nCoV-19 vaccine, $5x10^{10}$vp (prime) and 0.5mL boost	N= up to 5150 (up to 1600 invited from 1a to receive a booster dose	Participants recruited in group 1a will be invited to receive a booster dose, 4-12 weeks apart) and new

(3.5 – 6.5 × 10^{10} vp), 4-12 weeks apart + paracetamol	and new volunteers recruited)	participants recruited on version 4.0 of the protocol onwards will consent to receive a 2-dose schedule.
1d) MenACWY prime, and Saline Placebo boost (0.5mL)), 4-12 weeks apart + paracetamol.	N= up to 5150 (up to 1600 invited from 1b to receive a booster dose and new volunteers recruited)	Participants recruited in group 1b will be invited to receive a booster dose, 4-12 weeks apart) and new participants recruited on version 4.0 of the protocol onwards will consent to receive a 2-dose schedule.
1e A third late dose of ChAdOx1 nCov-19 vaccine, 11-13 months after the second dose, + paracetamol.	N= up to 5150 participants (+1%)	Group 1c participants, who received two doses of ChAdOx1 nCov-19 vaccine with an interval of 4 to 12 weeks, 11-13 months after the second dose, by October 31, 2021.
2) A fourth dose of ChAdOx1 nCov-19 vaccine after a late 3rd dose of ChAdOx1 nCov-19 vaccine.	N = 350 (+/- 10%)	Group 1e participants, who have received three doses of ChAdOx1 nCoV-19 with no confirmed COVID-19 diagnosis, and that have collected study visits blood samples within study timeframes (as per protocol).

The overall sample size will be up to **10,300** (with a margin of 1%) participants. All volunteers previously enrolled in groups 1a and 1b will be offered a booster dose. Any new participants recruited into the study on version 4.0 of the protocol onwards will necessarily have to consent to a 2-dose schedule. From Protocol 12.1, all participants of group 1c will be re-consented to the late third dose, by October 31, 2021.

5.2 Study participants

Adult participants over the age of 18 will be enrolled in the study. Participants will be considered enrolled immediately after the vaccine is administered. Recruitment will focus on healthcare professionals and those with likely high known exposure to COVID-19. For example, they are health professionals: students, residents and professionals who perform health care activities such as nurses and nursing technicians, pharmacists, doctors,

physiotherapists, speech therapists and radiology technicians. High exposure adults will be considered: cleaning and hygiene personnel; safety; reception and concierge; volunteers; drivers, among others. Participants in older age groups (56-69 years and 70 years and above) will be recruited at the investigators' discretion. Their likelihood of COVID-19 exposure will be judged on a case-by-case basis, regardless of their previous occupation.

5.3 Definition of the End of Study

The end of study is the date of the last test performed on the last sample collected.

5.4 Potential risks for participants

Potential risks are those associated with phlebotomy, vaccination, and disease potentiation.

Venepuncture

Hematomas and discomfort located at the venepuncture site may occur. More rarely, fainting may occur. These will not be documented as AE if they occur. The total volume of blood drawn during the study period will be approximately 330 mL, excluding any necessary repeated extraction (blood volumes may vary slightly between participants due to the use of different volume vacutainers, the operational procedures of the research sites and the number of symptomatic visits). Participants will be asked to refrain from donating blood during the period of their involvement in the study.

Allergic reactions

Mild to severe allergic reactions can occur in response to any component of a drug preparation. Anaphylaxis is extremely rare (about 1 in 1,000,000 doses of vaccine), but it can occur in response to any vaccine or medication.

Vaccination

Based on pooled clinical data from studies with ChAdOx1 nCoV-19, the most commonly expected local solicited AEs for participants in this study are vaccination site pain and tenderness. The most commonly reported local, solicited AEs were vaccination site pain and tenderness. The most commonly reported systemic solicited AEs were fatigue, headache, malaise, and myalgia. The majority of reported events have been mild or moderate in severity and resolved within 1 to 7 days. When compared with the first dose, adverse reactions reported after the second dose were milder and reported less frequently.

Post-authorisation hypersensitivity reactions, including anaphylaxis and angioedema, have occurred following administration of ChAdOx1 nCoV-19 and are considered an identified risk.

Important identified risk: A very rare and serious combination of thrombosis and thrombocytopenia, including TTS, in some cases accompanied by bleeding, has been observed following vaccination with ChAdOx1 nCoV-19 during post-authorisation use. This includes cases presenting as venous thrombosis, including unusual sites such as cerebral venous sinus thrombosis, splanchnic vein thrombosis, as well as arterial thrombosis, concomitant with thrombocytopenia. The majority of the events occurred within the first 21 days following vaccination and some events had a fatal outcome. The reporting rates after the second dose are lower compared to the first dose. No events have been observed in the clinical trials. A causal relationship between these events and ChAdOx1 nCoV-19 is considered plausible. The exact mechanism and/or pathophysiology of thrombosis in combination with thrombocytopenia following immunisation is unknown. Although specific risk factors for thromboembolism in combination with thrombocytopenia have not been identified, cases have occurred in patients with a previous history of thrombosis, as well as in patients with autoimmune disorders, including immune thrombocytopenia. The benefits and risks of vaccination should be considered in these patients.

Important potential risks:

Neurologic events and potential immune-mediated neurologic conditions: demyelinating diseases

Vaccine-associated enhanced disease including vaccine-associated enhanced respiratory disease

Cerebrovascular venous and sinus thrombosis without thrombocytopenia

Disease Enhancement

The risks of inducing disease enhancement and pulmonary immunopathology in case of COVID-19 disease after vaccination with ChAdOx1 nCoV-19 are unknown. Challenge studies on ferrets and PNH are ongoing and the results will be reviewed as they arise. Two studies on PNH so far have shown no evidence of disease potentiation until day 7 after the challenge. All preclinical data from challenge studies using ChAdOx1 nCoV-19 and other candidate vaccines (where available) will guide risk/benefit decisions for participants who receive the IMP. Any safety signs associated with the enhancement of the disease potentially observed in COV001/COV002 will also guide these decisions.

5.5 Known potential benefits

Participants enrolled in the control groups will receive MenACWY, a licensed vaccine that has been administered to adolescents on routine vaccination schedules in several countries, including the United Kingdom, and is used as a travel vaccine for high-risk areas. Most of the

participants in this study will not have received this vaccine before and therefore will gain the benefit of protection against meningococci in groups A, C, W and Y. Participants who had previously been vaccinated with MenACWY will have their immunity enhanced against these organisms and are not exposed to additional risks when receiving the additional dose of the comparator vaccine.

If the effectiveness of the vaccine against COVID-19 is proven, after the analysis of the effectiveness results and approval by the Data and Safety Monitoring Committee, the sponsor will make this vaccine available to the research participants who received the control vaccine, MenACWY.

4th dose potential benefits

Participants receiving the 4th vaccination dose might benefit from the potential benefit of added protection.

In addition, study participants will be informed about antibody (IgG) levels against SARS-CoV-2 after the 3rd dose and after the 4th booster dose (as applicable). This is an expectation of the Brazilian population as part of their participation in such studies.

6 RECRUITMENT AND WITHDRAWAL OF STUDY PARTICIPANTS

6.1 Identification of Participants

Participants can be recruited through advertisements approved by the local Ethics Committee. The leaflets will be distributed, including the name of the study, information from the centers, age group, disease, and vaccine.

6.2 Informed Consent Form

The participant will sign and date personally or electronically the last approved version of the Informed Consent Form. When the process is carried out in person, it will be presented to the participants, individually, a written version and verbal explanation of the Informed Consent Form. When the process is carried out electronically, participants will individually receive a link to access the electronic version of the Informed Consent Form. In both cases, it will be detailed:

- the exact nature of the study;
- what it will imply for the participant;
- the implications and restrictions of the protocol;
- the known side effects and any risks involved in participating;

- sample manipulation - participants will be informed about the samples that will be collected anonymously during the course of the study and that can be shared with the study collaborators;
- that individual results will not be shared with participants.

The Informed Consent Form will be made available to the participant before obtaining consent. However, participants will have the opportunity to individually question a properly trained and delegated researcher before signing the consent.

The following general principles will be emphasized:

- Participation in the study is completely voluntary;
- Refusal to participate does not involve a penalty or loss of medical benefits;
- The participant can withdraw from the study at any time;
- The participant is free to ask questions at any time to understand the purpose of the study and the procedures involved;
- The study involves researching an investigational vaccine;
- There is no direct benefit expected for the participant;
- The general practitioner/personal physician of the participant can be contacted to corroborate his/her medical history. Written or verbal information about the participant’s medical history can be requested from the general practitioner/personal physician or other sources;
- Blood samples taken as part of the study can be sent abroad, to the United Kingdom, to laboratories at the University of Oxford. These will not be identified. Participants will be asked whether they agree to biorepository storage for future use, but this will be optional.

The participant will have as much time as he/she wishes to consider the information and the opportunity to question the Investigator, his/her clinical physician or other independent parties to decide whether to participate in the study. The electronic Informed Consent Form must be signed and dated electronically by the adult participant, and the printed Informed Consent Form must be signed and dated by the adult participant and by the person responsible for obtaining the informed consent. This person must be suitably qualified and experienced, have been authorized to do so by the Lead/Principal Investigator and be listed on the delegation´s record. In the case of the printed Informed Consent Form, a copy of the signed document will be given to the participant. The signed original document will be retained at the research study sites.

6.3 Inclusion and exclusion criteria

This study will be performed in healthy adults, who meet the following inclusion and exclusion criteria:

6.3.1 Inclusion Criteria

The participant must meet all the following criteria to be eligible for the study:

- Adults from 18 to 55 years of age.
- Adults aged 56-69 years old (after review of safety data by DSMB in this age group in the UK trial)
- Adults aged 70 and above years old (after review of safety data by DSMB in this age group in the UK trial)
- Able and willing (in the Investigator's opinion) to fulfil all study requirements;
- Health professionals and/or adults at high risk of exposure to SARS-CoV-2, as defined in section 5.2 of this protocol;
- Serology with SARS-CoV-2 negative IgG antibodies; This inclusion criteria does not apply to participants enrolled from version 4.0 of the protocol onwards.
- Willing to allow investigators to discuss the participant’s clinical history with their GP/personal physician and access medical records relevant to the study procedures;
- Only for women of childbearing age: willing to practice continuous effective birth control (see below) during the study, and a negative pregnancy test on the screening and vaccination day(s);
- Consent to abstain from blood donation during the course of the study;
- Provide informed consent in writing.

Additionally for group 2

- Participants that received their third dose ChAdOx1 nCoV-19 in the trial
- Study visits blood samples for visits to date must have been collected within visit windows

The DSMB reviewed safety data from volunteers aged 56 and above recruited as part of the COV002 UK study before recruitment of older adults was allowed.

6.3.2 Exclusion Criteria

The participant will not be eligible for the study if any of the following criteria apply:

- Participation in trials of prophylactic drugs for COVID-19 during the course of the study;

  Note: Participation in COVID-19 treatment trials is permitted in case of hospitalization due to COVID-19, after confirmation of positive PCR. The study team should be informed as soon as possible. Participants with COVID-19 not hospitalized with positive PCR results for COVID-19 may be medicated according to standard clinical practice, however, participation in treatment trials will not be allowed.

- Planned receipt of any vaccine (authorized or investigational), within 30 days before and after vaccination with the exception of the licensed seasonal influenza vaccination and the licensed pneumococcal vaccination. Participants will be encouraged to receive these vaccinations at least 14 days before or after their study vaccine;
- Prior receipt of an investigational or licensed vaccine with the possibility of impacting the interpretation of the study data (for example, Adenovirus vector vaccines, any vaccines against coronavirus);
- Administration of immunoglobulins and/or any blood products in the three months prior to the planned administration of the candidate vaccine;
- Any confirmed or suspected immunosuppressive or immunodeficiency state, including HIV (regardless of treatment, CD4 count or viral load status); asplenia; severe recurrent infections and chronic use (more than 14 days) of immunosuppressive medication in the last 6 months, except for topical steroids or short-term oral steroids (cycle lasting ≤14 days);
- History of allergic disease or reactions possibly exacerbated by any component of ChAdOx1 nCoV-19 or MenACWY or paracetamol;
- Any history of angioedema;
- Any history of anaphylaxis;
- Pregnancy, lactation or willingness/intention to become pregnant during the study;
- Current diagnosis or treatment for cancer (except basal cell carcinoma of the skin and cervical carcinoma in situ);
- History of severe psychiatric illness that possibly affects your participation in the study;
- Hemorrhagic disorder (for example, factor deficiency, coagulopathy or platelet disorder), or a previous history of significant bleeding or bruising after IM injections or venipuncture;

- Current suspected or known dependence on alcohol or drugs;
- Severe and/or uncontrolled cardiovascular diseases, respiratory diseases, gastrointestinal diseases, liver disease, kidney disease, endocrine disorder, and neurological disease (mild/moderate well-controlled comorbidities are allowed);
- History of COVID-19 confirmed by laboratory (serology, rapid tests based on antigen or antibody or PCR);
- Seropositive for antibodies to SARS-CoV-2 before recruitment; This exclusion criteria does not apply to participants enrolled from version 4.0 of the protocol onwards
- Continued use of anticoagulants, such as coumarins and related anticoagulants (for example, warfarin) or new oral anticoagulants (for example, apixaban, rivaroxaban, dabigatran and edoxaban);
- Any other significant illness, disorder or finding that may significantly increase the risk for the participant, affect his/her ability to participate in the study or impair the interpretation of the study data.

6.3.3 Re-vaccination exclusion criteria (two-dose groups only)

The following AEs associated with any vaccine or identified on or before the day of vaccination constitute absolute contraindications to further administration of an IMP to the volunteer in question. If any of these events occur during the study, the subject will not be eligible to receive a booster dose and will be followed up by the clinical team or their regular doctor until resolution or stabilization of the event:

- Anaphylactic reaction following administration of vaccine
- Pregnancy – if the outcome of pregnancy is termination or miscarriage, volunteers can be boosted if appropriate to do so and given they have a negative pregnancy test at the time of boosting.
- Any AE that in the opinion of the Investigator may affect the safety of the participant or the interpretation of the study results
- Participants who develop COVID-19 symptoms and have a positive PCR test after the first vaccination can only receive a booster dose after a minimum 4 weeks interval from their first PCR positive test, provided their symptoms have significantly improved. The decision to proceed with booster vaccinations in those cases will be at clinical discretion of the investigators. For participants who are asymptomatic and have a positive PCR test, a minimum of 2 weeks from first PCR positivity will be required before boosting

6.3.4 Exclusion Criteria for the third dose

The following will not be eligible to receive a late third dose:

- experienced, at any time, infection with SARS CoV2 with a positive PCR test;
- those who received a COVID-19 vaccine other than ChAdOx1 nCoV-19.

6.3.5 Exclusion Criteria for the fourth dose

The following will not be eligible to receive a ChAdOx1 nCoV-19 fourth dose:

- Self reported confirmed COVID-19 infection, through PCR or lateral flow test
- those who received additional COVID-19 vaccines outside of the trial.
- Pregnancy
- Allergy or other contraindication to vaccination with ChAdOx1 nCoV-19
- History of Guillain-Barré syndrome
- Any confirmed or suspected immunosuppressive or immunodeficient state, including Asplenia
- History of clinically significant thrombocytopenia and/or thrombosis or clinically significant bleeding (eg, factor deficiency, coagulopathy, or platelet disorder), or prior history of significant bleeding or bruising following intramuscular injections or venepuncture
- Major venous and/or arterial thrombosis in combination with thrombocytopenia following vaccination with any Covid-19 vaccine
- Severe and/or uncontrolled cardiovascular disease, respiratory disease, gastrointestinal disease, liver disease, renal disease, endocrine disorder, and neurological illness, as judged by the Investigator (mild/ moderate well-controlled comorbidities are allowed
- Plans to move outside the study area

6.3.6 Effective birth control for volunteers

Participants of childbearing potential must use an effective form of birth control during the 12 months of study, or extended for one more month for those who choose to receive the third or fourth dose.

Acceptable forms of birth control for participants include:

- Established use of oral, injected or implanted hormonal of birth control methods;
- Placement of an intrauterine device (IUD) or intrauterine system (IUS);
- Complete hysterectomy;

- Bilateral occlusion of the tubes;
- Contraceptive barrier methods (condom or occlusive tampon with spermicide);
- Male sterilization, if the vasectomized partner is the participant's only sexual partner;
- True abstinence, when it is in line with the subject's preferred and usual lifestyle (periodic abstinence and withdrawal are not acceptable of birth control methods).

6.3.7 Withdrawal of participants

In accordance with the principles of the current version of the Declaration of Helsinki and any other applicable regulations, the participant has the right to withdraw from the study at any time and for any reason and is not required to give his/her reasons for doing so. The Investigator may withdraw the participant at any time for the sake of his/her health and well-being. In addition, the participant can withdraw/be withdrawn for any of the following reasons:

- Investigator's Decision;
- Ineligibility (both during the study and retrospectively, having been omitted in the screening);
- Significant deviation from the protocol;
- Non-adherence of the participant to the study requirements;
- An AE, which requires discontinuing participation in the study or resulting in an inability to continue to comply with the study procedures.

The reason for the withdrawal will be registered with the CRF. If the withdrawal is a consequence of an AE, appropriate follow-up visits and/or medical care will be scheduled, with the consent of the participant, until the AE is resolved, stabilized or a causality unrelated to his/her participation in the study has been attributed. Any participant who is withdrawn from the study may be replaced, if this is possible within the specified period. The DSMB or DSMB president may recommend withdrawing participants.

If a participant withdraws from the study, the data collected before their withdrawal will still be used in the analysis. The storage of blood samples will continue unless the participant specifically requests otherwise.

In all cases of withdrawal from the subject, the collection of long-term safety data, including some procedures such as a safety blood test, will continue as appropriate if individuals have received one or more doses of the vaccine, unless they refuse any additional follow-up

6.4 Pregnancy

If a participant becomes pregnant during the trial, she will be followed up for clinical safety assessment with her continuous consent and, in addition, she will be followed up until the outcome of the pregnancy is determined. We will not routinely perform venipuncture on a pregnant participant unless there is a clinical need. In addition, full and free follow-up and assistance will be ensured, for as long as necessary for: (a) the participants who become pregnant, and (b) the fetus, if applicable.

7 CLINICAL PROCEDURES

This section describes the clinical procedures for evaluating study participants and following up after administering the study vaccine.

7.1 Visit Schedule

All participants will have visits and clinical procedures as indicated in the visit schedule below table 4. The subjects will receive the ChAdOx1 nCoV-19 or MenACWY vaccine/saline solution placebo and will be followed up for a total of 12 months post second dose. Additional visits or procedures may be performed at the investigators' discretion, for example, medical history and additional physical examination, or additional blood tests, if they are clinically relevant.

All participants in groups 1a and 1b will be offered a booster dose of vaccine, however if a participant declines the booster dose, they will continue in group 1a or 1b and follow the planned visit schedule, as per table 4.

From Protocol 12.1, a third late dose of ChAdOx1 nCoV-19 vaccine will be offered to group 1c participants, 11-13 months after receiving the second dose. Those, who choose to receive the third dose, will constitute group 1e. They will be followed up with one more visit, with a total up to 13 months of follow-up after the second dose.

From protocol version 13.0 an optional fourth dose of ChAdOx1 nCoV-19 vaccine will be offered to up to 350 (+/- 10%) participants who have already received 3 doses of ChAdOx1 nCoV-19 in the trial, randomly selected. These participants will have further visits at days 28 and 180 post vaccine, and a telephone call on Day 7.

7.2 Observations, medical history and physical examination

Temperature will be routinely measured at the time-points indicated in the schedule of procedures. Respiratory rate, oxygen saturation, pulse, blood pressure and temperature will be measured at the COVID-19 testing visits and if clinically required. All subjects will undergo medical history and a targeted physical examination if considered necessary at screening or

pre-enrolment on D0. The purpose of this examination is to assess and document the subject's baseline health status so that any later change can be determined. Vital signs (temperature, heart rate, respiratory rate, blood pressure +/- oxygen saturation), height and weight will be measured at screening or pre-enrolment on D0 as part of baseline assessments. Further medical history, physical examination and observations may be done throughout the study based on clinical discretion.

4th dose participants:

Medical history for 4th dose participants will be updated to capture any changes not captured in the original medical history/AEs.

7.3 Blood samples, nose/throat swabs and urine analysis

- **PCR process for COVID-19.** A nose and throat swab will be collected for COVID-19 PCR.
- **SARS-CoV-2 viral genome sequencing.** Participant swab samples that are determined to be SARS-CoV-2 PCR positive may also subsequently undergo further analysis to determine SARS-CoV-2 viral genomes using RNA sequencing. Selection of swab samples for sequencing and the proportion of samples to be sequenced will be at Investigator's and or sponsor's discretion.
- **Immunology.** Immunogenicity will be assessed using a variety of immunological assays. This may include antibodies to SARS-CoV-Spike (ancestral and variant strains) and non-Spike antigens by binding assays, ex vivo ELISpot assays for interferon gamma and flow cytometry assays, neutralization (pseudoneutralisation and live neutralisation) and other functional antibody assays and B cell analyses. Other exploratory immunological assays, including cytokine analysis and other antibody assays, among others, may be performed at the Investigators' discretion. Immunology samples for the assays described above will be drawn as per the schedule of attendances below (table 4)
- **Urine analysis.** In the case of participants of childbearing age, urine will be tested for beta-human chorionic gonadotropin (β-HCG) at screening (when applicable) and immediately before vaccination.

Collaboration with other specialized laboratories in the UK, Europe and outside of Europe may take place for new exploratory tests and for some of the immunology testing described above. This would involve transferring serum, or plasma, PBMC and/or other study samples to these laboratories, but these would remain anonymous. For this, after evaluation and prior approval of the REC/CONEP system, the participant will be presented with a new Informed

Consent Form. Only after obtaining this new consent form can the samples be used for purposes other than those specified in this protocol.

Immunological and sequencing tests will be performed according to the standard operating procedures of the research sites, the University of Oxford, and collaborating international laboratories.

Subjects will be informed that their blood sample (after all tests for this study are completed), will be stored in a biorepository for future use. The subjects will be able to decide whether to allow such future use of any sample. With the informed consent of the participants, blood serum and/or PBMCs will be frozen for future analysis of COVID-19 and responses related to the vaccine. If a subject chooses not to allow this, no sample will be stored beyond the storage period required to meet Good Laboratory Practices (GLP) and regulatory requirements.

7.4 Study visits

Study visits and procedures will be performed by the research sites staff. The procedures to be included in each visit are documented in the visit schedule (table 4). Each visit is assigned a time and window period, within which the visit will take place.

7.4.1 Screening visit and recruitment

All potential participants will have a screening visit, up to 7 days before vaccination for a baseline assessment. For participants who are recruited in the study before version 4.0 of the study protocol, a serological evaluation of baseline antibodies against SARS-CoV-2 is performed. The results of the serology should be available within this period, no later than 7 days after collection. Volunteers with negative serology for IgG antibodies to SARS-CoV-2 may participate in the study (applicable to previous protocol versions only and not from version 4.0 onwards).

Having established that there is a low baseline seropositivity in the study population, the remaining participants can be included without baseline SARS-CoV-2 antibodies. This allows the screening visit to take place at the same day as the vaccination visit and will precede vaccination procedure.

At the screening visit, the objectives of the study and all tests to be performed will be described to the participants. Individually, each participant will have the opportunity to question a duly trained and delegated researcher before signing the consent. The informed consent procedure will be performed before screening/recruitment procedures, as described in section 6.2. A medical history, including previous vaccinations, and targeted physical examination (when necessary) will be conducted at the screening visit. Findings will be recorded as part of baseline

and eligibility assessment. A screening visit can be repeated if time from screening to vaccination is greater than the pre-specified window in the study protocol.

The research site staff may contact the subject's general practitioner/personal physician with written permission after screening to corroborate the clinical history when possible and practical to do so. The participant's doctor may be notified that the subject has been enrolled voluntarily in the study.

7.4.2 Day 0: Recruitment and vaccination visit

Participants will be considered enrolled in the trial at the time of vaccination. Before the investigational vaccination/treatment, the eligibility of the participant will be reviewed. The temperature and, if necessary, a medical history and physical examination will be performed to determine the need to postpone vaccination. The study vaccines/treatment will be administered as described below.

7.4.2.1 Vaccination

All vaccines will be administered intramuscularly according to specific standard operating procedures. The injection site will be covered with a sterile dressing and the participant will remain at the study site for observation, in case of immediate adverse events. Observations will be made at a minimum of 15 minutes after vaccination, the sterile dressing will be removed and the injection site inspected.

A sub sample of 200 participants will receive a thermometer, a metric ruler, access to the electronic diary via web, a printed diary (for use in case the electronic diary presents problems), guidelines and instructions for use, together with a contact card containing the number of 24-hour emergency telephone number to contact the research site if necessary. Participants will be instructed on how to self-assess the intensity and severity of requested adverse events (Table 3 - Requested and unsolicited AEs). There will also be space in the electronic (or paper) Symptom Diary for the participant to document unsolicited AEs for 28 days, and if a medication has been taken to relieve the symptoms. The diaries will collect information about the timing and severity of the following solicited AEs. Participants who were asked to fill out a diary post prime vaccination will be asked to fill out the same diary post booster vaccination.

For a subset of 100 participants, they will be asked to fill in a 7-day diary after the third dose, preferably for those who have already filled it out previously.

For those receiving the 4th dose, a subset of 100 participants will be asked to fill in a 7-day diary after the vaccination. Where possible these will be the same participants that have completed a diary after the third dose.

Table 3. Spontaneous, requested AEs collected in the post-vaccination electronic diary (or daily card) for reactogenicity

AE spontaneous locations	AE systemic spontaneous
Pain	Fever
Sensitivity	Feeling feverish
Redness	Chills
Heat	Joint pain
Itching	Muscle ache
Swelling	Fatigue
Local hardening	Headache
	Malaise
	Nausea
	Vomiting

The use of electronic diaries allows the real-time monitoring of the safety of the study vaccines, however paper back up can be used if electronic diaries are not available.

7.4.3 Later visits

Follow-up visits will take place according to the visit schedule described in table 4, with their respective windows.

If the participants experience adverse events (laboratory or clinical), for which the investigator (doctor) and/or DSMB president requires more rigorous observation, the participant may be admitted to the hospital for observation and subsequent medical treatment under the care of the hospitalization team.

Table 4 Visiting schedule for participants

Groups 1a and 1b

Visit number	Screenin g	1	2	3	4	5	Test for COVID-19 (S0)	(Phone follow up only) COVID-19 PCR positive + 7 days (S7)	Unblinding:<br>• Can be done as a separate event, face to face and online;<br>• Can be done at the site, followed by Control Group vaccination;	Booster dose for controls post unblinding
Chronology** (days)	-7	0	28	90	182	364	As required	7 days after positive PCR for COVID-19	Vaccine availability/ Volunteers visit schedule/ Local approvals	4-12 weeks post prime dose
Time window (days)	±7		-7/+14	±7	±14	±30	N/A	±2	N/A	N/A
Informed consent/Unblinding Consent	X								X	
Review of contraindications, inclusion and exclusion criteria		X								
Vaccination		X							X (subjects who received the control vaccine or subjects who received a single dose of ChAdOx1 nCoV-19, if they agree to a booster dose at least 4 weeks from prime)	X

Vital signs	X	X	X	X	X	X	(X)		X (Temperature only)	X (Temperature only)
Ascertainment of adverse events		X	X	X	X	X	X	X	X	X
COVID Hospital Admission Medical Records Review and Data Collection							If COVID-19 case results in Hospital Admission			
Electronic Diaries of Vaccine Symptoms [$] (subset of volunteers only)		X	X							
Clinical history, physical examination		(X)	(X)	(X)	(X)	(X)	(X)	(X)	(X)	(X)
Biochemistry, hematology (mL)							(5)			
Exploratory immunology (mL)		Up to 50mL	Up to 50mL	Up to 50mL	Up to 50mL	Up to 50mL	10		Up to 50mL (controls receiving ChAdOx1 nCoV-19 only)	Up to 50mL (controls receiving ChAdOx1 nCoV-19 only)
Nose and Throat Swab							X			
urinary bHCG (women only)	X	X							X	X
Blood volume per visit		50	50	50	50	10	15			
Accumulated blood volume [%]		50	100	150	200	250	265	265	315	365

(X) = if deemed necessary ^ = Vital signs at screening or pre-enrolment assessment on D0 include pulse, blood pressure, temperature, respiratory rate +/- oxygen saturation. Only temperature will be routinely measured at subsequent follow-up visits. At COVID-19 testing visits a full set of observations will only be taken if deemed clinically necessary ** Chronology is approximate only. The exact times of the visits refer to the day of recruitment, that is, each visit must take place within the indicated interval of days after recruitment ± time window. % Accumulated blood volume for participants if blood is drawn according to schedule and excluding any repeat safety blood tests that may be required. [$] Vaccine reactogenicity diaries are applicable to a subset of participants only.

NB Participants who refuse a booster vaccination should be followed-up as per the schedule of attendances above.

Group 1c and 1d

Attendance Number (boost)	Screening	1 (prime)	2 (booster)	3	4	5	6	COVID-19 Testing (S0)	(Phone follow up only) COVD-19 Positive PCR + 7 days (S7)	Unblinding<br>• Can be done as a separate event, face to face and online;<br>Can be done at the site, followed by Control Group vaccination;	Booster dose for controls post unblinding
Timeline** (days)	-7	0	4-12 weeks post prime	28 post booster	90 post booster	182 post booster	364 post booster	As required	7 days post COVID-19 PCR positive	Vaccine availability/ Volunteers visit schedule/ Local approvals	4-12 weeks post prime following the national immunization plan
Time window (days)	±7		+14	+7	±14	±14	±30	N/A	±2	N/A	
Informed Consent/Unblinding Consent	X		X[b]							X	
Review contraindications, inclusion and exclusion criteria		X	X								
Vaccination		X	X							X (subjects who received the control vaccine or subjects who received a single dose of ChAdOx1 nCoV-19, if they agree to a booster dose at least 4 weeks from prime)	X

Attendance Number (boost)	Screening	1 (prime)	2 (booster)	3	4	5	6	COVID-19 Testing (S0)	(Phone follow up only) COVD-19 Positive PCR + 7 days (S7)	Unblinding<br>• Can be done as a separate event, face to face and online;<br>Can be done at the site, followed by Control Group vaccination;	Booster dose for controls post unblinding
Vital signs^	X	X	(X)	(X)	(X)	(X)	(X)	(X)		X (Temperature only)	X (Temperature only)
Ascertainment of adverse events		X	X	X	X	X	X	X	X	X	X
COVID Hospital Admission Medical Records Review and Data Collection								If COVID-19 case results in Hospital Admission			
Electronic Diaries of Vaccine Symptoms $<br>(subset of volunteers only)		X	X								
Medical History / Physical Examination		(X)	(X)	(X)	(X)	(X)	(X)	(X)	(X)		
Biochemistry, Haematology (mL)								(5)			
Exploratory immunology (mL)		Up to 50mL	Up to 50mL	up to 50	up to 50	up to 50	up to 50	up to 10		Up to 50mL (controls receiving ChAdOx1 nCoV-19 only)	Up to 50mL (controls receiving ChAdOx1 nCoV-19 only)

Attendance Number (boost)	Screening	1 (prime)	2 (booster)	3	4	5	6	COVID-19 Testing (S0)	(Phone follow up only) COVD-19 Positive PCR + 7 days (S7)	Unblinding<br>• Can be done as a separate event, face to face and online;<br>Can be done at the site, followed by Control Group vaccination;	Booster dose for controls post unblinding
Nose and Throat Swab								X			
Urinary bHCG (women of childbearing potential only)	X	X	X							X	X
Blood volume per visit		50	50	50	50	50	50	up to 15		Up to 50	Up to 50
Cumulative blood volume (post boost)[%]		50	100	150	200	250	300	315	315	365	415

(X) = if deemed necessary ^ = Vital signs at screening or pre-enrolment assessment on D0 include pulse, blood pressure, temperature, respiratory rate +/- oxygen saturation. Only temperature will be routinely measured at subsequent follow-up visits. At COVID-19 testing visits a full set of observations will only be taken if deemed clinically necessary** Chronology is approximate only. The exact times of the visits refer to the day of recruitment, that is, each visit must take place within the indicated interval of days after recruitment ± time window. % Accumulated blood volume for participants if blood is drawn according to schedule and excluding any repeat safety blood tests that may be required. [$] Vaccine reactogenicity diaries are applicable to a subset of participants only [b] New PIS/ICF only for participants enrolled before protocol 4.0 from groups 1c and 1d

NB Participants who accept to take part in booster dose subgroup will have their schedule of attendances replaced by above schedule. Participants who already attended their D28 visit post prime will be asked to attend a separate visit for their booster.

Group 1e

Attendance Number (boost)	Screening	1 (prime)	2 (booster)	3	4	5	6	7	COVID-19 Testing (W0)	(Phone follow up only) COVID-19 Positive PCR + 7 days (W7)	Unblinding<br>• Can be done as a separate event, face to face and online;<br>Can be done at the site, followed by Control Group vaccination;	Booster dose for controls post unblinding
Timeline** (days)	-7	0	4-12 weeks post prime	28 post booster	90 post booster	182 post booster	364 post booster/third dose - booster	28 after third dose	As required	7 days post COVID-19 PCR positive	Vaccine availability/ Volunteers visit schedule/Local approvals	4-12 weeks post prime following the national immunization plan
Time window (days)	±7		+14	+7	±14	±14	±30	+7	N/A	±2	N/A	
Informed Consent/Unblinding Consent	X		X[b]				X[c]				X	
Review contraindications, inclusion and exclusion criteria		X	X				X					
Vaccination		X	X				X				X (subjects who received the control vaccine or subjects who received a single dose of ChAdOx1 nCoV-19, if they agree to a booster	X

Attendance Number (boost)	Screening	1 (prime)	2 (booster)	3	4	5	6	7	COVID-19 Testing (W0)	(Phone follow up only) COVID-19 Positive PCR + 7 days (W7)	Unblinding •Can be done as a separate event, face to face and online; Can be done at the site, followed by Control Group vaccination;	Booster dose for controls post unblinding
											dose at least 4 weeks from prime)	
Vital signs^	X	X	(X)	(X)	(X)	(X)	(X)	(X)	(X)		X (Temperature only)	X (Temperature only)
Ascertainment of adverse events		X	X	X	X	X	X	X	X	X	X	X
COVID Hospital Admission Medical Records Review and Data Collection									If COVID-19 case results in Hospital Admission			
Electronic Diaries of Vaccine Symptoms $ (subset of volunteers only)		X	X				X	X				
Medical History/Physical Examination		(X)	(X)	(X)	(X)	(X)	(X)	(X)	(X)	(X)		
Biochemistry, Haematology (mL)									(5)			
Exploratory immunology (mL)		Up to 50 mL	Up to 50 mL	Up to 50	Up to 50	Up to 50	Up to 50	Up to 50	Up to 10		Up to 50mL (controls receiving	Up to 50mL (controls receiving

Attendance Number (boost)	Screening	1 (prime)	2 (booster)	3	4	5	6	7	COVID-19 Testing (W0)	(Phone follow up only) COVID-19 Positive PCR + 7 days (W7)	Unblinding • Can be done as a separate event, face to face and online; Can be done at the site, followed by Control Group vaccination;	Booster dose for controls post unblinding
											ChAdOx1 nCoV-19 only)	ChAdOx1 nCoV-19 only)
Nose and Throat Swab									X			
Urinary bHCG (women of childbearing potential only)	X	X	X				X				X	X
Blood volume per visit		50	50	50	50	50	50	50	Up to 15		Up to 50	Up to 50
Cumulative blood volume (post boost)%		50	100	150	200	250	300	350	345	345	365	415

(X) = if deemed necessary ^ = Vital signs at screening or pre-enrolment assessment on D0 include pulse, blood pressure, temperature, respiratory rate +/- oxygen saturation. Only temperature will be routinely measured at subsequent follow-up visits. At COVID-19 testing visits, a full set of observations will only be taken if deemed clinically necessary** Chronology is approximate only. The exact times of the visits refer to the day of recruitment, that is, each visit must take place within the indicated interval of days after recruitment ± time window. % Accumulated blood volume for participants if blood is drawn according to the schedule, excluding any repeat safety blood tests that may be required. $ Vaccine reactogenicity diaries are applicable to a subset of participants only b New ICF only for participants enrolled before protocol version 4.0 from groups 1c and 1d. c New ICF for all participants with inclusion of the third dose.

Note: Participants who accept to take part in booster dose subgroup will have their schedule of attendances replaced with the above schedule. Participants who already attended their D28 visit post prime will be asked to attend a separate visit for their booster.

Group 2

Attendance Number (boost)	8<br>4th dose vaccination	Phone call/email contact	9	10
Timeline<br>(days)	At final study visit or at extra visit	7 days post 4th dose	28 days post 4th dose	180 days post 4th dose
Time window (days)		+3	+ 14	+/- 28
Informed Consent	X[b]			
Review contraindications, inclusion and exclusion criteria	X			
Vaccination	X			
Vital signs^	(X)			
Ascertainment of adverse events*		X	X	X
Details of any COVID-19 diagnosis	X	X	X	X
Electronic Diaries of Vaccine Symptoms $<br>(subset of volunteers only)	X	X		
Medical History/Physical Examination	(X)	(X)	(X)	(X)
Exploratory immunology (mL)	Up to 50 mL		Up to 50 mL	Up to 50 mL
IgG against SARS-CoV-2	X		X	X
Urinary bHCG (women of childbearing potential only)	X			
Blood volume per visit	Up to 50mls		Up to 50mls	Up to 50mls
Cumulative blood volume (post 4th dose)%	400		450	500

(X) = if deemed necessary ^ = Vital signs: only temperature will be routinely measured at Visit 08. Chronology is approximate only. The exact times of the visits is relative to the date of administration of 4th dose, that is, each visit must take place within the indicated interval of days after 4th dose ± time window. % Accumulated blood volume for participants if blood is drawn according to the schedule, excluding any safety blood tests that may be required. $ Vaccine reactogenicity diaries are applicable to a subset of participants only [b] New ICF for all participants with inclusion of the fourth dose. * Unsolicited AEs (that may not meet SAE or AESI criteria) will be collected by the investigator during Visit 9 Day 28 post 4th dose. SAE and Adverse Events of Special Interest will be collected throughout the study period.

7.4.4 Symptomatic participants from groups 1a, 1b, 1c, 1d and 1e

Participants who become symptomatic during follow-up will be instructed to call the study team, who will then advise on how to proceed with clinical trials for COVID-19, if necessary, according to the trial work instructions. If a participant is symptomatic, COVID-19 testing should take place from enrolment onwards, regardless of time elapsed from vaccination to symptom onset. An isolated fever ≥37.8^{0}C is an indication for COVID-19 testing, unless this isolated fever has occurred within 48 hours of vaccination. If fever persists beyond 48 hours post-vaccination, the patient will then be eligible for COVID-19 testing. Participants will receive weekly reminders (for example, text messages - SMS or Whatsapp, surveillance APPs notifications, email or telephone contacts) to contact the study team if they experience fever or cough or shortness of breath or anosmia/ageusia and if they are hospitalized for any reason.

7.4.4.1 Diagnostic SARS-CoV-2 PCR testing outside of the trial

In a (primary endpoint symptom) symptomatic illness episode, if a participant has already received a negative SARS-CoV-2 test result via a validated PCR assay that was performed after symptom onset and where this test was carried out outside of the study, they will now (as of protocol V11) be considered to be a negative case without requiring further PCR testing by the study team. In such instances, documentation relating to the result should be acquired by investigators and filed as part of the individual participant record. Additionally, if investigators believe a symptomatic participant can obtain their initial diagnostic SARS-CoV-2 PCR test more rapidly outside of the study (for logistical or other reasons) they may advise participants be tested via that route e.g. by being advised to arrange a test through a local testing clinic or similar service. Where symptomatic participants report a SARS-CoV-2 test outside of the trial but are unable to provide details of the assay and/or documentation of the test result, they will be asked to attend the trial site for a COVID-19 testing visit.

7.4.4.2 COVID-19 testing visit

Participants will be invited for a COVID-19 testing visit in two circumstances:

- They report primary endpoint symptoms AND have not already had a documented negative SARS-CoV-2 PCR test result (including tests performed outside of the trial) since their symptom onset

- They report primary endpoint symptoms and have already had a positive SARS-CoV-2 PCR from outside of the trial since symptom onset

During the test visit for COVID-19, examination with nose/throat swab and immunology bloods (serum and others) will be acquired. Further clinical assessments may be performed at COVID-19 testing visits at the discretion of investigators. Symptomatic participants can be regularly monitored by telephone, if appropriate. Participants who test positive for COVID-19 will continue to be followed throughout the duration of the trial, including repeated COVID-19 testing visits if symptomatic again during the course of the study and until the end of the trial. New episodes will be considered if they have a minimum 28 days interval between the previous PCR positive result. Participants who have a positive PCR at S0 will be reviewed by phone for symptom severity data at approximately 7 days post positive swab. Closer follow-up and safety monitoring may be carried out by local trial teams if felt this is clinically indicated. Symptomatic participants with a positive PCR test outside of the trial will be asked to attend for a COVID-19 testing visit in order to take a further swab to facilitate SARS-CoV-2 sequencing and additionally to acquire immunology bloods.

7.4.4.3 COVID-19 Testing plus 7 day phone call

For symptomatic participants with a positive SARS-CoV-2 PCR test, a remote follow up (via phone or other appropriate means) will take place at approximately 7 days (see schedule of procedure tables for window, section 7.3) from the date of their first positive PCR test in that illness episode. The purpose of this remote follow up is to capture further symptom severity data.

7.4.4.4 Positive SARS-CoV-2 PCR test and vaccination

Participants who develop COVID-19 symptoms and have a positive PCR test after the first vaccination can only receive a booster dose after a minimum 4 weeks interval from their PCR positive test, provided their symptoms have significantly improved. The decision to proceed with booster vaccinations in those cases will be at clinical discretion of investigators. Booster vaccinations of participants are allowed to take place beyond the pre-specified 12 weeks window limit if they are required to be delayed due to PCR positivity within the preceding 4 weeks of the scheduled booster dose.

Further details and instructions on the symptomatic pathway can be found on the clinical study plan.

7.4.5 Symptomatic participants from Protocol 13.0 onwards

Study participants receiving the fourth dose of ChAdOx1 nCOV-19 vaccine (group 2) will be asked about COVID-19 history, symptoms or any positive SARS-CoV-2 PCR or lateral flow antigen test results at each study visit.

All SARS-CoV2 infection cases will be rated as per the clinical progression scale proposed by the World Health Organization. The assessment of hospitalized cases (score 4 or higher) will be performed daily until symptom resolution. In non-hospitalized cases, the maximum score and symptom duration will be recorded (score 1-3).

Hospitalization due to COVID-19 will be considered a SAE and reported as described in Section 9.8.

7.4.6 Review of the medical record

With the consent of the participant, the study team will request access to the medical records or send a data collection form to be filled out by the clinical team, in any episodes of medically attended COVID-19. Investigators will aim to collect clinical data from medical records where participants with suspected COVID-19 have been admitted to hospital.

Prior to protocol V 13.0, Relevant data will be collected to verify the efficacy endpoints and disease enhancement. There is no internationally accepted definition of disease enhancement. Severity between groups will be described and compared. In addition, a proportion of serious illness/all illnesses will be constructed for recipients of the candidate and control vaccine. In case the vaccine induces increased disease, this proportion would be higher in the vaccine than in the control group. These probably include, but are not limited to information about ICU admissions, clinical parameters such as oxygen saturation, respiratory rates and vital signs, need for oxygen therapy, need for ventilatory support, blood test results and images, among others.

From Protocol V13.0 Relevant data will be collected to the extent required to support SAE reporting as described in Section 9.8.

7.4.7 Randomization, blinding, and unblinding

Participants will be randomized to investigational vaccine or MenACWY vaccine in a 1:1 allocation ratio, using block randomization of 4 participants. The blinding scheme will be applied to the participants in relation to the arm in which they were allocated. The blinding scheme will not apply to the study team administering the vaccine. Vaccines will be prepared out of the participant's reach and the syringes will be covered with an opaque label that will guarantee the unilateral blinding of the participant.

If a participant's clinical condition requires unblinding, this will be done according to a specific study work instruction and the group allocation will be sent to the attending physician if unblinding is considered relevant and possibly changes treatment clinical.

The unblinding process and vaccination with investigational product is assured to all volunteers (item 8.11) and will be offered as soon as possible. This process will be followed by the vaccination of the control group within two and a half month maximum for the first dose of the vaccine. The unblinding event may happen face to face or electronically/online in order to allow the volunteers to have the option to get another vaccine available in the country.

If the volunteer belongs to the Control Group and did not get the booster dose yet (placebo dose/saline solution) this dose will be replaced by the Chadox, if the volunteer agrees with that.

The booster dose will be offered up to three months post first dose.

Participants will be censored in the analysis of efficacy endpoints at the time of their unblinding and/or vaccination with an approved/licensed SARS-CoV-2 vaccine (whichever happens first).

7.4.8 Unblinded participants who are eligible to receive an approved or licensed SARS-CoV-2 vaccine

If the efficacy of the candidate vaccine is proven, after analysis of the primary endpoint and approval by the DSMB (Data and Safety Monitoring Committee), as established in the study protocol, the sponsor will make the candidate vaccine available to participants in the research allocated to the comparator group (MenACWY vaccine).

Also, In the event that ChAdOx1 nCoV-19 or other SARS-CoV-2 vaccines being approved for emergency use or licensed by the regulatory agency, all the participants will be invited to the unblinding event which can be face to face or online/electronically, in order to make this information available to all the volunteers as soon as possible, allowing them to have the free option to either receive the ChAdOx1 nCoV-19 (if control group) or another vaccine available in the country.

The order for the volunteers booking will follow, as much as possible, as far as it does not delay the unblinding, the priority groups identified by the NIP (National Immunization Program), but all the participants will be booked for the unblinding event.

We estimate to vaccinate the entire study control group in approximately 2 (two) and a half month. Those in the control group will receive two doses of the ChAdOx1 nCoV-19 vaccine if they wish and will have an optional blood sample collected before each vaccination. The interval between doses will be from 4 to 12 weeks. If after unblinded participants in the control group choose to receive an approved vaccine other than ChAdOx1 nCoV-19, and as part of the national roll-out strategy, they will be free to do so. Participants will be asked about the vaccine they received and date of administration which will be recorded along with any other vaccines received in a specific external vaccination CRF.

Follow up visits by both groups (investigational group and control group that will receive ChAdOx1 nCoV-19 vaccine) will continue according to the participants' previous schedule and their data will contribute to continuous safety monitoring and exploratory assessments of immunogenicity.

For those in the control group who chose to receive ChAdOx1 nCoV-19 as part of the study, a second extra appointment for their booster dose will be scheduled 4-12 weeks later, an optional blood sample will be collected before vaccination.

For those who were unblinded and received a single dose of the ChAdOx1 nCov-19 vaccine, a second dose will be offered at the unblinding visit (minimum 4 weeks interval from prime).

The control group participant who chooses to receive the ChAdOx1 nCov-19 vaccine and has not yet received a booster dose, which is a placebo (saline solution), and is at least 4 weeks apart from MenACWY vaccine, this dose will be replaced by the ChAdOx1 nCov-19 vaccine.

All participants that are unblinded should continue to follow their normal schedule of visits. Wherever possible, unblinding visits and vaccination of controls, or second dose of those who received single-dose only may be combined with pre-scheduled study visits.

All participants will receive a vaccination card with details of the vaccines taken during the study and if selected by the volunteer post unblinding, the new doses of the ChAdOx1 nCov-19 vaccine.

All SAEs and AEs will be collected from the point of unblinding onwards. Participants will then follow their normal schedule of attendances.

7.4.9 Eligible participants who will receive a third dose of ChAdOx1 nCoV-19 vaccine

From Protocol 12.1, a third late dose of ChAdOx1 nCoV-19 vaccine will be offered to a subgroup of participants who previously received two doses of Chadox with an interval of 4 to 12 weeks, 11-13 months after the second dose.

This information will be made available to all volunteers of group 1c and receiving the third dose will be optional. Those volunteers who wish to receive the third dose of ChAdOx1 nCoV-19 will be re-consented and will have a blood sample taken immediately before vaccination, and another 28 days after the third dose.

A subset of 100 volunteers will receive a 7-day electronic diary (hard copy back-up) to record requested adverse events. When completed on hard copy, this diary will be delivered to the center at visit after the third dose, 28 days after vaccination.

7.4.10 Eligible participants who will receive a fourth dose of ChAdOx1 nCoV-19 vaccine

From Protocol 13.0, a fourth dose of ChAdOx1 nCoV-19 vaccine will be offered to a randomly selected subgroup who previously received three doses of ChAdOx1 nCov-19 in the trial (group 1e) regardless of interval after the third dose. Up to 350 (+/-10%) volunteers who wish to receive the fourth dose of ChAdOx1 nCoV-19 will be re-enrolled (if they have completed the study) and provide informed consent to receiving a 4th dose of vaccine. Participants will have a blood sample taken immediately before vaccination, another sample taken at 28 days after the fourth dose, and a third blood sample 180 days after the fourth dose of .

A subset of 100 volunteers will receive a 7-day electronic diary (hard copy back-up) to record solicited adverse events.

8 Investigational Medicinal Product and Control

8.1 Description of ChAdOx1 nCoV-19

ChAdOx1 nCoV-19 vaccine consists of the ChAdOx1 deficient replication monkey adenovirus vector, containing the SARS-CoV-2 structural surface glycoprotein antigen.

8.2 Storage

The vaccine manufactured by Advent is stored at -80°C in a safe freezer at the clinics. The vaccine manufactured by Cobra Biologics Ltd is stored at 2-8°C in a secure fridge, at the clinical site. The traceability of the study vaccines will be documented according to the existing standard operating procedure (SOP). Accounting, storage, shipping, and handling of vaccines will be in accordance with relevant SOPs and forms.

8.3 Administration

On the day of vaccination, ChAdOx1 nCoV-19 will be thawed at room temperature and will be administered according to specific assay instructions. The vaccine manufactured by Cobra Biologics is a multi-dose vial which is stored at 2-8 degrees and does not require thawing. If the vaccine is stored outside of 2-8 it must be used within 6 hours. If stored at 2-8°C after the first vial puncture, it can be used within 48 hours. The vaccine will be administered intramuscularly in the deltoid of the non-dominant arm (preferably). All participants will be observed in the unit for a minimum of 15 minutes after vaccination. During the administration of research products, Advanced Life Support medications and resuscitation equipment will be immediately available for the treatment of anaphylaxis. Vaccination will be performed, and IMPs handled according to the relevant SOPs.

8.4 Rationale for selected dose

The dose to be administered in this study was selected based on clinical experience with the adenovirus vector ChAdOx1 expressing different inserts and other similar vectorized adenovirus vaccines (for example, ChAd63).

A first dose escalation study in humans using the ChAdOx1 vector encoding an influenza antigen (FLU004) administered ChAdOx1 NP + M1 safely at doses ranging from 5×10^8 to 5×10^{10} pv. The subsequent review of the data identified an optimal dose of 2.5×10^{10} pv, balancing immunogenicity and reactogenicity. This dose was later administered to hundreds of volunteers in numerous larger phase 1 studies at the Jenner Institute. ChAdOx1 vectorized vaccines have so far shown to be very well tolerated. The vast majority of AEs have been mild to moderate and there have been no SARs to date.

Another monkey adenovirus vector (ChAd63) was safely administered in doses of up to 2×10^{11} pv, with an optimal dose of 5×10^{10} pv, balancing immunogenicity and reactogenicity.

MERS001 was the first clinical trial of a vector ChAdOx1 expressing the total Spike protein from a separate, but related beta-coronavirus. ChAdOx1 MERS has been administered to 31

participants so far in doses ranging from $5x10^9$ pv to $5x10^{10}$ pv. Despite the greater reactogenicity observed with the dose of $5x10^{10}$ pv, this dose was safe, with self-limiting AE and without registered SARs. The dose of $5x10^{10}$ pv was the most immunogenic, in terms of inducing neutralizing antibodies against MERS-CoV using a live virus assay (Folegatti et al. Lancet Infect Dis, 2020, https://doi.org /10.1016/S1473-3099(20)30160-2). Due to the immunology findings and the safety profile observed with a ChAdOx1 vector vaccine against MERS-CoV, the dose of $5x10^{10}$ pv was chosen for ChAdOx1 nCoV-19.

An analytical comparability assessment of ChAdOx1 nCoV-19 (AZD1222) manufactured by CBF, Advent and Cobra Biologics was conducted using a comprehensive set of physiochemical and biological release and characterization tests. In order to support the analytical comparability assessment, A260 testing of Advent's process (K.0007, K.0008, and K.0009 lots) was performed, where corrections to the absorbance due to excess polysorbate 80 were made to compensate for polysorbate 80 concentrations above the formulation target of 0.1% (w/v).

Differences in strength related attributes (ie, virus particle concentration, virus genome concentration, and infectious virus concentration) are noted. These differences in strength is further examined for potential impact on clinical dosing. The target clinical dosage of CBF's product is 5×10^{10} viral particles per dose based on vp/mL concentration determined by UV spectroscopy (A260), whereas that of Advent's product is 5×10^{10} viral genome copies per dose based on vg/mL concentration determined by qPCR. The target clinical dosage of Symbiosis' product is $3.5 – 6.5 \times 10^{10}$ viral particles per dose based on the vp/mL concentration determined by A260, with a 0.5 mL dosing volume. This dosing range is based on a target 5×10^{10} viral particles per dose and a ± 30% range to take into account process and method variabilities. The planned clinical dosage of Symbiosis' product is compared to that of CBF and Advent products, the resulting Symbiosis' product dosage at 0.5 mL for lot 20481A is somewhat lower in total viral particle per dose (20% from the lower range limit), slightly higher in total viral genome copies per dose (12% from the higher range limit), and slightly lower in total infectious particle per dose (8% from the lower range limit). These differences are considered to be comparable to or within the variabilities from the analytical methods used in concentration determination (A260, qPCR, and infectivity) and the dosing volumes during clinical administration. In summary, with a 0.5 mL dosing volume for Symbiosis' product, strength difference from CBF and Advent products is not expected to have significant clinical impact in terms of reactogenicity and immunogenicity/efficacy

Table 12 Clinical Strengths of ChAdOx1 nCoV-19 (AZD1222) Drug Product

Strength Attribute	CBF		Advent			Cobra
	Lot 02P20-01	Lot 02P20-02	Lot K.0007	Lot K.0008	Lot K.0009	Lot 20481A
**Concentration**						
Virus particle concentration (A_{260}) (vp/mL)	1.49×10^{11}	1.22×10^{11}	3.12×10^{11}	3.16×10^{11}	2.45×10^{11}	0.8×10^{11}
Virus genome concentration (qPCR) (vg/mL)	1.7×10^{11}	Not tested	1.7×10^{11}	2.1×10^{11}	1.4×10^{11}	1.3×10^{11}
Infectious particle concentration (ifu/mL) [a]	2.6×10^{9}	Not tested	2.9×10^{9}	3.0×10^{9}	2.4×10^{9}	1.3×10^{9}
**Target Clinical Dosage**						
Equivalent DP volume per dose (mL)	0.34	0.41	0.294	0.235	0.356	0.50
Dosing of virus particle (vp/dose)	5.1×10^{10}	5.0×10^{10}	9.2×10^{10}	7.4×10^{10}	8.7×10^{10}	4.0×10^{10}
Dosing of viral genome (vg/dose)	5.8×10^{10}	NA	5.0×10^{10}	4.9×10^{10}	5.0×10^{10}	6.5×10^{10}
Dosing of infectious particle (ifu/dose)	8.8×10^{8}	NA	8.5×10^{8}	7.1×10^{8}	8.5×10^{8}	6.5×10^{8}

ifu = infectious units; NA = not applicable; vp = virus particle; vg = virus genome

[a] Testing performed using the Advent infectivity assay.

8.5 Environmental contamination control (GMO)

The possibility of environmental contamination with genetically modified organisms (GMOs) will be appropriately controlled. The study will be performed in accordance with the relevant local regulations regarding GMO products, following the recommendations of CTNBio. The approved SOPs will be followed to minimize the spread of the recombinant vector vaccine virus in the environment. GMO residues will be inactivated according to the approved SOPs. All material used during vaccination and by vaccination personnel will be autoclaved and incinerated later.

8.6 Investigational Medicinal Product for the 4th dose study

Recombinant Covid-19 vaccine (AstraZeneca/Fiocruz) containing chimpanzee adenovirus codifying Spike SARS-CoV-2 glycoprotein will be used for the 4th dose study. The standard dose of AstraZeneca/Fiocruz COVID-19 vaccine is $5x10^{10}$vp in 0.5ml. The vaccine should be administered intramuscularly. The AstraZeneca/Fiocruz vaccine is supplied in packs of 10 vials. Each vial contains 8 or 10 doses of vaccine, and is a colourless to slightly yellow, clear to slightly opaque liquid.

The AstraZeneca/Fiocruz vaccine should be stored at +2°C to +8°C and has a shelf life of 6 months. The vaccine does not contain any preservative. After first opening the vial, it should be used within 6 hours when stored at room temperature (up to 30°C) or within 48 hours when stored in a refrigerator (2 to 8°C). After this time, the vial must be discarded. The total cumulative storage time once opened must not exceed 48 hours.

8.7 Control Vaccine

Participants who are allocated to control groups will receive an injection of the MenACWY vaccine instead of ChAdOx1 nCoV-19. Either of the two MenACWY vaccines of authorized quadrivalent protein-polysaccharide conjugate – will be used, i.e.:

- Nimenrix (Pfizer). The authorized dosage of this vaccine for those over 6 months of age is a single intramuscular dose (0.5 mL), containing 5 mcg each of a polysaccharide from group A, C, W and Y of *Neisseria meningitidis*, each conjugated with 44 mcg of tetanus toxoid carrier protein.
- Menveo (Glaxo Smith Kline). The authorized dosage of this vaccine for those aged 2 years or older is a single intramuscular dose (0.5 mL), containing
    - 10 mcg of group A meningococcal polysaccharide, conjugated with 16.7 to 33.3 mcg of CRM $protein_{197}$ of *Corynebacterium diphtheriae*.
    - 5 mcg of group C meningococcal polysaccharide, conjugated with 7.1 to 12.5 mcg of CRM $protein_{197}$ of *Corynebacterium diphtheriae*.
    - 5 mcg of group W meningococcal polysaccharide, conjugated with 3.3 to 8.3 mcg of CRM $protein_{197}$ of *Corynebacterium diphtheriae*.
    - 5 mcg of group Y meningococcal polysaccharide, conjugated with 5.6 to 10.0 mcg of CRM $protein_{197}$ of *Corynebacterium diphtheriae.*

The summary of product characteristics for both vaccines allows the administration of a booster dose, if indicated by ongoing risk. Prior administration of a vaccine (or a quadrivalent simple meningococcal polysaccharide vaccine in groups A, C, W and Y) is not a contraindication to receiving another vaccine in this study.
The masking of the participants regarding the injection they are receiving will be maintained. A vaccination accounting record from MenACWY will be maintained at each study site.

MenACWY will be stored in a locked (or controlled access) refrigerator (2 °C to 8 °C) at the research site, according to the package insert.

8.8 Placebo

Participants who were allocated to the control group will receive a placebo injection of 0.9% saline instead of MenACWY at the time of boosting. The volume and site of injection will be the same as for the intervention arm and participants will be blinded as to which injection they are receiving. A vaccine accountability log of the saline will be maintained at each trial site, similar to what is done for the study intervention (ChAdOx1 nCoV-19) and the comparator used as the prime dose (MenACWY).

8.9 Compliance with the investigational treatment

All vaccines will be administered by the research team and registered with the CRF. The study medication will not be in the participant's possession at any time and, therefore, compliance will not be a problem.

8.10 Investigational treatment accounting

IMP accounting and control vaccines will be performed in accordance with the relevant SOPs.

8.11 Concomitant medication

As established by the exclusion criteria, participants cannot be enrolled in the study if they have received: any vaccine within 30 days prior to enrollment or if any other vaccine is expected to be administered within 30 days after each vaccination, any research product within 30 days prior to recruitment or if administration is planned during the study period, or if there is any chronic use (> 14 days) of any immunosuppressive medication in the 6 months prior to enrollment or if administration is planned at any time during the study period (topical steroids are allowed).

Participants who make continuous use of oral anticoagulants, such as coumarins and related anticoagulants (i.e. warfarin) or new oral anticoagulants (i.e. apixaban, rivaroxaban, dabigatran and edoxaban), and/or who received immunoglobulins and/or any blood products

in the three months prior to the planned administration of the candidate vaccine, will be excluded from this study, according to the exclusion criteria.

Participants will be advised to take paracetamol, unless contraindicated (in which case they will be excluded from the study), for 24 hours after vaccination. Paracetamol will be stored according to the package insert. There will be no additional labeling beyond its authorized packaging.

8.12 Provision of treatment for controls

If the efficacy of the candidate vaccine is proven, after analysis of the primary endpoint and approval by the DSMB (Data and Safety Monitoring Committee), as established in the study protocol, the sponsor will make the candidate vaccine available to participants in the research allocated to the comparator group (MenACWY vaccine).

9 SAFETY ASSESSMENT

Safety will be assessed by the frequency, incidence and nature of the AEs and SAE emerging during the study.

9.1 Definitions

9.1.1 Adverse Event (AE)

An AE is any unexpected medical occurrence in a participant, which can occur during or after the administration of an IMP and does not necessarily have a causal relationship to treatment. An AE can therefore be any unfavorable and unintended sign (including any clinically significant abnormal finding or change from baseline), symptom or disease temporally associated with study treatment, even if it is considered to be related to study treatment or not.

9.1.2 Adverse Reaction (AR)

An AR is any unexpected or unintended response to an IMP. This means that the causal relationship between the IMP and the AE is at least reasonable, that is, the relationship cannot be ruled out. All cases judged by the medical investigator to have a reasonable causal relationship with an IMP (that is, possibly, probably, or definitely related to an IMP) will qualify as AR.

Adverse events that may be related to the IMP are listed in the Investigator’s Brochure for each product, as well as within the product information for the applicable market product.

9.1.3 Serious Adverse Event (SAE)

An SAE is an AE that results in any of the following endpoints, considered or unrelated to the study treatment.

- Death;
- Life-threatening event (i.e., the participant was, in the Investigator's view, at immediate risk of death from the event);
- Persistent disability or disability or significant disability (i.e., substantial disruption of the ability to perform normal life functions);
- Hospitalization or extension of existing hospitalization, regardless of length of stay, even if it is a precautionary measure for continuous observation;
    - Note: Hospitalization (including hospitalization or outpatient hospitalization for an elective procedure) for a pre-existing condition that has not unexpectedly worsened does not constitute a serious AE.
- An important clinical event (which cannot cause death, be life-threatening or require hospitalization) that may, based on appropriate clinical criteria, harm the participant and/or require medical or surgical treatment to avoid one of the outcomes listed above. Examples of such clinical events include an allergic reaction that requires intensive treatment in an emergency room or clinic, blood dyscrasias or seizures that do not result in hospitalization;
- Congenital anomaly or birth defect.

9.1.4 Serious Adverse Reaction (SAR)

A serious AE that, in the opinion of the investigator or sponsor, is believed to be possibly, probably, or definitely related to IMP or any other treatment of the study, based on the information provided.

9.1.5 Suspected Unexpected Serious Adverse Reaction (SUSAR)

A SAR, whose nature and severity are not consistent with the information on the drug in question set out in the IB.

9.2 Expectation

Refer to expected SARs as listed within the product information for the applicable market product.

9.3 Predicted/expected adverse reactions:

Predictable/expected AR after vaccination with ChAdOx1 nCoV-19 include pain at the injection site, tenderness, erythema, heat, swelling, induration, itching, myalgia, arthralgia, headache, fatigue, fever, feverish feeling, chills, malaise, and nausea.

9.4 Adverse Events of Special Interest

AESI relevant to vaccination in general will also be monitored, such as: generalized seizure, Guillain-Barre Syndrome (GBS), Acute Disseminated Encephalomyelitis (ADEM), Thrombocytopenia, Anaphylaxis, Vasculitis, in addition to the requested serious AEs.

Thrombotic, thromboembolic, and neurovascular events will also be considered an AESI.

Additionally, potential immune mediated diseases (pIMDs), which include autoimmune and other inflammatory and/or disorders of interest that may or not have an autoimmune etiology, will be considered an AESI and are listed on Appendix A. However, the investigator will exercise their medical and scientific judgment when deciding whether other diseases have autoimmune origin (i.e., physiopathology involving systemic pathogenic or organ-specific autoantibodies) and should also be recorded as a pIMD.

9.5 Causality

For each AE, an assessment of the relationship between the event and the administration of the vaccine will be performed by the clinician delegated by the IC. An interpretation of the causal relationship of the treatment with the AE in question will be made, based on the type of event; the relationship of the event to the time of vaccine administration; and the known biology of vaccine therapy. Alternative causes of AE will be considered and investigated, such as the natural history of pre-existing medical conditions, concomitant therapy, other risk factors and the temporal relationship of the event to vaccination. The causality assessment will take place during planned safety reviews and in the final safety analysis, except for SAEs, which must be designated immediately by the investigator reporting the events.

0	Not related	No temporal relationship with the product under study ***and*** alternative etiology (clinical, environmental, or other treatments) ***and*** Does not follow known pattern of response to the product under study
1	Unlikely	Unlikely temporal relationship with the product under study ***and*** alternative etiology (clinical, environmental, or other treatments) ***and***

		It does not follow the typical or plausible pattern of response to the product under study.
2	Possible	Reasonable temporal relationship with the product under study; ***or*** Event not produced immediately by clinical, environmental, or other treatments; ***or*** Response pattern similar to that seen with other vaccines
3	Probable	Reasonable temporal relationship with the product under study; ***and*** Event not produced immediately by clinical, environmental, or other treatments ***or*** Known response pattern seen with other vaccines
4	Definitive	Reasonable temporal relationship with the product under study; ***and*** Event not produced immediately by clinical, environmental, or other treatments; ***and*** Known response pattern seen with other vaccines

Table 5. Guidelines for assessing the relationship between vaccine administration and an AE.

9.6 Reporting procedures for all adverse events

All local and systemic AEs after vaccination observed by the Investigator or reported by the participant, whether or not attributed to the study medication, will be recorded by the participants in the Diary of Symptoms and by the Investigators in the study CRF. All AEs that result in the withdrawal of a participant from the study will be followed up until a satisfactory resolution occurs (if the participant agrees to do so), or until a causality unrelated to the study is assigned. SAE and Adverse Events of Special Interest will be collected throughout the study period. All SAEs and AEs will be recorded for participants who are unblinded and receive COVID-19 vaccine as part of the study (ChAdOx1 nCoV-19 for those in the control arm) or any other approved SARS-CoV-2 vaccine given as part of national roll-out strategy

4th dose and reporting procedures for all AEs

All local and systemic AEs after vaccination reported by the participant, whether or not attributed to the study medication, will be recorded by a subset of 100 participants in the Diary of Symptoms for 7 days. Unsolicited AEs (that may not meet SAE or AESI criteria) will be collected by the investigator during Visit 2, at Day 28 post 4th dose. All AEs that result in the withdrawal of a participant from the study will be followed up until a satisfactory resolution occurs (if the participant agrees to do so), or until a causality unrelated to the study is assigned. SAE and Adverse Events of Special Interest will be collected throughout the study period.

9.7 Evaluation of severity

The severity of adverse events will be assessed according to toxicity rating scales adapted from the FDA for healthy volunteers recruited in preventive vaccine clinical trials, listed in the specific study work instructions and tables 6-8 below.

Adverse Event	Grade	Intensity
Sensitivity	1	Mild discomfort to touch
	2	Discomfort with movement
	3	Significant discomfort at rest
	4	Hospitalization or A/E visit
Injection site pain	1	Pain that is easily tolerated
	2	Pain that interferes with daily activity
	3	Pain that impairs daily activity
	4	Hospitalization or A/E visit
Injection site erythema	1	2.5 – 5 cm
	2	5.1 – 10 cm
	3	>10 cm
	4	Exfoliating dermatitis or necrosis
Injection site induration/swelling	1	2.5 – 5 cm and does not interfere with activity
	2	5.1 – 10 cm or interferes with activity
	3	> 10 cm or impairs daily activity
	4	Necrosis

Table 6. Severity criteria for local adverse events

*erythema ≤2.5cm is an expected consequence of skin puncture and therefore will not be considered an adverse event

Vital Signs	Grade 1 (mild)	Grade 2 (moderate)	Grade 3 (serious)	Grade 4 Potentially fatal
Fever (oral)	38, 0°C- 38, 4°C	38.5°C – 38.9°C	39.0°C – 40°C	> 40°C
Tachycardia (bpm)*	101 – 115	116-130	>130	A/E visit or hospitalization for arrhythmia
Bradycardia (bpm)**	50 – 54	45 – 49	<45	A/E visit or hospitalization for arrhythmia
Systolic hypertension (mmHg)	141 -150	151 – 155	≥155	A/E visit or hospitalization for malignant hypertension
Diastolic hypertension (mmHg)	91 – 95	96 – 100	>1100	A/E visit or hospitalization for malignant hypertension
Systolic hypotension (mmHg)***	85 – 89	80 – 84	<80	A/E visit or hospitalization for hypotensive shock
Respiratory Rate – breaths per minute	17 – 20	21-25	>25	Intubation

Table 7. Severity rating criteria for physical observations (applies to adults only).

*Measured after ≥10 minutes at rest ** When the resting heart rate is between 60 to 100 beats per minute. Use the clinical criterion when characterizing bradycardia among some populations of healthy subjects, for example, conditioned athletes. *** Only if symptomatic (for example, dizziness/vertigo)

**GRADE 0**	None
**GRADE 1**	Mild: Transient or mild discomfort (<48 hours); There was no interference with routine activity; no medical treatment/therapy was needed
**GRADE 2**	Moderate: Mild to moderate limitation in routine activity – some assistance may be required; minimal medical treatment/therapy was required or none
**GRADE 3**	Serious: Marked limitation in routine activity, some assistance is usually required; medical treatment/therapy was required.
**GRADE 4**	Potentially fatal: requires assessment in A/E or hospitalization

Table 8. Severity classification criteria for local and systemic AE.

9.8 Serious AE reporting procedures

To comply with the rules in force on reporting SAE to regulatory authorities, the event will be accurately documented, and the notification deadlines respected. Serious adverse events will be reported on the SAE forms to members of the study team as soon as the Investigators become aware of their occurrence. Copies of all reports will be forwarded for review by the Principal Investigator (as Sponsor's representative) within 24 hours after the Investigator becomes aware of the alleged SAE. The DSMB will be notified of SAE that are considered to be possibly, probably or definitely related to the study treatments; the DSMB president will be notified immediately (within 24 hours) as soon as the Sponsor become aware of the occurrence. Normally, SAE will not be reported immediately to the Ethics Committee, unless there is a clinically significant increase in the rate of occurrence, an unexpected endpoint, or a new event that may affect the safety of study participants, at the discretion of the Principal Investigator and/or DSMB. In addition to the expedited report above, the Investigator will include all SAE in the annual Development Safety Update (DSUR) report. In addition, all local reporting requirements apply.

Cases falling under the Hy's Law should be reported as SAEs. A Hy's Law Case is defined by FDA Guidance for Industry "Drug-Induced Liver Injury: Premarketing Clinical Evaluation" (2009). Any study participant with an increase in Aspartate Aminotransferase (AST) or Alanine Aminotransferase (ALT) ≥ 3x Upper Limit of Normal (ULN) together with Total Bilirubin ≥2xULN, where no other reason can be found to explain the combination of these abnormal results, e.g., elevated serum alkaline phosphatase (ALP) indicating cholestasis, viral hepatitis A, B or C, another drug capable of causing the observed injury, amongst others.

9.9 Procedures for reporting SUSARs

All SUSARs will be communicated by the sponsor's delegate to the Competent Authority and REC and other parties, as applicable. For fatal and life-threatening SUSARs, this will be done within 7 calendar days after the Sponsor or delegate becomes aware of the reaction. Any additional relevant information will be reported within 8 calendar days of the initial report. All other SUSARs will be informed within 15 calendar days.

The principal investigators will be informed of all SUSARs for the relevant IMP for all studies with the same Sponsor, even if the event occurred or not in the present study.

9.10 Safety assessments

The safety profile will be continuously assessed by the Investigators. The CI and relevant investigators (according to the study delegation record) will also review safety and SAE issues as they arise.

The DSMB will assess the frequency of events, safety, and efficacy data every 4-8 weeks and/or as needed. The DSMB will make recommendations regarding the conduct, continuation or modification of the study.

The Sponsor may put the study on hold and pause recruitment if SUSARs reported in other international trials within the ChAdOx1 nCoV-19 programme are considered to pose a significant safety concern to all participants in the programme. The DSMB will review such events and will make a recommendation as to whether or not recruitment can continue. Study procedures other than vaccinations (e.g. safety follow-ups, immunogenicity assessments, and COVID-19 testing procedures) will continue as normal, regardless of length of study pause.

9.11 Data Safety Monitoring Committee

The Data Safety Monitoring Committee that is in place for the British studies will also oversee this study and review the safety data for this study.

At least one properly qualified clinician/scientist from each international study site will be invited to attend meetings of the existing trial's DSMB.

The DSMB president can be contacted for independent advice and review by the Researcher or study sponsor in the following situations:

- Follow-up on any SAE considered possibly, probably or definitely related to a study treatment;

- Any other situation in which the Investigator or Study Sponsor feels that independent advice or review is important.

The DSMB will review the SAE considered to be possibly, probably, or definitely related to the study treatments. The DSMB will be notified within 24 hours after the Sponsor become aware of the occurrence. The DSMB can recommend stopping recruitment into the study, if necessary, to follow up on an SAE related to a study treatment. It will also recommend restarting the study, when appropriate, following review of such safety events (i.e. SUSARs associated with ChAdOx1 nCoV-19).

The DSMB will review safety data from volunteers aged 56 and above recruited as part of the COV002 UK study. Recruitment of older adults will only be allowed following advice from the DSMB.

10 STATISTICS

10.1 Description of statistical methods

Both a fully detailed study level statistical analysis plan (SAP) as well as a separate Statistical Analysis Plan for the Marketing Authorization Application (MAA SAP) will be written and signed off before any interim data analyses are conducted. A SAP describing the analyses for 3rd and 4th dose (including non-inferiority assessments) will be separate from the study-specific and pooled MAA SAP. Immunogenicity analyses on 3rd and 4th dose will be conducted after SAP finalization and sign-off.

The data from this study will be included in prospective pooled analyses of studies for efficacy and safety of ChAdOx1 nCoV-19 to provide greater precision of both efficacy and safety outcomes

10.2 Efficacy Outcomes

The primary efficacy endpoint is PCR positive symptomatic COVID-19.

This is defined as a participant with a PCR+ swab and at least one of the following symptoms: cough, fever > 37.8, shortness of breath, anosmia, or ageusia.

Where possible, sensitivity analyses will be conducted using common alternative definitions of virologically-confirmed COVID-19 disease, including those in use in other phase 3 protocols (including but not limited to: USA AstraZeneca phase 3 trial, South Africa COV005 trial, WHO solidarity trial, CEPI definition). This will aid in comparisons between various studies and meta-analyses. These alternative definitions will be detailed in the statistical analysis plan as exploratory analyses.

Due to the vaccine-induced disease mitigation potential, the inclusion of all positive PCR infections as a primary result may lower the vaccine's estimated effectiveness and reduce its accuracy. COVID-19 disease, positive for PCR and symptomatic, is a more specific primary outcome and may lead to an earlier demonstration of vaccine efficacy, although it includes fewer cases.

Regarding the differentiation of ADE and the lack of effectiveness of the vaccine: there is no internationally accepted definition of ADE. Differences in disease severity between groups will be described. If the proportion of serious illness is similar between the two groups, this would support the lack of effectiveness and not the improvement of the illness.

10.2.1 Efficacy

The primary and secondary analysis will be conducted on participants who are seronegative at baseline. A sensitivity analysis will be conducted including all participants regardless of baseline serostatus.

The screening of the participants will be based on the serological exam with IgG antibodies negative for SARs-CoV-2. However, for the analysis of the primary outcome, a validated assay detecting antibodies against SARS-CoV-2 nucleoprotein will be used to exclude any remaining participants who were seropositive at baseline.

Analysis of the primary endpoint will be computed as follows:

1. **Efficacy of two doses of ChAdOx1 nCoV-19**. Participants will be included who received two doses of ChAdOx1 nCoV-19. Events will be included if they occurred more than 14 days after the booster dose.
   Participants who are symptomatic up to 14 days after the second dose of vaccine will be excluded from the analysis. In addition, those with less than 14 days follow up post-second dose will also be excluded.

Secondary analyses of the primary outcome:

2. **Efficacy of at least one dose of vaccine**. Cases occurring more than 21 days after the first vaccination will be included.
   Participants who are symptomatic up to 21 days from vaccination will still attend site for PCR testing and blood samples but will be excluded from the analysis as these participants may have been exposed to SARS-CoV-2 prior to vaccination or before the immune system has had time to mount a response to the vaccine. In addition, those with less than 21 days follow up post-vaccination will also be excluded.

The proportions of participants meeting the primary outcome definition will be compared between groups of recipients of ChAdOx1 nCoV-19 and MenACWY using a Poisson regression

model with robust variance (Zou 2004). The model will contain terms including treatment group, and age group at randomization if there is a sufficient sample size within each age category. The logarithm of the period at risk for primary endpoint will be used as an offset variable in the model to adjust for volunteers having different follow up times during which the events occur. Vaccine efficacy (VE) will be calculated as (1 – RR) x 100%, where RR is the relative risk of symptomatic infection (ChADOx1 nCoV-19: Control) and 95% confidence intervals will be presented.If the Poisson regression model with robust variance fails to converge, the exact conditional method for stratified Poisson regression will be used.

The cumulative incidence of symptomatic infections will be presented using the Kaplan-Meier method.

Secondary efficacy endpoints will be analyzed in the same way as the primary efficacy endpoint.

Analyses will be conducted for all adults combined as well as conducting analyses stratified by age cohorts.

All data from participants with PCR-positive swabs will be assessed for inclusion in the efficacy analyses by two blinded assessors who will independently review each case according to pre-specified criteria as detailed in the statistical analysis plan, to classify each for inclusion in the primary and secondary outcomes. A separate CRF will be designed for this purpose.

All PCR-positive results will be assessed for the primary outcome, including those with symptoms who were swabbed by trial staff and other potential sources of information such as health-care workers who are tested at their workplace as either a routine test procedure or due to developing symptoms.

Exploratory COVID-19 disease endpoints may be assessed by trained endpoint adjudicators within the trial team, following pre-specified criteria, rather than independent blinded assessors.

10.2.2 Safety and Reactogenicity

For each group, the counts and percentages of each local and systemic adverse reaction requested from the daily cards, and all unsolicited AEs and SAEs will be presented.

10.2.3 Immunogenicity

Highly deviated antibody data will be transformed logarithmically before analysis. The geometric mean of the concentration and the associated 95% confidence interval will be summarized for each group at each timepoint, calculating the anti-log of the average difference of the logarithmically transformed data.

The geometric means of concentration on day 28 and the proportion of participants with serum conversion to S-spike protein from day 0 to day 28 will be computed. Comparisons between the ChAdOx1 nCoV-19 vaccine and control groups will be made using a Mann Whitney U test due to the low titers expected in the control group that will cause non-normal distribution.

10.3 3rd and 4th dose studies

The overall hypothesis for the extension to examine 3rd and 4th dose in this study is that 28 days after receiving a 4th dose the response will be non-inferior to the response 28 days after receiving the 3rd dose for immunogenicity (ie, neutralising antibodies geometric mean titre ratio). The specific null and alternative hypotheses for each objective will be presented in a $3^{rd}/4^{th}$ dose SAP.

The immune response 28 days after the 3rd or 4th dose vaccines, will be compared to the immune response 28 days after the previous vaccine (2nd or 3rd dose) using non-inferiority comparisons. The within-person difference between log-transformed responses (dose $4^{+28\ days}$ minus dose $3^{+28\ days}$) will be computed and non-inferiority will be assessed. All non-inferiority comparisons of geometric mean titre ratios will be made utilizing the lower bound of two-sided confidence intervals ($\alpha = 0.05$) with non-inferiority margin 2/3

All non-inferiority comparisons of seroresponse rates will be made utilizing the lower bound of two-sided confidence intervals ($\alpha = 0.05$) with non-inferiority margin 10%.

10.4 Subgroup analyses

Subgroup comparisons of efficacy, and safety will be conducted by incorporating vaccine-group by subgroup interaction terms into appropriate regression models. Subgroup comparisons will only be conducted if there are at least 5 cases in all subgroups.

Comparisons will include:

1. Males vs females
2. Age (18 to 55 years vs 56-<70 years vs 70+ years)
3. Seropositive to S-spike or non-spike proteins at baseline vs not seropositive
4. Health care workers and highly-exposed participants versus others
5. Ethnicity
6. BMI (< 30 / >= 30 kg/m2)

10.5 Number of Participants

The research sites will include up to 10,300 participants (with a margin of 1%).

Fourth dose boost substudy

Data are available from Flaxman et al to inform sample size calculations as follows (https://www.sciencedirect.com/science/article/pii/S0140673621016998)

Table 8 Data available for sample size calculation from Flaxman et al

Assay	Visit	N	Mean (log10)	SD (log10)	Pearson correlation coefficient
Anti-spike IgG (ELISA)	Dose 2 + 28 days	71	3.28169	0.51413	0.33506
	Dose 3 + 28 days	71	3.55010	0.38949	
	**Difference**	**71**	**0.26841**	**0.53089**	

Assuming the standard deviation for the difference between dose 3 and dose 4 is similar to the standard deviation for the difference between dose 2 and dose 3 as seen in Flaxman et al, then 300 participants will provide >99% power to show non-inferiority of the 4th dose compared with the 3rd dose, assuming a non-inferiority margin for the lower bound of the GMFR of 2/3 (-0.176 on $\log_{10}$ scale), and alpha of 0.025. The final numbers for inclusion in the analysis will depend on the proportion who are eligible at the time of vaccination.

Table 9 Power available for different numbers of participants included in the 4th dose sub-study analysis

Power	N	SD	Alpha
>99%	250	0.531	0.025
>99%	300		
>99%	350		

10.6 Interim and primary analyses of the primary outcome

It is planned that the primary evidence of efficacy and safety for the ChADOx1 nCoV-19 vaccine will be based on global analyses utilizing studies COV001 (the UK P1/2 study), COV002 (the UK P2/3 study), COV003 (the Brazil P3 study) and COV005 (the South Africa P1/2 study) including a pooled analysis across the studies. As such the interim and primary analyses for the primary outcome will be based on cases accumulated across multiple studies, details of which will be specified within the MAA SAP rather than for each individual study. Interim and primary data cuts from this study will therefore be carried out to support the pooled analysis. The global MAA SAP allows for interim and primary analyses to be conducted once sufficient eligible cases have accumulated, where the overall type 1 error is controlled at the 5% level using a flexible alpha–spending approach that accounts for the incorporation of data from this study into pooled interim analyses under the global MAA SAP.

Evidence of efficacy will be determined if the lower bound of the multiplicity adjusted confidence interval is greater than a 20% threshold. The primary analysis will have approximately 90% power assuming a vaccine efficacy of 60%. A flexible alpha spending approach will be implemented to allow an earlier primary analysis in the situation where accumulation of eligible cases was lower than expected.

Evidence of efficacy at an interim or primary analysis of pooled data will not be considered a reason to stop the trial, but instead will be interpreted as early evidence of efficacy. However, if an interim analysis demonstrates evidence of efficacy, then a study level analysis according to the study SAP may be used to support study level evidence of efficacy.

10.7 Final Analysis

A final analysis will be conducted at the end of the study. The final study-specific analysis will incorporate all data from the study, including data that has previously contributed to global efficacy estimates under the pooled analysis strategy. The final analysis will be considered a supportive analysis to the global efficacy analysis. Participants will be censored in the analysis at the time of unblinding or their vaccination with an alternative COVID-19 vaccine.

Although the roll-out of COVID19 vaccines will affect vaccine efficacy estimation over time, all participants remain in the studies and continued to be followed-up. Respective immunological assessments will provide important insights regarding duration of protection.

In this context, exploring duration of protection through efficacy endpoints will utilize different statistical approaches. This will include traditional techniques, using all available data with censoring of at the time of unblinding because of the decision to switch to a vaccine. Given the Poisson regression model includes an offset term for follow-up, it has the flexibility to allow for multiple imputation approaches to assess vaccine efficacy using different assumptions around the control attack rate after unblinding. Data collected after unblinding will also allow for a longer-

term descriptive assessment of cumulative incidence of efficacy for participants randomized to the vaccine arm. In addition, more novel techniques for an inferred control arm are also being considered, extending the methods described in Follmann et al 2020.

10.8 Inclusion in the analysis

All vaccinated participants will be included in the analysis, unless otherwise specified

10.9 Data and Safety Monitoring Committee

The independent DSMB will meet regularly to review safety data from all available studies of ChAdOx1 nCoV-19. Additionally, the independent DSMB will make recommendations based on the interim analyses to assess evidence of efficacy. The DSMB works according to the DSMB statute and/or follows the trigger points of the different protocols of the global clinical development plan, allowing the different steps to be achieved with respect to safety.

11 DATA MANAGEMENT

11.1 Data processing

The Principal Investigator will be responsible for all data that accumulates in the study.

All study data, including the participant's diary, will be recorded directly in an Electronic Data Capture (EDC) system (for example, OpenClinica, REDCap or similar) or in a paper source document for later insertion in EDC if the direct entry is not available. This includes safety data, laboratory data and endpoints data. All documents will be stored securely and in confidential conditions.

Participants will be identified by a unique number and code specific to the study in any database. The name and any other identifying details will NOT be included in any electronic study data file.

The EDC system (CRF data) uses a relational database (MySQL/PostgreSQL) through a secure web interface with data checks applied during data entry to ensure data quality. The database includes a full set of features that comply with GCP, EU and UK regulations and Sponsor security policies, including a full audit trail, user-based privileges and integration with the institutional LDAP server. The MySQL and PostgreSQL databases and the web server will be hosted on servers that are kept secure. The backups will be stored according to the IT department's schedule on a daily, weekly, and monthly basis and retained for one month, three months and six months, respectively. IT servers provide a stable, secure, well-maintained, high-capacity data storage environment. RedCap and OpenClinica are widely

used, powerful, reliable and well supported systems. Access to the study database will be restricted to members of the study team with a username and password.

Unblinding: The date of unblinding will be recorded in the database. If the control group receives the vaccine through the study team, the date and details of vaccination will be entered into the database. If the vaccine is received in the national roll out but the national programme, participant may be contacted to provide information or if no response is received the information will be collected at the next study visit.

11.2 Record keeping

Investigators will maintain adequate medical and research records for this trial, in accordance with the GCP and regulatory and institutional requirements to protect the confidentiality of participants. The principal investigator, sub investigators and clinical research nurses will have access to the records. Investigators will allow Sponsor's authorized representatives, as well as ethical and regulatory agencies, to examine (and when required by applicable law, copy) clinical records for the purposes of quality assurance reviews, audits and evaluation of the safety and progress of the study.

11.3 Source data and technical data sheets (CRFs)

All information required by the protocol will be collected in CRFs designed by the Sponsor. All source documents will be archived. The source documents are documents, data and original records from which the participant's CRF data is obtained. For this study, these will include, but are not limited to, the Informed Consent Form, blood test results, and response letters from the general practitioner, laboratory records, diaries, medical records and correspondence. In this study, this will include, but is not limited to, medical history, medication records, vital signs, physical exam records, urine tests, blood test results, adverse event data and vaccine details. All source data and CRFs of the participants will be safely stored.

Where data are recorded directly onto the electronic data system these will be considered source documents. However, if local regulations require these electronic case report forms to be printed, they will be printed and filed in the participants

11.4 Data protection

The study protocol, documentation, data, and all other information generated will be kept strictly confidential. No information about the study or its data will be disclosed to unauthorized third parties without prior written approval from the sponsor.

Identifiable details, such as contact details, will be stored for a minimum of 5 years. Unidentified search data may be stored indefinitely. If participants agree to be contacted for future research, information about their Informed Consent Form will be recorded, retained and stored securely and separately from the research data.

11.5 Data quality

The data collection tools will undergo proper validation to ensure that the data is collected accurately and completely. The datasets provided for analysis will be subject to quality control processes to ensure that the data analyzed is a true reflection of the source data. Study data will be managed in accordance with local data management SOPs. If an additional study of specific processes is necessary, an approved Data Management Plan will be implemented.

11.6 Archiving

Study data can be stored electronically on a secure server, and paper notes will be kept in a filing cabinet locked with a key in the site. All essential documents will be retained for a minimum of 5 years after the end of the study. The need to store study data for a longer time in relation to vaccine authorization will be subject to continuous review. For effective vaccines that can be authorized, we can safely store research data in the sites at least 15 years after the study ends, subject to adjustments to clinical trial regulations. Where participants' relevant bank details will be stored for 7 years, in accordance with the sites's financial policy. Unidentified search data may be stored indefinitely.

12 QUALITY CONTROL PROCEDURES AND QUALITY ASSURANCE

12.1 Investigator’s Procedures

The approved standard operating procedures (SOPs) will be used in the research sites and in all laboratory centers.

12.2Monitoring

Regular monitoring will be performed in accordance with the GCP by the monitor. When proceeding in accordance with the written SOPs, the monitor will verify that the clinical trial is performed and the data are generated, documented and reported in accordance with the protocol, GCP and applicable regulatory requirements. The sites will provide direct access to all data/documents and source reports related to the study for the purpose of monitoring and auditing by the Sponsor and to inspect by local and regulatory authorities.

12.3 Deviation from the protocol

Any deviations from the protocol will be documented on a protocol deviation form and filed in the study’s master file.

Each deviation will be assessed for its impact on the safety of the participants and the conduct of the study. Significant deviations from the protocol will be listed at the end of the study report.

12.4 Audit and inspection

The QA manager conducts internal systems-based audits to verify that trials are being conducted in accordance with local procedures and in accordance with study protocols, departmental SOPs, GCP and applicable regulations.

The Sponsor, the study sites, the Research Ethics Committee, and the Regulatory Agencies may conduct audits to ensure compliance with the appropriate protocol, GCP, and regulations.

13 ETHICAL AND REGULATORY CONSIDERATIONS

13.1 Declaration of Helsinki

Investigators will ensure that this study is being conducted in accordance with the principles of the current revision of the Declaration of Helsinki.

13.2 Guidelines for good clinical practices

The Investigator will ensure that this trial is being conducted in accordance with the relevant standards and good clinical practices.

13.3 Ethical and regulatory approvals

After the Sponsor's approval, the Protocol, the Informed Consent Form, the participant's information sheet and any proposed advertising material will be submitted to an appropriate Research Ethics Committee (REC), regulatory authorities and host institutions for written approval. No changes to this protocol will be made without consulting the Sponsor and without its consent.

The Investigator is responsible for ensuring that changes in an approved study, during the period for which the approval of the Research Ethics Committee and Regulatory Agency has already been given, are not initiated without their review and approval, except to eliminate immediate risks apparent to the subject (i.e., as an urgent safety measure).

13.4Volunteers confidentiality

The study will comply with the EU General Data Protection Regulation (GDPR) and the UK Data Protection Act of 2018, as well as local data protection regulations, which require data not to be identified, whenever and when practical to do so. The processing of participants' personal data will be minimized by using only a single study number of the participant in all study documents and in any electronic database, with the exception of informed consent forms and participant identification records. All documents will be stored securely, and accessible only by study staff and authorized personnel. The study team will protect the privacy of participants' personal data. A separate confidential file containing personally identifiable information will be stored in a secure location in accordance with current data protection legislation. The photographs taken at the vaccination sites (if necessary, with the written and informed consent of the participant) will not include the face of the participant and will be identified by the date, study code and the subject's unique identifier. Once developed, the photographs will be stored as confidential records, as above. This material can be shown to other professionals, used for educational purposes, or included in a scientific publication.

If participants are diagnosed with COVID-19 during the course of the study, the study team will pass on their details to the local health protection team, if necessary, in accordance with the relevant notifiable disease legislation. Samples collected for the purpose of diagnosing COVID-19 can be sent to reference laboratories together with your personal data. This would be in line with national guidance and the policy of sending samples for testing in reference laboratories.

14 FUNDING AND INSURANCE

14.1 Funding

University of Oxford and external donors (Fundação Lemann, Fundacao Brava, Fundacao Telles, Instituto D'or de Ensino e Pesquisa and AstraZeneca Brasil).

14.2 Insurance

Global Insurance

Insured Party: University of Oxford

Research participants who suffer direct damage as a result of their participation in the study are entitled to claim compensation from the sponsor and the institutions involved in this study, covered by global and local insurance for research protocols.

14.3 Publication Policy

Researchers will be involved in reviewing draft manuscripts, abstracts, press releases and any other publications resulting from the study. The study data can also be used as part of a doctoral or master's thesis.

15 DEVELOPMENT OF A NEW PRODUCT/PROCESS OR INTELLECTUAL PROPERTY GENERATION

The IP title generated by University employees belongs to the University. The protection and exploitation of any new IP is managed by the University's technology transfer office, Oxford University Innovations. Researchers in this study can benefit from the University's royalty-sharing policy if new intellectual property is generated from the study. Several investigators are applicants or co-inventors of past patent registrations or patents related to ChAdOx1 vaccines. University of Oxford, which is a partner of Oxford University Hospitals NHS Foundation Trust at the NIHR Oxford Biomedical Research Centre, is committed to translational progress and the commercial development of healthcare products potentially serving medical and global healthcare needs and works and will work with business partners for these purposes.

References

1 Zhu, N. *et al.* A Novel Coronavirus from Patients with Pneumonia in China, 2019. *New England Journal of Medicine* **382**, 727-733, doi:10.1056/NEJMoa2001017 (2020).

2 Lu, R. *et al.* Genomic characterisation and epidemiology of 2019 novel coronavirus: implications for virus origins and receptor binding. *Lancet (London, England)* **395**, 565-574, doi:10.1016/S0140-6736(20)30251-8 (2020).

3 Li, F. Structure, Function, and Evolution of Coronavirus Spike Proteins. *Annual review of virology* **3**, 237-261, doi:10.1146/annurev-virology-110615-042301 (2016).

4 Zhou, P. *et al.* A pneumonia outbreak associated with a new coronavirus of probable bat origin. *Nature*, doi:10.1038/s41586-020-2012-7 (2020).

5 Alharbi, N. K. *et al.* ChAdOx1 and MVA based vaccine candidates against MERS-CoV elicit neutralising antibodies and cellular immune responses in mice. *Vaccine* **35**, 3780-3788, doi:10.1016/j.vaccine.2017.05.032 (2017).

6 Tseng, C. T. *et al.* Immunization with SARS coronavirus vaccines leads to pulmonary immunopathology on challenge with the SARS virus. *PloS one* **7**, e35421, doi:10.1371/journal.pone.0035421 (2012).

7 Weingartl, H. *et al.* Immunization with modified vaccinia virus Ankara-based recombinant vaccine against severe acute respiratory syndrome is associated with enhanced hepatitis in ferrets. *Journal of virology* **78**, 12672-12676, doi:10.1128/jvi.78.22.12672-12676.2004 (2004).

8 Liu, L. *et al.* Anti-spike IgG causes severe acute lung injury by skewing macrophage responses during acute SARS-CoV infection. *JCI insight* **4**, e123158, doi:10.1172/jci.insight.123158 (2019).

9 Agrawal, A. S. *et al.* Immunization with inactivated Middle East Respiratory Syndrome coronavirus vaccine leads to lung immunopathology on challenge with live virus. *Human vaccines & immunotherapeutics* **12**, 2351-2356, doi:10.1080/21645515.2016.1177688 (2016).

10 Munster, V. J. *et al.* Protective efficacy of a novel simian adenovirus vaccine against lethal MERS-CoV challenge in a transgenic human DPP4 mouse model. *NPJ vaccines* **2**, 28, doi:10.1038/s41541-017-0029-1 (2017).

11 Alharbi, N. K. *et al.* Humoral Immunogenicity and Efficacy of a Single Dose of ChAdOx1 MERS Vaccine Candidate in Dromedary Camels. *Scientific reports* **9**, 16292, doi:10.1038/s41598-019-52730-4 (2019).

12 Antrobus, R. D. *et al.* Clinical assessment of a novel recombinant simian adenovirus ChAdOx1 as a vectored vaccine expressing conserved Influenza A antigens. *Mol Ther* **22**, 668-674, doi:10.1038/mt.2013.284 (2014).

13 Coughlan, L. *et al.* Heterologous Two-Dose Vaccination with Simian Adenovirus and Poxvirus Vectors Elicits Long-Lasting Cellular Immunity to Influenza Virus A in Healthy Adults. *EbioMedicine*, doi:https://doi.org/10.1016/j.ebiom.2018.02.011 (2018).

14 Wilkie, M. *et al.* A phase I trial evaluating the safety and immunogenicity of a candidate tuberculosis vaccination regimen, ChAdOx1 85A prime – MVA85A boost in healthy UK adults. *Vaccine* **38**, 779-789, doi:10.1016/j.vaccine.2019.10.102 (2020).

15 Modjarrad, K. MERS-CoV vaccine candidates in development: The current landscape. *Vaccine* **34**, 2982-2987, doi:10.1016/j.vaccine.2016.03.104 (2016).

16 She, J., Liu, L. & Liu, W. COVID-19 epidemic: Disease characteristics in children. *J Med Virol*, doi:10.1002/jmv.25807 (2020).

17 Dong, Y. *et al.* Epidemiology of COVID-19 Among Children in China. *Pediatrics*, doi:10.1542/peds.2020-0702 (2020).

18 Ludvigsson, J. F. Systematic review of COVID-19 in children shows milder cases and a better prognosis than adults. *Acta Paediatr*, doi:10.1111/apa.15270 (2020).

19 Zimmermann, P. & Curtis, N. Coronavirus Infections in Children Including COVID-19: An Overview of the Epidemiology, Clinical Features, Diagnosis, Treatment and Prevention Options in Children. The Pediatric Infectious Disease Journal 39, 355-368, doi:10.1097/inf.0000000000002660 (2020).

20. _ Baxter D, et al. Safety and immunogenicity of seven COVID-19 vaccines as a third dose (booster) following two doses of ChAdOx1 nCov-19 or BNT162b2 in the UK (COV-BOOST): a blinded, multicentre, randomised, controlled, phase 2 trial. The Lancet, 398(10318):2258-2276. doi: https://doi.org/10.1016/S0140-6736(21)02717-3 (2021)

21. Nota técnica Nº 20/2022 -SECOVID/GAB/SECOVID/MS. Recomendação da segunda dose de reforço de vacinas contra Covid-19 em pessoas com 80 anos ou mais. Ministério da saúde; 30/03/2022.

22. Regev-Yochay, G, Gonen, T, Gilboa M, et al. 4th dose COVID m RNA vaccines' immunogenicity & efficacy against omicron VOC. medRix preprint 25 Feb 2022, doi: https://doi.org/10.1101/2022.02.15.22270948.

23.YinonM. BarOn, Yair Goldberg, Micha Mandel, Omri Bodenheimer, Ofra Amir, Laurence Freedma n, Sharon Alroy-Preis, Nachman Ash, Amit Huppert, Ron Milo. Protection by 4th dose of BNT162b2 against Omicron in Israel. medRix preprint 01 Feb 2022, doi: https://doi.org/10.1101/2022.02.01.22270232

APPENDIX A: Adverse Events of Special Interest

Neuroinflammatory disorders	• Cranial nerve neuropathy, including palsy and paresis (eg, Bell's palsy).<br>• Optic neuritis.<br>•Multiple sclerosis.<br>• Transverse myelitis.<br>• Guillain-Barré syndrome, including Miller Fisher syndrome and other variants.<br>• Acute disseminated encephalomyelitis, including site-specific variants, eg: non-infectious encephalitis, encephalomyelitis, myelitis, myeloradiculoneuritis.<br>• Myasthenia gravis, including Lambert-Eaton myasthenic syndrome.<br>• Peripheral demyelinating neuropathies, including:<br>- Chronic inflammatory demyelinating polyneuropathy.<br>- Multifocal motor neuropathy.<br>• Polyneuropathies associated with monoclonal gammopathy.<br>• Narcolepsy.
Musculoskeletal disorders	• Systemic lupus erythematosus and associated conditions.<br>• Systemic scleroderma (systemic sclerosis), including:<br>- Diffuse scleroderma.<br>- Calcinosis, Raynaud's phenomenon, esophageal dysmotility, sclerodactyly and telangiectasia syndrome (CREST).<br>• Idiopathic inflammatory myopathies, including:<br>- Dermatomyositis.<br>- Polymyositis.<br>• Anti-synthetase syndrome.<br>• Rheumatoid arthritis and associated conditions, including:<br>- Juvenile idiopathic arthritis.<br>- Still's disease.<br>• Polymyalgia rheumatica.<br>• Spondyloarthropathies, including:<br>-Ankylosing spondylitis.<br>- Reactive arthritis (Reiter's syndrome).<br>- Undifferentiated spondylarthritis.

	- Psoriatic arthritis.<br>- Enteropathic arthritis.<br>• Recurrent polychondritis.<br>• Disorder of mixed connective tissue.<br>• Gout
Skin disorders	• Psoriasis.<br>• Vitiligo.<br>•Erythema nodosum.<br>• Autoimmune bullous skin diseases (including pemphigus, pemphigoid, and dermatitis herpetiformis).<br>• Lichen planus.<br>• Sweet's syndrome.<br>• Localized scleroderma (morphea).
Vasculitis	• Large vessel vasculitis including:<br>- Giant cell arteritis (temporal arteritis).<br>- Takayasu's arteritis.<br>• Vasculitis of medium and/or small vessels, including:<br>- Polyarteritis nodosa.<br>- Kawasaki disease.<br>- Microscopic polyangiitis.<br>- Wegener's granulomatosis (granulomatosis with polyangiitis).<br>- Churg-Strauss syndrome (allergic granulomatous angiitis or eosinophilic granulomatosis with polyangiitis).<br>- Buerger's disease (thromboangiitis obliterans).<br>- Necrotizing vasculitis (cutaneous or systemic).<br>- Vasculitis positive for antineutrophil cytoplasmic antibody (type unspecified).<br>- Henoch-Schonlein purpura (immunoglobulin A vasculitis).<br>- Behcet's syndrome.<br>- Leukocytoclastic vasculitis.
Blood disorders	• Autoimmune hemolytic anemia.<br>• Autoimmune thrombocytopenia.<br>• Antiphospholipid syndrome.<br>• Pernicious anemia.<br>• Autoimmune aplastic anemia.

	• Autoimmune neutropenia.<br>• Autoimmune pancytopenia.<br>• Anti-platelet antibodies<br>• Vascular thrombosis<br>• Stroke
Liver disorders	• Autoimmune hepatitis.<br>• Primary biliary cirrhosis.<br>• Primary sclerosing cholangitis.<br>• Autoimmune cholangitis.
Gastrointestinal Disorders	• Inflammatory bowel disease, including:<br>-Crohn's disease.<br>- Ulcerative colitis.<br>- Microscopic colitis.<br>- Ulcerative proctitis.<br>•Celiac disease.<br>• Autoimmune pancreatitis.
Endocrine Disorders	• Autoimmune thyroiditis (Hashimoto's thyroiditis).<br>• Grave's or Basedow's Disease.<br>• Type 1 diabetes mellitus.<br>• Addison's Disease.<br>• Polyglandular autoimmune syndrome.<br>• Autoimmune hypophysis.
Others	• Autoimmune glomerulonephritis including:<br>- Immunoglobulin A nephropathy.<br>- Rapidly progressive glomerulonephritis.<br>- Membranous glomerulonephritis.<br>- Membranoproliferative glomerulonephritis.<br>- Mesangioproliferative glomerulonephritis.<br>- Tubulointerstitial nephritis and uveitis syndrome.<br>• Autoimmune eye diseases, including:<br>- Autoimmune uveitis.<br>- Autoimmune retinitis.

	• Autoimmune myocarditis.<br>• Sarcoidosis.<br>• Stevens-Johnson syndrome.<br>• Sjögren's Syndrome.<br>• Alopecia areata.<br>• Idiopathic pulmonary fibrosis.<br>• Goodpasture's syndrome.<br>• Raynaud's phenomenon.

APPENDIX B AMENDMENTS HISTORY

Amendment No.	Version of the protocol No.	Date issued	Amendment author(s)	Details of Amendments made
N/A	1.0	May 27th, 2020	N/A	First version
1	1.0	May 31st, 2020	PPD	Ethical requirements of the Brazilian Ethics Committee/CONEP system
2	2.0	June 10th, 2020	PPD<br>PPD	Ethical requirements of the English Ethics Committee (OxTREC) – updated data according to IB 6.0
3	3.0	June 14th, 2020	PPD, PPD	ANVISA requests, updating of participating centers, electronic ICF, correction of sample size and exclusion of assistance to pregnant partners of research participants.

Amendment No.	Version of the protocol No.	Date issued	Amendment author(s)	Details of Amendments made
4	4.0	July 28th, 2020	PPD	Added booster groups. Removed requirement for negative COVID-19 serology prior to enrollment; Added details on COBRA batch; clarified process around data entry/source data; updated statistical analysis section to reflect changes in trial procedures (e.g. addition of booster doses); added cellular immune responses in a subset of individuals as exploratory objective; added clarifications to swabbing procedures; added Hy's law cases as part of the requirement for SAE reporting; added clarifications to DSMB composition and their role in advising stopping recruitment.
5	4.1	August 11th, 2020	PPD<br>PPD	Clarify boosting dose windows; include placebo as comparator; update abbreviations; clarify the cellular immune response volunteers selection; clarify study groups; clarify non-hospitalized volunteers shall not take part in covid-19 treatment clinical trials; clarify serology criteria

Amendment No.	Version of the protocol No.	Date issued	Amendment author(s)	Details of Amendments made
				before/after protocol 4.0; update table 4
6	5.0	August 16th, 2020	PPD	Increased sample size to up to 10,000; Changes to statistical analysis section, including changes to how the primary endpoint will be analysed; changes to the symptomatic pathway; clarifications to the inclusion and exclusion criteria
7	6.0	September 30th 2020	PPD	Increased time from vial piercing to vaccine administration from 4 to 6 hours; clarifications to inclusion and exclusion criteria and removal of one of the exclusion criteria (participation in serological surveys); clarification and minor changes to symptomatic pathway in line with clinical study plan (requirements on visit S3-5, diaries, COVID-19 hospitalisation data collection); clarifications to vital signs collected at different timepoints; additional funders;
8	7.0	October 29th 2020	PPD	Increase in sample size to up to 10.300 people to account for the competitive and simultaneous

Amendment No.	Version of the protocol No.	Date issued	Amendment author(s)	Details of Amendments made
				recruitment strategy at multiple sites.
9	8.0	12 Nov 2020	PPD	Clarifications to vital signs and physical measurements required at different visits; Updated Statistical Analysis section in line with global programme and with references to study level SAP and a separate SAP for Marketing Authorisation Application<br><br>Increased number of subset of individuals for CMI assessment from 50 to 60.
10	9.0	15 Jan 2021	PPD	Described procedures for study unblinding following country roll out of COVID-18 vaccines .<br><br>Only SAEs and AESI to be recorded following unblinding and vaccination of those in the control arm.<br><br>Increased use of vaccine from first vial puncture from 6 to 48 hours when vial stored at 2-8°C

11.	10	21 Jan 2021	PPD	Sequencing of SARS-CoV-2 added to study procedures and tertiary endpoints<br><br>Details on efficacy analysis for unblinding<br><br>Unblinding procedures
12	11	28 April 2021	PPD	Co-Participant Sites inclusion<br><br>Exclusion criteria updated with the exception of the licensed seasonal influenza vaccination and the licensed pneumococcal vaccination.<br><br>Secondary objectives including assessment of immunological correlates<br><br>Changes to symptomatic participant follow up (to reduce study procedure burden on participants and simplify safety data collection):<br><br>If there is already a negative PCR test outside of the trial during a symptomatic episode then no need to bring participant in for repeat swab<br><br>If there is a positive PCR test outside of the trial + symptoms then still bring in for repeat swab and immunology bloods (no safety bloods, observations or examination needed)

				No repeat swab at S3-S5 (A single negative PCR test in or out of the trial is<br>now sufficient to declare it a negative case)<br>Replace S7 physical visit with a remote phone call<br>Removal of symptomatic ediary<br>Removal of S3-5 repeat swab/visit<br>Immunological correlate of protection endpoint added<br>Change to exclusion criteria to permit participants to receive licensed seasonal influenza and pneumococcal vaccines from 14 days post vaccination<br>Unblinded exploratory COVID-19 disease endpoints may be determined by trained trial team members (rather than blinded independent endpoint assessors)
13	12	08 June 2021	PPD	Inclusion of third dose of ChAdOx1 nCoV-19 for subgroup 1 – which received 2 doses of ChAdOx1 nCoV-19 vaccine 4 weeks apart, 11-13 months after the second dose.

				Evaluation of immunogenicity and reactogenicity of the third dose.<br><br>Group 2 – inclusion of the third dose for a subgroup of 350 participants who received 2 doses of ChAdOx1 nCoV-19 12 weeks apart, 5-7 months after the second dose.<br><br>Evaluation of immunogenicity and reactogenicity of the third dose.<br><br>Groups 3 and 4 – evaluation of immunogenicity and reactogenicity of the third dose with ChAdOx1 nCoV-19 vaccine or with Sinovac/Butantan vaccine in a subgroup of 600 volunteers who previously received 2 doses of Sinovac/Butantan with an interval of 2-4 weeks.
14	12.1	29 June 2021	PPD	Inclusion of the third dose with ChAdOx1 nCoV-19 for the subgroup of participants who received 2 doses of the ChAdOx1 nCoV-19 vaccine with a 4-week interval, 11-13 months after the second dose.<br><br>Evaluation of immunogenicity and reactogenicity of the third dose.

15	12.2	19 July 2021	PPD	Inclusion of paracetamol after vaccination.<br><br>Interval of 4 to 12 weeks between the first and the second dose.<br><br>End of the third dose on October 31, 2021.<br><br>7-day diary for a subset of 100 participants.
16	13.0	08 Mar 2022	PPD	Inclusion of a fourth dose booster in participants from group 1e, who previously received two doses of ChAdOx1 nCov-19, followed by a third late dose of ChAdOx1 nCOV-19, regardless of interval between the third and fourth dose.<br><br>Evaluation of immunogenicity and reactogenicity of the fourth dose.

List details of all protocol amendments here whenever a new protocol version is produced.

COV003_ChAdOx1_nCoV_19_Trial_Protocol_V13_12Apr2022_Clean_ENG

Final Audit Report 2022-09-08

Created:	2022-08-22
By:	PPD
Status:	Signed
Transaction ID:	PPD

"COV003_ChAdOx1_nCoV_19_Trial_Protocol_V13_12Apr2022_Clean_ENG" History

- Document created by PPD
  2022-08-22 - 8:05:49 PM GMT

- Document emailed to PPD for signature
  2022-08-22 - 8:08:16 PM GMT

- Email viewed by PPD
  2022-08-22 - 9:36:13 PM GMT

- Email viewed by PPD
  2022-09-08 - 1:38:00 PM GMT

- Signer PPD entered name at signing as PPD
  2022-09-08 - 2:52:31 PM GMT

- Document e-signed by PPD
  Signature Date: 2022-09-08 - 2:52:32 PM GMT - Time Source: server

- Agreement completed.
  2022-09-08 - 2:52:32 PM GMT

Adobe Acrobat Sign

Redacted for Public Disclosure

Study Title: A Randomized, Controlled, Phase III Study to Determine the Safety, Efficacy, and Immunogenicity of the Non-Replicating ChAdOx1 nCoV-19 Vaccine.

Study Reference: COV003

Protocol Version: 14.0

Date: **21 August 2022**

Chief Investigator: PPD

Sponsor: University of Oxford

Funder: OXFORD University and external donors (Fundação Lemann, Fundação Brava, Fundação Telles, Instituto D'or de Ensino e Pesquisa and AstraZeneca Brasil, AstraZeneca UK)

Main contacts of the study

Chief Investigator PPD

Address
Center for Clinical Vaccinology and Tropical Medicine
University of Oxford,
Churchill Hospital, Old Road, Headington
Oxford, OX3 7LE
Email: PPD

Local Principal Investigator PPD
Centro de Referência para Imunobiológicos Especiais - CRIE
Universidade Federal de São Paulo - UNIFESP
Rua Borges Lagoa, 770 - Vila Clementino
São Paulo - SP, 04038-001, Brazil
Email: PPD

Co-Principal Investigator – National Coordination
PPD
PPD
PPD
Institute for Global Health - Siena University
Via Val Di Montone, 1
Siena, 53-100, Italy

Centro de Referência para Imunobiológicos Especiais - CRIE
Universidade Federal de São Paulo - UNIFESP
Rua Borges Lagoa, 770 - Vila Clementino
São Paulo - SP, 04038-001, Brazil
Email: PPD

Co-Investigators
PPD
PPD

Oxford Vaccine Group
Center for Clinical Vaccinology and Tropical Medicine

University of Oxford,
Churchill Hospital, Old Road, Headington
Oxford, OX3 7LE

Study Sites

Centro de Referência para Imunobiológicos Especiais - CRIE
Universidade Federal de São Paulo - UNIFESP
Rua Borges Lagoa, 770 - Vila Clementino
São Paulo - SP, 04038-001, Brazil

Instituto D'Or de Pesquisa e Ensino - I'Dor
Rua Diniz Cordeiro, 30 - Botafogo
Rio de Janeiro - RJ, 22281-100, Brazil

Co-Participant Site:
Hospital Gloria D'or
Rua Santo Amaro, 80
Rio de Janeiro - RJ

Instituto D'Or de Pesquisa e Ensino - I'Dor
Avenida São Rafael, 2152 - São Marcos
Salvador - BA, 41253-190, Brazil

Centro de Pesquisas Clinicas de Natal (CPCLIN)
Rua Dr Ponciano Barbosa, 282 – Cidade Alta
Natal – RN, 59025-050, Brazil

Universidade Federal de Santa Maria (UFSM)
Av. Roraima, 1000 Cidade Universitária Bairro Camobi
Santa Maria - RS, 97105-900, Brazil

	Hospital das Clínicas de Porto Alegre<br>Universidade Federal do Rio Grande do Sul (UFRGS).<br>Rua Ramiro Barcelos, 2.350 Bairro Santa Cecília<br>Porto Alegre – RS, 90035-903, Brazil
**Sponsor Institution**	University of Oxford
**Funder**	University of Oxford and external donors (Fundação Lemann, Fundação Brava, Fundação Telles, Instituto D'or de Ensino e Pesquisa and AstraZeneca Brasil, AstraZeneca UK)

Confidentiality Statement

This document contains confidential information that should not be disclosed to anyone other than the Sponsor, the Research Team, the host organization, members of the Research Ethics Committee, and other regulatory bodies. This information may not be used for any purpose other than evaluating or conducting clinical research without the prior written consent of the Chief Investigator.

Compliance Statement

The study will be conducted in compliance with the Protocol, the principles of Good Clinical Practices, Standards for Medicines for Human Use (Clinical Trial) 2004 (as amended), and all other applicable regulatory requirements.

Investigator's Agreement and Conflict of Interest Notification

I approve this Protocol for use in the abovementioned clinical trial and agree to comply with all provisions established therein.

In accordance with the current revision of the Declaration of Helsinki, I have read this protocol, and declare that I have no conflict of interest.

Lead Investigator	Signature	Date

Research Site

I have read this protocol and agree to comply with all the provisions established therein.

In accordance with the current revision of the Declaration of Helsinki, I have read this protocol, and declare that I have no conflict of interest.

PPD	PPD<br>PPD Sep 8, 2022 15:51 GMT+1)	08-Sep-2022
Principal Investigator	Signature	Date

TABLE OF CONTENTS

3.1 Background 19

3.2 Pre-clinical Studies 20

3.2.1 Immunogenicity (Jenner Institute) 20

3.2.2 Efficacy 23

3.2.3 Immunopathology and antibody-dependent potentiation 23

3.3 Previous clinical experience 24

3.4 Rationale 31

5.1 Study groups 40

5.2 Study participants 41

5.3 Definition of the End of Study 42

5.4 Potential risks for participants 42

5.5 Known potential benefits 43

6.1 Identification of Participants 44

6.2 Informed Consent Form 44

6.3 Inclusion and exclusion criteria 46

6.3.1 Inclusion Criteria 46

6.3.2 Exclusion Criteria 47

6.3.3 Re-vaccination exclusion criteria (two-dose groups only) 48

6.3.4 Exclusion Criteria for the third dose 49

6.3.5 Exclusion Criteria for the fourth dose 49

6.3.6 Effective birth control for volunteers 49

6.3.7 Withdrawal of participants 50

6.4 Pregnancy 51

7.1 Visit Schedule 51

7.2 Observations, medical history and physical examination 52

7.3 Blood samples, nose/throat swabs and urine analysis 52

7.4 Study visits 53

7.4.1 Screening visit and recruitment 53

7.4.2 Day 0: Recruitment and vaccination visit 54

7.4.2.1 Vaccination 54

7.4.3 Later visits 55

7.4.4 Symptomatic participants from groups 1a, 1b, 1c, 1d and 1e 65

7.4.4.1 Diagnostic SARS-CoV-2 PCR testing outside of the trial 65

7.4.4.2 COVID-19 testing visit 65

7.4.4.3 COVID-19 Testing plus 7 day phone call 66

7.4.4.4 Positive SARS-CoV-2 PCR test and vaccination 66

7.4.5 Symptomatic participants from Protocol 13 onwards 67

7.4.6 Review of the medical record 67

7.4.7 Randomization, blinding, and unblinding 67

7.4.8 Unblinded participants who are eligible to receive an approved or licensed SARS-CoV-2 vaccine 68

7.4.9 Eligible participants who will receive a third dose of ChAdOx1 nCoV-19 vaccine 70

7.4.10 Eligible participants who will receive a fourth dose of ChAdOx1 nCoV-19 vaccine 70

8.1 Description of ChAdOx1 nCoV-19 71

8.2 Storage 71

8.3 Administration 71

8.4 Rationale for selected dose 71

8.5 Environmental contamination control (GMO) 73

8.6 Investigational Medicinal Product for the 4th dose study 74

8.7 Control Vaccine 74

8.8 Placebo 75

8.9 Compliance with the investigational treatment 75

8.10 Investigational treatment accounting 75

8.11 Concomitant medication 75

8.12 Provision of treatment for controls 76

9.1 Definitions 76

9.1.1 Adverse Event (AE) 76

9.1.2 Adverse Reaction (AR) ... 76
9.1.3 Serious Adverse Event (SAE) ... 76
9.1.4 Serious Adverse Reaction (SAR) ... 77
9.1.5 Suspected Unexpected Serious Adverse Reaction (SUSAR) ... 77
9.2 Expectation ... 77
9.3 Predicted/expected adverse reactions: ... 78
9.4 Adverse Events of Special Interest ... 78
9.5 Causality ... 78
9.6 Reporting procedures for all adverse events ... 79
9.7 Evaluation of severity ... 80
9.8 Serious AE reporting procedures ... 82
9.9 Procedures for reporting SUSARs ... 83
9.10 Safety assessments ... 83
9.11 Data Safety Monitoring Committee ... 83
10.1 Description of statistical methods ... 84
10.2 Efficacy Outcomes ... 84
10.2.1 Efficacy ... 85
10.2.2 Safety and Reactogenicity ... 87
10.2.3 Immunogenicity ... 87
10.3 3rd and 4th dose studies ... 87
10.4 Subgroup analyses ... 88
10.5 Number of Participants ... 88
1.1.1 Fourth dose boost substudy ... 88
10.6 Interim and primary analyses of the primary outcome ... 89
10.7 Final Analysis ... 90
10.8 Inclusion in the analysis ... 90
10.9 Data and Safety Monitoring Committee ... 91

11.1 Data processing 91

11.2 Record keeping 92

11.3 Source data and technical data sheets (CRFs) 92

11.4 Data protection 92

11.5 Data quality 92

11.6 Archiving 93

12.1 Investigator's Procedures 94

12.2 Monitoring 94

12.3 Deviation from the protocol 94

12.4 Audit and inspection 94

13.1 Declaration of Helsinki 94

13.2 Guidelines for good clinical practices 94

13.3 Ethical and regulatory approvals 95

13.4 Volunteers confidentiality 95

14.1 Funding 96

14.2 Insurance 96

14.3 Publication Policy 96

1 SYNOPSIS

**Title**	A Randomized, Controlled, Phase III Study to Determine the Safety, Efficacy, and Immunogenicity of the Non-Replicating ChAdOx1 nCoV-19 Vaccine.
**Study Identifier**	COV003
**Trial Record**	https://www.isrctn.com/ (registration number: ISRCTN89951424)
**Study Sites**	Centro de Referência para Imunobiológicos Especiais (CRIE) - UNIFESP, Universidade Federal de São Paulo, Rua Borges Lagoa, 770, Vila Clementino, Zip Code 04038-001, São Paulo/SP, Brazil<br><br>Instituto D'Or de Pesquisa e Ensino<br>Rua Diniz Cordeiro, 30, Botafogo,<br>Zip Code 22281-100, Rio de Janeiro - RJ, Brazil<br><br>Instituto D'Or de Pesquisa e Ensino<br>Avenida São Rafael, 2152, São Marcos,<br>Zip Code 41253-190, Salvador - BA, Brazil<br><br>Centro de Pesquisas Clínicas de Natal (CPCLIN)<br>Rua Dr Ponciano Barbosa, 282 – Cidade Alta<br>Natal – RN, 59025-050, Brazil<br><br>Universidade Federal de Santa Maria (UFSM)<br>Av. Roraima, 1000 Cidade Universitária Bairro Camobi<br>Santa Maria - RS, 97105-900, Brazil<br><br>Hospital das Clínicas de Porto Alegre<br>Universidade Federal do Rio Grande do Sul (UFRGS).

	Rua Ramiro Barcelos, 2.350 Bairro Santa Cecília<br>Porto Alegre – RS, 90035-903, Brazil
**Clinical Phase**	3
**Design**	A Single-Blind, Randomized Study of Safety, Efficacy, and Immunogenicity.
**Population**	Health professionals and adults with high potential for exposure to SARS-CoV-2, aged ≥18 years.
**Planned Sample Size**	The total sample size will be up to 10,300 participants (with a margin of 1%).
**Planned Duration**	11 to 13 months post second vaccine dose, per participant<br>or<br>6 to 7 months post fourth vaccine dose, per participant in Group 2

**MAIN STUDY OBJECTIVES**		
	**Objective**	**Endpoint Measure**
**Primary Objective**	To evaluate the efficacy of ChAdOx1 nCoV-19 vaccine against COVID-19 disease confirmed with PCR.	a) COVID-19 virologically confirmed symptomatic cases (PCR positive).
**Secondary Objectives**	To evaluate the safety, tolerability, and reactogenicity profile of ChAdOx1 nCoV-19 candidate vaccine.	a) Occurrence of signs and symptoms of local and systemic reactogenicity requested during 7 days after vaccination (in a subset of 200 participants*);<br>b) Occurrence of serious adverse events;<br>c) Occurrence of disease enhancement episodes.

	To evaluate the efficacy of ChAdOx1 nCoV-19 candidate vaccine against severe and non-severe COVID-19 disease.	a) Hospitalization for COVID-19 disease confirmed by PCR;<br>b) COVID-19 severe disease confirmed by PCR;<br>c) Death associated with COVID-19 disease;<br>d) Antibodies against SARS-CoV-2 non-Spike protein (efficacy against non-spike seroconversion rates).
	To assess the humoral immunogenicity of ChAdOx1 nCoV-19 candidate vaccine.	a) Antibodies against SARS-CoV-2 spike protein (sero-conversion rates).<br>b) Virus neutralizing antibodies (NAb) against live and/or pseudotyped SARS-CoV-2 virus.
	To assess the cellular immunogenicity of ChAdOx1 nCoV-19 candidate vaccine.	a) Interferon-gamma (IFN-γ) enzyme-linked immunospot (ELISpot) responses to SARS-CoV-2 spike protein;
	To assess immunological correlates of protection in relation to occurrence of COVID-19 disease in ChAdOx1 nCoV-19 recipients	Immunological endpoints and COVID-19 disease endpoints in ChAdOx1 nCoV-19 recipients
**3rd DOSE OBJECTIVES**		
	**Objective**	**Endpoint Measure**
**Secondary Objectives**	To evaluate the reactogenicity profile of the third dose of ChAdOx1 nCoV-19 candidate vaccine.	Occurrence of signs and symptoms of local and systemic reactogenicity requested during 7 days after vaccination.
	To determine if the neutralising antibody response to a third dose ChAdOx1 nCoV-19 is non-inferior to the response to the ChAdOx1 nCoV-19 primary series	GMTR of nAb response to the ancestral strain 28 days after a 3rd dose administration vs 28 days after primary series

	To determine if the humoral immune response to a 3rd dose ChAdOx1 nCoV-19 is non-inferior to the response to the ChAdOx1 nCoV-19 primary series	GMTR of Spike binding antibody response to the ancestral strain 28 days after 3rd dose administration vs 28 days after primary series
**4th DOSE OBJECTIVES****		
	**Objective**	**Endpoint Measure**
**Primary Objective*****	To determine if the neutralising antibody response to a 4th dose ChAdOx1 nCoV-19 is non-inferior to the response to the first ChAdOx1 nCoV-19 dose in a subgroup of study participants	GMTR of nAb response to the ancestral strain 28 days after 4th dose administration vs 28 days after a 3rd dose
**Secondary Objectives**	To determine if the humoral immune response to a 4th dose ChAdOx1 nCoV-19 is non-inferior to the response to the first ChAdOx1 nCoV-19 booster dose in a subgroup of study participants	a.) GMTR of nAb response to a contemporary variant of concern 28 days after 4th dose administration vs 28 days after a 3rd dose<br>b.) GMTR of Spike binding antibody response to the ancestral strain 28 days after 4th dose administration vs 28 days after a 3rd dose
	To determine if the humoral immune response to a 4th dose ChAdOx1 nCoV-19 is non-inferior to the response to primary series ChAdOx1 nCoV-19 in a subgroup of study participants	a.) GMTR of nAb response to the ancestral strain 28 days after 4th dose administration vs 28 days after primary series ChAdOx1 nCoV-19<br>b.) GMTR of nAb response to a contemporary variant of concern 28 days after 4th dose administration vs 28 days after primary series ChAdOx1 nCoV-19

		c.) GMTR of Spike binding antibody response to the ancestral strain, variant of concern 28 days after 4th dose administration vs 28 days after primary series ChAdOx1 nCoV-19
	To characterise the cellular immune responses of a 4th dose ChAdOx1 nCoV-19 in seronegative and seropositive participants who previously received an initial ChAdOx1 nCoV-19 boost in a subgroup of participants	a.) Cellular immune responses by ICS (Th1/Th2) over time<br>b.) Cellular immune responses by ELISpot over time
	To evaluate the reactogenicity profile of the fourth dose of ChAdOx1 nCoV-19 vaccine.	a) Occurrence of signs and symptoms of local and systemic reactogenicity requested during 7 days after vaccination (in a subset of 100 participants).<br>b) Recording of unsolicited AEs for 28 days after vaccination
**Exploratory objectives**	To explore anti-vector responses to the ChAdOx-1 vector of a 4th dose ChAdOx1 nCoV-19 in seronegative and seropositive participants who previously received an initial ChAdOx1 nCoV-19 boost.	a.) Geometric mean titres of ChAdOx1 neutralising antibody titres over time<br>b.) Pairwise correlations between anti-S, pseudo-neutralisation, and live neutralisation antibody titres over time
	To further evaluate humoral and cellular immunogenicity across all doses through exploratory immunology	Exploratory immunology such as systems serology profiling

**Investigational Products**	a) ChAdOx1 nCoV-19, a replication-defective adenoviral vector expressing the SARS-CoV-2 spike (S) protein;<br>b) MenACWY vaccine;<br>c) Saline Placebo (for the control arm boosting dose)

**Detailed assessments of local and systemic reactogenicity for 7 days after vaccination with ChAdOx1 nCoV-19 compared to MenACWY as a control have been documented in a sufficient number of participants in previous studies. In study COV003, detailed local and systemic reactogenicity will be evaluated in 200 randomized participants, who received two doses, a quantity statistically determined to ensure proportionality and comparative representativeness compared to studies COV001 and COV002. Local and systemic reactogenicity will be evaluated for 7 days in all participants who received the third dose and in a subset of 100 participants that have received the fourth dose.*

*** The fourth dose will be administered to a subset of 350 participants who have previously received 3 doses of ChAdOx1 nCoV-19 vaccine without prior confirmed COVID-19 infections in the last four weeks.*

**** If the number of participants seronegative at baseline, intra-subject correlation and the observed standard derivation combine to provide ≥ 80% power for non-inferiority assessments, the analysis will be performed as planned. Alternatively, if any factor or combination of factors results in <80% power, the primary analysis will be descriptive immunogenicity only, with all comparative analyses being exploratory, including any subgroup comparison. The details will be described in the SAP.*

**Formulation**	ChAdOx1 nCoV-19: Liquid<br>MenACWY: powder and solvent for solution for injection
**Route of Administration**	Intramuscular (IM)
**Doses per Administration**	ChAdOx1 nCoV-19: 5 x 10^{10} vp<br>ChAdOx1 nCoV-19: 0.5mL (3.5 x 10^{10} to 6.5 x 10^{10})<br>MenACWY: 0.5 mL<br>0.9% saline solution: 0.5mL<br>Both groups will receive prophylactic paracetamol: 500mg - 1 g q6h/24 hours.

2 ABBREVIATIONS

**AdHu**	Human adenovirus
**AdHu5**	Human adenovirus serotype 5
**ADE**	Antibody-Dependent Enhancement
**AE**	Adverse Event
**AESI**	Adverse Event of Special Interest
**AID**	Autoimmune Disease
**CCVTM**	Centre for Clinical Vaccinology and Tropical Medicine, Oxford
**CBF**	Clinical Bio manufacturing Facility
**ChAdOx**	Chimpanzee adenovirus 1
**CI**	Confidence interval
**COP**	Code of Practice
**CRF**	Clinical Record or Clinical Research Facility
**CTRG**	Clinical Trials & Research Governance Office, University of Oxford
**CTL**	Cytotoxic T Lymphocyte
**DSMB**	Data and Safety Monitoring Board
**DSUR**	Development Safety Update Report
**ELISPOT**	Enzyme-linked immunospot
**GCP**	Good Clinical Practices
**GMO**	Genetically modified organism
**GMT**	Geometric mean titer
**GP**	General Practitioner
**HCG**	Human Chorionic Gonadotrophin
**HEK**	Human embryonic kidney
**HLA**	Human leukocyte antigen
**HRA**	Health Research Authority
**IB**	Investigator's Brochure

**ICH**	International Conference on Harmonization
**ICMJE**	International Committee of Medical Journal Editors
**ICS**	Intracellular Cytokine Staining
**ID**	Intradermal
**IFNγ**	Gamma interferon
**IM**	Intramuscular
**IMP**	Investigational medicinal product
**IMP-D**	Investigational Medicinal Product Dossier
**IV**	Intravenous
**MenACWY**	Quadrivalent meningococcal conjugate vaccine (protein-polysaccharide) against group A, C, W, and Y capsular serotype
**MHRA**	Medicines and Healthcare Products Regulatory Agency
**MVA**	Modified Vaccinia Ankara virus
**NHS**	National Health Service
**NIH**	National Institutes of Health
**NIHR**	National Institute for Health Research
**PBMC**	Peripheral blood mononuclear cell
**PCR**	Polymerase chain reaction
**PI**	Principal Investigator
**QP**	Qualified Person
**qPCR**	Quantitative polymerase chain reaction
**REC**	Research Ethics Committee
**SAE**	Serious adverse event
**SC**	Subcutaneous
**SOP**	Standard Operating Procedure
**SUSAR**	Suspected unexpected serious adverse reaction

**µg**	Microgram
**VP**	viral particle
**VV**	viral vector
**WHO**	World Health Organization

3 BACKGROUND AND RATIONALE

3.1 Background

In December 2019, a group of pneumonia patients of unknown cause was linked to a wholesale seafood market in Wuhan, China, and it was later confirmed that they were infected with a new coronavirus, known as 2019-nCoV [1]. The virus was later renamed SARS-CoV-2 because it is similar to the coronavirus responsible for severe acute respiratory syndrome (SARS-CoV), a *Betacoronavirus* lineage B. SARS-CoV-2 shares more than 79% of its sequence with SARS-CoV, and 50% with the coronavirus responsible for Middle East respiratory syndrome (MERS-CoV), a member of *Betacoronavirus* lineage C[2]. Covid-19 is the infectious disease caused by SARS-CoV-2. In January 2020, there was an increase in evidence of human-to-human transmission as the number of cases began to increase rapidly in China. Despite the unprecedented containment measures adopted by the Chinese government, SARS-CoV-2 has spread rapidly across the world. WHO declared the COVID-19 outbreak as an international public health emergency on January 30, 2020.

Coronaviruses (CoVs) are large, spherical, and enveloped single-stranded RNA genomes. A quarter of its genome is responsible for encoding structural proteins, such as glycoprotein spike (S), envelope (E), membrane (M) and nucleocapsid proteins (N). E, M, and N are mainly responsible for virion assembly, while protein S is involved in binding to the receptor, mediating the entry of the virus into host cells during CoVs infection through different receptors. [3] SARS-CoV-2 belongs to the phylogenetic lineage B of the genus *Betacoronavirus* and recognizes the angiotensin-converting enzyme 2 (ACE2) as an input receptor[4]. This is the seventh CoV that has been proven to cause infections in humans and the third that has been proven to cause serious illness after SARS-CoV and MERS-CoV.

The spike protein is a type I transmembrane, trimeric, type I glycoprotein located on the surface of the viral envelope of CoVs, which can be divided into two functional subunits: the N-terminal S1 and the C-terminal S2. S1 and S2 are responsible for the binding to the cellular receptor through the receptor binding domain (RBD, for its acronym in English) and fusion of virus and cell membranes, respectively, thereby mediating the entry of SARS-CoV-2 into target cells. [3] The functions of S in receptor binding and membrane fusion make it an ideal target for vaccine and antiviral development, as it is the main target for neutralizing antibodies.

ChAdOx1 nCoV-19 vaccine consists of a replication-deficient monkey adenoviral vector ChAdOx1, containing the SARS CoV-2 structural surface glycoprotein antigen (spike protein) (nCoV-19), with a signal sequence from the leading tissue plasminogen activator (tPA). ChAdOx1 nCoV-19 expresses a codon-optimized coding sequence for the Spike protein from GenBank genomic sequence access: MN908947. The leader tPA sequence was shown to be beneficial in increasing the immunogenicity of another CoV vaccine vectorized by ChAdOx1 (ChAdOx1 MERS)[5].

3.2 Pre-clinical Studies

Refer the Investigator's Brochure for the most recent update of preclinical data.

3.2.1 Immunogenicity (Jenner Institute)

The mice (balb/c and CD-1) were immunized with ChAdOx1 which expresses the SARS-CoV-2 Spike protein or green fluorescent protein (GFP). From 9 to 10 days after vaccination, spleen samples were used to assess IFN-γ ELISpot responses and serum samples for assessments of S1 and S2 antibody responses with ELISA. The results of these studies show that a single dose of ChAdOx1 nCoV was immunogenic in mice.

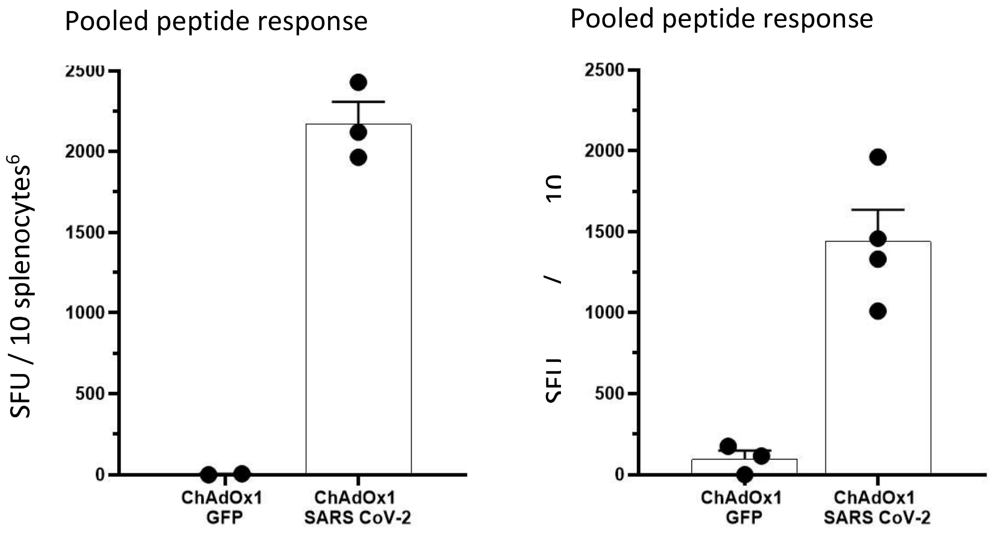

Figure 1. Splenic responses combined with IFN-γ ELISpot from BALB/c (left panel) and CD-1 (right panel) mice, in response to peptides that comprise the SARS-CoV-2 spike protein, nine or ten days after vaccination, with 1.7×10^{10} pv ChAdOx1 nCoV-19 or 8×10^{9} pv ChAdOx1 GFP. The means with SEM are described.

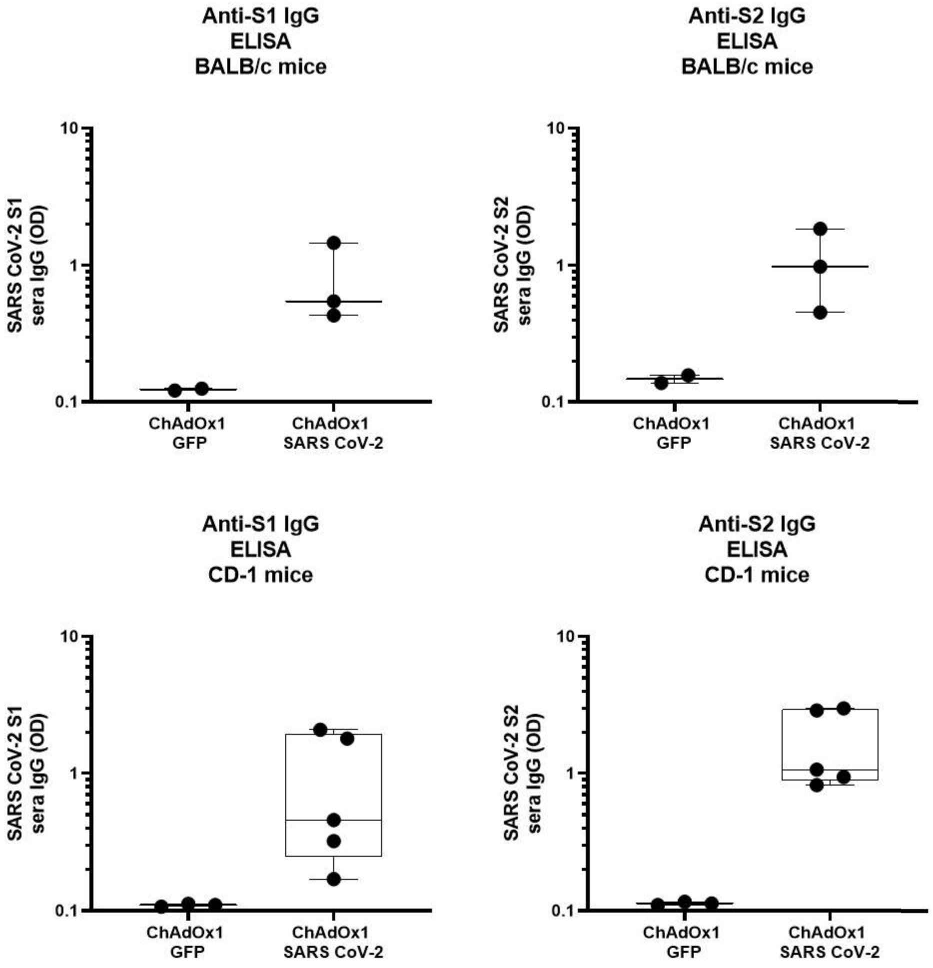

Figure 2. Box and mustache plot of optical densities after ELISA analysis of BALB/C mouse serum (top panel) incubated with purified protein spanning the S1 domain (left) or purified protein spanning the S2 domain (right) of the SARS-CoV spike -2 nine or ten days after vaccination, with 1.7×10^{10} pv ChAdOx1 nCoV-19 or 8×10^{9} pv ChAdOx1 GFP. Box and mustache plots of optical densities after ELISA analysis of CD-1 mouse serum (bottom panel) incubated with purified protein spanning the S1 domain (left) or purified protein spanning the S2 domain (right) of the SARS-CoV spike -2.

A second experiment was performed with a different dose. The results are summarized in the figure below. Intracellular cytokine staining shows a pattern consistent with predominantly Th1 responses.

A. ELISpot Responses

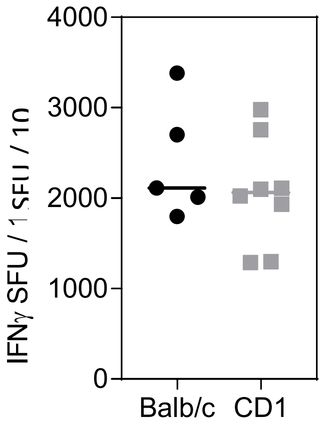

B. Intracellular cytokine staining

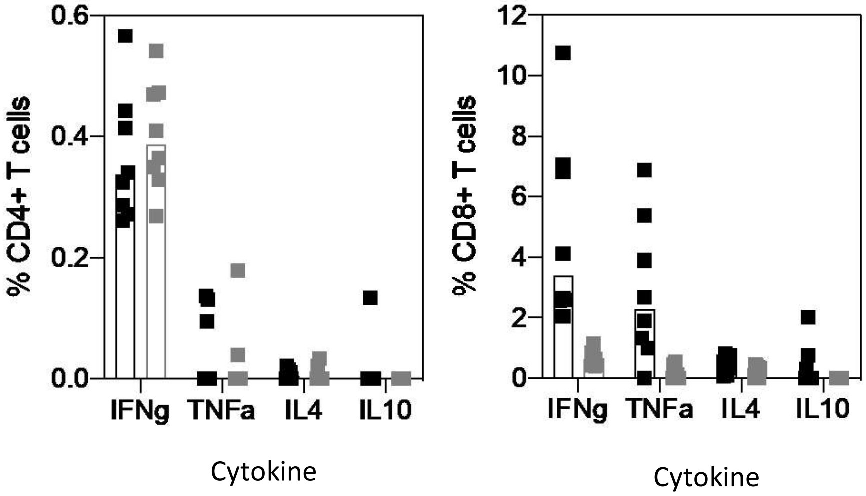

Figure 3. Specific antigen responses after vaccination with ChAdOx nCov19. 10^8 UI ChAdOx nCoV-19 was administered intramuscularly to heterogeneous BALB/c mice (CD1). Fourteen days later, the harvested spleens and cells stimulated peptides that span the extension of S1 and S2.

A. The graph shows the IFN-γ ELIspot responses summed up in BALB/c (black circles) and heterogeneous cd1 (gray squares) mice.
B. The graphs show the frequency of cytokine positive CD4 (left) or CD8 (right) cells, measured by intracellular cytokine staining after splenocyte stimulation with clustered S1 (black) or clustered S2 (gray) peptides in CD1 mice.

3.2.2 Efficacy

Pre-clinical efficacy studies of ChAdOx1 nCoV-19 in ferrets and non-human primates are ongoing. These results are included in the Investigator's Brochure.

3.2.3 Immunopathology and antibody-dependent potentiation

Safety concerns have arisen around the use of coronavirus Spike glycoproteins to their full extent and other viral antigens (nucleoprotein) as vaccine antigens after historical and limited reports of immunopathology and antibody dependent potentiation (ADE) reported *in vitro* and post challenge with SARS-CoV in mice, ferrets and non-human primates immunized with vaccines based on inactivated complete SARS-CoV or protein S in its full extension, including a study that used modified Vaccinia Ankara as a vector. [6-8] So far, there has been a report of pulmonary immunopathology after the challenge with MERS-CoV in mice immunized with a candidate vaccine against inactivated MERS-CoV. [9] However, in preclinical trials of immunization with ChAdOx1 and challenge with MERS-CoV, ADE was not observed in hDPP4 transgenic mice, dromedary camels or non-human primates (van Doremalen et al, submitted manuscript).[10.11]

The risks of inducing pulmonary immunopathology in the case of COVID-19 after vaccination with ChAdOx1 nCoV-19 are unknown. The NHP study conducted by NIH described in the investigator’s brochure showed no evidence of immune-enhanced inflammation in ChAdOx1 nCoV-19 vaccinated animals who underwent SARS-CoV-2 challenge 4 weeks post immunization, at 7 days post challenge. Results from a separate challenge study conducted on a purified inactivated SARS-CoV-2 vaccine also corroborate with NIH findings where no ADE has been detected in vaccinated animals [1]. However, the negative findings on ADE and lung immunopathology from both reports should be interpreted with caution, as challenged animals were sacrificed and examined shortly after challenge (7 days post inoculation). Challenge studies in ferrets and non-human primates (PNH) are ongoing and these pre-clinical trials will report on the presence or absence of pulmonary pathology. The results will be reviewed as soon as they are available and will be part of the risk/benefit discussions for participants who receive the investigational product (IMP). All pathological data from challenge studies of other SARS-CoV-2 candidate vaccines will also be taken into account.

3.3 Previous clinical experience

Prior to current COVID-19 studies, vaccines vectorized by ChAdOx1 that express different inserts have been used previously in more than 320 healthy volunteers participating in clinical trials conducted by University of Oxford in the United Kingdom and abroad (tables 1 and 2). Most importantly, a ChAdOx1 vector vaccine expressing the total Spike protein from another *Betacoronavirus*, MERS-CoVCoV, has been administered to 31 participants, so far, as part of the MERS001 and MERS002 studies. ChAdOx1 MERS was administered in doses ranging from $5x10^9$ pv $5x10^{10}$ pv (table 2) with no serious adverse reactions reported. Further references and results on safety and immunogenicity about ChAdOx1 MERS can be found in the ChAdOx1 Investigator's Brochure nCoV-19.

Clinical trials of ChAdOx1 vectorized vaccines encoding antigens for Influenza (NP + M1 fusion protein), Tuberculosis (85A), Prostate Cancer (5T4), Malaria (LS2), Chikungunya (structural polyprotein), Zika (prM and E), MERS-CoV (total spike protein) and Meningitis B are listed below.

None of the clinical trials mentioned below reported serious adverse events associated with the administration of ChAdOx1, which had a good safety profile.

Table 1. Non-COVID-19 clinical experience with ChAdOx1 viral vector vaccines.

Country	Study	Vaccine	Age	Route	Dose	Number of volunteers (received ChAdOx1)	Publication/Registration Number
UK	FLU004	ChAdOx1 NP + M1	18-50	IM	$5x10^{8}$ pv	3	Antrobus et al, 2014. Molecular Therapy. DOI: 10.1038/mt. 2013.284 [2]
					$5x10^{9}$ pv	3	
					$2.5x10^{10}$ pv	3	
					$5x10^{10}$ pv	6	
UK	FLU005	ChAdOx1 NP + M1<br>MVA NP + M1 (week 8)	18-50	IM	$2.5x10^{10}$ pv	12	Coughlan et al, 2018. EBioMedicine DOI: 10.1016/j.ebiom.2018.02.011 DOI: 10.1016/j.ebiom.2018.05.001 [3]
		ChAdOx1 NP + M1<br>MVA NP + M1 (week 52)	18-50	IM	$2.5x10^{10}$ pv	12	
		MVA NP+M1<br>ChAdOx1 NP + M1 (week 8)	18-50	IM	$2.5x10^{10}$ pv	12	
		MVA NP+M1<br>ChAdOx1 NP + M1 (week 52)	18-50	IM	$2.5x10^{10}$ pv	9	
		ChAdOx1 NP + M1	>50	IM	$2.5x10^{10}$ pv	12	

Country	Study	Vaccine	Age	Route	Dose	Number of volunteers (received ChAdOx1)	Publication/Registration Number
		ChAdOx1 NP + M1<br>MVA NP + M1 (week 8)	>50	IM	$2.5x10^{10}$ pv	12	
		ChAdOx1 85A	18-50	IM	$5x10^{9}$ pv	6	Wilkie et al, 2020 Vaccine
					$2.5x10^{10}$ pv	12	
UK	TB034	ChAdOx1 85A<br>MVA85A (week 8)	18-50	IM	$2.5x10^{10}$ pv	12	
		ChAdOx1 85A<br>(x2, 4 weeks apart)<br>MVA85A (in 4 months)	18-50	IM	$2.5x10^{10}$ pv	12	
Switzerland	TB039<br>(ongoing)	ChAdOx1 85A	18-55	Aerosol	$1x10^{9}$ pv	3	Clinicaltrials.gov:<br>NCT04121494
				Aerosol	$5x10^{9}$ pv	3	
				Aerosol	$1x10^{10}$ pv	11	
				Aerosol/IM	$1x10^{10}$ pv	15	
Uganda	TB042<br>(ongoing)	ChAdOx1 85A	18-49	IM	$5x10^{9}$ pv	6	Clinicaltrials.gov:<br>NCT03681860
					$2.5 x10^{10}$	6	

Country	Study	Vaccine	Age	Route	Dose	Number of volunteers (received ChAdOx1)	Publication/Registration Number
UK	VANCE01	ChAdOx1.5T4<br>MVA.5T4	18 - 75	IM	$2.5x10^{10}$ pv	34	Clinicaltrials.gov:<br>NCT02390063
UK	ADVANCE (ongoing)	ChAdOx1.5T4<br>MVA.5T4	≥18	IM	$2.5x10^{10}$ pv	23 (on Feb 20)	Clinicaltrials.gov:<br>NCT03815942
UK	VAC067	ChAdOx1 LS2	18-45	IM	$5x10^{9}$ pv<br>$2.5x10^{10}$ pv	3<br>10	Clinicaltrials.gov:<br>NCT03203421
UK	VAMBOX	ChAdOx1 MenB.1	18-50	IM	$2.5x10^{10}$ pv<br>$5x10^{10}$ pv	3<br>26	ISRCTN46336916
UK	CHIK001	ChAdOx1 Chik	18-50	IM	$5x10^{9}$ pv<br>$2.5x10^{10}$ pv<br>$5x10^{10}$ pv	6<br>9<br>9	Clinicaltrials.gov:<br>NCT03590392<br>DOI: https://doi.org/10.4269/ajtmh.abstract2019<br>Abstract # 59, page 19.
UK	ZIKA001	ChAdOx1 Zika	18-50	IM	$5x10^{9}$ pv	6	Clinicaltrials.gov:

Country	Study	Vaccine	Age	Route	Dose	Number of volunteers (received ChAdOx1)	Publication/Registration Number
	(ongoing)				$2.5x10^{10}$ pv	3 (on Feb 20)	NCT04015648
					$5x10^{10}$ pv	-	

Table 2. Clinical experience with ChAdOx1 MERS

Country	Study	Vaccine	Age	Route	Dose	Number of volunteers (received ChAdOx1)	Publication/Registration Number
UK	MERS001 (ongoing)	ChAdOx1 MERS	18-50	IM	$5x10^9$ pv<br>$2.5x10^{10}$ pv<br>$5x10^{10}$ pv<br>$2.5x10^{10}$ pv Initiation - homologous reinforcement	6<br>9<br>9<br>3	Clinicaltrials.gov: NCT03399578<br>Folegatti et.al. 2020, Lancet Infect.Dis<br>DOI: https://doi.org/10.1016/S1473-3099(20)30160-2
Saudi Arabia	MERS002 (ongoing)	ChAdOx1 MERS	18-50	IM	$5x10^9$ pv<br>$2.5x10^{10}$ pv<br>$5x10^{10}$ pv	4<br>3<br>-	Clinicaltrials.gov: NCT04170829

The first clinical trial of the ChAdOx1 nCoV-19 candidate vaccine (COV001) started on April 23 2020, after approval by the IRB and CTA by the MHRA. ChAdOx1 nCoV-19 candidate vaccine has been studied in the following clinical trials:

- Eight clinical trials evaluating ChAdOx1 nCoV-19 given to adults as a 2-dose primary series: 5 ongoing studies sponsored by the University of Oxford, (COV001, COV002, COV003, COV004 and COV005), 2 ongoing studies sponsored by AstraZeneca (D8110C00001 and D8111C00002), and 1 completed study sponsored by the Serum Institute of India (COVISHIELD).
- One ongoing University of Oxford study evaluates ChAdOx1 nCoV-19 given as a 2-dose primary series to children and adolescents (COV006).
- Five additional clinical trials or sub-studies in adults. Of these:
    - One AstraZeneca study evaluates ChAdOx1 nCoV-19 given as a 2-dose primary series in immunocompromised adults (D8111C0010).
    - Two studies evaluate ChAdOx1 nCoV-19 as a booster immunisation at least 10 months after the first vaccine dose, are sub-studies of COV001 and COV003 (noted above) and are ongoing.
    - Three studies evaluate a 2-dose primary series consisting of ChAdOx1 nCoV-19 and another adenoviral vector-based SARS-CoV-2 vaccine, rAD26-S (CV03872097, formerly known as D8111C00003, CV03872091, and CV03872092), are sponsored by RPharm and are ongoing.
- One study sponsored by AstraZeneca has also evaluated AZD2816, a vaccine that uses the same adenoviral platform as ChAdOx1 nCoV-19 but is based on the Beta variant (B.1.351). The study evaluates both AZD2816 and ChAdOx1 nCoV-19 when given to adults with no prior history of SARS CoV-2 vaccination, and when given as a third dose boost to adults who previously completed a primary vaccination course with ChAdOx1 nCoV-19 or a SARS CoV-2 mRNA vaccine.

These clinical trials are summarised in full in the Investigator's Brochure.

3.4 Rationale

The epidemic of COVID-19 has caused a major disruption in health systems with significant socioeconomic impacts. Containment measures have failed to stop the spread of the virus, which has reached pandemic levels. Currently, there are no specific treatments available against COVID-19 and accelerated vaccine development is sorely needed.

Live attenuated viruses have historically been one of the most immunogenic platforms available, as they have the ability to present multiple antigens throughout the viral life cycle in their native conformations. However, the manufacture of live attenuated viruses requires complex measures of containment and biosafety. In addition, live attenuated viruses carry the risk of inadequate attenuation, causing widespread disease, particularly in immunocompromised hosts. Given that COVID-19 is a serious and potentially fatal disease, which disproportionately affects elderly people with comorbidities, producing a vaccine with live attenuated viruses is the least viable option. The replication of competent viral vectors may pose a similar threat in relation to the disease spread in immunosuppressed individuals. Vectors with poor replication, however, avoid this risk by maintaining the advantages of the presentation of native antigens, increased immunity of T cells and the ability to express multiple antigens[15]. Subunit vaccines generally require the use of adjuvants and, although DNA and RNA vaccines can offer manufacturing advantages, they are often precariously immunogenic and require multiple doses, which is highly undesirable in the context of a pandemic.

Chimpanzee adenovirus vaccine vectors have been safely administered to thousands of people targeting a wide range of infectious diseases. ChAdOx1 vectorized vaccines were administered to more than 320 volunteers without safety concerns and were shown to be highly immunogenic with the administration of single doses. Relevant information refers to recent clinical trials where a single dose of a vectorized ChAdOx1 vaccine expressing the total spike protein of another beta-choronavirus (MERS-CoV) has been shown to induce neutralizing antibodies.

The use of an active vaccine as a comparator for the control group will minimize the chances of accidentally unblinding the participant, reducing the bias in the analysis of reactogenicity, in the safety report and/or changes in the search for health services once that were symptomatic for COVID-19.

Groups 1c and 1d have been added following interim immunogenicity results on homologous prime-boost groups (as part of the COV001 UK study – see investigator's brochure for further details) showing improved neutralizing antibody titers after 2 doses when compared to 1 dose regimen. A saline placebo will be used in place of an active comparator in group 1d. As we have seen less reactogenicity when giving the booster dose in the UK studies, there is less risk of unblinding the participant by using placebo as a comparator for the booster dose.

Late/annual booster vaccination

An urgent public health question related to COVID-19 vaccination that remains unmet is whether seasonal booster vaccinations may be necessary to enhance immune responses and strengthen protection against the SARS-CoV-2 virus. However, the impact of an annual vaccination with the same adenoviral vector on antigen responses remains uncertain. There are concerns that repeated doses of adenovirus vector vaccines may generate anti-vector immunity, which could compromise the vaccine response, although we have previously shown that ChAdOx1 anti-vector antibodies do not affect anti-spike or anti-spike antibody responses, the specific T cell responses against the S-protein (Spike) and also that individuals who received a previous vaccine with the ChAdOx1 vector for a non-spike transgene at least one year prior to receiving ChAdOx1 nCoV-19 have similar S-protein binding antibody responses (Spike) compared to ChAdOx1 naive individuals.

The addition of a third late dose of ChAdox1 nCoV-19 in subgroups of study participants who have already received two doses will allow the assessment of the immunogenicity and safety of a booster dose.

Fourth dose

A homologous third dose vaccine was shown to be effective in mounting a significant humoral immune response, with acceptable reactogenicity in the UK[20]. However, the emergence of new SARS-CoV-2 virus variants and the reported decrease in antibody titers in vaccinated subjects are of concern.

Because of the rapid decline of neutralizing activity and reduction in vaccine effectiveness against Omicron variant even in individuals who have received 3 doses of vaccine, regulatory authorities (i.e., Israel, Germany, Brazil[21]) now recommend a 4th dose especially for risk groups. The results of an Israeli effectiveness study suggest that a homologous fourth dose of mRNA vaccines could increase protection against severe illness relative to three doses that have been administered over four months ago[22,23]. Therefore, considering the current pandemic scenario and the risk of new variants surge, assessing the immunogenicity and safety of a 4th dose in individuals, who have already received three doses of ChAdOx1 nCOV-19, can underpin public health decisions and policy making.

4 OBJECTIVES AND ENDPOINTS

	MAIN STUDY OBJECTIVES	
	**Objective**	**Endpoint Measure**
**Primary Objective**	To evaluate the efficacy of ChAdOx1 nCoV-19 vaccine against COVID-19 disease confirmed with PCR.	a) COVID-19 virologically confirmed symptomatic cases (PCR positive).
**Secondary Objectives**	To evaluate the safety, tolerability, and reactogenicity profile of ChAdOx1 nCoV-19 candidate vaccine.	a) Occurrence of signs and symptoms of local and systemic reactogenicity requested during 7 days after vaccination (in a subset of 200 participants*);<br>b) Occurrence of serious adverse events;<br>c) Occurrence of disease enhancement episodes.
	To evaluate the efficacy of ChAdOx1 nCoV-19 candidate vaccine against severe and non-severe COVID-19 disease.	a) Hospitalization for COVID-19 disease confirmed by PCR;<br>b) COVID-19 severe disease confirmed by PCR;<br>c) Death associated with COVID-19 disease;<br>d) Antibodies against SARS-CoV-2 non-Spike protein (efficacy against non-spike seroconversion rates).
	To assess the humoral immunogenicity of ChAdOx1 nCoV-19 candidate vaccine.	a) Antibodies against SARS-CoV-2 spike protein (sero-conversion rates).<br>b) Virus neutralizing antibodies (NAb) against live and/or pseudotyped SARS-CoV-2 virus.

	To assess the cellular immunogenicity of ChAdOx1 nCoV-19 candidate vaccine.**	a) Interferon-gamma (IFN-γ) enzyme-linked immunospot (ELISpot) responses to SARS-CoV-2 spike protein;
	To assess immunological correlates of protection in relation to occurrence of COVID-19 disease in ChAdOx1 nCoV-19 recipients	Immunological endpoints and COVID-19 disease endpoints in ChAdOx1 nCoV-19 recipients
**3rd DOSE OBJECTIVES**		
	**Objective**	**Endpoint Measure**
Secondary Objectives	To evaluate the reactogenicity profile of the third dose of ChAdOx1 nCoV-19 candidate vaccine.	Occurrence of signs and symptoms of local and systemic reactogenicity requested during 7 days after vaccination.
	To determine if the neutralising antibody response to a third dose ChAdOx1 nCoV-19 is non-inferior to the response to the ChAdOx1 nCoV-19 primary series	GMTR of nAb response to the ancestral strain 28 days after a 3rd dose administration vs 28 days after primary series
	To determine if the humoral immune response to a 3rd dose ChAdOx1 nCoV-19 is non-inferior to the response to the ChAdOx1 nCoV-19 primary series	GMTR of Spike binding antibody response to the ancestral strain 28 days after 3rd dose administration vs 28 days after primary series
**4th DOSE*** OBJECTIVES**		
	**Objective**	**Endpoint Measure**

**Primary Objective**** **	To determine if the neutralising antibody response to a 4th dose ChAdOx1 nCoV-19 is non-inferior to the response to the first ChAdOx1 nCoV-19 booster dose in a subgroup of study participants	GMTR of nAb response to the ancestral strain 28 days after 4th dose administration vs 28 days after a 3rd dose
**Secondary Objectives**	To determine if the humoral immune response to a 4th dose ChAdOx1 nCoV-19 is non-inferior to the response to the first ChAdOx1 nCoV-19 dose in a subgroup of study participants	a) GMTR of nAb response to a contemporary variant of concern 28 days after 4th dose administration vs 28 days after a 3rd dose<br>b) GMTR of Spike binding antibody response to the ancestral strain 28 days after 4th dose administration vs 28 days after a 3rd dose
	To determine if the humoral immune response to a 4th dose ChAdOx1 nCoV-19 is non-inferior to the response to primary series ChAdOx1 nCoV-19 in a subgroup of study participants	a) GMTR of nAb response to the ancestral strain 28 days after 4th dose administration vs 28 days after primary series ChAdOx1 nCoV-19<br>b) GMTR of nAb response to a contemporary variant of concern 28 days after 4th dose administration vs 28 days after primary series ChAdOx1 nCoV-19<br>c) GMTR of Spike binding antibody response to the ancestral strain, variant of concern 28 days after 4th dose administration vs 28 days after primary series ChAdOx1 nCoV-19
	To characterise the cellular immune responses of a 4th dose ChAdOx1 nCoV-19 in seronegative and seropositive participants who previously received an initial ChAdOx1 nCoV-19 boost in a subgroup of participants*****	a) Cellular immune responses by ICS (Th1/Th2) over time<br>b) Cellular immune responses by ELISpot over time

	To evaluate the reactogenicity profile of the fourth dose of ChAdOx1 nCoV-19 vaccine.	a) Occurrence of signs and symptoms of local and systemic reactogenicity requested during 7 days after vaccination (in a subset of 100 participants).<br>b) Recording of unsolicited AEs for 28 days post 4th dose vaccine
**Exploratory objectives**	To explore anti-vector responses to the ChAdOx-1 vector of a 4th dose ChAdOx1 nCoV-19 in seronegative and seropositive participants who previously received an initial ChAdOx1 nCoV-19 boost.	a) Geometric mean titres of ChAdOx1 neutralising antibody titres over time<br>b) Pairwise correlations between anti-S, pseudo-neutralisation, and live neutralisation antibody titres over time
	To further evaluate humoral and cellular immunogenicity across all doses through exploratory immunology	Exploratory immunology such as systems serology profiling

*Detailed assessments of local and systemic reactogenicity for 7 days after vaccination with ChAdOx1 nCoV-19 compared to MenACWY as a control have been documented in a sufficient number of participants in previous studies. In study COV003, detailed local and systemic reactogenicity will be evaluated in 200 randomized participants, who received two doses, a quantity statistically determined to ensure proportionality and comparative representativeness compared to studies COV001 and COV002. Local and systemic reactogenicity will be evaluated for 7 days in a subset of 100 participants after a third or fourth dose of ChAdOx1 nCoV-19.

** Cellular immune responses will be measured in a subset of individuals only (up to 60 volunteers who will be recruited from the CRIE-UNIFESP site, sequentially)

*** The fourth dose will be administered to a subset of 350 participants who have previously received 3 doses of ChAdOx1 nCoV-19 vaccine without prior confirmed COVID-19 infections, in the last four weeks.

**** If the number of participants seronegative at baseline, intra-subject correlation and the observed standard derivation combine to provide ≥ 80% power for non-inferiority assessments, the analysis will be performed as planned. Alternatively, if any factor or combination of factors results in <80% power, the primary analysis will be descriptive

immunogenicity only, with all comparative analyses being exploratory, including any subgroup comparison. The details will be described in the SAP.

***** Cellular immune responses will be measured in a subset of individuals only (up to 40 volunteers).

5 STUDY DESIGN

This is a phase III, controlled, randomized, single-blind study to be conducted in adults with high exposure to COVID-19, who are administered two-doses of ChAdOx1 nCoV-19 or MenACWY and saline placebo by means of an IM injection with co-administered paracetamol.

After reviewing all available data from animal studies and UK studies (COV001 and COV002), participants will be randomized to ChAdOx1 nCoV-19 or MenACWY vaccine in a 1:1 ratio in blocks of 4, and all participants will be blinded to the allocation of the vaccine groups. The DSMB will periodically evaluate safety and efficacy data, every 4-8 weeks and/or as needed. The DSMB will consist of the members of the DSMB currently convened who oversee trials in the UK.

Following the immunogenicity results of the UK phase I/II study which showed higher levels of neutralizing antibodies with a prime-boost schedule (https://doi.org/10.1016/S0140-6736(20)31604-4), a booster dose of vaccine will be offered to all participants in the study.

Participants enrolled on version 4.0 of the protocol onwards will only be allowed to take part in the study if they agree to receive 2 doses of either ChAdOx1 nCoV-19 or MenACWY/saline placebo.

Participants who already received a dose of either ChAdOx1 nCoV-19 or MenACWY (before approvals for the second dose were in place) will be offered a booster dose 4-12 weeks after the prime dose of either ChAdOx1 nCoV-19 or saline placebo, depending on which arm they were originally allocated to. Any volunteers enrolled prior to the booster dose protocol amendment will be able to refuse a second dose and will continue their follow-up as per their previously agreed schedule of attendances.

Participants will be followed for the duration of the study to record adverse events and episodes of symptomatic COVID-19 confirmed by PCR. Participants will be assessed for COVID-19 if they have a new fever (≥ 37.8 ºC) OR cough OR shortness of breath OR anosmia/ageusia.

Moderate and severe COVID-19 disease will be defined by clinical criteria. Detailed clinical parameters will be collected from medical records and aligned with the definitions of moderate and severe disease agreed by the international scientific community, such as a score greater than 6 on the NEWS-2 scale, or a score of 4 and above on ISARIC/WHO (International Severe Acute Respiratory and Emerging Infection Consortium; WHO Working Group on Clinical Characterization and Management of COVID-19). Such parameters include, but are not limited to oxygen saturation, need for oxygen therapy, respiratory rate, and other vital signs, need for ventilatory support, radiographic and computed tomography, and blood test results, among other clinically relevant parameters. Considering that the NEWS-2 scale is not used in the clinical routine of the research site, all staff involved in conducting the project will receive specific training.

From protocol 12.1, by October 31, 2021, a third late dose of ChAdOx1 nCov-19 will be offered to a subgroup of participants who previously received two doses of ChAdOx1 nCov-19 with an interval of 4 to 12 weeks between doses, 11-13 months after the second dose. Receiving the third dose will be optional.

From protocol 13.0, an optional fourth dose of ChAdOx1 nCov-19 will be offered to a subgroup of 350 (+/- 10%) participants who previously received three doses of ChAdOx1 nCov-19 within the trial, randomly selected, and with an interval of 6 to 15 months after the third dose. These participants must also have collected study visits blood samples within study timeframes. For these participants collection of details of COVID-19 disease will be limited to the extent required to determine a score on ISARIC/WHO scale (section 7.4.5) or to meet safety reporting requirements.

NEWS-2 scoring system for serious COVID-19 assessment:

				Score			
**Physiological parameter**	3	2	1	0	1	2	3
**Breathing rate (per minute)**	≤8		9–11	12–20		21–24	≥25
**SpO2 Scale 1 (%)**	≤91	92–93	94–95	≥96			
**SpO2 Scale 2 (%) - use in hypercapnic respiratory failure**	≤83	84–85	86–87	88–92<br>≥93 in the air	93–94 in oxygen	95–96 in oxygen	≥97 in oxygen
**Air or oxygen?**		Oxygen		Air			
**Systolic blood pressure (mmHg)**	≤90	91–100	101–110	111–219			≥220
**Pulse (per minute)**	≤40		41–50	51–90	91–110	111–130	≥131
**Consciousness**				Alert			CVPU
**Temperature (°C)**	≤35.0		35.1–36.0	36.1–38.0	38.1–39.0	≥39.1	

Patient State	Descriptor	Score
Uninfected	Uninfected; no viral RNA detected	0
Ambulatory mild disease	Asymptomatic; viral RNA detected	1
	Symptomatic; independent	2
	Symptomatic; assistance needed	3
Hospitalised: moderate disease	Hospitalised; no oxygen therapy*	4
	Hospitalised; oxygen by mask or nasal prongs	5
Hospitalised: severe diseases	Hospitalised; oxygen by NIV or high flow	6
	Intubation and mechanical ventilation, $pO_2/FiO_2 \geq 150$ or $SpO_2/FiO_2 \geq 200$	7
	Mechanical ventilation $pO_2/FiO_2 < 150$ ($SpO_2/FiO_2 < 200$) or vasopressors	8
	Mechanical ventilation $pO_2/FiO_2 < 150$ and vasopressors, dialysis, or ECMO	9
Dead	Dead	10

WHO clinical progression scale

ECMO=extracorporeal membrane oxygenation. FiO2=fraction of inspired oxygen. NIV=non-invasive ventilation. pO2=partial pressure of oxygen. SpO2=oxygen saturation. *If hospitalised for isolation only, record status as for ambulatory patient.

5.1 Study groups

Vaccine	Number of Participants	Participants
1a) Single dose of ChAdOx1nCOV19 vaccine, $5x10^{10}$ vp + paracetamol	N = up to 1600 participants	Health professionals and adults with high known likely exposure to COVID-19.
1b) Single dose of MenACWY + paracetamol	N = up to 1600 participants	Health professionals and adults with high known likely exposure to COVID-19.
1c) Two doses of ChAdOx1 nCoV-19 vaccine, $5x10^{10}$vp (prime) and 0.5mL boost	N= up to 5150 (up to 1600 invited from 1a to receive a booster dose	Participants recruited in group 1a will be invited to receive a booster dose, 4-12 weeks apart) and new

(3.5 – 6.5 × 10^{10} vp), 4-12 weeks apart + paracetamol	and new volunteers recruited)	participants recruited on version 4.0 of the protocol onwards will consent to receive a 2-dose schedule.
1d) MenACWY prime, and Saline Placebo boost (0.5mL)), 4-12 weeks apart + paracetamol.	N= up to 5150 (up to 1600 invited from 1b to receive a booster dose and new volunteers recruited)	Participants recruited in group 1b will be invited to receive a booster dose, 4-12 weeks apart) and new participants recruited on version 4.0 of the protocol onwards will consent to receive a 2-dose schedule.
1e A third late dose of ChAdOx1 nCov-19 vaccine, 11-13 months after the second dose, + paracetamol.	N= up to 5150 participants (+1%)	Group 1c participants, who received two doses of ChAdOx1 nCov-19 vaccine with an interval of 4 to 12 weeks, 11-13 months after the second dose, by October 31, 2021.
2) A fourth dose of ChAdOx1 nCov-19 vaccine after a late 3rd dose of ChAdOx1 nCov-19 vaccine.	N = 350 (+/- 10%)	Group 1e participants, who have received three doses of ChAdOx1 nCoV-19 with no confirmed COVID-19 diagnosis in the last 4 weeks, and that have collected study visits blood samples within study timeframes (as per protocol).

The overall sample size will be up to **10,300** (with a margin of 1%) participants. All volunteers previously enrolled in groups 1a and 1b will be offered a booster dose. Any new participants recruited into the study on version 4.0 of the protocol onwards will necessarily have to consent to a 2-dose schedule. From Protocol 12.1, all participants of group 1c will be re-consented to the late third dose, by October 31, 2021.

5.2 Study participants

Adult participants over the age of 18 will be enrolled in the study. Participants will be considered enrolled immediately after the vaccine is administered. Recruitment will focus on healthcare professionals and those with likely high known exposure to COVID-19. For example, they are health professionals: students, residents and professionals who perform health care activities such as nurses and nursing technicians, pharmacists, doctors,

physiotherapists, speech therapists and radiology technicians. High exposure adults will be considered: cleaning and hygiene personnel; safety; reception and concierge; volunteers; drivers, among others. Participants in older age groups (56-69 years and 70 years and above) will be recruited at the investigators' discretion. Their likelihood of COVID-19 exposure will be judged on a case-by-case basis, regardless of their previous occupation.

5.3 Definition of the End of Study

The end of study is the date of the last test performed on the last sample collected.

5.4 Potential risks for participants

Potential risks are those associated with phlebotomy, vaccination, and disease potentiation.

Venepuncture

Hematomas and discomfort located at the venepuncture site may occur. More rarely, fainting may occur. These will not be documented as AE if they occur. The total volume of blood drawn during the study period will be approximately 515 mL, excluding any necessary repeated extraction (blood volumes may vary slightly between participants due to the use of different volume vacutainers, the operational procedures of the research sites and the number of symptomatic visits). Participants will be asked to refrain from donating blood during the period of their involvement in the study.

Allergic reactions

Mild to severe allergic reactions can occur in response to any component of a drug preparation. Anaphylaxis is extremely rare (about 1 in 1,000,000 doses of vaccine), but it can occur in response to any vaccine or medication.

Vaccination

Based on pooled clinical data from studies with ChAdOx1 nCoV-19, the most commonly expected local solicited AEs for participants in this study are vaccination site pain and tenderness. The most commonly reported local, solicited AEs were vaccination site pain and tenderness. The most commonly reported systemic solicited AEs were fatigue, headache, malaise, and myalgia. The majority of reported events have been mild or moderate in severity and resolved within 1 to 7 days. When compared with the first dose, adverse reactions reported after the second dose were milder and reported less frequently.

Post-authorisation hypersensitivity reactions, including anaphylaxis and angioedema, have occurred following administration of ChAdOx1 nCoV-19 and are considered an identified risk.

Important identified risk: A very rare and serious combination of thrombosis and thrombocytopenia, including TTS, in some cases accompanied by bleeding, has been observed following vaccination with ChAdOx1 nCoV-19 during post-authorisation use. This includes cases presenting as venous thrombosis, including unusual sites such as cerebral venous sinus thrombosis, splanchnic vein thrombosis, as well as arterial thrombosis, concomitant with thrombocytopenia. The majority of the events occurred within the first 21 days following vaccination and some events had a fatal outcome. The reporting rates after the second dose are lower compared to the first dose. No events have been observed in the clinical trials. A causal relationship between these events and ChAdOx1 nCoV-19 is considered plausible. The exact mechanism and/or pathophysiology of thrombosis in combination with thrombocytopenia following immunisation is unknown. Although specific risk factors for thromboembolism in combination with thrombocytopenia have not been identified, cases have occurred in patients with a previous history of thrombosis, as well as in patients with autoimmune disorders, including immune thrombocytopenia. The benefits and risks of vaccination should be considered in these patients.

Important potential risks:

Neurologic events and potential immune-mediated neurologic conditions: demyelinating diseases

Vaccine-associated enhanced disease including vaccine-associated enhanced respiratory disease

Cerebrovascular venous and sinus thrombosis without thrombocytopenia

Disease Enhancement

The risks of inducing disease enhancement and pulmonary immunopathology in case of COVID-19 disease after vaccination with ChAdOx1 nCoV-19 are unknown. Challenge studies on ferrets and PNH are ongoing and the results will be reviewed as they arise. Two studies on PNH so far have shown no evidence of disease potentiation until day 7 after the challenge. All preclinical data from challenge studies using ChAdOx1 nCoV-19 and other candidate vaccines (where available) will guide risk/benefit decisions for participants who receive the IMP. Any safety signs associated with the enhancement of the disease potentially observed in COV001/COV002 will also guide these decisions.

5.5 Known potential benefits

Participants enrolled in the control groups will receive MenACWY, a licensed vaccine that has been administered to adolescents on routine vaccination schedules in several countries, including the United Kingdom, and is used as a travel vaccine for high-risk areas. Most of the

participants in this study will not have received this vaccine before and therefore will gain the benefit of protection against meningococci in groups A, C, W and Y. Participants who had previously been vaccinated with MenACWY will have their immunity enhanced against these organisms and are not exposed to additional risks when receiving the additional dose of the comparator vaccine.

If the effectiveness of the vaccine against COVID-19 is proven, after the analysis of the effectiveness results and approval by the Data and Safety Monitoring Committee, the sponsor will make this vaccine available to the research participants who received the control vaccine, MenACWY.

4th dose potential benefits

Participants receiving the 4th vaccination dose might benefit from the potential benefit of added protection.

In addition, study participants will be informed about antibody (IgG) levels against SARS-CoV-2 after the 3rd dose and after the 4th booster dose (as applicable). This is an expectation of the population that participates in clinical trials in Brazil, as part of their participation in such studies.

6 RECRUITMENT AND WITHDRAWAL OF STUDY PARTICIPANTS

6.1 Identification of Participants

Participants can be recruited through advertisements approved by the local Ethics Committee. The leaflets will be distributed, including the name of the study, information from the centers, age group, disease, and vaccine.

6.2 Informed Consent Form

The participant will sign and date personally or electronically the last approved version of the Informed Consent Form. When the process is carried out in person, it will be presented to the participants, individually, a written version and verbal explanation of the Informed Consent Form. When the process is carried out electronically, participants will individually receive a link to access the electronic version of the Informed Consent Form. In both cases, it will be detailed:

- the exact nature of the study;
- what it will imply for the participant;
- the implications and restrictions of the protocol;
- the known side effects and any risks involved in participating;

- sample manipulation - participants will be informed about the samples that will be collected anonymously during the course of the study and that can be shared with the study collaborators;
- that individual results will not be shared with participants.

The Informed Consent Form will be made available to the participant before obtaining consent. However, participants will have the opportunity to individually question a properly trained and delegated researcher before signing the consent.

The following general principles will be emphasized:

- Participation in the study is completely voluntary;
- Refusal to participate does not involve a penalty or loss of medical benefits;
- The participant can withdraw from the study at any time;
- The participant is free to ask questions at any time to understand the purpose of the study and the procedures involved;
- The study involves researching an investigational vaccine;
- There is no direct benefit expected for the participant;
- The general practitioner/personal physician of the participant can be contacted to corroborate his/her medical history. Written or verbal information about the participant's medical history can be requested from the general practitioner/personal physician or other sources;
- Blood samples taken as part of the study can be sent abroad, to the United Kingdom, to laboratories at the University of Oxford. These will not be identified. Participants will be asked whether they agree to biorepository storage for future use, in Brazil or abroad, but this will be optional.

The participant will have as much time as he/she wishes to consider the information and the opportunity to question the Investigator, his/her clinical physician or other independent parties to decide whether to participate in the study. The electronic Informed Consent Form must be signed and dated electronically by the adult participant, and the printed Informed Consent Form must be signed and dated by the adult participant and by the person responsible for obtaining the informed consent. This person must be suitably qualified and experienced, have been authorized to do so by the Lead/Principal Investigator and be listed on the delegation´s record. In the case of the printed Informed Consent Form, a copy of the signed document will be given to the participant. The signed original document will be retained at the research study sites.

6.3 Inclusion and exclusion criteria

This study will be performed in healthy adults, who meet the following inclusion and exclusion criteria:

6.3.1 Inclusion Criteria

The participant must meet all the following criteria to be eligible for the study:

- Adults from 18 to 55 years of age.
- Adults aged 56-69 years old (after review of safety data by DSMB in this age group in the UK trial)
- Adults aged 70 and above years old (after review of safety data by DSMB in this age group in the UK trial)
- Able and willing (in the Investigator's opinion) to fulfil all study requirements;
- Health professionals and/or adults at high risk of exposure to SARS-CoV-2, as defined in section 5.2 of this protocol;
- Serology with SARS-CoV-2 negative IgG antibodies; This inclusion criteria does not apply to participants enrolled from version 4.0 of the protocol onwards.
- Willing to allow investigators to discuss the participant’s clinical history with their GP/personal physician and access medical records relevant to the study procedures;
- Only for women of childbearing age: willing to practice continuous effective birth control (see below) during the study, and a negative pregnancy test on the screening and vaccination day(s);
- Consent to abstain from blood donation during the course of the study;
- Provide informed consent in writing.

Additionally for group 2

- Participants that received their third dose ChAdOx1 nCoV-19 in the trial
- Study visits blood samples for visits to date must have been collected within visit windows, i.e., participants who had their PB28 visit bloods within 28 – 35 days post vaccine, and who had their 28 days post third dose blood within 28-35 days post third vaccine.

The DSMB reviewed safety data from volunteers aged 56 and above recruited as part of the COV002 UK study before recruitment of older adults was allowed.

6.3.2 Exclusion Criteria

The participant will not be eligible for the study if any of the following criteria apply:

- Participation in trials of prophylactic drugs for COVID-19 during the course of the study;

  Note: Participation in COVID-19 treatment trials is permitted in case of hospitalization due to COVID-19, after confirmation of positive PCR. The study team should be informed as soon as possible. Participants with COVID-19 not hospitalized with positive PCR results for COVID-19 may be medicated according to standard clinical practice, however, participation in treatment trials will not be allowed.

- Planned receipt of any vaccine (authorized or investigational), within 30 days before and after vaccination with the exception of the licensed seasonal influenza vaccination and the licensed pneumococcal vaccination. Participants will be encouraged to receive these vaccinations at least 14 days before or after their study vaccine;
- Prior receipt of an investigational or licensed vaccine with the possibility of impacting the interpretation of the study data (for example, Adenovirus vector vaccines, any vaccines against coronavirus);
- Administration of immunoglobulins and/or any blood products in the three months prior to the planned administration of the candidate vaccine;
- Any confirmed or suspected immunosuppressive or immunodeficiency state, including HIV (regardless of treatment, CD4 count or viral load status); asplenia; severe recurrent infections and chronic use (more than 14 days) of immunosuppressive medication in the last 6 months, except for topical steroids or short-term oral steroids (cycle lasting ≤14 days);
- History of allergic disease or reactions possibly exacerbated by any component of ChAdOx1 nCoV-19 or MenACWY or paracetamol;
- Any history of angioedema;
- Any history of anaphylaxis;
- Pregnancy, lactation or willingness/intention to become pregnant during the study;
- Current diagnosis or treatment for cancer (except basal cell carcinoma of the skin and cervical carcinoma in situ);
- History of severe psychiatric illness that possibly affects your participation in the study;

- Hemorrhagic disorder (for example, factor deficiency, coagulopathy or platelet disorder), or a previous history of significant bleeding or bruising after IM injections or venipuncture;
- Current suspected or known dependence on alcohol or drugs;
- Severe and/or uncontrolled cardiovascular diseases, respiratory diseases, gastrointestinal diseases, liver disease, kidney disease, endocrine disorder, and neurological disease (mild/moderate well-controlled comorbidities are allowed);
- History of COVID-19 confirmed by laboratory (serology, rapid tests based on antigen or antibody or PCR);
- Seropositive for antibodies to SARS-CoV-2 before recruitment; This exclusion criteria does not apply to participants enrolled from version 4.0 of the protocol onwards
- Continued use of anticoagulants, such as coumarins and related anticoagulants (for example, warfarin) or new oral anticoagulants (for example, apixaban, rivaroxaban, dabigatran and edoxaban);
- Any other significant illness, disorder or finding that may significantly increase the risk for the participant, affect his/her ability to participate in the study or impair the interpretation of the study data.

6.3.3 Re-vaccination exclusion criteria (two-dose groups only)

The following AEs associated with any vaccine or identified on or before the day of vaccination constitute absolute contraindications to further administration of an IMP to the volunteer in question. If any of these events occur during the study, the subject will not be eligible to receive a booster dose and will be followed up by the clinical team or their regular doctor until resolution or stabilization of the event:

- Anaphylactic reaction following administration of vaccine
- Pregnancy – if the outcome of pregnancy is termination or miscarriage, volunteers can be boosted if appropriate to do so and given they have a negative pregnancy test at the time of boosting.
- Any AE that in the opinion of the Investigator may affect the safety of the participant or the interpretation of the study results
- Participants who develop COVID-19 symptoms and have a positive PCR test after the first vaccination can only receive a booster dose after a minimum 4 weeks interval from their first PCR positive test, provided their symptoms have significantly improved. The decision to proceed with booster vaccinations in those cases will be at clinical discretion of the investigators. For participants who are

asymptomatic and have a positive PCR test, a minimum of 2 weeks from first PCR positivity will be required before boosting

6.3.4 Exclusion Criteria for the third dose

The following will not be eligible to receive a late third dose:

- experienced, at any time, infection with SARS CoV2 with a positive PCR test;
- those who received a COVID-19 vaccine other than ChAdOx1 nCoV-19.

6.3.5 Exclusion Criteria for the fourth dose

The following will not be eligible to receive a ChAdOx1 nCoV-19 fourth dose:

- Self-reported confirmed COVID-19 infection, through PCR or lateral flow test in the last four weeks;
- those who received additional COVID-19 vaccines outside of the trial.
- Pregnancy
- Allergy or other contraindication to vaccination with ChAdOx1 nCoV-19
- History of Guillain-Barré syndrome
- Any confirmed or suspected immunosuppressive or immunodeficient state, including Asplenia
- History of clinically significant thrombocytopenia and/or thrombosis or clinically significant bleeding (eg, factor deficiency, coagulopathy, or platelet disorder), or prior history of significant bleeding or bruising following intramuscular injections or venepuncture, including Capillary Leakage Syndrome.
- Major venous and/or arterial thrombosis in combination with thrombocytopenia following vaccination with any Covid-19 vaccine
- Severe and/or uncontrolled cardiovascular disease, respiratory disease, gastrointestinal disease, liver disease, renal disease, endocrine disorder, and neurological illness, as judged by the Investigator (mild/ moderate well-controlled comorbidities are allowed
- Plans to move outside the study area

6.3.6 Effective birth control for volunteers

Participants of childbearing potential must use an effective form of birth control during the 12 months of study, or extended for one more month for those who choose to receive the third or fourth dose.

Acceptable forms of birth control for participants include:

- Established use of oral, injected or implanted hormonal of birth control methods;

- Placement of an intrauterine device (IUD) or intrauterine system (IUS);
- Complete hysterectomy;
- Bilateral occlusion of the tubes;
- Contraceptive barrier methods (condom or occlusive tampon with spermicide);
- Male sterilization, if the vasectomized partner is the participant's only sexual partner;
- True abstinence, when it is in line with the subject's preferred and usual lifestyle (periodic abstinence and withdrawal are not acceptable of birth control methods).

6.3.7 Withdrawal of participants

In accordance with the principles of the current version of the Declaration of Helsinki and any other applicable regulations, the participant has the right to withdraw from the study at any time and for any reason and is not required to give his/her reasons for doing so. The Investigator may withdraw the participant at any time for the sake of his/her health and well-being. In addition, the participant can withdraw/be withdrawn for any of the following reasons:

- Investigator's Decision;
- Ineligibility (both during the study and retrospectively, having been omitted in the screening);
- Significant deviation from the protocol;
- Non-adherence of the participant to the study requirements;
- An AE, which requires discontinuing participation in the study or resulting in an inability to continue to comply with the study procedures.

The reason for the withdrawal will be registered with the CRF. If the withdrawal is a consequence of an AE, appropriate follow-up visits and/or medical care will be scheduled, with the consent of the participant, until the AE is resolved, stabilized or a causality unrelated to his/her participation in the study has been attributed. Any participant who is withdrawn from the study may be replaced, if this is possible within the specified period. The DSMB or DSMB president may recommend withdrawing participants.

If a participant withdraws from the study, the data collected before their withdrawal will still be used in the analysis. The storage of blood samples will continue unless the participant specifically requests otherwise.

In all cases of withdrawal from the subject, the collection of long-term safety data, including some procedures such as a safety blood test, will continue as appropriate if individuals have received one or more doses of the vaccine, unless they refuse any additional follow-up

6.4 Pregnancy

If a participant becomes pregnant during the trial, she will be followed up for clinical safety assessment with her continuous consent and, in addition, she will be followed up until the outcome of the pregnancy is determined. We will not routinely perform venipuncture on a pregnant participant unless there is a clinical need. In addition, full and free follow-up and assistance will be ensured, for as long as necessary for: (a) the participants who become pregnant, and (b) the fetus, if applicable.

7 CLINICAL PROCEDURES

This section describes the clinical procedures for evaluating study participants and following up after administering the study vaccine.

7.1 Visit Schedule

All participants will have visits and clinical procedures as indicated in the visit schedule below table 4. The subjects will receive the ChAdOx1 nCoV-19 or MenACWY vaccine/saline solution placebo and will be followed up for a total of 12 months post second dose. Additional visits or procedures may be performed at the investigators' discretion, for example, medical history and additional physical examination, or additional blood tests, if they are clinically relevant.

All participants in groups 1a and 1b will be offered a booster dose of vaccine, however if a participant declines the booster dose, they will continue in group 1a or 1b and follow the planned visit schedule, as per table 4.

From Protocol 12.1, a third late dose of ChAdOx1 nCoV-19 vaccine will be offered to group 1c participants, 11-13 months after receiving the second dose. Those, who choose to receive the third dose, will constitute group 1e. They will be followed up with one more visit, with a total up to 13 months of follow-up after the second dose.

From protocol version 13.0 an optional fourth dose of ChAdOx1 nCoV-19 vaccine will be offered to up to 350 (+/- 10%) participants who have already received 3 doses of ChAdOx1 nCoV-19 in the trial, randomly selected. These participants will have further visits at days 28 and 180 post vaccine, and a telephone call on Day 7.

7.2 Observations, medical history and physical examination

Temperature will be routinely measured at the time-points indicated in the schedule of procedures. Respiratory rate, oxygen saturation, pulse, blood pressure and temperature will be measured at the COVID-19 testing visits and if clinically required. All subjects will undergo medical history and a targeted physical examination if considered necessary at screening or pre-enrolment on D0. The purpose of this examination is to assess and document the subject's baseline health status so that any later change can be determined. Vital signs (temperature, heart rate, respiratory rate, blood pressure +/- oxygen saturation), height and weight will be measured at screening or pre-enrolment on D0 as part of baseline assessments. Further medical history, physical examination and observations may be done throughout the study based on clinical discretion.

4th dose participants:

Medical history for 4th dose participants will be updated to capture any changes not captured in the original medical history/AEs.

7.3 Blood samples, nose/throat swabs and urine analysis

- **PCR process for COVID-19.** A nose and throat swab will be collected for COVID-19 PCR.
- **SARS-CoV-2 viral genome sequencing.** Participant swab samples that are determined to be SARS-CoV-2 PCR positive may also subsequently undergo further analysis to determine SARS-CoV-2 viral genomes using RNA sequencing. Selection of swab samples for sequencing and the proportion of samples to be sequenced will be at Investigator's and or sponsor's discretion.
- **Immunology.** Immunogenicity will be assessed using a variety of immunological assays. This may include antibodies to SARS-CoV-Spike (ancestral and variant strains) and non-Spike antigens by binding assays, ex vivo ELISpot assays for interferon gamma and flow cytometry assays, neutralization (pseudoneutralisation and live neutralisation) and other functional antibody assays and B cell analyses. Other exploratory immunological assays, including cytokine analysis and other antibody assays, among others, may be performed at the Investigators' discretion. Immunology samples for the assays described above will be drawn as per the schedule of attendances below (table 4)
- **Urine analysis.** In the case of participants of childbearing age, urine will be tested for beta-human chorionic gonadotropin (β-HCG) at screening (when applicable) and immediately before vaccination.

Collaboration with other specialized laboratories in the UK, Europe and outside of Europe may take place for new exploratory tests and for some of the immunology testing described above. This would involve transferring serum, or plasma, PBMC and/or other study samples to these laboratories, but these would remain anonymous. For this, after evaluation and prior approval of the REC/CONEP system, the participant will be presented with a new Informed Consent Form. Only after obtaining this new consent form can the samples be used for purposes other than those specified in this protocol.

Immunological and sequencing tests will be performed according to the standard operating procedures of the research sites, the University of Oxford, and collaborating international and national laboratories.

Subjects will be informed that their blood sample (after all tests for this study are completed), will be stored in a biorepository for future use. The subjects will be able to decide whether to allow such future use of any sample. With the informed consent of the participants, blood serum and/or PBMCs will be frozen for future analysis of COVID-19 and responses related to the vaccine. If a subject chooses not to allow this, no sample will be stored beyond the storage period required to meet Good Laboratory Practices (GLP) and regulatory requirements.

7.4 Study visits

Study visits and procedures will be performed by the research sites staff. The procedures to be included in each visit are documented in the visit schedule (table 4). Each visit is assigned a time and window period, within which the visit will take place.

7.4.1 Screening visit and recruitment

All potential participants will have a screening visit, up to 7 days before vaccination for a baseline assessment. For participants who are recruited in the study before version 4.0 of the study protocol, a serological evaluation of baseline antibodies against SARS-CoV-2 is performed. The results of the serology should be available within this period, no later than 7 days after collection. Volunteers with negative serology for IgG antibodies to SARS-CoV-2 may participate in the study (applicable to previous protocol versions only and not from version 4.0 onwards).

Having established that there is a low baseline seropositivity in the study population, the remaining participants can be included without baseline SARS-CoV-2 antibodies. This allows the screening visit to take place at the same day as the vaccination visit and will precede vaccination procedure.

At the screening visit, the objectives of the study and all tests to be performed will be described to the participants. Individually, each participant will have the opportunity to question a duly

trained and delegated researcher before signing the consent. The informed consent procedure will be performed before screening/recruitment procedures, as described in section 6.2. A medical history, including previous vaccinations, and targeted physical examination (when necessary) will be conducted at the screening visit. Findings will be recorded as part of baseline and eligibility assessment. A screening visit can be repeated if time from screening to vaccination is greater than the pre-specified window in the study protocol.

The research site staff may contact the subject's general practitioner/personal physician with written permission after screening to corroborate the clinical history when possible and practical to do so. The participant's doctor may be notified that the subject has been enrolled voluntarily in the study.

7.4.2 Day 0: Recruitment and vaccination visit

Participants will be considered enrolled in the trial at the time of vaccination. Before the investigational vaccination/treatment, the eligibility of the participant will be reviewed. The temperature and, if necessary, a medical history and physical examination will be performed to determine the need to postpone vaccination. The study vaccines/treatment will be administered as described below.

7.4.2.1 Vaccination

All vaccines will be administered intramuscularly according to specific standard operating procedures. The injection site will be covered with a sterile dressing and the participant will remain at the study site for observation, in case of immediate adverse events. Observations will be made at a minimum of 15 minutes after vaccination, the sterile dressing will be removed and the injection site inspected.

A sub sample of 200 participants will receive a thermometer, a metric ruler, access to the electronic diary via web, a printed diary (for use in case the electronic diary presents problems), guidelines and instructions for use, together with a contact card containing the number of 24-hour emergency telephone number to contact the research site if necessary. Participants will be instructed on how to self-assess the intensity and severity of requested adverse events (Table 3 - Requested and unsolicited AEs). There will also be space in the electronic (or paper) Symptom Diary for the participant to document unsolicited AEs for 28 days, and if a medication has been taken to relieve the symptoms. The diaries will collect information about the timing and severity of the following solicited AEs. Participants who were asked to fill out a diary post prime vaccination will be asked to fill out the same diary post booster vaccination.

For a subset of 100 participants, they will be asked to fill in a 7-day diary after the third dose, preferably for those who have already filled it out previously.

For those receiving the 4th dose, a subset of 100 participants will be asked to fill in a 7-day diary after the vaccination. Where possible these will be the same participants that have completed a diary after the third dose.

Table 3. Spontaneous, requested AEs collected in the post-vaccination electronic diary (or daily card) for reactogenicity

AE spontaneous locations	AE systemic spontaneous
Pain	Fever
Sensitivity	Feeling feverish
Redness	Chills
Heat	Joint pain
Itching	Muscle ache
Swelling	Fatigue
Local hardening	Headache
	Malaise
	Nausea
	Vomiting

The use of electronic diaries allows the real-time monitoring of the safety of the study vaccines, however paper back up can be used if electronic diaries are not available.

7.4.3 Later visits

Follow-up visits will take place according to the visit schedule described in table 4, with their respective windows.

If the participants experience adverse events (laboratory or clinical), for which the investigator (doctor) and/or DSMB president requires more rigorous observation, the participant may be admitted to the hospital for observation and subsequent medical treatment under the care of the hospitalization team.

Table 4 Visiting schedule for participants

Groups 1a and 1b

Visit number	Screenin g	1	2	3	4	5	Test for COVID-19 (S0)	(Phone follow up only) COVID-19 PCR positive + 7 days (S7)	Unblinding:<br>• Can be done as a separate event, face to face and online;<br>• Can be done at the site, followed by Control Group vaccination;	Booster dose for controls post unblinding
Chronology** (days)	-7	0	28	90	182	364	As required	7 days after positive PCR for COVID-19	Vaccine availability/ Volunteers visit schedule/ Local approvals	4-12 weeks post prime dose
Time window (days)	±7		-7/+14	±7	±14	±30	N/A	±2	N/A	N/A
Informed consent/Unblinding Consent	X								X	
Review of contraindications, inclusion and exclusion criteria		X								
Vaccination		X							X (subjects who received the control vaccine or subjects who received a single dose of ChAdOx1 nCoV-19, if they agree to a booster dose at least 4 weeks from prime)	X

Vital signs	X	X	X	X	X	X	(X)		X (Temperature only)	X (Temperature only)
Ascertainment of adverse events		X	X	X	X	X	X	X	X	X
COVID Hospital Admission Medical Records Review and Data Collection							If COVID-19 case results in Hospital Admission			
Electronic Diaries of Vaccine Symptoms $ (subset of volunteers only)		X	X							
Clinical history, physical examination		(X)	(X)	(X)	(X)	(X)	(X)	(X)	(X)	(X)
Biochemistry, hematology (mL)							(5)			
Exploratory immunology (mL)		Up to 50mL	Up to 50mL	Up to 50mL	Up to 50mL	Up to 50mL	10		Up to 50mL (controls receiving ChAdOx1 nCoV-19 only)	Up to 50mL (controls receiving ChAdOx1 nCoV-19 only)
Nose and Throat Swab							X			
urinary bHCG (women only)	X	X							X	X
Blood volume per visit		50	50	50	50	10	15			
Accumulated blood volume %		50	100	150	200	250	265	265	315	365

(X) = if deemed necessary ^ = Vital signs at screening or pre-enrolment assessment on D0 include pulse, blood pressure, temperature, respiratory rate +/- oxygen saturation. Only temperature will be routinely measured at subsequent follow-up visits. At COVID-19 testing visits a full set of observations will only be taken if deemed clinically necessary ** Chronology is approximate only. The exact times of the visits refer to the day of recruitment, that is, each visit must take place within the indicated interval of days after recruitment ± time window. % Accumulated blood volume for participants if blood is drawn according to schedule and excluding any repeat safety blood tests that may be required. $ Vaccine reactogenicity diaries are applicable to a subset of participants only.

NB Participants who refuse a booster vaccination should be followed-up as per the schedule of attendances above.

Group 1c and 1d

Attendance Number (boost)	Screening	1 (prime)	2 (booster)	3	4	5	6	COVID-19 Testing (S0)	(Phone follow up only) COVD-19 Positive PCR + 7 days (S7)	Unblinding<br>• Can be done as a separate event, face to face and online;<br>Can be done at the site, followed by Control Group vaccination;	Booster dose for controls post unblinding
Timeline** (days)	-7	0	4-12 weeks post prime	28 post booster	90 post booster	182 post booster	364 post booster	As required	7 days post COVID-19 PCR positive	Vaccine availability/ Volunteers visit schedule/ Local approvals	4-12 weeks post prime following the national immunization plan
Time window (days)	±7		+14	+7	±14	±14	±30	N/A	±2	N/A	
Informed Consent/Unblinding Consent	X		X[b]							X	
Review contraindications, inclusion and exclusion criteria		X	X								
Vaccination		X	X							X (subjects who received the control vaccine or subjects who received a single dose of ChAdOx1 nCoV-19, if they agree to a booster dose at least 4 weeks from prime)	X

Attendance Number (boost)	Screening	1 (prime)	2 (booster)	3	4	5	6	COVID-19 Testing (S0)	(Phone follow up only) COVD-19 Positive PCR + 7 days (S7)	Unblinding<br>• Can be done as a separate event, face to face and online;<br>Can be done at the site, followed by Control Group vaccination;	Booster dose for controls post unblinding
Vital signs^	X	X	(X)	(X)	(X)	(X)	(X)	(X)		X (Temperature only)	X (Temperature only)
Ascertainment of adverse events		X	X	X	X	X	X	X	X	X	X
COVID Hospital Admission Medical Records Review and Data Collection								If COVID-19 case results in Hospital Admission			
Electronic Diaries of Vaccine Symptoms $<br>(subset of volunteers only)		X	X								
Medical History / Physical Examination		(X)	(X)	(X)	(X)	(X)	(X)	(X)	(X)		
Biochemistry, Haematology (mL)								(5)			
Exploratory immunology (mL)		Up to 50mL	Up to 50mL	up to 50	up to 50	up to 50	up to 50	up to 10		Up to 50mL (controls receiving ChAdOx1 nCoV-19 only)	Up to 50mL (controls receiving ChAdOx1 nCoV-19 only)

Attendance Number (boost)	Screening	1 (prime)	2 (booster)	3	4	5	6	COVID-19 Testing (S0)	(Phone follow up only) COVD-19 Positive PCR + 7 days (S7)	Unblinding • Can be done as a separate event, face to face and online; Can be done at the site, followed by Control Group vaccination;	Booster dose for controls post unblinding
Nose and Throat Swab								X			
Urinary bHCG (women of childbearing potential only)	X	X	X							X	X
Blood volume per visit		50	50	50	50	50	50	up to 15		Up to 50	Up to 50
Cumulative blood volume (post boost)%		50	100	150	200	250	300	315	315	365	415

(X) = if deemed necessary ^ = Vital signs at screening or pre-enrolment assessment on D0 include pulse, blood pressure, temperature, respiratory rate +/- oxygen saturation. Only temperature will be routinely measured at subsequent follow-up visits. At COVID-19 testing visits a full set of observations will only be taken if deemed clinically necessary** Chronology is approximate only. The exact times of the visits refer to the day of recruitment, that is, each visit must take place within the indicated interval of days after recruitment ± time window. % Accumulated blood volume for participants if blood is drawn according to schedule and excluding any repeat safety blood tests that may be required. $ Vaccine reactogenicity diaries are applicable to a subset of participants only [b] New PIS/ICF only for participants enrolled before protocol 4.0 from groups 1c and 1d

NB Participants who accept to take part in booster dose subgroup will have their schedule of attendances replaced by above schedule. Participants who already attended their D28 visit post prime will be asked to attend a separate visit for their booster.

Group 1e

Attendance Number (boost)	Screening	1 (prime)	2 (booster)	3	4	5	6	7	COVID-19 Testing (W0)	(Phone follow up only) COVID-19 Positive PCR + 7 days (W7)	Unblinding<br>• Can be done as a separate event, face to face and online;<br>Can be done at the site, followed by Control Group vaccination;	Booster dose for controls post unblinding
Timeline** (days)	-7	0	4-12 weeks post prime	28 post booster	90 post booster	182 post booster	364 post booster/third dose - booster	28 after third dose	As required	7 days post COVID-19 PCR positive	Vaccine availability/ Volunteers visit schedule/Local approvals	4-12 weeks post prime following the national immunization plan
Time window (days)	±7		+14	+7	±14	±14	±30	+7	N/A	±2	N/A	
Informed Consent/Unblinding Consent	X		X[b]				X[c]				X	
Review contraindications, inclusion and exclusion criteria		X	X				X					
Vaccination		X	X				X				X (subjects who received the control vaccine or subjects who received a single dose of ChAdOx1 nCoV-19, if they agree to a booster	X

Attendance Number (boost)	Screening	1 (prime)	2 (booster)	3	4	5	6	7	COVID-19 Testing (W0)	(Phone follow up only) COVID-19 Positive PCR + 7 days (W7)	Unblinding •Can be done as a separate event, face to face and online; Can be done at the site, followed by Control Group vaccination;	Booster dose for controls post unblinding
											dose at least 4 weeks from prime)	
Vital signs^	X	X	(X)	(X)	(X)	(X)	(X)	(X)	(X)		X (Temperature only)	X (Temperature only)
Ascertainment of adverse events		X	X	X	X	X	X	X	X	X	X	X
COVID Hospital Admission Medical Records Review and Data Collection									If COVID-19 case results in Hospital Admission			
Electronic Diaries of Vaccine Symptoms $ (subset of volunteers only)		X	X				X	X				
Medical History/Physical Examination		(X)	(X)	(X)	(X)	(X)	(X)	(X)	(X)	(X)		
Biochemistry, Haematology (mL)									(5)			
Exploratory immunology (mL)		Up to 50 mL	Up to 50 mL	Up to 50	Up to 50	Up to 50	Up to 50	Up to 50	Up to 10		Up to 50mL (controls receiving	Up to 50mL (controls receiving

Attendance Number (boost)	Screening	1 (prime)	2 (booster)	3	4	5	6	7	COVID-19 Testing (W0)	(Phone follow up only) COVID-19 Positive PCR + 7 days (W7)	Unblinding • Can be done as a separate event, face to face and online; Can be done at the site, followed by Control Group vaccination;	Booster dose for controls post unblinding
											ChAdOx1 nCoV-19 only)	ChAdOx1 nCoV-19 only)
Nose and Throat Swab									X			
Urinary bHCG (women of childbearing potential only)	X	X	X				X				X	X
Blood volume per visit		50	50	50	50	50	50	50	Up to 15		Up to 50	Up to 50
Cumulative blood volume (post boost)%		50	100	150	200	250	300	350	345	345	365	415

(X) = if deemed necessary ^ = Vital signs at screening or pre-enrolment assessment on D0 include pulse, blood pressure, temperature, respiratory rate +/- oxygen saturation. Only temperature will be routinely measured at subsequent follow-up visits. At COVID-19 testing visits, a full set of observations will only be taken if deemed clinically necessary** Chronology is approximate only. The exact times of the visits refer to the day of recruitment, that is, each visit must take place within the indicated interval of days after recruitment ± time window. % Accumulated blood volume for participants if blood is drawn according to the schedule, excluding any repeat safety blood tests that may be required. $ Vaccine reactogenicity diaries are applicable to a subset of participants only [b] New ICF only for participants enrolled before protocol version 4.0 from groups 1c and 1d. [c] New ICF for all participants with inclusion of the third dose.

Note: Participants who accept to take part in booster dose subgroup will have their schedule of attendances replaced with the above schedule. Participants who already attended their D28 visit post prime will be asked to attend a separate visit for their booster.

Group 2

Attendance Number (boost)	8<br>4th dose vaccination	Phone call/email contact	9	10
Timeline<br>(days)	At final study visit or at extra visit	7 days post 4th dose	28 days post 4th dose	180 days post 4th dose
Time window (days)		+3	+ 14	+/- 28
Informed Consent	X[b]			
Review contraindications, inclusion and exclusion criteria	X			
Vaccination	X			
Vital signs^	(X)			
Ascertainment of adverse events*		X	X	X
Details of any COVID-19 diagnosis	X	X	X	X
Electronic Diaries of Vaccine Symptoms $<br>(subset of volunteers only)	X	X		
Medical History/Physical Examination	(X)	(X)	(X)	(X)
Exploratory immunology (mL)	Up to 50 mL		Up to 50 mL	Up to 50 mL
IgG against SARS-CoV-2	X		X	X
Urinary bHCG (women of childbearing potential only)	X			
Blood volume per visit	Up to 55mls		Up to 55mls	Up to 55mls
Cumulative blood volume (post 4th dose)%	405		460	515

(X) = if deemed necessary ^ = Vital signs: only temperature will be routinely measured at Visit 08. Chronology is approximate only. The exact times of the visits is relative to the date of administration of 4th dose, that is, each visit must take place within the indicated interval of days after 4th dose ± time window. % Accumulated blood volume for participants if blood is drawn according to the schedule, excluding any safety blood tests that may be required. $ Vaccine reactogenicity diaries are applicable to a subset of participants only [b] New ICF for all participants with inclusion of the fourth dose. * Unsolicited AEs (that may not meet SAE or AESI criteria) will be collected by the investigator during Visit 9 Day 28 post 4th dose. SAE and Adverse Events of Special Interest will be collected throughout the study period.

7.4.4 Symptomatic participants from groups 1a, 1b, 1c, 1d and 1e

Participants who become symptomatic during follow-up will be instructed to call the study team, who will then advise on how to proceed with clinical trials for COVID-19, if necessary, according to the trial work instructions. If a participant is symptomatic, COVID-19 testing should take place from enrolment onwards, regardless of time elapsed from vaccination to symptom onset. An isolated fever ≥37.8^{0}C is an indication for COVID-19 testing, unless this isolated fever has occurred within 48 hours of vaccination. If fever persists beyond 48 hours post-vaccination, the patient will then be eligible for COVID-19 testing. Participants will receive weekly reminders (for example, text messages - SMS or Whatsapp, surveillance APPs notifications, email or telephone contacts) to contact the study team if they experience fever or cough or shortness of breath or anosmia/ageusia and if they are hospitalized for any reason.

7.4.4.1 Diagnostic SARS-CoV-2 PCR testing outside of the trial

In a (primary endpoint symptom) symptomatic illness episode, if a participant has already received a negative SARS-CoV-2 test result via a validated PCR assay that was performed after symptom onset and where this test was carried out outside of the study, they will now (as of protocol V11) be considered to be a negative case without requiring further PCR testing by the study team. In such instances, documentation relating to the result should be acquired by investigators and filed as part of the individual participant record. Additionally, if investigators believe a symptomatic participant can obtain their initial diagnostic SARS-CoV-2 PCR test more rapidly outside of the study (for logistical or other reasons) they may advise participants be tested via that route e.g. by being advised to arrange a test through a local testing clinic or similar service. Where symptomatic participants report a SARS-CoV-2 test outside of the trial but are unable to provide details of the assay and/or documentation of the test result, they will be asked to attend the trial site for a COVID-19 testing visit.

7.4.4.2 COVID-19 testing visit

Participants will be invited for a COVID-19 testing visit in two circumstances:

- They report primary endpoint symptoms AND have not already had a documented negative SARS-CoV-2 PCR test result (including tests performed outside of the trial) since their symptom onset

- They report primary endpoint symptoms and have already had a positive SARS-CoV-2 PCR from outside of the trial since symptom onset

During the test visit for COVID-19, examination with nose/throat swab and immunology bloods (serum and others) will be acquired. Further clinical assessments may be performed at COVID-19 testing visits at the discretion of investigators. Symptomatic participants can be regularly monitored by telephone, if appropriate. Participants who test positive for COVID-19 will continue to be followed throughout the duration of the trial, including repeated COVID-19 testing visits if symptomatic again during the course of the study and until the end of the trial. New episodes will be considered if they have a minimum 28 days interval between the previous PCR positive result. Participants who have a positive PCR at S0 will be reviewed by phone for symptom severity data at approximately 7 days post positive swab. Closer follow-up and safety monitoring may be carried out by local trial teams if felt this is clinically indicated. Symptomatic participants with a positive PCR test outside of the trial will be asked to attend for a COVID-19 testing visit in order to take a further swab to facilitate SARS-CoV-2 sequencing and additionally to acquire immunology bloods.

7.4.4.3 COVID-19 Testing plus 7 day phone call

For symptomatic participants with a positive SARS-CoV-2 PCR test, a remote follow up (via phone or other appropriate means) will take place at approximately 7 days (see schedule of procedure tables for window, section 7.3) from the date of their first positive PCR test in that illness episode. The purpose of this remote follow up is to capture further symptom severity data.

7.4.4.4 Positive SARS-CoV-2 PCR test and vaccination

Participants who develop COVID-19 symptoms and have a positive PCR test after the first vaccination can only receive a booster dose after a minimum 4 weeks interval from their PCR positive test, provided their symptoms have significantly improved. The decision to proceed with booster vaccinations in those cases will be at clinical discretion of investigators. Booster vaccinations of participants are allowed to take place beyond the pre-specified 12 weeks window limit if they are required to be delayed due to PCR positivity within the preceding 4 weeks of the scheduled booster dose.

Further details and instructions on the symptomatic pathway can be found on the clinical study plan.

7.4.5 Symptomatic participants from Protocol 13.0 onwards

Study participants receiving the fourth dose of ChAdOx1 nCOV-19 vaccine (group 2) will be asked about COVID-19 history, symptoms or any positive SARS-CoV-2 PCR or lateral flow antigen test results at each study visit.

All SARS-CoV2 infection cases will be rated as per the clinical progression scale proposed by the World Health Organization. In both hospitalized and non-hospitalized cases, the maximum score and symptom duration will be recorded.

Hospitalization due to COVID-19 will be considered a SAE and reported as described in Section 9.8.

7.4.6 Review of the medical record

With the consent of the participant, the study team will request access to the medical records or send a data collection form to be filled out by the clinical team, in any episodes of medically attended COVID-19. Investigators will aim to collect clinical data from medical records where participants with suspected COVID-19 have been admitted to hospital.

Prior to protocol V 13.0, Relevant data will be collected to verify the efficacy endpoints and disease enhancement. There is no internationally accepted definition of disease enhancement. Severity between groups will be described and compared. In addition, a proportion of serious illness/all illnesses will be constructed for recipients of the candidate and control vaccine. In case the vaccine induces increased disease, this proportion would be higher in the vaccine than in the control group. These probably include, but are not limited to information about ICU admissions, clinical parameters such as oxygen saturation, respiratory rates and vital signs, need for oxygen therapy, need for ventilatory support, blood test results and images, among others.

From Protocol V13.0 Relevant data will be collected to the extent required to support SAE reporting as described in Section 9.8.

7.4.7 Randomization, blinding, and unblinding

Participants will be randomized to investigational vaccine or MenACWY vaccine in a 1:1 allocation ratio, using block randomization of 4 participants. The blinding scheme will be

applied to the participants in relation to the arm in which they were allocated. The blinding scheme will not apply to the study team administering the vaccine. Vaccines will be prepared out of the participant's reach and the syringes will be covered with an opaque label that will guarantee the unilateral blinding of the participant.

If a participant's clinical condition requires unblinding, this will be done according to a specific study work instruction and the group allocation will be sent to the attending physician if unblinding is considered relevant and possibly changes treatment clinical.

The unblinding process and vaccination with investigational product is assured to all volunteers (item 8.11) and will be offered as soon as possible. This process will be followed by the vaccination of the control group within two and a half month maximum for the first dose of the vaccine. The unblinding event may happen face to face or electronically/online in order to allow the volunteers to have the option to get another vaccine available in the country.

If the volunteer belongs to the Control Group and did not get the booster dose yet (placebo dose/saline solution) this dose will be replaced by the Chadox, if the volunteer agrees with that.

The booster dose will be offered up to three months post first dose.

Participants will be censored in the analysis of efficacy endpoints at the time of their unblinding and/or vaccination with an approved/licensed SARS-CoV-2 vaccine (whichever happens first).

7.4.8 Unblinded participants who are eligible to receive an approved or licensed SARS-CoV-2 vaccine

If the efficacy of the candidate vaccine is proven, after analysis of the primary endpoint and approval by the DSMB (Data and Safety Monitoring Committee), as established in the study protocol, the sponsor will make the candidate vaccine available to participants in the research allocated to the comparator group (MenACWY vaccine).

Also, In the event that ChAdOx1 nCoV-19 or other SARS-CoV-2 vaccines being approved for emergency use or licensed by the regulatory agency, all the participants will be invited to the unblinding event which can be face to face or online/electronically, in order to make this information available to all the volunteers as soon as possible, allowing them to have the free option to either receive the ChAdOx1 nCoV-19 (if control group) or another vaccine available in the country.

The order for the volunteers booking will follow, as much as possible, as far as it does not delay the unblinding, the priority groups identified by the NIP (National Immunization Program), but all the participants will be booked for the unblinding event.

We estimate to vaccinate the entire study control group in approximately 2 (two) and a half month. Those in the control group will receive two doses of the ChAdOx1 nCoV-19 vaccine if they wish and will have an optional blood sample collected before each vaccination. The interval between doses will be from 4 to 12 weeks. If after unblinded participants in the control group choose to receive an approved vaccine other than ChAdOx1 nCoV-19, and as part of the national roll-out strategy, they will be free to do so. Participants will be asked about the vaccine they received and date of administration which will be recorded along with any other vaccines received in a specific external vaccination CRF.

Follow up visits by both groups (investigational group and control group that will receive ChAdOx1 nCoV-19 vaccine) will continue according to the participants' previous schedule and their data will contribute to continuous safety monitoring and exploratory assessments of immunogenicity.

For those in the control group who chose to receive ChAdOx1 nCoV-19 as part of the study, a second extra appointment for their booster dose will be scheduled 4-12 weeks later, an optional blood sample will be collected before vaccination.

For those who were unblinded and received a single dose of the ChAdOx1 nCov-19 vaccine, a second dose will be offered at the unblinding visit (minimum 4 weeks interval from prime).

The control group participant who chooses to receive the ChAdOx1 nCov-19 vaccine and has not yet received a booster dose, which is a placebo (saline solution), and is at least 4 weeks apart from MenACWY vaccine, this dose will be replaced by the ChAdOx1 nCov-19 vaccine.

All participants that are unblinded should continue to follow their normal schedule of visits. Wherever possible, unblinding visits and vaccination of controls, or second dose of those who received single-dose only may be combined with pre-scheduled study visits.

All participants will receive a vaccination card with details of the vaccines taken during the study and if selected by the volunteer post unblinding, the new doses of the ChAdOx1 nCov-19 vaccine.

All SAEs and AEs will be collected from the point of unblinding onwards. Participants will then follow their normal schedule of attendances.

From 4th dose administration onwards, all local and systemic AEs after vaccination reported by the participant will be recorded by a subset of 100 participants in the Diary of Symptoms for 7 days. Unsolicited AEs (that may not meet SAE or AESI criteria) will be collected by the investigator during Visit 2, at Day 28 post 4th dose. All AEs that result in the withdrawal of a participant from the study will be followed up until a satisfactory resolution occurs (if the

participant agrees to do so), or until a causality unrelated to the study is assigned. SAE and Adverse Events of Special Interest will be collected throughout the study period.

7.4.9 Eligible participants who will receive a third dose of ChAdOx1 nCoV-19 vaccine

From Protocol 12.1, a third late dose of ChAdOx1 nCoV-19 vaccine will be offered to a subgroup of participants who previously received two doses of Chadox with an interval of 4 to 12 weeks, 11-13 months after the second dose.

This information will be made available to all volunteers of group 1c and receiving the third dose will be optional. Those volunteers who wish to receive the third dose of ChAdOx1 nCoV-19 will be re-consented and will have a blood sample taken immediately before vaccination, and another 28 days after the third dose.

A subset of 100 volunteers will receive a 7-day electronic diary (hard copy back-up) to record requested adverse events. When completed on hard copy, this diary will be delivered to the center at visit after the third dose, 28 days after vaccination.

7.4.10 Eligible participants who will receive a fourth dose of ChAdOx1 nCoV-19 vaccine

From Protocol 13.0, a fourth dose of ChAdOx1 nCoV-19 vaccine will be offered to a randomly selected subgroup who previously received three doses of ChAdOx1 nCov-19 in the trial (group 1e), with an interval of 6 to 15 months after the third dose. Up to 350 (+/-10%) volunteers who wish to receive the fourth dose of ChAdOx1 nCoV-19 will be re-enrolled (if they have completed the study) and provide informed consent to receiving a 4th dose of vaccine. Participants will have a blood sample taken immediately before vaccination, another sample taken at 28 days after the fourth dose, and a third blood sample 180 days after the fourth dose.

A subset of 100 volunteers will receive a 7-day electronic diary (hard copy back-up) to record solicited adverse events.

8 Investigational Medicinal Product and Control

8.1 Description of ChAdOx1 nCoV-19

ChAdOx1 nCoV-19 vaccine consists of the ChAdOx1 deficient replication monkey adenovirus vector, containing the SARS-CoV-2 structural surface glycoprotein antigen.

8.2 Storage

The vaccine manufactured by Advent is stored at -80°C in a safe freezer at the clinics. The vaccine manufactured by Cobra Biologics Ltd is stored at 2-8°C in a secure fridge, at the clinical site. The traceability of the study vaccines will be documented according to the existing standard operating procedure (SOP). Accounting, storage, shipping, and handling of vaccines will be in accordance with relevant SOPs and forms.

8.3 Administration

On the day of vaccination, ChAdOx1 nCoV-19 will be thawed at room temperature and will be administered according to specific assay instructions. The vaccine manufactured by Cobra Biologics is a multi-dose vial which is stored at 2-8 degrees and does not require thawing. If the vaccine is stored outside of 2-8 it must be used within 6 hours. If stored at 2-8°C after the first vial puncture, it can be used within 48 hours. The vaccine will be administered intramuscularly in the deltoid of the non-dominant arm (preferably). All participants will be observed in the unit for a minimum of 15 minutes after vaccination. During the administration of research products, Advanced Life Support medications and resuscitation equipment will be immediately available for the treatment of anaphylaxis. Vaccination will be performed, and IMPs handled according to the relevant SOPs.

8.4 Rationale for selected dose

The dose to be administered in this study was selected based on clinical experience with the adenovirus vector ChAdOx1 expressing different inserts and other similar vectorized adenovirus vaccines (for example, ChAd63).

A first dose escalation study in humans using the ChAdOx1 vector encoding an influenza antigen (FLU004) administered ChAdOx1 NP + M1 safely at doses ranging from 5 x 10^8 to 5 x 10^{10} pv. The subsequent review of the data identified an optimal dose of 2.5 x 10^{10} pv, balancing immunogenicity and reactogenicity. This dose was later administered to hundreds of volunteers in numerous larger phase 1 studies at the Jenner Institute. ChAdOx1 vectorized

vaccines have so far shown to be very well tolerated. The vast majority of AEs have been mild to moderate and there have been no SARs to date.

Another monkey adenovirus vector (ChAd63) was safely administered in doses of up to 2 x 10^{11} pv, with an optimal dose of 5 x 10^{10} pv, balancing immunogenicity and reactogenicity.

MERS001 was the first clinical trial of a vector ChAdOx1 expressing the total Spike protein from a separate, but related beta-coronavirus. ChAdOx1 MERS has been administered to 31 participants so far in doses ranging from 5x10^{9} pv to 5x10^{10} pv. Despite the greater reactogenicity observed with the dose of 5x10^{10} pv, this dose was safe, with self-limiting AE and without registered SARs. The dose of 5x10^{10} pv was the most immunogenic, in terms of inducing neutralizing antibodies against MERS-CoV using a live virus assay (Folegatti et al. Lancet Infect Dis, 2020, https://doi.org /10.1016/S1473-3099(20)30160-2). Due to the immunology findings and the safety profile observed with a ChAdOx1 vector vaccine against MERS-CoV, the dose of 5x10^{10} pv was chosen for ChAdOx1 nCoV-19.

An analytical comparability assessment of ChAdOx1 nCoV-19 (AZD1222) manufactured by CBF, Advent and Cobra Biologics was conducted using a comprehensive set of physiochemical and biological release and characterization tests. In order to support the analytical comparability assessment, A260 testing of Advent's process (K.0007, K.0008, and K.0009 lots) was performed, where corrections to the absorbance due to excess polysorbate 80 were made to compensate for polysorbate 80 concentrations above the formulation target of 0.1% (w/v).

Differences in strength related attributes (ie, virus particle concentration, virus genome concentration, and infectious virus concentration) are noted. These differences in strength is further examined for potential impact on clinical dosing. The target clinical dosage of CBF's product is 5 × 10^{10} viral particles per dose based on vp/mL concentration determined by UV spectroscopy (A260), whereas that of Advent's product is 5 × 10^{10} viral genome copies per dose based on vg/mL concentration determined by qPCR. The target clinical dosage of Symbiosis' product is 3.5 – 6.5 × 10^{10} viral particles per dose based on the vp/mL concentration determined by A260, with a 0.5 mL dosing volume. This dosing range is based on a target 5 × 10^{10} viral particles per dose and a ± 30% range to take into account process and method variabilities. The planned clinical dosage of Symbiosis' product is compared to that of CBF and Advent products, the resulting Symbiosis' product dosage at 0.5 mL for lot 20481A is somewhat lower in total viral particle per dose (20% from the lower range limit), slightly higher in total viral genome copies per dose (12% from the higher range limit), and slightly lower in total infectious particle per dose (8% from the lower range limit). These differences are considered to be comparable to or within the variabilities from the analytical

methods used in concentration determination (A260, qPCR, and infectivity) and the dosing volumes during clinical administration. In summary, with a 0.5 mL dosing volume for Symbiosis' product, strength difference from CBF and Advent products is not expected to have significant clinical impact in terms of reactogenicity and immunogenicity/efficacy

Table 12 Clinical Strengths of ChAdOx1 nCoV-19 (AZD1222) Drug Product

Strength Attribute	CBF		Advent			Cobra
	Lot 02P20-01	Lot 02P20-02	Lot K.0007	Lot K.0008	Lot K.0009	Lot 20481A
**Concentration**						
Virus particle concentration (A_{260}) (vp/mL)	1.49×10^{11}	1.22×10^{11}	3.12×10^{11}	3.16×10^{11}	2.45×10^{11}	0.8×10^{11}
Virus genome concentration (qPCR) (vg/mL)	1.7×10^{11}	Not tested	1.7×10^{11}	2.1×10^{11}	1.4×10^{11}	1.3×10^{11}
Infectious particle concentration (ifu/mL) [a]	2.6×10^{9}	Not tested	2.9×10^{9}	3.0×10^{9}	2.4×10^{9}	1.3×10^{9}
**Target Clinical Dosage**						
Equivalent DP volume per dose (mL)	0.34	0.41	0.294	0.235	0.356	0.50
Dosing of virus particle (vp/dose)	5.1×10^{10}	5.0×10^{10}	9.2×10^{10}	7.4×10^{10}	8.7×10^{10}	4.0×10^{10}
Dosing of viral genome (vg/dose)	5.8×10^{10}	NA	5.0×10^{10}	4.9×10^{10}	5.0×10^{10}	6.5×10^{10}
Dosing of infectious particle (ifu/dose)	8.8×10^{8}	NA	8.5×10^{8}	7.1×10^{8}	8.5×10^{8}	6.5×10^{8}

ifu = infectious units; NA = not applicable; vp = virus particle; vg = virus genome

[a] Testing performed using the Advent infectivity assay.

8.5 Environmental contamination control (GMO)

The possibility of environmental contamination with genetically modified organisms (GMOs) will be appropriately controlled. The study will be performed in accordance with the relevant local regulations regarding GMO products, following the recommendations of CTNBio. The approved SOPs will be followed to minimize the spread of the recombinant vector vaccine virus in the environment. GMO residues will be inactivated according to the approved SOPs. All material used during vaccination and by vaccination personnel will be autoclaved and incinerated later.

8.6 Investigational Medicinal Product for the 4th dose study

Recombinant Covid-19 vaccine (AstraZeneca/Halix) containing chimpanzee adenovirus codifying Spike SARS-CoV-2 glycoprotein will be used for the 4th dose study. The standard dose of AstraZeneca/Halix COVID-19 vaccine is $5x10^{10}$vp in 0.5ml. The vaccine should be administered intramuscularly. The AstraZeneca/Halix vaccine is supplied in packs of 10 vials. Each vial contains 10 doses of vaccine, and is a colourless to slightly yellow, clear to slightly opaque liquid.

The AstraZeneca/Halix vaccine should be stored at +2°C to +8°C and has a shelf life of 6 months. The vaccine does not contain any preservative. After first opening the vial, it should be used within 6 hours when stored at room temperature (up to 30°C) or within 48 hours when stored in a refrigerator (2 to 8°C). After this time, the vial must be discarded. The total cumulative storage time once opened must not exceed 48 hours.

8.7 Control Vaccine

Participants who are allocated to control groups will receive an injection of the MenACWY vaccine instead of ChAdOx1 nCoV-19. Either of the two MenACWY vaccines of authorized quadrivalent protein-polysaccharide conjugate – will be used, i.e.:

- Nimenrix (Pfizer). The authorized dosage of this vaccine for those over 6 months of age is a single intramuscular dose (0.5 mL), containing 5 mcg each of a polysaccharide from group A, C, W and Y of *Neisseria meningitidis*, each conjugated with 44 mcg of tetanus toxoid carrier protein.
- Menveo (Glaxo Smith Kline). The authorized dosage of this vaccine for those aged 2 years or older is a single intramuscular dose (0.5 mL), containing
  - 10 mcg of group A meningococcal polysaccharide, conjugated with 16.7 to 33.3 mcg of CRM $protein_{197}$ of *Corynebacterium diphtheriae*.
  - 5 mcg of group C meningococcal polysaccharide, conjugated with 7.1 to 12.5 mcg of CRM $protein_{197}$ of *Corynebacterium diphtheriae*.
  - 5 mcg of group W meningococcal polysaccharide, conjugated with 3.3 to 8.3 mcg of CRM $protein_{197}$ of *Corynebacterium diphtheriae*.
  - 5 mcg of group Y meningococcal polysaccharide, conjugated with 5.6 to 10.0 mcg of CRM $protein_{197}$ of *Corynebacterium diphtheriae.*

The summary of product characteristics for both vaccines allows the administration of a booster dose, if indicated by ongoing risk. Prior administration of a vaccine (or a quadrivalent simple meningococcal polysaccharide vaccine in groups A, C, W and Y) is not a contraindication to receiving another vaccine in this study.
The masking of the participants regarding the injection they are receiving will be maintained. A vaccination accounting record from MenACWY will be maintained at each study site.

MenACWY will be stored in a locked (or controlled access) refrigerator (2 °C to 8 °C) at the research site, according to the package insert.

8.8 Placebo

Participants who were allocated to the control group will receive a placebo injection of 0.9% saline instead of MenACWY at the time of boosting. The volume and site of injection will be the same as for the intervention arm and participants will be blinded as to which injection they are receiving. A vaccine accountability log of the saline will be maintained at each trial site, similar to what is done for the study intervention (ChAdOx1 nCoV-19) and the comparator used as the prime dose (MenACWY).

8.9 Compliance with the investigational treatment

All vaccines will be administered by the research team and registered with the CRF. The study medication will not be in the participant’s possession at any time and, therefore, compliance will not be a problem.

8.10 Investigational treatment accounting

IMP accounting and control vaccines will be performed in accordance with the relevant SOPs.

8.11 Concomitant medication

As established by the exclusion criteria, participants cannot be enrolled in the study if they have received: any vaccine within 30 days prior to enrollment or if any other vaccine is expected to be administered within 30 days after each vaccination, any research product within 30 days prior to recruitment or if administration is planned during the study period, or if there is any chronic use (> 14 days) of any immunosuppressive medication in the 6 months prior to enrollment or if administration is planned at any time during the study period (topical steroids are allowed).

Participants who make continuous use of oral anticoagulants, such as coumarins and related anticoagulants (i.e. warfarin) or new oral anticoagulants (i.e. apixaban, rivaroxaban, dabigatran and edoxaban), and/or who received immunoglobulins and/or any blood products

in the three months prior to the planned administration of the candidate vaccine, will be excluded from this study, according to the exclusion criteria.

Participants will be advised to take paracetamol, unless contraindicated (in which case they will be excluded from the study), for 24 hours after vaccination. Paracetamol will be stored according to the package insert. There will be no additional labeling beyond its authorized packaging.

8.12 Provision of treatment for controls

If the efficacy of the candidate vaccine is proven, after analysis of the primary endpoint and approval by the DSMB (Data and Safety Monitoring Committee), as established in the study protocol, the sponsor will make the candidate vaccine available to participants in the research allocated to the comparator group (MenACWY vaccine).

9 SAFETY ASSESSMENT

Safety will be assessed by the frequency, incidence and nature of the AEs and SAE emerging during the study.

9.1 Definitions

9.1.1 Adverse Event (AE)

An AE is any unexpected medical occurrence in a participant, which can occur during or after the administration of an IMP and does not necessarily have a causal relationship to treatment. An AE can therefore be any unfavorable and unintended sign (including any clinically significant abnormal finding or change from baseline), symptom or disease temporally associated with study treatment, even if it is considered to be related to study treatment or not.

9.1.2 Adverse Reaction (AR)

An AR is any unexpected or unintended response to an IMP. This means that the causal relationship between the IMP and the AE is at least reasonable, that is, the relationship cannot be ruled out. All cases judged by the medical investigator to have a reasonable causal relationship with an IMP (that is, possibly, probably, or definitely related to an IMP) will qualify as AR.

Adverse events that may be related to the IMP are listed in the Investigator's Brochure for each product, as well as within the product information for the applicable market product.

9.1.3 Serious Adverse Event (SAE)

An SAE is an AE that results in any of the following endpoints, considered or unrelated to the study treatment.

- Death;
- Life-threatening event (i.e., the participant was, in the Investigator's view, at immediate risk of death from the event);
- Persistent disability or disability or significant disability (i.e., substantial disruption of the ability to perform normal life functions);
- Hospitalization or extension of existing hospitalization, regardless of length of stay, even if it is a precautionary measure for continuous observation;
    - Note: Hospitalization (including hospitalization or outpatient hospitalization for an elective procedure) for a pre-existing condition that has not unexpectedly worsened does not constitute a serious AE.
- An important clinical event (which cannot cause death, be life-threatening or require hospitalization) that may, based on appropriate clinical criteria, harm the participant and/or require medical or surgical treatment to avoid one of the outcomes listed above. Examples of such clinical events include an allergic reaction that requires intensive treatment in an emergency room or clinic, blood dyscrasias or seizures that do not result in hospitalization;
- Congenital anomaly or birth defect.

9.1.4 Serious Adverse Reaction (SAR)

A serious AE that, in the opinion of the investigator or sponsor, is believed to be possibly, probably, or definitely related to IMP or any other treatment of the study, based on the information provided.

9.1.5 Suspected Unexpected Serious Adverse Reaction (SUSAR)

A SAR, whose nature and severity are not consistent with the information on the drug in question set out in the IB.

9.2 Expectation

Refer to expected SARs as listed within the product information for the applicable market product.

9.3 Predicted/expected adverse reactions:

Predictable/expected AR after vaccination with ChAdOx1 nCoV-19 include pain at the injection site, tenderness, erythema, heat, swelling, induration, itching, myalgia, arthralgia, headache, fatigue, fever, feverish feeling, chills, malaise, and nausea.

9.4 Adverse Events of Special Interest

AESI relevant to vaccination in general will also be monitored, such as: generalized seizure, Guillain-Barre Syndrome (GBS), Acute Disseminated Encephalomyelitis (ADEM), Thrombocytopenia, Anaphylaxis, Vasculitis, in addition to the requested serious AEs.

Thrombotic, thromboembolic, and neurovascular events will also be considered an AESI.

Additionally, potential immune mediated diseases (pIMDs), which include autoimmune and other inflammatory and/or disorders of interest that may or not have an autoimmune etiology, will be considered an AESI and are listed on Appendix A. However, the investigator will exercise their medical and scientific judgment when deciding whether other diseases have autoimmune origin (i.e., physiopathology involving systemic pathogenic or organ-specific autoantibodies) and should also be recorded as a pIMD.

9.5 Causality

For each AE, an assessment of the relationship between the event and the administration of the vaccine will be performed by the clinician delegated by the IC. An interpretation of the causal relationship of the treatment with the AE in question will be made, based on the type of event; the relationship of the event to the time of vaccine administration; and the known biology of vaccine therapy. Alternative causes of AE will be considered and investigated, such as the natural history of pre-existing medical conditions, concomitant therapy, other risk factors and the temporal relationship of the event to vaccination. The causality assessment will take place during planned safety reviews and in the final safety analysis, except for SAEs, which must be designated immediately by the investigator reporting the events.

0	Not related	No temporal relationship with the product under study ***and*** alternative etiology (clinical, environmental, or other treatments) ***and*** Does not follow known pattern of response to the product under study
1	Unlikely	Unlikely temporal relationship with the product under study ***and*** alternative etiology (clinical, environmental, or other treatments) ***and***

		It does not follow the typical or plausible pattern of response to the product under study.
2	Possible	Reasonable temporal relationship with the product under study; ***or*** Event not produced immediately by clinical, environmental, or other treatments; ***or*** Response pattern similar to that seen with other vaccines
3	Probable	Reasonable temporal relationship with the product under study; ***and*** Event not produced immediately by clinical, environmental, or other treatments ***or*** Known response pattern seen with other vaccines
4	Definitive	Reasonable temporal relationship with the product under study; ***and*** Event not produced immediately by clinical, environmental, or other treatments; ***and*** Known response pattern seen with other vaccines

Table 5. Guidelines for assessing the relationship between vaccine administration and an AE.

9.6 Reporting procedures for all adverse events

All local and systemic AEs after vaccination observed by the Investigator or reported by the participant, whether or not attributed to the study medication, will be recorded by the participants in the Diary of Symptoms and by the Investigators in the study CRF. All AEs that result in the withdrawal of a participant from the study will be followed up until a satisfactory resolution occurs (if the participant agrees to do so), or until a causality unrelated to the study is assigned. SAE and Adverse Events of Special Interest will be collected throughout the study period. All SAEs and AEs will be recorded for participants who are unblinded and receive COVID-19 vaccine as part of the study (ChAdOx1 nCoV-19 for those in the control arm) or any other approved SARS-CoV-2 vaccine given as part of national roll-out strategy

4th dose and reporting procedures for all AEs

All local and systemic AEs after vaccination reported by the participant, whether or not attributed to the study medication, will be recorded by a subset of 100 participants in the Diary of Symptoms for 7 days. Unsolicited AEs (that may not meet SAE or AESI criteria) will be collected by the investigator during Visit 2, at Day 28 post 4th dose. All AEs that result in the withdrawal of a participant from the study will be followed up until a satisfactory resolution occurs (if the participant agrees to do so), or until a causality unrelated to the study is assigned. SAE and Adverse Events of Special Interest will be collected throughout the study period.

9.7 Evaluation of severity

The severity of adverse events will be assessed according to toxicity rating scales adapted from the FDA for healthy volunteers recruited in preventive vaccine clinical trials, listed in the specific study work instructions and tables 6-8 below.

Adverse Event	Grade	Intensity
Injection site pain	1	Pain that is easily tolerated
	2	Pain that interferes with daily activity
	3	Pain that impairs daily activity
	4	Hospitalization or A/E visit
Sensitivity	1	Mild discomfort to touch
	2	Discomfort with movement
	3	Significant discomfort at rest
	4	Hospitalization or A/E visit
Injection site erythema	1	2.5 – 5 cm
	2	5.1 – 10 cm
	3	>10 cm
	4	Exfoliating dermatitis or necrosis
Injection site induration/swelling	1	2.5 – 5 cm and does not interfere with activity
	2	5.1 – 10 cm or interferes with activity
	3	> 10 cm or impairs daily activity

	4	Necrosis

Table 6. Severity criteria for local adverse events

*erythema ≤2.5cm is an expected consequence of skin puncture and therefore will not be considered an adverse event

Vital Signs	Grade 1 (mild)	Grade 2 (moderate)	Grade 3 (serious)	Grade 4 Potentially fatal
Fever (oral)	38, 0°C- 38, 4°C	38.5°C – 38.9°C	39.0°C – 40°C	> 40°C
Tachycardia (bpm)*	101 – 115	116-130	>130	A/E visit or hospitalization for arrhythmia
Bradycardia (bpm)**	50 – 54	45 – 49	<45	A/E visit or hospitalization for arrhythmia
Systolic hypertension (mmHg)	141 -150	151 – 155	≥155	A/E visit or hospitalization for malignant hypertension
Diastolic hypertension (mmHg)	91 – 95	96 – 100	>1100	A/E visit or hospitalization for malignant hypertension
Systolic hypotension (mmHg)***	85 – 89	80 – 84	<80	A/E visit or hospitalization for hypotensive shock
Respiratory Rate – breaths per minute	17 – 20	21-25	>25	Intubation

Table 7. Severity rating criteria for physical observations (applies to adults only).

*Measured after ≥10 minutes at rest ** When the resting heart rate is between 60 to 100 beats per minute. Use the clinical criterion when characterizing bradycardia among some populations of healthy subjects, for example, conditioned athletes. *** Only if symptomatic (for example, dizziness/vertigo)

**GRADE 0**	None
**GRADE 1**	Mild: Transient or mild discomfort (<48 hours); There was no interference with routine activity; no medical treatment/therapy was needed
**GRADE 2**	Moderate: Mild to moderate limitation in routine activity – some assistance may be required; minimal medical treatment/therapy was required or none
**GRADE 3**	Serious: Marked limitation in routine activity, some assistance is usually required; medical treatment/therapy was required.
**GRADE 4**	Potentially fatal: requires assessment in A/E or hospitalization

Table 8. Severity classification criteria for local and systemic AE.

9.8 Serious AE reporting procedures

To comply with the rules in force on reporting SAE to regulatory authorities, the event will be accurately documented, and the notification deadlines respected. Serious adverse events will be reported on the SAE forms to members of the study team as soon as the Investigators become aware of their occurrence. Copies of all reports will be forwarded for review by the Principal Investigator (as Sponsor's representative) within 24 hours after the Investigator becomes aware of the alleged SAE. The DSMB will be notified of SAE that are considered to be possibly, probably or definitely related to the study treatments; the DSMB president will be notified immediately (within 24 hours) as soon as the Sponsor become aware of the occurrence. Normally, SAE will not be reported immediately to the Ethics Committee, unless there is a clinically significant increase in the rate of occurrence, an unexpected endpoint, or a new event that may affect the safety of study participants, at the discretion of the Principal Investigator and/or DSMB. In addition to the expedited report above, the Investigator will include all SAE in the annual Development Safety Update (DSUR) report. In addition, all local reporting requirements apply.

Cases falling under the Hy's Law should be reported as SAEs. A Hy's Law Case is defined by FDA Guidance for Industry "Drug-Induced Liver Injury: Premarketing Clinical Evaluation" (2009). Any study participant with an increase in Aspartate Aminotransferase (AST) or Alanine

Aminotransferase (ALT) ≥ 3x Upper Limit of Normal (ULN) together with Total Bilirubin ≥2xULN, where no other reason can be found to explain the combination of these abnormal results, e.g., elevated serum alkaline phosphatase (ALP) indicating cholestasis, viral hepatitis A, B or C, another drug capable of causing the observed injury, amongst others.

9.9 Procedures for reporting SUSARs

All SUSARs will be communicated by the sponsor's delegate to the Competent Authority and REC and other parties, as applicable. For fatal and life-threatening SUSARs, this will be done within 7 calendar days after the Sponsor or delegate becomes aware of the reaction. Any additional relevant information will be reported within 8 calendar days of the initial report. All other SUSARs will be informed within 15 calendar days.

The principal investigators will be informed of all SUSARs for the relevant IMP for all studies with the same Sponsor, even if the event occurred or not in the present study.

9.10 Safety assessments

The safety profile will be continuously assessed by the Investigators. The CI and relevant investigators (according to the study delegation record) will also review safety and SAE issues as they arise.

The DSMB will assess the frequency of events, safety, and efficacy data every 4-8 weeks and/or as needed. The DSMB will make recommendations regarding the conduct, continuation or modification of the study.

The Sponsor may put the study on hold and pause recruitment if SUSARs reported in other international trials within the ChAdOx1 nCoV-19 programme are considered to pose a significant safety concern to all participants in the programme. The DSMB will review such events and will make a recommendation as to whether or not recruitment can continue. Study procedures other than vaccinations (e.g. safety follow-ups, immunogenicity assessments, and COVID-19 testing procedures) will continue as normal, regardless of length of study pause.

9.11 Data Safety Monitoring Committee

The Data Safety Monitoring Committee that is in place for the British studies will also oversee this study and review the safety data for this study.

At least one properly qualified clinician/scientist from each international study site will be invited to attend meetings of the existing trial's DSMB.

The DSMB president can be contacted for independent advice and review by the Researcher or study sponsor in the following situations:

- Follow-up on any SAE considered possibly, probably or definitely related to a study treatment;
- Any other situation in which the Investigator or Study Sponsor feels that independent advice or review is important.

The DSMB will review the SAE considered to be possibly, probably, or definitely related to the study treatments. The DSMB will be notified within 24 hours after the Sponsor become aware of the occurrence. The DSMB can recommend stopping recruitment into the study, if necessary, to follow up on an SAE related to a study treatment. It will also recommend restarting the study, when appropriate, following review of such safety events (i.e. SUSARs associated with ChAdOx1 nCoV-19).

The DSMB will review safety data from volunteers aged 56 and above recruited as part of the COV002 UK study. Recruitment of older adults will only be allowed following advice from the DSMB.

10 STATISTICS

10.1 Description of statistical methods

Both a fully detailed study level statistical analysis plan (SAP) as well as a separate Statistical Analysis Plan for the Marketing Authorization Application (MAA SAP) will be written and signed off before any interim data analyses are conducted. A SAP describing the analyses for 3rd and 4th dose (including non-inferiority assessments) will be separate from the study-specific and pooled MAA SAP. Immunogenicity analyses on 3rd and 4th dose will be conducted after SAP finalization and sign-off.

The data from this study will be included in prospective pooled analyses of studies for efficacy and safety of ChAdOx1 nCoV-19 to provide greater precision of both efficacy and safety outcomes

10.2 Efficacy Outcomes

The primary efficacy endpoint is PCR positive symptomatic COVID-19.

This is defined as a participant with a PCR+ swab and at least one of the following symptoms: cough, fever > 37.8, shortness of breath, anosmia, or ageusia.

Where possible, sensitivity analyses will be conducted using common alternative definitions of virologically-confirmed COVID-19 disease, including those in use in other phase 3 protocols (including but not limited to: USA AstraZeneca phase 3 trial, South Africa COV005 trial, WHO solidarity trial, CEPI definition). This will aid in comparisons between various studies and meta-analyses. These alternative definitions will be detailed in the statistical analysis plan as exploratory analyses.

Due to the vaccine-induced disease mitigation potential, the inclusion of all positive PCR infections as a primary result may lower the vaccine's estimated effectiveness and reduce its accuracy. COVID-19 disease, positive for PCR and symptomatic, is a more specific primary outcome and may lead to an earlier demonstration of vaccine efficacy, although it includes fewer cases.

Regarding the differentiation of ADE and the lack of effectiveness of the vaccine: there is no internationally accepted definition of ADE. Differences in disease severity between groups will be described. If the proportion of serious illness is similar between the two groups, this would support the lack of effectiveness and not the improvement of the illness.

10.2.1 Efficacy

The primary and secondary analysis will be conducted on participants who are seronegative at baseline. A sensitivity analysis will be conducted including all participants regardless of baseline serostatus.

The screening of the participants will be based on the serological exam with IgG antibodies negative for SARs-CoV-2. However, for the analysis of the primary outcome, a validated assay detecting antibodies against SARS-CoV-2 nucleoprotein will be used to exclude any remaining participants who were seropositive at baseline.

Analysis of the primary endpoint will be computed as follows:

1. **Efficacy of two doses of ChAdOx1 nCoV-19**. Participants will be included who received two doses of ChAdOx1 nCoV-19. Events will be included if they occurred more than 14 days after the booster dose.
   Participants who are symptomatic up to 14 days after the second dose of vaccine will be excluded from the analysis. In addition, those with less than 14 days follow up post-second dose will also be excluded.

Secondary analyses of the primary outcome:

2. **Efficacy of at least one dose of vaccine**. Cases occurring more than 21 days after the first vaccination will be included.

Participants who are symptomatic up to 21 days from vaccination will still attend site for PCR testing and blood samples but will be excluded from the analysis as these participants may have been exposed to SARS-CoV-2 prior to vaccination or before the immune system has had time to mount a response to the vaccine. In addition, those with less than 21 days follow up post-vaccination will also be excluded.

The proportions of participants meeting the primary outcome definition will be compared between groups of recipients of ChAdOx1 nCoV-19 and MenACWY using a Poisson regression model with robust variance (Zou 2004). The model will contain terms including treatment group, and age group at randomization if there is a sufficient sample size within each age category. The logarithm of the period at risk for primary endpoint will be used as an offset variable in the model to adjust for volunteers having different follow up times during which the events occur. Vaccine efficacy (VE) will be calculated as (1 – RR) x 100%, where RR is the relative risk of symptomatic infection (ChADOx1 nCoV-19: Control) and 95% confidence intervals will be presented.If the Poisson regression model with robust variance fails to converge, the exact conditional method for stratified Poisson regression will be used.

The cumulative incidence of symptomatic infections will be presented using the Kaplan-Meier method.

Secondary efficacy endpoints will be analyzed in the same way as the primary efficacy endpoint.

Analyses will be conducted for all adults combined as well as conducting analyses stratified by age cohorts.

All data from participants with PCR-positive swabs will be assessed for inclusion in the efficacy analyses by two blinded assessors who will independently review each case according to pre-specified criteria as detailed in the statistical analysis plan, to classify each for inclusion in the primary and secondary outcomes. A separate CRF will be designed for this purpose.

All PCR-positive results will be assessed for the primary outcome, including those with symptoms who were swabbed by trial staff and other potential sources of information such as health-care workers who are tested at their workplace as either a routine test procedure or due to developing symptoms.

Exploratory COVID-19 disease endpoints may be assessed by trained endpoint adjudicators within the trial team, following pre-specified criteria, rather than independent blinded assessors.

10.2.2 Safety and Reactogenicity

For each group, the counts and percentages of each local and systemic adverse reaction requested from the daily cards, and all unsolicited AEs and SAEs will be presented.

10.2.3 Immunogenicity

Highly deviated antibody data will be transformed logarithmically before analysis. The geometric mean of the concentration and the associated 95% confidence interval will be summarized for each group at each timepoint, calculating the anti-log of the average difference of the logarithmically transformed data.

The geometric means of concentration on day 28 and the proportion of participants with serum conversion to S-spike protein from day 0 to day 28 will be computed. Comparisons between the ChAdOx1 nCoV-19 vaccine and control groups will be made using a Mann Whitney U test due to the low titers expected in the control group that will cause non-normal distribution.

10.3 3rd and 4th dose studies

The overall hypothesis for the extension to examine 3rd and 4th dose in this study is that 28 days after receiving a 4th dose the response will be non-inferior to the response 28 days after receiving the 3rd dose for immunogenicity (ie, neutralising antibodies geometric mean titre ratio). A high variation of intervals between 3rd and 4th dose was expected, and all the participants will be combined in the primary analysis when comparing with the response at 28 days post 3rd and 4th dose. Subgroup analyses will be also conducted by presenting the neutralising antibodies geometric mean titre ratio in participants with different 3rd to 4th dose intervals (below and above median). The specific null and alternative hypotheses for each objective will be presented in a 3rd/4th dose SAP.

The immune response 28 days after the 3rd or 4th dose vaccines, will be compared to the immune response 28 days after the previous vaccine (2nd or 3rd dose) using non-inferiority comparisons. The within-person difference between log-transformed responses (dose 4$^{+28 \text{ days}}$ minus dose 3$^{+28 \text{ days}}$) will be computed and non-inferiority will be assessed. All non-inferiority comparisons of geometric mean titre ratios will be made utilizing the lower bound of two-sided confidence intervals ($\alpha = 0.05$) with non-inferiority margin 2/3

All non-inferiority comparisons of seroresponse rates will be made utilizing the lower bound of two-sided confidence intervals ($\alpha = 0.05$) with non-inferiority margin 10%.

If the number of participants seronegative at baseline, intra-subject correlation and the observed standard derivation combine to provide ≥ 80% power for non-inferiority

assessments, the analysis will be performed as planned. Alternatively, if any factor or combination of factors results in <80% power, the primary analysis will be descriptive immunogenicity only, with all comparative analyses being exploratory, including any subgroup comparison. The details will be described in the SAP.

To evaluate the cellular immune responses, a subset of up to 40 participants was chosen base on practical constraints and the sizes of previous similar studies. The analysis on cellular responses will be descriptive and no formal non-inferiority hypothesis testing will be conducted.

Given the reactogenicity of ChADOx1 nCoV-19 has been well-studied, we chose a subset of 100 participants to collect the reactogenicity data post 3rd and 4th dose. Assuming the true rate of a solicited AE is 5%, a sample size of 100 provides >99% probability to observe at least one solicited AE.

10.4 Subgroup analyses

Subgroup comparisons of efficacy, and safety will be conducted by incorporating vaccine-group by subgroup interaction terms into appropriate regression models. Subgroup comparisons will only be conducted if there are at least 5 cases in all subgroups.

Comparisons will include:

1. Males vs females
2. Age (18 to 55 years vs 56-<70 years vs 70+ years)
3. Seropositive to S-spike or non-spike proteins at baseline vs not seropositive
4. Health care workers and highly-exposed participants versus others
5. Ethnicity
6. BMI (< 30 / >= 30 kg/m2)

10.5 Number of Participants

The research sites will include up to 10,300 participants (with a margin of 1%).

Fourth dose boost substudy

Data are available from Flaxman et al to inform sample size calculations as follows (https://www.sciencedirect.com/science/article/pii/S0140673621016998)

Table 8 Data available for sample size calculation from Flaxman et al

Assay	Visit	N	Mean (log10)	SD (log10)	Pearson correlation coefficient
Anti-spike IgG (ELISA)	Dose 2 + 28 days	71	3.28169	0.51413	0.33506
	Dose 3 + 28 days	71	3.55010	0.38949	
	**Difference**	**71**	**0.26841**	**0.53089**	

Assuming the standard deviation for the difference between dose 3 and dose 4 is similar to the standard deviation for the difference between dose 2 and dose 3 as seen in Flaxman et al, then 300 participants will provide >99% power to show non-inferiority of the 4th dose compared with the 3rd dose, assuming a non-inferiority margin for the lower bound of the GMFR of 2/3 (-0.176 on $\log_{10}$ scale), and alpha of 0.025. The final numbers for inclusion in the analysis will depend on the proportion who are eligible at the time of vaccination.

Table 9 Power available for different numbers of participants included in the 4th dose sub-study analysis

Power	N	SD	Alpha
>99%	250	0.531	0.025
>99%	300		
>99%	350		

10.6 Interim and primary analyses of the primary outcome

It is planned that the primary evidence of efficacy and safety for the ChADOx1 nCoV-19 vaccine will be based on global analyses utilizing studies COV001 (the UK P1/2 study), COV002 (the UK P2/3 study), COV003 (the Brazil P3 study) and COV005 (the South Africa P1/2 study) including a pooled analysis across the studies. As such the interim and primary analyses for the primary outcome will be based on cases accumulated across multiple studies, details of which will be specified within the MAA SAP rather than for each individual study. Interim and primary data cuts from this study will therefore be carried out to support the pooled analysis. The global

MAA SAP allows for interim and primary analyses to be conducted once sufficient eligible cases have accumulated, where the overall type 1 error is controlled at the 5% level using a flexible alpha–spending approach that accounts for the incorporation of data from this study into pooled interim analyses under the global MAA SAP.

Evidence of efficacy will be determined if the lower bound of the multiplicity adjusted confidence interval is greater than a 20% threshold. The primary analysis will have approximately 90% power assuming a vaccine efficacy of 60%. A flexible alpha spending approach will be implemented to allow an earlier primary analysis in the situation where accumulation of eligible cases was lower than expected.

Evidence of efficacy at an interim or primary analysis of pooled data will not be considered a reason to stop the trial, but instead will be interpreted as early evidence of efficacy. However, if an interim analysis demonstrates evidence of efficacy, then a study level analysis according to the study SAP may be used to support study level evidence of efficacy.

10.7 Final Analysis

A final analysis will be conducted at the end of the study. The final study-specific analysis will incorporate all data from the study, including data that has previously contributed to global efficacy estimates under the pooled analysis strategy. The final analysis will be considered a supportive analysis to the global efficacy analysis. Participants will be censored in the analysis at the time of unblinding or their vaccination with an alternative COVID-19 vaccine.

Although the roll-out of COVID19 vaccines will affect vaccine efficacy estimation over time, all participants remain in the studies and continued to be followed-up. Respective immunological assessments will provide important insights regarding duration of protection.

In this context, exploring duration of protection through efficacy endpoints will utilize different statistical approaches. This will include traditional techniques, using all available data with censoring of at the time of unblinding because of the decision to switch to a vaccine. Given the Poisson regression model includes an offset term for follow-up, it has the flexibility to allow for multiple imputation approaches to assess vaccine efficacy using different assumptions around the control attack rate after unblinding. Data collected after unblinding will also allow for a longer-term descriptive assessment of cumulative incidence of efficacy for participants randomized to the vaccine arm. In addition, more novel techniques for an inferred control arm are also being considered, extending the methods described in Follmann et al 2020.

10.8 Inclusion in the analysis

All vaccinated participants will be included in the analysis, unless otherwise specified

10.9 Data and Safety Monitoring Committee

The independent DSMB will meet regularly to review safety data from all available studies of ChAdOx1 nCoV-19. Additionally, the independent DSMB will make recommendations based on the interim analyses to assess evidence of efficacy. The DSMB works according to the DSMB statute and/or follows the trigger points of the different protocols of the global clinical development plan, allowing the different steps to be achieved with respect to safety.

11 DATA MANAGEMENT

11.1 Data processing

The Principal Investigator will be responsible for all data that accumulates in the study.

All study data, including the participant's diary, will be recorded directly in an Electronic Data Capture (EDC) system (for example, OpenClinica, REDCap or similar) or in a paper source document for later insertion in EDC if the direct entry is not available. This includes safety data, laboratory data and endpoints data. All documents will be stored securely and in confidential conditions.

Participants will be identified by a unique number and code specific to the study in any database. The name and any other identifying details will NOT be included in any electronic study data file.

The EDC system (CRF data) uses a relational database (MySQL/PostgreSQL) through a secure web interface with data checks applied during data entry to ensure data quality. The database includes a full set of features that comply with GCP, EU and UK regulations and Sponsor security policies, including a full audit trail, user-based privileges and integration with the institutional LDAP server. The MySQL and PostgreSQL databases and the web server will be hosted on servers that are kept secure. The backups will be stored according to the IT department's schedule on a daily, weekly, and monthly basis and retained for one month, three months and six months, respectively. IT servers provide a stable, secure, well-maintained, high-capacity data storage environment. RedCap and OpenClinica are widely used, powerful, reliable and well supported systems. Access to the study database will be restricted to members of the study team with a username and password.

Unblinding: The date of unblinding will be recorded in the database. If the control group receives the vaccine through the study team, the date and details of vaccination will be entered into the database. If the vaccine is received in the national roll out but the national programme, participant may be contacted to provide information or if no response is received the information will be collected at the next study visit.

11.2 Record keeping

Investigators will maintain adequate medical and research records for this trial, in accordance with the GCP and regulatory and institutional requirements to protect the confidentiality of participants. The principal investigator, sub investigators and clinical research nurses will have access to the records. Investigators will allow Sponsor's authorized representatives, as well as ethical and regulatory agencies, to examine (and when required by applicable law, copy) clinical records for the purposes of quality assurance reviews, audits and evaluation of the safety and progress of the study.

11.3 Source data and technical data sheets (CRFs)

All information required by the protocol will be collected in CRFs designed by the Sponsor. All source documents will be archived. The source documents are documents, data and original records from which the participant's CRF data is obtained. For this study, these will include, but are not limited to, the Informed Consent Form, blood test results, and response letters from the general practitioner, laboratory records, diaries, medical records and correspondence. In this study, this will include, but is not limited to, medical history, medication records, vital signs, physical exam records, urine tests, blood test results, adverse event data and vaccine details. All source data and CRFs of the participants will be safely stored.

Where data are recorded directly onto the electronic data system these will be considered source documents. However, if local regulations require these electronic case report forms to be printed, they will be printed and filed in the participants

11.4 Data protection

The study protocol, documentation, data, and all other information generated will be kept strictly confidential. No information about the study or its data will be disclosed to unauthorized third parties without prior written approval from the sponsor.

Identifiable details, such as contact details, will be stored for a minimum of 5 years. Unidentified search data may be stored indefinitely. If participants agree to be contacted for future research, information about their Informed Consent Form will be recorded, retained and stored securely and separately from the research data.

11.5 Data quality

The data collection tools will undergo proper validation to ensure that the data is collected accurately and completely. The datasets provided for analysis will be subject to quality control processes to ensure that the data analyzed is a true reflection of the source data. Study data

will be managed in accordance with local data management SOPs. If an additional study of specific processes is necessary, an approved Data Management Plan will be implemented.

11.6 Archiving

Study data can be stored electronically on a secure server, and paper notes will be kept in a filing cabinet locked with a key in the site. All essential documents will be retained for a minimum of 5 years after the end of the study. The need to store study data for a longer time in relation to vaccine authorization will be subject to continuous review. For effective vaccines that can be authorized, we can safely store research data in the sites at least 15 years after the study ends, subject to adjustments to clinical trial regulations. Unidentified search data may be stored indefinitely.

12 QUALITY CONTROL PROCEDURES AND QUALITY ASSURANCE

12.1 Investigator's Procedures

The approved standard operating procedures (SOPs) will be used in the research sites and in all laboratory centers.

12.2Monitoring

Regular monitoring will be performed in accordance with the GCP by the monitor. When proceeding in accordance with the written SOPs, the monitor will verify that the clinical trial is performed and the data are generated, documented and reported in accordance with the protocol, GCP and applicable regulatory requirements. The sites will provide direct access to all data/documents and source reports related to the study for the purpose of monitoring and auditing by the Sponsor and to inspect by local and regulatory authorities.

12.3 Deviation from the protocol

Any deviations from the protocol will be documented on a protocol deviation form and filed in the study's master file.

Each deviation will be assessed for its impact on the safety of the participants and the conduct of the study. Significant deviations from the protocol will be listed at the end of the study report.

12.4 Audit and inspection

The QA manager conducts internal systems-based audits to verify that trials are being conducted in accordance with local procedures and in accordance with study protocols, departmental SOPs, GCP and applicable regulations.

The Sponsor, the study sites, the Research Ethics Committee, and the Regulatory Agencies may conduct audits to ensure compliance with the appropriate protocol, GCP, and regulations.

13 ETHICAL AND REGULATORY CONSIDERATIONS

13.1 Declaration of Helsinki

Investigators will ensure that this study is being conducted in accordance with the principles of the current revision of the Declaration of Helsinki.

13.2 Guidelines for good clinical practices

The Investigator will ensure that this trial is being conducted in accordance with the relevant standards and good clinical practices.

13.3 Ethical and regulatory approvals

After the Sponsor's approval, the Protocol, the Informed Consent Form, the participant's information sheet and any proposed advertising material will be submitted to an appropriate Research Ethics Committee (REC), regulatory authorities and host institutions for written approval. No changes to this protocol will be made without consulting the Sponsor and without its consent.

The Investigator is responsible for ensuring that changes in an approved study, during the period for which the approval of the Research Ethics Committee and Regulatory Agency has already been given, are not initiated without their review and approval, except to eliminate immediate risks apparent to the subject (i.e., as an urgent safety measure).

13.4Volunteers confidentiality

The study will comply with the EU General Data Protection Regulation (GDPR) and the UK Data Protection Act of 2018, as well as local data protection regulations, which require data not to be identified, whenever and when practical to do so. The processing of participants' personal data will be minimized by using only a single study number of the participant in all study documents and in any electronic database, with the exception of informed consent forms and participant identification records. All documents will be stored securely, and accessible only by study staff and authorized personnel. The study team will protect the privacy of participants' personal data. A separate confidential file containing personally identifiable information will be stored in a secure location in accordance with current data protection legislation. The photographs taken at the vaccination sites (if necessary, with the written and informed consent of the participant) will not include the face of the participant and will be identified by the date, study code and the subject's unique identifier. Once developed, the photographs will be stored as confidential records, as above. This material can be shown to other professionals, used for educational purposes, or included in a scientific publication.

If participants are diagnosed with COVID-19 during the course of the study, the study team will pass on their details to the local health protection team, if necessary, in accordance with the relevant notifiable disease legislation. Samples collected for the purpose of diagnosing COVID-19 can be sent to reference laboratories together with your personal data. This would be in line with national guidance and the policy of sending samples for testing in reference laboratories.

14 FUNDING AND INSURANCE

14.1 Funding

University of Oxford and external donors (Fundação Lemann, Fundacao Brava, Fundacao Telles, Instituto D'or de Ensino e Pesquisa and AstraZeneca Brasil).

14.2 Insurance

Global Insurance

Insured Party: University of Oxford

Research participants who suffer direct damage as a result of their participation in the study are entitled to claim compensation from the sponsor and the institutions involved in this study, covered by global and local insurance for research protocols.

14.3 Publication Policy

Researchers will be involved in reviewing draft manuscripts, abstracts, press releases and any other publications resulting from the study. The study data can also be used as part of a doctoral or master's thesis.

15 DEVELOPMENT OF A NEW PRODUCT/PROCESS OR INTELLECTUAL PROPERTY GENERATION

The IP title generated by University employees belongs to the University. The protection and exploitation of any new IP is managed by the University's technology transfer office, Oxford University Innovations. Researchers in this study can benefit from the University's royalty-sharing policy if new intellectual property is generated from the study. Several investigators are applicants or co-inventors of past patent registrations or patents related to ChAdOx1 vaccines. University of Oxford, which is a partner of Oxford University Hospitals NHS Foundation Trust at the NIHR Oxford Biomedical Research Centre, is committed to translational progress and the commercial development of healthcare products potentially serving medical and global healthcare needs and works and will work with business partners for these purposes.

References

1 Zhu, N. *et al.* A Novel Coronavirus from Patients with Pneumonia in China, 2019. *New England Journal of Medicine* **382**, 727-733, doi:10.1056/NEJMoa2001017 (2020).

2 Lu, R. *et al.* Genomic characterisation and epidemiology of 2019 novel coronavirus: implications for virus origins and receptor binding. *Lancet (London, England)* **395**, 565-574, doi:10.1016/S0140-6736(20)30251-8 (2020).

3 Li, F. Structure, Function, and Evolution of Coronavirus Spike Proteins. *Annual review of virology* **3**, 237-261, doi:10.1146/annurev-virology-110615-042301 (2016).

4 Zhou, P. *et al.* A pneumonia outbreak associated with a new coronavirus of probable bat origin. *Nature*, doi:10.1038/s41586-020-2012-7 (2020).

5 Alharbi, N. K. *et al.* ChAdOx1 and MVA based vaccine candidates against MERS-CoV elicit neutralising antibodies and cellular immune responses in mice. *Vaccine* **35**, 3780-3788, doi:10.1016/j.vaccine.2017.05.032 (2017).

6 Tseng, C. T. *et al.* Immunization with SARS coronavirus vaccines leads to pulmonary immunopathology on challenge with the SARS virus. *PloS one* **7**, e35421, doi:10.1371/journal.pone.0035421 (2012).

7 Weingartl, H. *et al.* Immunization with modified vaccinia virus Ankara-based recombinant vaccine against severe acute respiratory syndrome is associated with enhanced hepatitis in ferrets. *Journal of virology* **78**, 12672-12676, doi:10.1128/jvi.78.22.12672-12676.2004 (2004).

8 Liu, L. *et al.* Anti-spike IgG causes severe acute lung injury by skewing macrophage responses during acute SARS-CoV infection. *JCI insight* **4**, e123158, doi:10.1172/jci.insight.123158 (2019).

9 Agrawal, A. S. *et al.* Immunization with inactivated Middle East Respiratory Syndrome coronavirus vaccine leads to lung immunopathology on challenge with live virus. *Human vaccines & immunotherapeutics* **12**, 2351-2356, doi:10.1080/21645515.2016.1177688 (2016).

10 Munster, V. J. *et al.* Protective efficacy of a novel simian adenovirus vaccine against lethal MERS-CoV challenge in a transgenic human DPP4 mouse model. *NPJ vaccines* **2**, 28, doi:10.1038/s41541-017-0029-1 (2017).

11 Alharbi, N. K. *et al.* Humoral Immunogenicity and Efficacy of a Single Dose of ChAdOx1 MERS Vaccine Candidate in Dromedary Camels. *Scientific reports* **9**, 16292, doi:10.1038/s41598-019-52730-4 (2019).

12 Antrobus, R. D. *et al.* Clinical assessment of a novel recombinant simian adenovirus ChAdOx1 as a vectored vaccine expressing conserved Influenza A antigens. *Mol Ther* **22**, 668-674, doi:10.1038/mt.2013.284 (2014).

13 Coughlan, L. *et al.* Heterologous Two-Dose Vaccination with Simian Adenovirus and Poxvirus Vectors Elicits Long-Lasting Cellular Immunity to Influenza Virus A in Healthy Adults. *EbioMedicine*, doi:https://doi.org/10.1016/j.ebiom.2018.02.011 (2018).

14 Wilkie, M. *et al.* A phase I trial evaluating the safety and immunogenicity of a candidate tuberculosis vaccination regimen, ChAdOx1 85A prime – MVA85A boost in healthy UK adults. *Vaccine* **38**, 779-789, doi:10.1016/j.vaccine.2019.10.102 (2020).

15 Modjarrad, K. MERS-CoV vaccine candidates in development: The current landscape. *Vaccine* **34**, 2982-2987, doi:10.1016/j.vaccine.2016.03.104 (2016).

16 She, J., Liu, L. & Liu, W. COVID-19 epidemic: Disease characteristics in children. *J Med Virol*, doi:10.1002/jmv.25807 (2020).

17 Dong, Y. *et al.* Epidemiology of COVID-19 Among Children in China. *Pediatrics*, doi:10.1542/peds.2020-0702 (2020).

18 Ludvigsson, J. F. Systematic review of COVID-19 in children shows milder cases and a better prognosis than adults. *Acta Paediatr*, doi:10.1111/apa.15270 (2020).

19 Zimmermann, P. & Curtis, N. Coronavirus Infections in Children Including COVID-19: An Overview of the Epidemiology, Clinical Features, Diagnosis, Treatment and Prevention Options in Children. The Pediatric Infectious Disease Journal 39, 355-368, doi:10.1097/inf.0000000000002660 (2020).

20. _ Baxter D, et al. Safety and immunogenicity of seven COVID-19 vaccines as a third dose (booster) following two doses of ChAdOx1 nCov-19 or BNT162b2 in the UK (COV-BOOST): a blinded, multicentre, randomised, controlled, phase 2 trial. The Lancet, 398(10318):2258-2276. doi: https://doi.org/10.1016/S0140-6736(21)02717-3 (2021)

21. Nota técnica Nº 20/2022 -SECOVID/GAB/SECOVID/MS. Recomendação da segunda dose de reforço de vacinas contra Covid-19 em pessoas com 80 anos ou mais. Ministério da saúde; 30/03/2022.

22. Regev-Yochay, G, Gonen, T, Gilboa M, et al. 4th dose COVID m RNA vaccines' immunogenicity & efficacy against omicron VOC. medRix preprint 25 Feb 2022, doi: https://doi.org/10.1101/2022.02.15.22270948.

23.YinonM. BarOn, Yair Goldberg, Micha Mandel, Omri Bodenheimer, Ofra Amir, Laurence Freedma n, Sharon Alroy-Preis, Nachman Ash, Amit Huppert, Ron Milo. Protection by 4th dose of BNT162b2 against Omicron in Israel. medRix preprint 01 Feb 2022, doi: https://doi.org/10.1101/2022.02.01.22270232

APPENDIX A: Adverse Events of Special Interest

Neuroinflammatory disorders	• Cranial nerve neuropathy, including palsy and paresis (eg, Bell's palsy).<br>• Optic neuritis.<br>•Multiple sclerosis.<br>• Transverse myelitis.<br>• Guillain-Barré syndrome, including Miller Fisher syndrome and other variants.<br>• Acute disseminated encephalomyelitis, including site-specific variants, eg: non-infectious encephalitis, encephalomyelitis, myelitis, myeloradiculoneuritis.<br>• Myasthenia gravis, including Lambert-Eaton myasthenic syndrome.<br>• Peripheral demyelinating neuropathies, including:<br>- Chronic inflammatory demyelinating polyneuropathy.<br>- Multifocal motor neuropathy.<br>• Polyneuropathies associated with monoclonal gammopathy.<br>• Narcolepsy.
Musculoskeletal disorders	• Systemic lupus erythematosus and associated conditions.<br>• Systemic scleroderma (systemic sclerosis), including:<br>- Diffuse scleroderma.<br>- Calcinosis, Raynaud's phenomenon, esophageal dysmotility, sclerodactyly and telangiectasia syndrome (CREST).<br>• Idiopathic inflammatory myopathies, including:<br>- Dermatomyositis.<br>- Polymyositis.<br>• Anti-synthetase syndrome.<br>• Rheumatoid arthritis and associated conditions, including:<br>- Juvenile idiopathic arthritis.<br>- Still's disease.<br>• Polymyalgia rheumatica.<br>• Spondyloarthropathies, including:<br>-Ankylosing spondylitis.<br>- Reactive arthritis (Reiter's syndrome).<br>- Undifferentiated spondylarthritis.

	- Psoriatic arthritis.<br>- Enteropathic arthritis.<br>• Recurrent polychondritis.<br>• Disorder of mixed connective tissue.<br>• Gout
Skin disorders	• Psoriasis.<br>• Vitiligo.<br>•Erythema nodosum.<br>• Autoimmune bullous skin diseases (including pemphigus, pemphigoid, and dermatitis herpetiformis).<br>• Lichen planus.<br>• Sweet's syndrome.<br>• Localized scleroderma (morphea).
Vasculitis	• Large vessel vasculitis including:<br>- Giant cell arteritis (temporal arteritis).<br>- Takayasu's arteritis.<br>• Vasculitis of medium and/or small vessels, including:<br>- Polyarteritis nodosa.<br>- Kawasaki disease.<br>- Microscopic polyangiitis.<br>- Wegener's granulomatosis (granulomatosis with polyangiitis).<br>- Churg-Strauss syndrome (allergic granulomatous angiitis or eosinophilic granulomatosis with polyangiitis).<br>- Buerger's disease (thromboangiitis obliterans).<br>- Necrotizing vasculitis (cutaneous or systemic).<br>- Vasculitis positive for antineutrophil cytoplasmic antibody (type unspecified).<br>- Henoch-Schonlein purpura (immunoglobulin A vasculitis).<br>- Behcet's syndrome.<br>- Leukocytoclastic vasculitis.
Blood disorders	• Autoimmune hemolytic anemia.<br>• Autoimmune thrombocytopenia.<br>• Antiphospholipid syndrome.<br>• Pernicious anemia.<br>• Autoimmune aplastic anemia.

	• Autoimmune neutropenia.<br>• Autoimmune pancytopenia.<br>• Anti-platelet antibodies<br>• Vascular thrombosis<br>• Stroke
Liver disorders	• Autoimmune hepatitis.<br>• Primary biliary cirrhosis.<br>• Primary sclerosing cholangitis.<br>• Autoimmune cholangitis.
Gastrointestinal Disorders	• Inflammatory bowel disease, including:<br>-Crohn's disease.<br>- Ulcerative colitis.<br>- Microscopic colitis.<br>- Ulcerative proctitis.<br>•Celiac disease.<br>• Autoimmune pancreatitis.
Endocrine Disorders	• Autoimmune thyroiditis (Hashimoto's thyroiditis).<br>• Grave's or Basedow's Disease.<br>• Type 1 diabetes mellitus.<br>• Addison's Disease.<br>• Polyglandular autoimmune syndrome.<br>• Autoimmune hypophysis.
Others	• Autoimmune glomerulonephritis including:<br>- Immunoglobulin A nephropathy.<br>- Rapidly progressive glomerulonephritis.<br>- Membranous glomerulonephritis.<br>- Membranoproliferative glomerulonephritis.<br>- Mesangioproliferative glomerulonephritis.<br>- Tubulointerstitial nephritis and uveitis syndrome.<br>• Autoimmune eye diseases, including:<br>- Autoimmune uveitis.<br>- Autoimmune retinitis.

	• Autoimmune myocarditis.<br>• Sarcoidosis.<br>• Stevens-Johnson syndrome.<br>• Sjögren's Syndrome.<br>• Alopecia areata.<br>• Idiopathic pulmonary fibrosis.<br>• Goodpasture's syndrome.<br>• Raynaud's phenomenon.

APPENDIX B AMENDMENTS HISTORY

Amendment No.	Version of the protocol No.	Date issued	Amendment author(s)	Details of Amendments made
N/A	1.0	May 27th, 2020	N/A	First version
1	1.0	May 31st, 2020	PPD	Ethical requirements of the Brazilian Ethics Committee/CONEP system
2	2.0	June 10th, 2020	PPD<br>PPD	Ethical requirements of the English Ethics Committee (OxTREC) – updated data according to IB 6.0
3	3.0	June 14th, 2020	PPD<br>PPD	ANVISA requests, updating of participating centers, electronic ICF, correction of sample size and exclusion of assistance to pregnant partners of research participants.

Amendment No.	Version of the protocol No.	Date issued	Amendment author(s)	Details of Amendments made
4	4.0	July 28th, 2020	PPD	Added booster groups. Removed requirement for negative COVID-19 serology prior to enrollment; Added details on COBRA batch; clarified process around data entry/source data; updated statistical analysis section to reflect changes in trial procedures (e.g. addition of booster doses); added cellular immune responses in a subset of individuals as exploratory objective; added clarifications to swabbing procedures; added Hy's law cases as part of the requirement for SAE reporting; added clarifications to DSMB composition and their role in advising stopping recruitment.
5	4.1	August 11th, 2020	PPD<br>PPD	Clarify boosting dose windows; include placebo as comparator; update abbreviations; clarify the cellular immune response volunteers selection; clarify study groups; clarify non-hospitalized volunteers shall not take part in covid-19 treatment clinical trials; clarify serology criteria

Amendment No.	Version of the protocol No.	Date issued	Amendment author(s)	Details of Amendments made
				before/after protocol 4.0; update table 4
6	5.0	August 16th, 2020	PPD	Increased sample size to up to 10,000; Changes to statistical analysis section, including changes to how the primary endpoint will be analysed; changes to the symptomatic pathway; clarifications to the inclusion and exclusion criteria
7	6.0	September 30th 2020	PPD	Increased time from vial piercing to vaccine administration from 4 to 6 hours; clarifications to inclusion and exclusion criteria and removal of one of the exclusion criteria (participation in serological surveys); clarification and minor changes to symptomatic pathway in line with clinical study plan (requirements on visit S3-5, diaries, COVID-19 hospitalisation data collection); clarifications to vital signs collected at different timepoints; additional funders;
8	7.0	October 29th 2020	PPD	Increase in sample size to up to 10.300 people to account for the competitive and simultaneous

Amendment No.	Version of the protocol No.	Date issued	Amendment author(s)	Details of Amendments made
				recruitment strategy at multiple sites.
9	8.0	12 Nov 2020	PPD	Clarifications to vital signs and physical measurements required at different visits; Updated Statistical Analysis section in line with global programme and with references to study level SAP and a separate SAP for Marketing Authorisation Application<br><br>Increased number of subset of individuals for CMI assessment from 50 to 60.
10	9.0	15 Jan 2021	PPD	Described procedures for study unblinding following country roll out of COVID-18 vaccines .<br><br>Only SAEs and AESI to be recorded following unblinding and vaccination of those in the control arm.<br><br>Increased use of vaccine from first vial puncture from 6 to 48 hours when vial stored at 2-8°C

11.	10	21 Jan 2021	PPD	Sequencing of SARS-CoV-2 added to study procedures and tertiary endpoints<br>Details on efficacy analysis for unblinding<br>Unblinding procedures
12	11	28 April 2021	PPD	Co-Participant Sites inclusion<br>Exclusion criteria updated with the exception of the licensed seasonal influenza vaccination and the licensed pneumococcal vaccination.<br>Secondary objectives including assessment of immunological correlates<br>Changes to symptomatic participant follow up (to reduce study procedure burden on participants and simplify safety data collection):<br>If there is already a negative PCR test outside of the trial during a symptomatic episode then no need to bring participant in for repeat swab<br>If there is a positive PCR test outside of the trial + symptoms then still bring in for repeat swab and immunology bloods (no safety bloods, observations or examination needed)

				No repeat swab at S3-S5 (A single negative PCR test in or out of the trial is<br><br>now sufficient to declare it a negative case)<br><br>Replace S7 physical visit with a remote phone call<br><br>Removal of symptomatic ediary<br><br>Removal of S3-5 repeat swab/visit<br><br>Immunological correlate of protection endpoint added<br><br>Change to exclusion criteria to permit participants to receive licensed seasonal influenza and pneumococcal vaccines from 14 days post vaccination<br><br>Unblinded exploratory COVID-19 disease endpoints may be determined by trained trial team members (rather than blinded independent endpoint assessors)
13	12	08 June 2021	PPD	Inclusion of third dose of ChAdOx1 nCoV-19 for subgroup 1 – which received 2 doses of ChAdOx1 nCoV-19 vaccine 4 weeks apart, 11-13 months after the second dose.

				Evaluation of immunogenicity and reactogenicity of the third dose.<br><br>Group 2 – inclusion of the third dose for a subgroup of 350 participants who received 2 doses of ChAdOx1 nCoV-19 12 weeks apart, 5-7 months after the second dose.<br><br>Evaluation of immunogenicity and reactogenicity of the third dose.<br><br>Groups 3 and 4 – evaluation of immunogenicity and reactogenicity of the third dose with ChAdOx1 nCoV-19 vaccine or with Sinovac/Butantan vaccine in a subgroup of 600 volunteers who previously received 2 doses of Sinovac/Butantan with an interval of 2-4 weeks.
14	12.1	29 June 2021	PPD	Inclusion of the third dose with ChAdOx1 nCoV-19 for the subgroup of participants who received 2 doses of the ChAdOx1 nCoV-19 vaccine with a 4-week interval, 11-13 months after the second dose.<br><br>Evaluation of immunogenicity and reactogenicity of the third dose.

15	12.2	19 July 2021	PPD	Inclusion of paracetamol after vaccination.<br><br>Interval of 4 to 12 weeks between the first and the second dose.<br><br>End of the third dose on October 31, 2021.<br><br>7-day diary for a subset of 100 participants.
16	13.0	08 Mar 2022	PPD	Inclusion of a fourth dose booster in participants from group 1e, who previously received two doses of ChAdOx1 nCov-19, followed by a third late dose of ChAdOx1 nCOV-19, regardless of interval between the third and fourth dose.<br><br>Evaluation of immunogenicity and reactogenicity of the fourth dose.
17	13.1	18 July 2022	PPD	Clarification that the interval between the third and the fourth dose of ChAdOx1 nCOV-19 shall be of 6 to 15 months.<br><br>Clarification that the secondary objective “To evaluate the reactogenicity profile of the fourth dose” will address occurrence of signs and symptoms of local and systemic reactogenicity requested during 7 days after vaccination.

				Clarification that the following exclusion criteria: *clinically significant bleeding (eg, factor deficiency, coagulopathy, or platelet disorder), or prior history of significant bleeding or bruising following intramuscular injections or venepuncture,* also includs Capillary Leakage Syndrome.<br><br>Update of the formatting of the objectives table to clarify that for the third dose, immunogenicity and reactogenicity will be evaluated.<br><br>Clarification that having the immunogenicity results is an expectation of the population that participates in clinical trials in Brazil, as part of their participation in such studies.
18	14.0	21 August 2022	PPD	Update of a fourth dose exclusion criteria based on previous COVID-19 infection, limiting it to self-reported confirmed infections in the last four weeks.<br><br>Clarification that only the maximum severity grade of hospitalized patients will be recorded.<br><br>Update on 4th dose objective if the number of participants seronegative at baseline, intra-subject correlation and the

				observed standard derivation combine to provide ≥ 80% power for non-inferiority assessments, the analysis will be performed as planned. Alternatively, if any factor or combination of factors results in <80% power, the primary analysis will be descriptive immunogenicity only, with all comparative analyses being exploratory, including any subgroup comparison. The details will be described in the SAP.<br><br>Clarification that the 4th dose inclusion criteria *"Study visits blood samples for visits to date must have been collected within visit windows"* will contemplate, participants who had their PB28 visit bloods within 28 – 35 days post vaccine, and who had their 28 days post third dose blood within 28-35 days post third vaccine.<br><br>Clarification that up to 55ml of blood may be collected during visits 8, 9 and 10 as IgG to SARS-CoV-2 will be performed in a National Laboratory.<br><br>Clarification that immunological analysis will also be performed in a collaborating national laboratory.<br><br>Subtitution of vaccines' supplier from AstraZeneca/Fiocruz to AstraZeneca/Halix, and increased the vial size to 10 vaccines per unit.

				Clarification that the biorepository storage for future use can be in Brazil or abroad.<br>Clarification that in accordance with Brazilian law, we do not store participants' relevant bank details.

List details of all protocol amendments here whenever a new protocol version is produced.

PPD

Final Audit Report 2022-09-08

Created:	2022-09-08
By:	PPD
Status:	Signed
Transaction ID:	PPD

"PPD" History

Document created by PPD
2022-09-08 - 1:09:42 PM GMT

Document emailed to PPD for signature
2022-09-08 - 1:10:47 PM GMT

Email viewed by PPD
2022-09-08 - 2:50:10 PM GMT

Signer PPD entered name at signing as PPD
2022-09-08 - 2:51:17 PM GMT

Document e-signed by PPD
Signature Date: 2022-09-08 - 2:51:18 PM GMT - Time Source: server

Agreement completed.
2022-09-08 - 2:51:18 PM GMT
